# Benchmarking DFT for Chain-Length and Water Effects in Thermal Conia–Ene Cyclization

**DOI:** 10.1021/acsomega.6c05834

**Published:** 2026-08-18

**Authors:** Jorge Gutiérrez-Flores, Eduardo H. Huerta, Javier Serrano Medina, Jorge Garza, Marcos Hernández-Rodriguez, Rubicelia Vargas

**Affiliations:** † Departamento de Química, División de Ciencias Básicas e Ingeniería, 428230 Universidad Autónoma Metropolitana Iztapalapa, San Rafael Atlixco 186, Col. Vicentina, Iztapalapa, Ciudad de México C.P. 09340, México; ‡ Universidad Nacional Autónoma de México, 7180 Instituto de Química, Circuito Exterior, Ciudad Universitaria, Alc. Coyoacán, Ciudad de México 04510, México

## Abstract

Reliable density functional theory (DFT) methods for thermally driven cyclization reactions remain insufficiently established, particularly for systems in which proton transfer, conformational preorganization, and weak environmental effects jointly shape the energy landscape. Here, we present a systematic benchmark of DFT exchange–correlation functional/basis-set combinations against CCSD­(T) reference energies for the thermal Conia–Ene cyclization, using chain length and explicit water assistance as chemically relevant probes of functional performance. Representative reaction profiles were constructed for two model substrates (hex-5-ynal and hept-6-ynal), considering pathways with and without explicit water, in both gas phase and implicit solvent. The reaction proceeds stepwise through enolization followed by cyclization, with enolization defining the intrinsic kinetic bottleneck in the water-free pathway (ca. 70 kcal mol^–1^). Explicit water selectively lowers this barrier by up to 30 kcal mol^–1^ through proton-transfer mediation, whereas the cyclization barrier is primarily controlled by chain length through transition-state preorganization; implicit solvation has only a minor energetic effect. Across 38 approximate exchange–correlation functionals and 14 basis sets, range-separated hybrids provide the most accurate and balanced description of both activation barriers and reaction energies. In particular, ωB97XD and CAM-B3LYP-D3BJ achieve near-chemical accuracy, with mean absolute errors close to 1 kcal mol^–1^ when combined with triple-ζ basis sets containing polarization and diffuse functions. Comparable accuracy is also achieved by the double-hybrid functionals mPW2PLYP and DSD-PBEP86, albeit at a substantially higher computational cost. Overall, this study establishes a practical CCSD­(T)-referenced benchmark and identifies robust DFT protocols for modeling thermal Conia–Ene cyclizations, with expected transferability to more complex substrates and confined environments.

## Graphical abstract

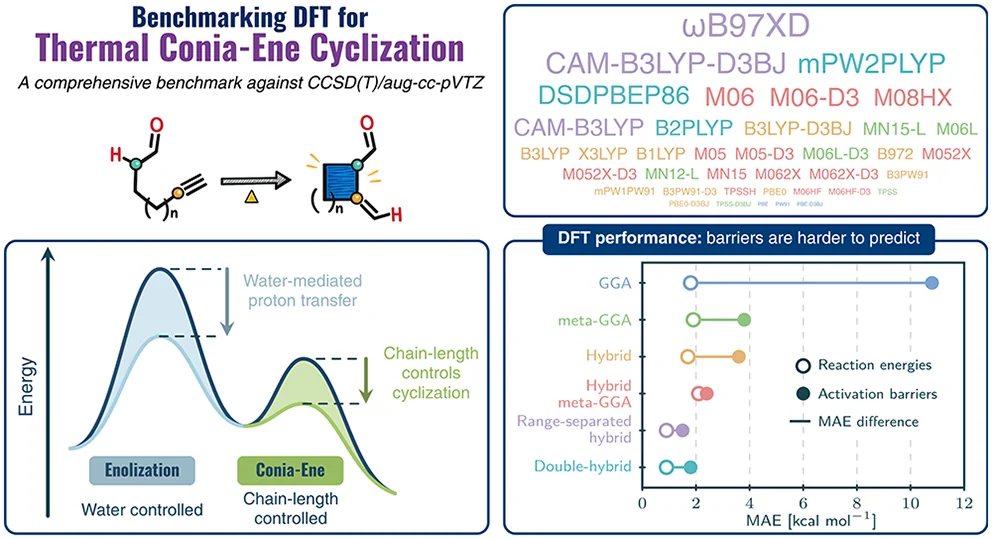

## 1 Introduction

The Conia–Ene cyclization constitutes a highly atom-economical intramolecular C–C bond-forming transformation that enables access to both carbo- and heterocyclic frameworks from unsaturated, enolizable carbonyl compounds (Figure 1a).^1^ Owing to its efficiency, this reaction has become a strategic tool for the construction of complex cyclic architectures,^2–5^ for the total synthesis of natural products such as alkaloids and terpenoids,^6–9^ and for the development of asymmetric variants.^10^ However, beyond its synthetic value, the inherent instability of enol tautomers, together with the low α-proton acidity of typical carbonyl precursors, renders the reaction unfeasible at room temperature, thus requiring temperatures above 300 °C to proceed (Figure 1b).^11,12^ These restrictive conditions significantly limit the scope of the thermal Conia–Ene reaction, particularly for sensitive substrates,^13^ and consequently underscore the need for a deeper mechanistic understanding and improved strategies to boost its reactivity.

To overcome these intrinsic limitations, some activation strategies have been developed to increase the accessibility of the reactive intermediate and to promote its preorganization. These can be broadly classified into three approaches (Figure 1c, left): (i) enhancement of enolization (e.g., β-dicarbonyl systems, Lewis acid assistance, or strong non-nucleophilic bases);^14,15^ (ii) activation of the alkyne through increased electrophilicity via transition-metal coordination;^16–18^ and (iii) dual-activation strategies involving one or two metal centers.^19–21^ While these methods have expanded the synthetic scope, they often rely on substrate preactivation or costly metal catalysis, which ultimately limits their generality. Despite these advances, alternative activation pathways remain largely unexplored, particularly those arising from nonclassical effects such as dispersion-assisted stabilization by dispersion energy donor (DED) substituents,^22–24^ as well as from molecular confinement, where spatial restriction and enhanced host–guest interactions can stabilize transition states and modulate the reaction pathway (Figure 1c, right).^25–27^ In principle, both factors could lower the barrier by stabilizing reactive configurations, modulating the sensitivity of the barrier to the thermodynamic driving force, and promoting favorable organization along the reaction coordinate. However, the extent to which these effects reshape the Conia–Ene energy landscape remains not studied, highlighting the need for a theoretical framework capable of rigorously assessing their feasibility.

**Figure 1:**
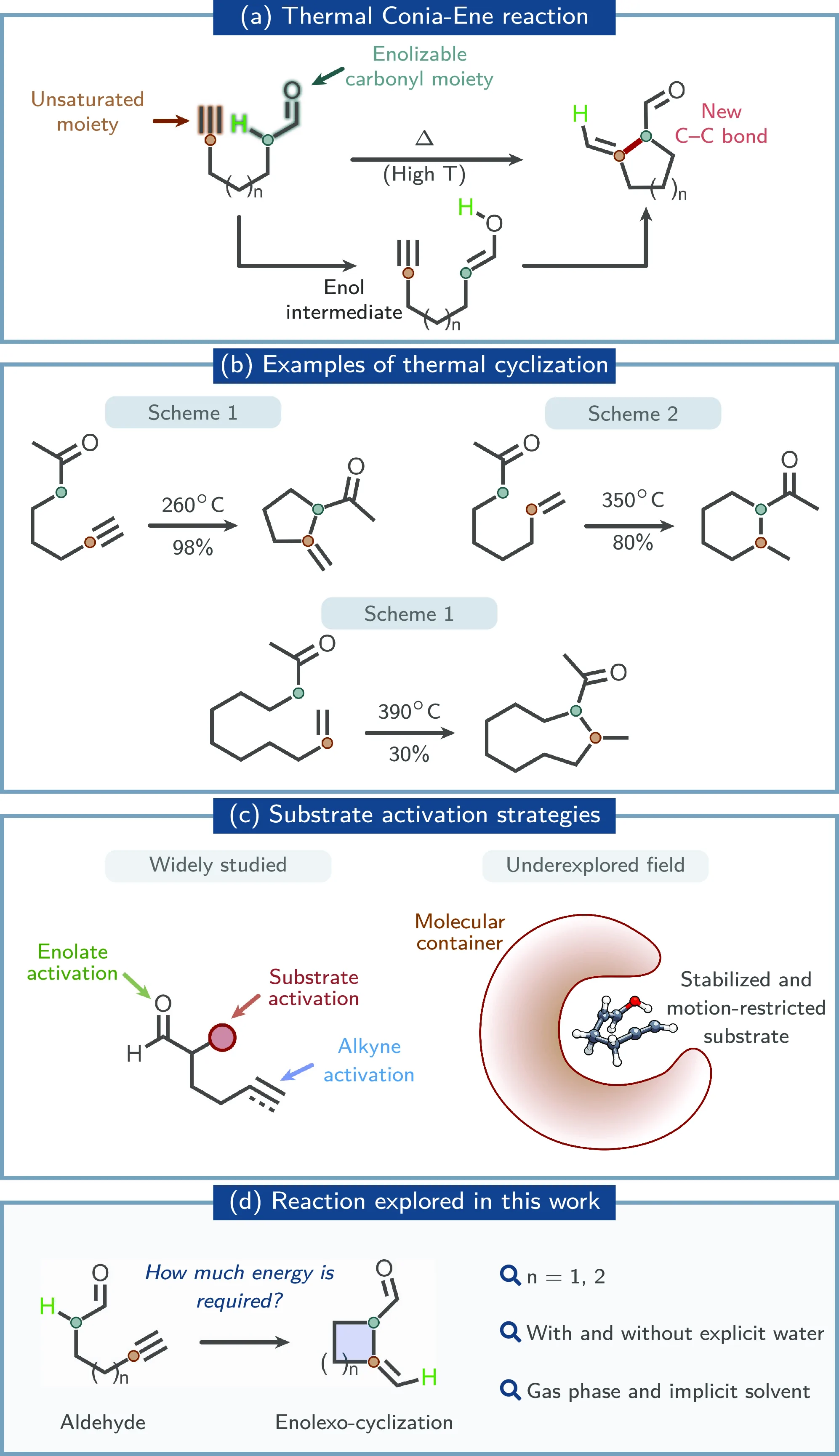
Conia-Ene cyclization, examples, activation strategies, and reaction explored in this work.

Experimental access to tailored substrates and confined environments, and the difficulty in quantifying their effects on reaction thermodynamics and kinetics, hinders the systematic exploration of alternative activation pathways for the Conia–Ene reaction. In this context, computational methods provide a valuable framework for generating reliable predictions. From a theoretical standpoint, an accurate description of the reaction, particularly its energetics, requires an explicit treatment of electronic correlation, which is essential for capturing both thermodynamic and kinetic parameters in proton-transfer processes. Accordingly, post-Hartree–Fock methods that include correlation effects beyond second-order perturbation theory are typically required to obtain reliable barrier heights and potential energy surfaces,^28,29^ with coupled-cluster (CC) methods providing high-level descriptions of reaction profiles.^30^ However, their computational cost severely limits their applicability to relatively small systems, excluding many chemically relevant scenarios, especially those involving complex or confined environments. As a result, Density Functional Theory (DFT) has emerged as a practical alternative, offering a more favorable balance between accuracy and efficiency. Nevertheless, its reliability depends critically on the choice of exchange–correlation functional^31,32^ and basis set.^33,34^ Given the diversity of density functional approximations, their performance must be systematically assessed against higher-level methods or experimental data. Additionally, the basis-set influence must also be evaluated to ensure a consistent description of reaction energetics. To the best of our knowledge, such systematic assessments have not been reported for thermal Conia–Ene reactions, underscoring the need for a validated, computationally affordable theoretical framework to enable broad exploration of the electronic and environmental factors that alleviate the high activation barriers.

This work presents a systematic benchmark of DFT functional/basis-set combinations against CCSD­(T) for the description of activation barriers and reaction energies in thermal Conia–Ene cyclizations, using chain length and explicit water assistance as chemically distinct probes of functional performance. The assessment is carried out on a representative set of reaction profiles spanning two substrates (hex-5-ynal and hept-6-ynal), the presence or absence of explicit water, and the environment (gas phase and implicit solvent), Figure 1d. Reference geometries are obtained at the CCSD level, while reference energies are taken from high-level CCSD­(T) calculations. A total of 38 density functionals, ranging from GGAs, metaGGAs, hybrids, hybrid metaGGAs, range-separated hybrids, to double-hybrids, including dispersion-corrected variants, are evaluated in combination with 14 basis sets. The primary objective is to identify functional/basis-set combinations that reliably reproduce the kinetic and thermodynamic features of the reaction while maintaining a computational cost suitable for larger systems; mechanistic analysis is used to establish chemically meaningful benchmark profiles and to interpret the response of the energy landscape to chain length and water assistance. The resulting protocol is intended to be transferable to both free and confined conditions, providing a practical basis for DFT-based studies of thermally activated cyclizations in more complex environments.

## 2 Computational Methods

The thermal Conia–Ene cyclization of alkynyl aldehydes, specifically hex-5-ynal (1a) and hept-6-ynal (1b), was investigated considering two mechanistic models: (i) in the absence of explicit water and (ii) including one explicit water molecule acting as a potential proton relay. These substrates were chosen as the shortest chain lengths that enable the targeted cyclization that reproduce the experimental preference for five-membered exocyclizations, while avoiding the substantial increase in computational cost associated with longer methylene chains at the high-level methods employed. All calculations were performed in the gas phase and within a polarizable continuum model to account for bulk solvation effects, employing the SMD model^35^ with water as solvent. The potential energy surface (PES) was explored using coupled-cluster theory with single and double excitations (CCSD)^36–40^ in conjunction with the 6–31G­(d,p) basis set.^41–43^ All stationary points were fully optimized without symmetry constraints, and harmonic vibrational frequency analyses were carried out at the same level of theory to confirm their nature: minima were characterized by the absence of imaginary frequencies, whereas transition states exhibited a single imaginary frequency. The associated normal mode was inspected to ensure proper connection along the reaction coordinate. To refine the energetic profile, single-point electronic energies were computed on the CCSD/6–31G­(d,p) optimized geometries using canonical coupled-cluster theory including single, double, and perturbative triple excitations, CCSD­(T),^36–40,44^ combined with the aug-cc-pVTZ basis set. CCSD­(T)/aug-cc-pVTZ single-point energies were adopted as a uniform high-level reference throughout this benchmark for assessing the performance of exchange–correlation functionals (XC). Although these values may retain residual basis-set incompleteness error, they were not intended to represent the complete-basis-set (CBS) limit. A reliable CBS extrapolation would require additional calculations employing larger correlation-consistent basis sets.^45–47^ Rather than extrapolating from relatively low-cardinality basis sets, the directly computed aug-cc-pVTZ energies were retained for all stationary points to preserve a consistent reference framework throughout the study.

Upon these reference geometries, single-point electronic energies were computed using a broad set of density functional approximations (DFAs) in combination with the aug-cc-pVTZ basis set,^80,81^ denoted as XC/aug-cc-pVTZ//CCSD/6–31G­(d,p). The complete list of exchange–correlation functionals is provided in Table 1. The selected DFAs span multiple rungs of Jacob’s ladder, including GGA, metaGGA, hybrid, hybrid metaGGA, range-separated, and double-hybrid functionals, with selected cases incorporating empirical dispersion corrections. This diverse selection enables a systematic assessment of how different levels of theoretical sophistication capture the key electronic effects governing the reaction. All DFA calculations were carried out under the same gas-phase and solvation conditions described above, ensuring direct comparability with the reference CCSD­(T) energetic framework.

**Table 1:** Exchange-Correlation DFAs Considered in This Benchmark Study

name	exact exchange	dispersion correction	refs
GGA			
PBE	N.A.	None	^48,49^
PBE-D3(BJ)	N.A.	D3(BJ)	^48–50^
PW91	N.A.	None	^51,52^
MetaGGA			
M06L	N.A.	None	^53^
M06L-D3	N.A.	D3	^53,54^
MN12-L	N.A.	None	^55^
MN15-L	N.A.	None	^56^
TPSS	N.A.	None	^57^
TPSS-D3(BJ)	N.A.	D3(BJ)	^50,57^
Hybrid			
B3LYP	20%	None	^58–60^
B3LYP-D3(BJ)	20%	D3(BJ)	^50,58–60^
PBE0	25%	None	^61^
PBE0-D3(BJ)	25%	D3(BJ)	^50,61^
B3PW91	20%	None	^51,52,59^
B3PW91-D3	20%	D3	^51,52,54,59^
X3LYP	21.8%	None	^62^
B1LYP	25%	None	^63^
mPW1PW91	25%	None	^64^
B972	21%	None	^65^
Hybrid MetaGGA			
MN15	44%	None	^66^
M08HX	52.23%	None	^67^
M06	27%	None	^68^
M06-D3	27%	D3	^54,68^
M06HF	100%	None	^69^
M06HF-D3	100%	D3	^54,69^
M062X	54%	None	^68^
M062X-D3	54%	D3	^54,68^
M05	28%	None	^70^
M05-D3	28%	D3	^54,70^
M052X	56%	None	^71^
M052X-D3	56%	D3	^54,71^
TPSSh	10%	None	^72,73^
Range-Separated Hybrid^a^			
CAM-B3LYP	19%, 65%	None	^74^
CAM-B3LYP-D3(BJ)	19%, 65%	D3(BJ)	^50,74^
ωB97XD	22.2%, 100%	D2	^75,76^
Double-Hybrid^b^			
B2PLYP	53%	None	^77^
mPW2PLYP	55%	None	^78^
DSD-PBEP86	70%	D3(BJ)	^50,79^

a The min, max for range separated hybrids.

b Double-hybrid functionals incorporate a MP2 correlation contribution. The fraction of the MP2 correlation energy included is 27% (B2PLYP), 25% (mPW2PLYP), and 100% (DSD-PBEP86).

To assess the influence of the basis set, additional calculations were performed for each DFA using a variety of basis sets listed in Table 2. These sets were selected from widely used families in computational chemistry, including Pople, Dunning, Ahlrichs, and Jensen basis sets, spanning a broad range of basis-function sizes and polarization and diffuse-function levels. To isolate intrinsic electronic effects and ensure consistent comparisons across basis sets, all calculations in this stage were performed exclusively in the gas phase. In all cases, the reference energy used for comparison was that obtained at the CCSD­(T)/aug-cc-pVTZ level, ensuring consistency with the benchmark energetic framework established in this work.

**Table 2:** Basis Sets Employed in This Benchmark Study^a^

basis set	zeta	polarization functions	diffuse functions	refs
Pople				
6–31G	Double	No	No	^41,42^
6–31G(d,p)	Double	Yes	No	^41–43^
6–31+G(d,p)	Double	Yes	Yes	^41,42,82^
6–311G(d,p)	Triple	Yes	No	^83^
6–311++G(2d,2p)	Triple	Yes	Yes	^82,83^
Dunning				
cc-pVDZ	Double	Yes	No	^80^
aug-cc-pVDZ	Double	Yes	Yes	^80,81^
cc-pVTZ	Triple	Yes	No	^80^
aug-cc-pVTZ	Triple	Yes	Yes	^80,81^
Ahlrichs				
def2-SVP	Double	Yes	No	^84^
def2-TZVP	Triple	Yes	No	^84^
def2-TZVPP	Triple	Yes	No	^84^
Jensen				
pc-2	Triple	Yes	No	^85,86^
pc-3	Quadruple	Yes	No	^85,86^

a Polarization functions are typically added to heavy atoms (*d*-functions) and hydrogen atoms (*p*-functions), depending on the definition of each basis set. Diffuse functions are applied only to heavy atoms in single-signed sets (+), and to all atoms (including hydrogen atoms) in double-signed sets (++ or aug-). For the cc-pVnZ, def2, and pc-*n* families, polarization functions are incorporated into the original basis set construction.

The performance of each XC/basis set combination was evaluated through a systematic comparison of the computed activation barriers and reaction energies against the reference CCSD­(T)/aug-cc-pVTZ//CCSD/6–31G­(d,p) values. For the full set of 38 functionals and 14 basis sets, statistical metrics including the mean error (ME), mean absolute error (MAE), maximum unsigned error (MUE), and standard deviation (SD) were determined for activation energies, reaction energies, and their combined data set. This analysis provides a quantitative framework for assessing the accuracy and robustness of different methodological choices, offering practical guidance for selecting computational approaches that balance predictive reliability and computational cost to meet the requirements of the system under study.

All electronic structure calculations were carried out using the Gaussian16 software package.^87^

## 3 Results and Discussion

### 3.1 Mechanistic Analysis of the Reaction Energy Profile

To establish a chemically diverse and CCSD­(T)-referenced benchmark set for assessing DFA performance, the thermal Conia–Ene transformation was first characterized at the mechanistic level. The reaction proceeds through a two-step pathway: an initial enolization followed by cyclization. A schematic energy profile is shown in Figure 2a as a general mechanistic illustration, and it defines the activation barriers and reaction energies for each elementary step employed throughout this study. In the first step, the aldehyde form (1) undergoes enolization via TS_enol_ to yield the enol intermediate (2); this process involves α-proton transfer from C3 to O1 accompanied by π-electron reorganization that forms the C2=C3 bond. The resulting enol establishes the reactive configuration required for the subsequent transformation. In the second step, the Conia–Ene cyclization proceeds through TS_conia_ and involves concerted C–C bond formation between the enolic carbon (C3) and the alkyne carbon (C4), together with allylic hydrogen transfer from O1 to C5, to afford the cyclized product (3). For hex-5-ynal (1a; *n* = 1) and hept-6-ynal (1b; *n* = 2), this step yields 2-methylenecyclobutane-1-carbaldehyde (3a) and 2-methylenecyclopentane-1-carbaldehyde (3b), respectively, consistent with the revised Baldwin’s rules for ring closure.^88^ The optimized geometries of all intermediates are provided in Section 6 of the Supporting Information (SI).

**Figure 2:**
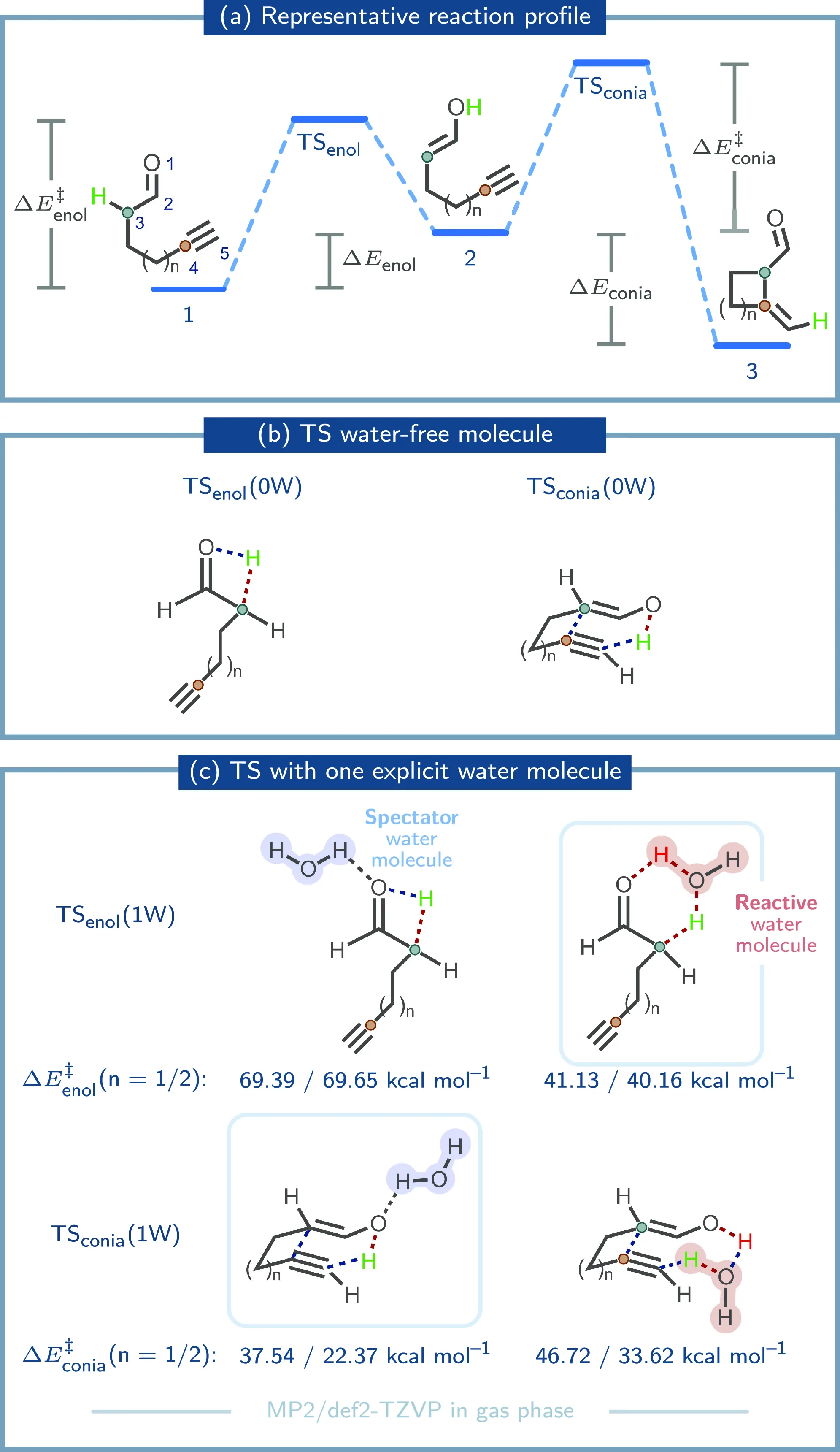
Thermal Conia–Ene cyclization: (a) representative energy profile, (b) water-free transition states, and (c) explicit water-assisted pathways highlighting spectator and reactive roles.

The computed energetic parameters for the two model substrates reveal clear trends in kinetic control and structural dependence for the water-free mechanism (entries 1–4 in Table 3; see Figure 2b for representative transition-state geometries). Across both substrates, the enolization barrier is consistently higher than that of the Conia–Ene step, thereby identifying enolization as the rate-determining step within the present mechanistic framework. This trend is reflected in Δ*E*_enol_^‡^, which is close to 70 kcal mol^–1^ for both substrates (1a and 1b), whereas Δ*E*_conia_^‡^ is substantially lower (25–41 kcal mol^–1^, depending on the substrate). Consequently, formation of the enol intermediate requires crossing a high barrier and incurs an energetic penalty due to the endothermic nature of the process; once formed, the subsequent cyclization proceeds with a lower barrier and is thermodynamically favorable, being strongly exothermic, particularly for hept-6-ynal (ca. −40 kcal mol^–1^; entries 3–4 in Table 3). In addition, the impact of implicit solvation remains minor relative to the gas phase, as SMD alters both activation barriers and reaction energies by only 1–2 kcal mol^–1^, indicating that the overall profile is largely insensitive to solvation. By contrast, intramolecular geometric preorganization associated with chain length exerts a pronounced effect on the cyclization barrier; specifically, Δ*E*_conia_^‡^ decreases by 15.6 kcal mol^–1^ from hex-5-ynal to hept-6-ynal, highlighting its role in facilitating C–C bond formation.

**Table 3:**
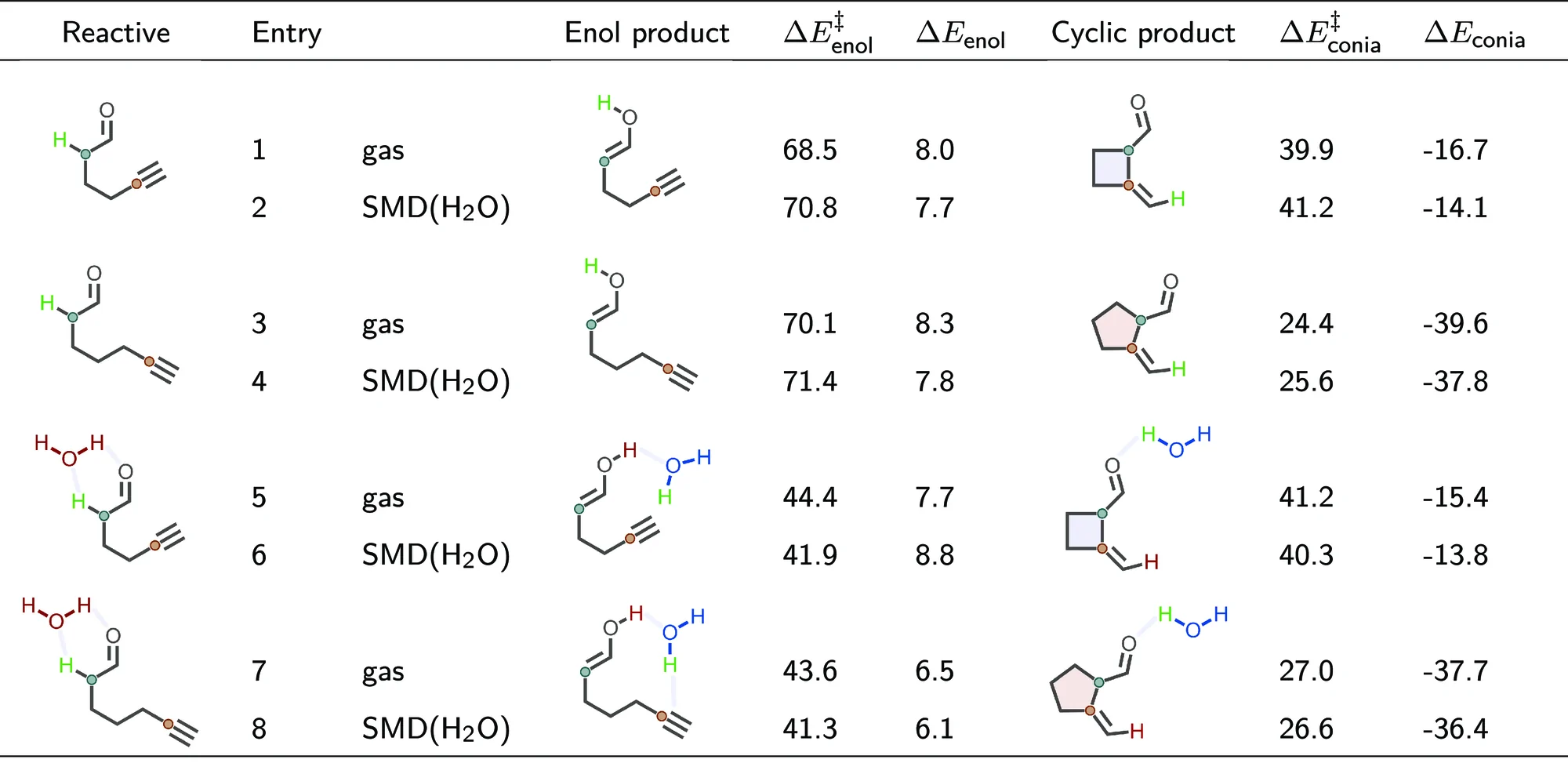
Barriers and Reaction Energies (in kcal mol^–1^) Computed at CCSD­(T)/aug-cc-pVTZ Level^a^

a Absolute energies are provided in Table S1 of the SI.

To assess the role of an explicit water molecule, its participation was examined at the MP2 ^89–94^ /def2–TZVP level (Figure 2c), where both reactive and spectator arrangements were considered for each elementary step. In the reactive arrangement, the water molecule is positioned between the proton donor and acceptor and mediates proton transfer as a transient shuttle, forming a hydrogen-bonded relay along the reaction coordinate. In contrast, in the spectator disposition the water molecule is placed to form a hydrogen-bond with the oxygen atom (without exhaustive placement sampling) and remains bound to the substrate without participating in bond reorganization, exerting primarily a stabilizing electrostatic effect. Comparison of the corresponding activation barriers (lowest Δ*E* ^‡^) indicates that water acts as a reactive participant in the enolization step, whereas such assistance is not favored along the Conia–Ene pathway. On this basis, the energy profiles discussed below adopt a mixed mechanistic scheme in which water participates reactively during enolization and remains spectator during cyclization (entries 5–8 in Table 3). Upon inclusion of explicit water, Δ*E*_enol_^‡^ decreases to 41–44 kcal mol^–1^ for both substrates (1a and 1b), corresponding to a reduction of 26–30 kcal mol^–1^ relative to the water-free case; this behavior is consistent with proton-transfer mediation by the water molecule.^95–101^ In contrast, Δ*E*_conia_^‡^ shows a markedly different response: it remains essentially unchanged for hex-5-ynal (40–41 kcal mol^–1^) and increases for hept-6-ynal to 27.0 kcal mol^–1^ in gas and 26.6 kcal mol^–1^ in SMD, indicating that explicit water does not stabilize the C–C bond-forming/allylic proton-transfer transition state. As a result, the balance between the activation barriers for enolization and cyclization is substantially altered, leading to nearly comparable barriers for hex-5-ynal (Δ*E*_enol_^‡^ vs Δ*E*_conia_^‡^: 44.4 vs 41.2 kcal mol^–1^ in gas; 41.9 vs 40.3 kcal mol^–1^ in SMD), whereas for hept-6-ynal enolization remains the kinetically controlling step, exceeding the Conia–Ene barrier by 16.6 kcal mol^–1^ in gas and 14.7 kcal mol^–1^ in SMD. Despite these kinetic changes, the underlying thermodynamic trends are preserved: enolization remains slightly endothermic (6–9 kcal mol^–1^), whereas the Conia–Ene step is strongly exothermic, with reaction energies of about −14 to −15 kcal mol^–1^ for hex-5-ynal and −36 to −38 kcal mol^–1^ for hept-6-ynal. Overall, these results define a compact benchmark manifold in which proton-transfer assistance, chain-length-dependent preorganization, and weak bulk-solvation effects can be evaluated simultaneously. Inclusion of implicit solvation exerts only a minor effect, whereas the presence of an explicit water molecule selectively lowers the barrier for tautomerization, leaving the cyclization step primarily governed by intramolecular geometric factors. This combination of chemically distinct yet computationally tractable profiles provides a stringent test for assessing the ability of DFAs to reproduce both kinetic and thermodynamic trends in thermally driven cyclizations.

### 3.2 Benchmarking DFT Approximations

The performance of different density functional approximations (DFAs) across Jacob’s ladder, from GGAs to double-hybrid functionals, was first assessed against the CCSD­(T) reference energies using the aug-cc-pVTZ basis set. The evaluation considers kinetic (Figure 3a), thermodynamic (Figure 3b), and combined metrics (Figure 3c) using mean absolute error (MAE) and associated standard deviations (SD). The values of the statistical parameters ME, MAE, MUE, and SD are listed in the Tables S2 and S3 of the SI. Both elementary steps of the Conia–Ene reaction were included, considering models with and without an explicit water molecule, under gas-phase (light bars) and implicit solvent (dark bars) conditions.

**Figure 3:**
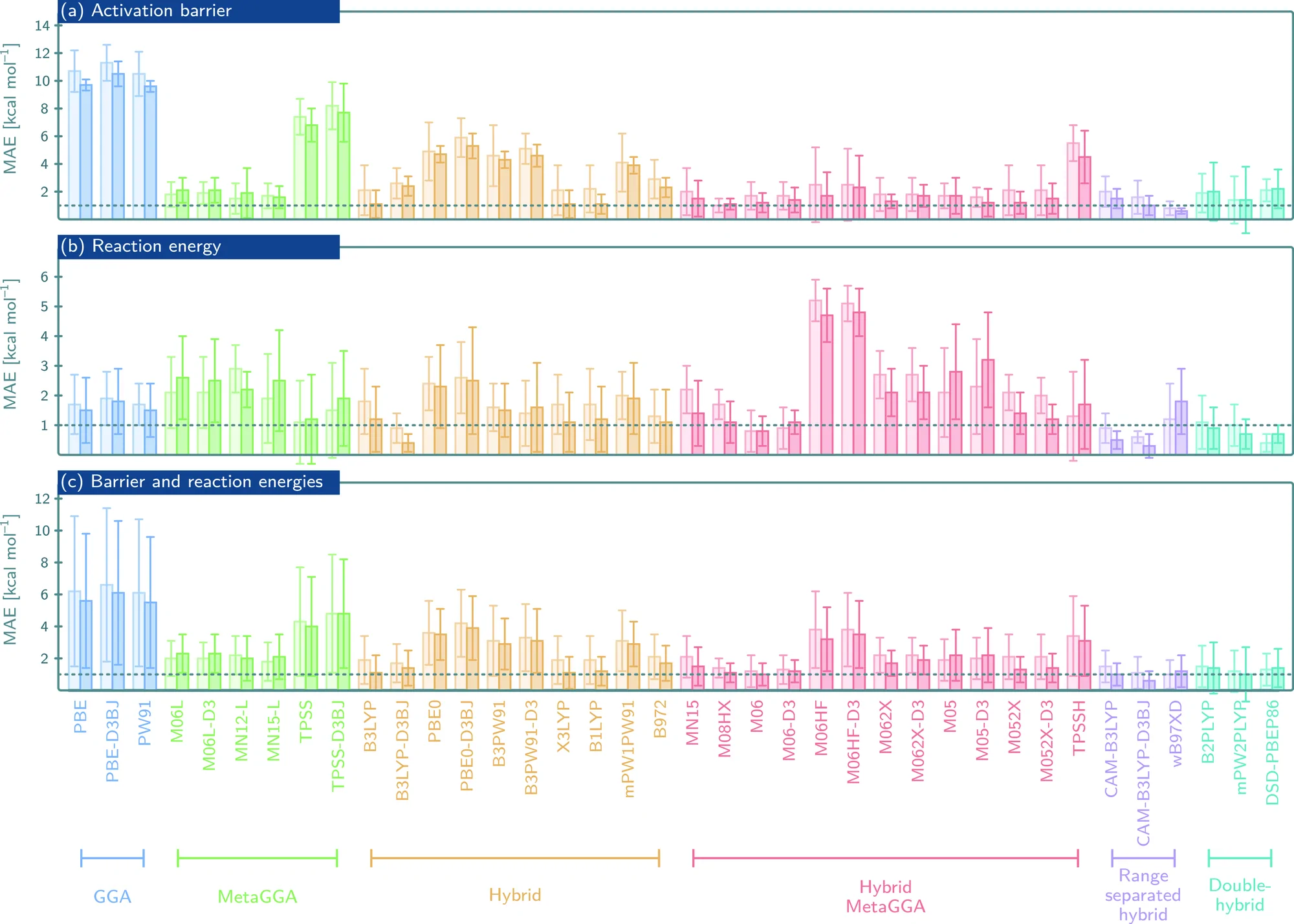
Mean absolute errors (MAEs) and standard deviations (SDs) for Conia–Ene reaction energies computed using DFAs at the XC/aug-cc-pVTZ//CCSD/6–31G­(d,p) level, relative to CCSD­(T)/aug-cc-pVTZ//CCSD/6–31G­(d,p) reference values. (a) Activation barriers, (b) reaction energies, and (c) combined analysis of barriers and reaction energies. For each functional, light and dark bars correspond to gas-phase and implicit solvent (SMD, water) calculations, respectively.

As shown in Figure 3, the differences between gas-phase and solvated MAEs are consistently lower or equal to 1.1 kcal mol^–1^, and both environments follow the same qualitative trends. This behavior is consistent with the weak influence of implicit solvation on the reaction energetics discussed in the mechanistic analysis, indicating that the relative performance of the DFAs is largely insensitive to the dielectric environment, introduced by the SMD approximation. Accordingly, the following analysis focuses on general trends that are valid for both conditions.

A comparison between the MAEs associated with activation barriers and reaction energies (Figure 3a and 3b, respectively) reveals that kinetic quantities are systematically more challenging to describe. Larger errors are observed for activation barriers, with a clear improvement along Jacob’s ladder. This trend is further supported by the error distributions in Figure 4a, which exhibits a significantly broader range of deviations, spanning from −13.80 to 7.50 kcal mol^–1^, compared to that found for reaction energies, ranging between −5.60 to 6.70 kcal mol^–1^. GGAs show the poorest performance, with narrow distributions but strongly negative medians, indicating a systematic underestimation of activation barriers. This behavior is consistent with the delocalization error, which leads to an artificial stabilization of transition states by excessive electron delocalization.^102^

**Figure 4:**
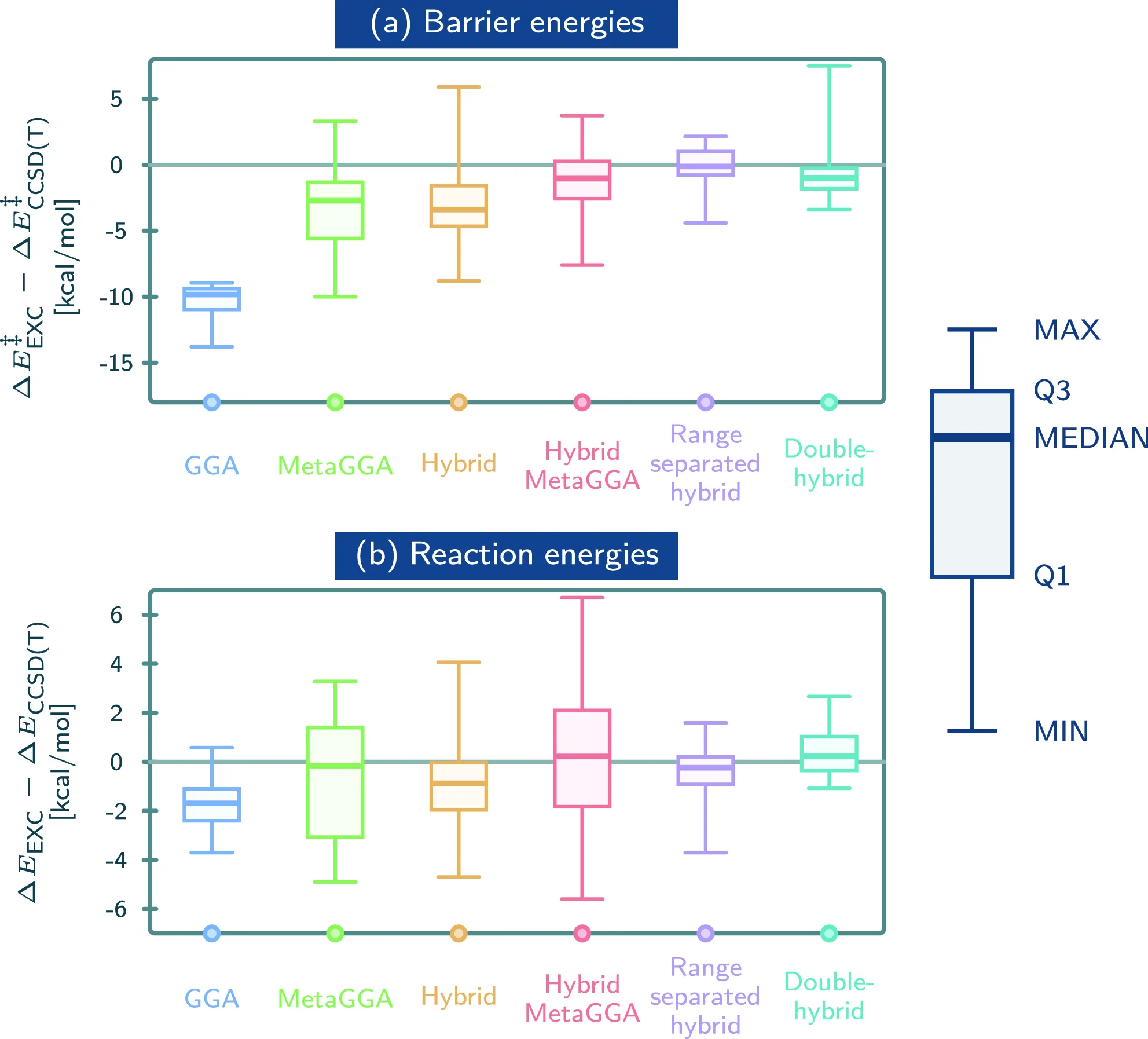
Error distribution of DFAs across different rungs of Jacob’s ladder for the Conia–Ene reaction. Boxplots represent the distribution of signed errors relative to CCSD­(T)/aug-cc-pVTZ//CCSD/6–31G­(d,p) reference values for (a) activation barriers and (b) reaction energies. Boxes denote the interquartile range, the central line indicates the median, and whiskers represent the full data range.

MetaGGAs, with the inclusion of kinetic energy density terms, reduce MAEs (Figure 3a) but exhibit broader distributions, predominantly negative values (Figure 4a), indicating persistent underestimation and increased variability. Hybrid functionals do not systematically improve this behavior, as several yield errors comparable to those of metaGGAs (Figure 3a), and their distributions remain largely shifted toward negative values (Figure 4a), highlighting that the used exchange-correlation approximation, rather than the mere inclusion of HF exchange, governs barrier accuracy. In contrast, hybrid metaGGAs, which combine the kinetic energy density and HF exchange with an appropriate parametrization, yield more balanced descriptions of activation barriers (Figure 3a). They show distributions that are closer to zero, although some dispersion remains (Figure 4a). Range-separated hybrids deliver the best performance, showing the lowest MAEs (Figure 3a) and minimal dispersion, with medians near zero (Figure 4a), reflecting reduced delocalization error and a more consistent description of transition states. Double-hybrid functionals exhibit a similarly accurate description of activation barriers. In particular, mPW2PLYP and DSD-PBEP86 yield MAEs comparable to those of the best range-separated hybrids (Figure 3a) while maintaining narrow error distributions centered near the reference values (Figure 4a). Their excellent performance is consistent with the explicit inclusion of perturbative second-order correlation.

In contrast, reaction energies (Figure 3b) are more accurately described across all DFAs, with smaller MAEs and no clear monotonic trend with the level of theory. This improved behavior is reflected in the narrower distributions shown in Figure 4b. GGAs and hybrid functionals exhibit relatively small dispersions but slightly negative medians, indicating a mild tendency to overestimate reaction energies. MetaGGAs and hybrid metaGGAs display broader distributions, with both over- and underestimations, reflecting a higher sensitivity to functional parametrization. Range-separated hybrids show tightly clustered distributions around zero, combining low dispersion with negligible bias, thereby providing the most reliable thermodynamic predictions. Double-hybrid functionals also reproduce reaction energies with excellent accuracy. Among them, mPW2PLYP and DSD-PBEP86 consistently exhibit low MAEs and narrow error distributions comparable to those of the best range-separated hybrids.

Finally, the global performance (Figure 3c) is largely dominated by the errors in activation barriers. GGAs can thus be ruled out due to their consistently large MAEs (ca. 5 kcal mol^–1^). MetaGGAs generally provide moderate improvements (MAEs < 3 kcal mol^–1^), making them suitable for exploratory studies. Hybrid functionals such as B3LYP, B3LYP-D3BJ, X3LYP, and B1LYP achieve MAEs on the order of 2 kcal mol^–1^, representing a reasonable compromise between accuracy and computational cost. Hybrid metaGGAs (e.g., M08HX, M06, M06-D3) approach chemical accuracy, while range-separated hybrids consistently deliver MAEs close to 1 kcal mol^–1^, emerging as the most reliable choice for describing both kinetic and thermodynamic aspects of the Conia–Ene reaction. Double-hybrid functionals, particularly mPW2PLYP and DSD-PBEP86, provide a comparable level of accuracy but at a substantially higher computational cost, making range-separated hybrids the more practical option for routine applications.

Notably, empirical dispersion corrections do not introduce significant differences for the systems studied here. However, their inclusion is expected to be relevant in more complex substrates bearing DED groups or confined environments where noncovalent interactions become significant.

### 3.3 Benchmarking Basis Sets

Since the prediction of kinetic and thermodynamic parameters depends not only on the choice of the DFA but also on the basis set, activation and reaction energies were computed in gas phase, for all DFAs using a variety of basis sets from commonly employed families in computational chemistry. The global MAEs, defined by combining the errors associated with both activation barriers and reaction energies, are summarized in the heat map shown in Figure 5, where higher color intensity indicates larger deviations. The remaining statistical parameters (ME, MAE, MUE and SD) are listed in Tables S4–S16 of the SI.

**Figure 5:**
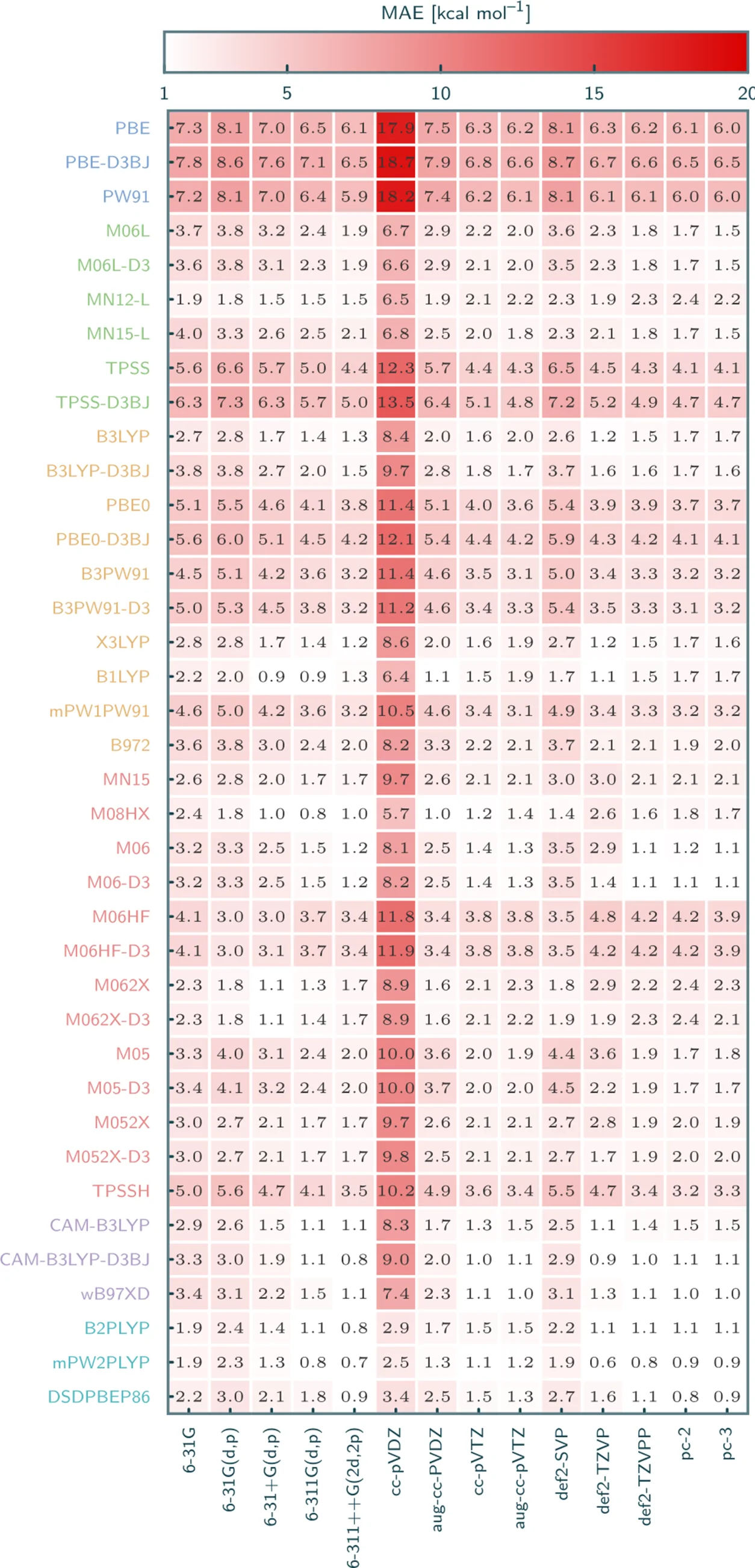
Barrier and reaction energies error distribution among different computational methods.

As a first observation, the general trends discussed above are preserved across all basis sets, indicating that the dominant source of error arises from the exchange–correlation functional. Nevertheless, the choice of basis set can significantly modulate the magnitude of these errors, either improving or deteriorating the overall performance. In this analysis, GGAs are excluded because they consistently yield the largest MAEs across all basis sets.

For the Pople basis sets (columns 1–5 in Figure 5), a systematic reduction of errors is observed upon inclusion of polarization and diffuse functions. In particular, from the 6–31+G­(d,p) level onward, most DFAs (especially those identified as top performers in the previous section) yield MAEs below 2 kcal mol^–1^. Functionals such as MN12-L, B3LYP, MN15, M062X, and M062X-D3 benefit notably from these basis sets, indicating that this family provides a reasonable balance between accuracy and computational cost while expanding the range of DFAs capable of reliably describing the Conia–Ene reaction.

For the Dunning basis sets (columns 6–9 in Figure 5), which were designed to systematically converge electron correlation effects, a strong dependence on basis size is observed. The double-ζ cc-pVDZ basis set is clearly inadequate, yielding MAEs consistently above 5 kcal mol^–1^. The inclusion of diffuse functions in the aug-cc-pVDZ basis set significantly improves results, approaching the performance of triple-ζ basis sets. In some cases, such as MN12-L, M062X, and M062X-D3, the MAEs decrease to values below 2 kcal mol^–1^. Moving to triple-ζ sets (cc-pVTZ and aug-cc-pVTZ), a general improvement with increasing basis size is observed, though notable exceptions exist. For certain functionals, including B3LYP and B3LYP-D3BJ, the nonaugmented cc-pVTZ basis yields slightly lower MAEs than its augmented counterpart, indicating that the inclusion of diffuse functions does not systematically enhance the prediction of kinetic and thermodynamic parameters within this family.

A similar trend is observed for the Ahlrichs basis sets (columns 10–12 in Figure 5), where the accuracy generally improves with increasing basis size. From the def2-TZVP level onward, several DFAs exhibit MAEs below 2 kcal mol^–1^. Functionals such as MN12-L, B3LYP, B3LYP-D3BJ, M062X-D3, and M052X-D3 show notable improvements within this family. Additionally, metaGGA functionals such as M06L and M06L-D3 achieve MAEs below 2 kcal mol^–1^ with the larger def2-TZVPP basis set.

Finally, for the Jensen basis sets (the last two columns in Figure 5), which are specifically optimized for DFT calculations, consistently improved performance is observed. Starting from the pc-2 basis, several DFAs yield MAEs comparable to, or even lower than, those obtained with the reference aug-cc-pVTZ basis. This results in a broader set of functionals capable of accurately reproducing both activation and reaction energies, including M06L, M06L-D3, MN15-L, B3LYP, B3LYP-D3BJ, M06, and M06-D3. A similar improvement is observed for the double-hybrid functionals, whose MAEs decrease markedly with larger basis sets. In particular, mPW2PLYP and DSD-PBEP86 achieve errors below 2 kcal mol^–1^ for several triple-ζ basis sets, confirming that their excellent performance is maintained across different basis-set families. Despite this favorable performance, the increased computational cost associated with Jensen basis sets may limit their applicability to larger systems.

## 4 Conclusions

This work establishes a CCSD­(T)-referenced benchmark framework for assessing density functional approximations in the thermal Conia–Ene cyclization and provides practical guidance for selecting DFT methods capable of reliably reproducing its activation and reaction energetics. By combining chain-length variation, explicit water assistance, and gas-phase/implicit-solvent conditions, the benchmark set captures chemically distinct situations involving proton-transfer mediation, transition-state preorganization, and weak environmental effects.

The reference energy profiles support a stepwise mechanism in which enolization is the intrinsic kinetic bottleneck of the water-free reaction, whereas the subsequent Conia–Ene cyclization is kinetically more accessible and strongly exothermic. Explicit water selectively lowers the enolization barrier through proton-transfer mediation, while chain length primarily modulates the cyclization step through improved geometric preorganization. In contrast, implicit solvation produces only minor energetic changes and does not alter the qualitative mechanistic picture.

Across the 38 density functional approximations examined, range-separated hybrids provide the most accurate and balanced description of both activation barriers and reaction energies relative to the CCSD­(T) reference data. In particular, ωB97XD delivers the best overall performance, while CAM-B3LYP-D3BJ also reaches near-chemical accuracy. Hybrid metaGGAs such as M06, M06-D3, and M08HX offer a competitive compromise between accuracy and computational cost. Comparable accuracy is also achieved by the double-hybrid functionals mPW2PLYP and DSD-PBEP86, although at a substantially higher computational cost. Basis-set analysis further shows that triple-ζ basis sets with polarization functions, and in several cases diffuse functions, are required for robust performance, whereas smaller double-ζ sets are generally inadequate.

Overall, the present results define a practical and transferable DFT benchmark for thermally activated cyclizations and establish a direct connection between mechanistic complexity and functional performance. The protocol identified here provides a sound basis for extending DFT studies to larger substrates and to more complex environments, including systems in which DED substituents promote dispersion-assisted stabilization, as well as confined environments where spatial restriction and host–guest interactions can further reshape the reaction energy landscape.

## Supplementary Material



## Notes

CCSD­(T)/aug-cc-pVTZ reference energies, complete statistical analysis of density functional approximations and basis sets, gas-phase and SMD single-point energies for all reaction profiles, and optimized Cartesian coordinates (XYZ) for all stationary points (PDF)

## References

[1] Conia J. M., Le Perchec P. (1975). The Thermal Cyclisation of Unsaturated Carbonyl Compounds. Synthesis.

[2] Aggarwal R. S., Mao W., Robertson C. M., Bower J. F. (2026). An Iridium-Catalyzed Conia-ene Reaction
for the Diastereo- and Enantioselective Synthesis of Complex Ring
Systems. Angew. Chem..

[3] Ling X., Wang J., Chen Y., Zhao P., Ni D., Wei Y., Nie S., He Y. (2025). Gold-Catalyzed Conia-Ene Cyclization/Aza-Michael
Addition Cascades: Access to Indolinylazepinone PNPs with Anticancer
Activity. J. Org. Chem..

[4] Muskan, Verma A. K. (2026). Conia-Ene Cyclization
of Pyrazolones with o -Alkynyl Aldehydes: A Strategic Shift from Classical
Condensation to Spiroannulation. Org. Lett..

[5] Badr S. M., Durmaz M., Menges N. (2026). A gold­(iii)-catalyzed
Conia-ene reaction
for the direct synthesis of substituted pyrroles from *β*-ketopropargyl amines. Org. Biomol. Chem..

[6] Hatakeyama S. (2009). Indium-catalyzed
Conia-ene reaction for alkaloid synthesis. Pure
Appl. Chem..

[7] Hatakeyama S. (2014). Total Synthesis
of Biologically Active Natural Products Based on Highly Selective
Synthetic Methodologies. Chem. Pharm. Bull..

[8] Du G., Wang G., Ma W., Yang Q., Bao W., Liang X., Zhu L., Lee C.-S. (2017). Syntheses of DiverseNatural
Products via Dual-Mode Lewis Acid Induced Cascade Cyclization Reactions. Synlett.

[9] Ruan Z., Zhang H., Liu F., Xie X. (2025). Applications of Conia-ene
reaction in total synthesis of natural products. Tetrahedron.

[10] Lin E.-Z., Xu Y., Ji K., Ye L.-W. (2021). Recent advances towards catalytic
asymmetric Conia-ene-type reactions. Chin. Chem.
Lett..

[11] Bloch R., Le Perchec P., Rouessac F., Conia J.-M. (1968). Thermolyse et photolyse
de cétones non saturées—IX. Tetrahedron.

[12] Moinet G., Brocard J., Conia J. M. (1972). Sur la thermocyclisation de cetones
d-ethyleniques en cyclohexanones. Tetrahedron
Lett..

[13] Dénès F., Pérez-Luna A., Chemla F. (2010). Addition of Metal Enolate Derivatives
to Unactivated Carbon-Carbon Multiple Bonds. Chem. Rev..

[14] Kitagawa O., Fujiwara H., Suzuki T., Taguchi T., Shiro M. (2000). Intramolecular
Carbostannation Reaction of Active Methine Compounds with an Unactivated
C-C p-Bond Mediated by SnCl_4_ -Et_3_N. J. Org. Chem..

[15] Cerveri A., Vettori M., Serafino A., Maestri G. (2023). Base-promoted Conia-ene
cyclization of propargyl amides. Org. Biomol.
Chem..

[16] Kennedy-Smith J. J., Staben S. T., Dean Toste F. (2004). Gold­(I)-Catalyzed Conia-Ene Reaction
of *β*-Ketoesters with Alkynes. J. Am. Chem. Soc..

[17] Brazeau J.-F., Zhang S., Colomer I., Corkey B. K., Dean
Toste F. (2012). Enantioselective Cyclizations of Silyloxyenynes Catalyzed by Cationic
Metal Phosphine Complexes. J. Am. Chem. Soc..

[18] Fang W., Presset M., Guérinot A., Bour C., Bezzenine-Lafollée S., Gandon V. (2014). Silver-Free Two-Component Approach in Gold Catalysis:
Activation of [LAuCl] Complexes with Derivatives of Copper, Zinc,
Indium, Bismuth, and Other Lewis Acids. Chem.
- Eur. J..

[19] Itoh Y., Tsuji H., Yamagata K.-I., Endo K., Tanaka I., Nakamura M., Nakamura E. (2008). Efficient Formation of Ring Structures
Utilizing Multisite Activation by Indium Catalysis. J. Am. Chem. Soc..

[20] Lim H. N., Dong G. (2015). Catalytic Intramolecular Ketone Alkylation with Olefins by Dual Activation. Angew. Chem..

[21] Sahu A. K., Unnava R., Shit S., Saikia A. K. (2020). In­(OTf)_3_ -Catalyzed One-Pot Tandem Mannich and Conia-Ene Cyclization Reaction
of N -Propargyl Amido Alcohols with 1,3-Dicarbonyl Compounds: An Approach
To Construct Tetrahydro-1 H -pyrrolo­[2,1- a ]­isoindolone-1,1-dicarboxylate
and Its Application. J. Org. Chem..

[22] Lonsdale R., Harvey J. N., Mulholland A. J. (2012). Effects
of Dispersion in Density
Functional Based Quantum Mechanical/Molecular Mechanical Calculations
on Cytochrome P450 Catalyzed Reactions. J. Chem.
Theory Comput..

[23] Lyngvi E., Sanhueza I. A., Schoenebeck F. (2015). Dispersion
Makes the Difference:
Bisligated Transition States Found for the Oxidative Addition of Pd­(P
t Bu_3_)_2_ to Ar-OSO_2_ R and Dispersion-Controlled
Chemoselectivity in Reactions with Pd­[P­(i Pr)­(t Bu_2_)]_2_. Organometallics.

[24] Solel E., Ruth M., Schreiner P. R. (2021). London
dispersion helps refine steric
a-values: Dispersion energy donor scales. J.
Am. Chem. Soc..

[25] Brown C. J., Toste F. D., Bergman R. G., Raymond K. N. (2015). Supramolecular Catalysis
in Metal-Ligand Cluster Hosts. Chem. Rev..

[26] Liu L., Leutzsch M., Zheng Y., Alachraf M. W., Thiel W., List B. (2015). Confined Acid-Catalyzed
Asymmetric Carbonyl-Ene Cyclization. J. Am.
Chem. Soc..

[27] Mu X., Ji X., Chen X., Wu H., Rui J., Hong X., Worth M. M., Reitz A. D., Goldberg L. T. M., Garcia-Borràs M., Michel S. L. J., Yang Y., Huang X. (2025). Unlocking Lewis acid
catalysis in non-haem enzymes for an abiotic ene reaction. Nat. Catal..

[28] Matanović I., Došlić N., Mihalić Z. (2004). Exploring
the potential energy surface
for proton transfer in acetylacetone. Chem.
Phys..

[29] Mangiatordi G. F., Brémond E., Adamo C. (2012). DFT and Proton Transfer Reactions:
A Benchmark Study on Structure and Kinetics. J. Chem. Theory Comput..

[30] Samson C. C. M., Klopper W. (2002). Ab initio calculation of proton barrier and binding
energy of the (H2O)­OH- complex. J. Mol. Struct.
Theochem.

[31] Bursch M., Mewes J.-M., Hansen A., Grimme S. (2022). Best-Practice DFT Protocols
for Basic Molecular Computational Chemistry**. Angew. Chem., Int. Ed..

[32] Karton A., De Oliveira M. T. (2025). Good Practices in Database Generation for Benchmarking
Density Functional Theory. WIREs Comput. Mol.
Sci..

[33] Boese A. D., Martin J. M. L., Handy N. C. (2003). The role of the basis set: Assessing
density functional theory. J. Chem. Phys..

[34] Hill J. G. (2013). Gaussian
basis sets for molecular applications. Int.
J. Quantum Chem..

[35] Marenich A. V., Cramer C. J., Truhlar D. G. (2009). Universal
Solvation Model Based on
Solute Electron Density and on a Continuum Model of the Solvent Defined
by the Bulk Dielectric Constant and Atomic Surface Tensions. J. Phys. Chem. B.

[36] Advances in Chemical Physics, 1st ed.; LeFebvre, R. ; Moser, C. , Eds.; Wiley, 1969; Vol. 14.

[37] Bartlett R. J., Purvis G. D. (1978). Many-body perturbation theory, coupled-pair many-electron
theory, and the importance of quadruple excitations for the correlation
problem. Int. J. Quantum Chem..

[38] Purvis G. D., Bartlett R. J. (1982). A full coupled-cluster
singles and doubles model: The
inclusion of disconnected triples. J. Chem.
Phys..

[39] Scuseria G.
E., Janssen C. L., Schaefer H. F. (1988). An efficient reformulation of the
closed-shell coupled cluster single and double excitation (CCSD) equations. J. Chem. Phys..

[40] Scuseria G. E., Schaefer H. F. (1989). Is coupled cluster singles and doubles (CCSD) more
computationally intensive than quadratic configuration interaction
(QCISD)?. J. Chem. Phys..

[41] Ditchfield R., Hehre W. J., Pople J. A. (1971). Self-consistent
molecular-orbital
methods. ix. an extended gaussian-type basis for molecular-orbital
studies of organic molecules. J. Chem. Phys..

[42] Hehre W. J., Ditchfield R., Pople J. A. (1972). Self-consistent molecular orbital
methods. xii. further extensions of gaussian-type basis sets for use
in molecular orbital studies of organic molecules. J. Chem. Phys..

[43] Hariharan P. C., Pople J. A. (1973). The influence of polarization functions on molecular
orbital hydrogenation energies. Theor. Chim.
Acta.

[44] Pople J. A., Head-Gordon M., Raghavachari K. (1987). Quadratic configuration interaction.
A general technique for determining electron correlation energies. J. Chem. Phys..

[45] Lang J., Przybytek M., Lesiuk M. (2025). Estimating the Complete Basis Set
Extrapolation Error through Random Walks. J.
Phys. Chem. Lett..

[46] Mehta N., Martin J. M. L. (2022). Explicitly Correlated
Double-Hybrid DFT: A Comprehensive
Analysis of the Basis Set Convergence on the GMTKN55 Database. J. Chem. Theory Comput..

[47] Karton A. (2020). Effective
basis set extrapolations for CCSDT, CCSDT­(Q), and CCSDTQ correlation
energies. J. Chem. Phys.,.

[48] Perdew J. P., Burke K., Ernzerhof M. (1996). Generalized Gradient Approximation
Made Simple. Phys. Rev. Lett..

[49] Perdew J. P., Burke K., Ernzerhof M. (1997). Generalized
Gradient Approximation
Made Simple [Phys. Rev. Lett. 77, 3865 (1996)]. Phys. Rev. Lett..

[50] Grimme S., Ehrlich S., Goerigk L. (2011). Effect of the damping
function in
dispersion corrected density functional theory. J. Comput. Chem..

[51] Perdew J. P., Chevary J. A., Vosko S. H., Jackson K. A., Pederson M. R., Singh D. J., Fiolhais C. (1992). Atoms, molecules, solids, and surfaces:
Applications of the generalized gradient approximation for exchange
and correlation. Phys. Rev. B.

[52] Perdew J. P., Chevary J. A., Vosko S. H., Jackson K. A., Pederson M. R., Singh D. J., Fiolhais C. (1993). Erratum: Atoms, molecules,
solids,
and surfaces: Applications of the generalized gradient approximation
for exchange and correlation. Phys. Rev. B.

[53] Zhao Y., Truhlar D. G. (2006). A new local density
functional for main-group thermochemistry,
transition metal bonding, thermochemical kinetics, and noncovalent
interactions. J. Chem. Phys..

[54] Grimme S., Antony J., Ehrlich S., Krieg H. (2010). A consistent and accurate
ab initio parametrization of density functional dispersion correction
(DFT-D) for the 94 elements H-Pu. J. Chem. Phys..

[55] Peverati R., Truhlar D. G. (2012). An improved and broadly accurate local approximation
to the exchange-correlation density functional: The MN12-L functional
for electronic structure calculations in chemistry and physics. Phys. Chem. Chem. Phys..

[56] Yu H. S., He X., Truhlar D. G. (2016). MN15-L: A New Local Exchange-Correlation Functional
for Kohn-Sham Density Functional Theory with Broad Accuracy for Atoms,
Molecules, and Solids. J. Chem. Theory Comput..

[57] Tao J., Perdew J. P., Staroverov V. N., Scuseria G. E. (2003). Climbing the Density
Functional Ladder: Nonempirical Meta-Generalized Gradient Approximation
Designed for Molecules and Solids. Phys. Rev.
Lett..

[58] Lee C., Yang W., Parr R. G. (1988). Development
of the Colle-Salvetti
correlation-energy formula into a functional of the electron density. Phys. Rev. B.

[59] Becke A. D. (1993). Density-functional
thermochemistry. III. The role of exact exchange. J. Chem. Phys..

[60] Stephens P. J., Devlin F. J., Chabalowski C. F., Frisch M. J. (1994). Ab Initio Calculation
of Vibrational Absorption and Circular Dichroism Spectra Using Density
Functional Force Fields. J. Phys. Chem. A.

[61] Adamo C., Barone V. (1999). Toward reliable density
functional methods without
adjustable parameters: The PBE0 model. J. Chem.
Phys..

[62] Xu X., Goddard W. A. (2004). The X3LYP extended
density functional for accurate
descriptions of nonbond interactions, spin states, and thermochemical
properties. Proc. Natl. Acad. Sci. U.S.A..

[63] Adamo C., Barone V. (1997). Toward reliable adiabatic
connection models free from
adjustable parameters. Chem. Phys. Lett..

[64] Adamo C., Barone V. (1998). Exchange functionals
with improved long-range behavior
and adiabatic connection methods without adjustable parameters: The
mPW and mPW1PW models. J. Chem. Phys..

[65] Wilson P. J., Bradley T. J., Tozer D. J. (2001). Hybrid exchange-correlation functional
determined from thermochemical data and ab initio potentials. J. Chem. Phys..

[66] Yu H. S., He X., Li S. L., Truhlar D. G. (2016). MN15: A Kohn-Sham global-hybrid exchange-correlation
density functional with broad accuracy for multi-reference and single-reference
systems and noncovalent interactions. Chem.
Sci..

[67] Zhao Y., Truhlar D. G. (2008). Exploring the Limit
of Accuracy of the Global Hybrid
Meta Density Functional for Main-Group Thermochemistry, Kinetics,
and Noncovalent Interactions. J. Chem. Theory
Comput..

[68] Zhao Y., Truhlar D. G. (2008). The M06 suite of
density functionals for main group
thermochemistry, thermochemical kinetics, noncovalent interactions,
excited states, and transition elements: two new functionals and systematic
testing of four M06-class functionals and 12 other functionals. Theor. Chem. Acc..

[69] Zhao Y., Truhlar D. G. (2006). Density Functional for Spectroscopy:
No Long-Range
Self-Interaction Error, Good Performance for Rydberg and Charge-Transfer
States, and Better Performance on Average than B3LYP for Ground States. J. Phys. Chem. A.

[70] Zhao Y., Schultz N. E., Truhlar D. G. (2005). Exchange-correlation functional with
broad accuracy for metallic and nonmetallic compounds, kinetics, and
noncovalent interactions. J. Chem. Phys..

[71] Zhao Y., Schultz N. E., Truhlar D. G. (2006). Design of Density Functionals by
Combining the Method of Constraint Satisfaction with Parametrization
for Thermochemistry, Thermochemical Kinetics, and Noncovalent Interactions. J. Chem. Theory Comput..

[72] Staroverov V. N., Scuseria G. E., Tao J., Perdew J. P. (2003). Comparative assessment
of a new nonempirical density functional: Molecules and hydrogen-bonded
complexes. J. Chem. Phys..

[73] Staroverov V. N., Scuseria G. E., Tao J., Perdew J. P. (2004). Erratum: “Comparative
assessment of a new nonempirical density functional: Molecules and
hydrogen-bonded complexes” [J. Chem. Phys. 119, 12129 (2003)]. J. Chem. Phys..

[74] Yanai T., Tew D. P., Handy N. C. (2004). A new hybrid
exchange-correlation
functional using the Coulomb-attenuating method (CAM-B3LYP). Chem. Phys. Lett..

[75] Chai J.-D., Head-Gordon M. (2008). Long-range corrected hybrid density
functionals with
damped atom-atom dispersion corrections. Phys.
Chem. Chem. Phys..

[76] Grimme S. (2006). Semiempirical
GGA-type density functional constructed with a long-range dispersion
correction. J. Comput. Chem..

[77] Grimme S. (2006). Semiempirical
hybrid density functional with perturbative second-order correlation. J. Chem. Phys..

[78] Schwabe T., Grimme S. (2006). Towards chemical accuracy
for the thermodynamics of
large molecules: new hybrid density functionals including non-local
correlation effects. Phys. Chem. Chem. Phys..

[79] Kozuch S., Martin J. M. L. (2011). DSD-PBEP86: in
search of the best double-hybrid DFT
with spin-component scaled MP2 and dispersion corrections. Phys. Chem. Chem. Phys..

[80] Dunning T. H. (1989). Gaussian
basis sets for use in correlated molecular calculations. i. the atoms
boron through neon and hydrogen. J. Chem. Phys..

[81] Kendall R. A., Dunning T. H., Harrison R. J. (1992). Electron
affinities of the first-row
atoms revisited. systematic basis sets and wave functions. J. Chem. Phys..

[82] Clark T., Chandrasekhar J., Spitznagel G. W., Schleyer P. V. R. (1983). Efficient diffuse
function-augmented basis sets for anion calculations. iii. the 3–21+g
basis set for first-row elements, li-f. J. Comput.
Chem..

[83] Krishnan R., Binkley J. S., Seeger R., Pople J. A. (1980). Self-consistent
molecular orbital methods. xx. a basis set for correlated wave functions. J. Chem. Phys..

[84] Weigend F., Ahlrichs R. (2005). Balanced basis sets of split valence, triple zeta valence
and quadruple zeta valence quality for h to rn: Design and assessment
of accuracy. Phys. Chem. Chem. Phys..

[85] Jensen F. (2001). Polarization
consistent basis sets: Principles. J. Chem.
Phys..

[86] Jensen F. (2002). Polarization
consistent basis sets. ii. estimating the kohn-sham basis set limit. J. Chem. Phys..

[87] Frisch, M. J. ; Trucks, G. W. ; Schlegel, H. B. ; Scuseria, G. E. ; Robb, M. A. ; Cheeseman, J. R. ; Scalmani, G. ; Barone, V. ; Petersson, G. A. ; Nakatsuji, H. ; Li, X. ; Caricato, M. ; Marenich, A. V. ; Bloino, J. ; Janesko, B. G. ; Gomperts, R. ; Mennucci, B. ; Hratchian, H. P. ; Ortiz, J. V. ; Izmaylov, A. F. ; Sonnenberg, J. L. ; Williams-Young, D. ; Ding, F. ; Lipparini, F. ; Egidi, F. ; Goings, J. ; Peng, B. ; Petrone, A. ; Henderson, T. ; Ranasinghe, D. ; Zakrzewski, V. G. ; Gao, J. ; Rega, N. ; Zheng, G. ; Liang, W. ; Hada, M. ; Ehara, M. ; Toyota, K. ; Fukuda, R. ; Hasegawa, J. ; Ishida, M. ; Nakajima, T. ; Honda, Y. ; Kitao, O. ; Nakai, H. ; Vreven, T. ; Throssell, K. ; Montgomery, J. A. ; Peralta, J. J. E. ; Ogliaro, F. ; Bearpark, M. J. ; Heyd, J. J. ; Brothers, E. N. ; Kudin, K. N. ; Staroverov, V. N. ; Keith, T. A. ; Kobayashi, R. ; Normand, J. ; Raghavachari, K. ; Rendell, A. P. ; Burant, J. C. ; Iyengar, S. S. ; Tomasi, J. ; Cossi, M. ; Millam, J. M. ; Klene, M. ; Adamo, C. ; Cammi, R. ; Ochterski, J. W. ; Martin, R. L. ; Morokuma, K. ; Farkas, O. ; Foresman, J. B. ; Fox, D. J. Gaussian Inc. 16 Revision C.01; Gaussian Inc.: Wallingford CT, 2016.

[88] Alabugin I.
V., Gilmore K. (2013). Finding the
right path: Baldwin “Rules for Ring
Closure” and stereoelectronic control of cyclizations. Chem. Commun..

[89] Møller C., Plesset M. S. (1934). Note on an Approximation Treatment for Many-Electron
Systems. Phys. Rev..

[90] Head-Gordon M., Pople J. A., Frisch M. J. (1988). MP2 energy
evaluation by direct methods. Chem. Phys. Lett..

[91] Sæbø S., Almlöf J. (1989). Avoiding the
integral storage bottleneck in LCAO calculations
of electron correlation. Chem. Phys. Lett..

[92] Frisch M. J., Head-Gordon M., Pople J. A. (1990). A direct MP2 gradient method. Chem. Phys. Lett..

[93] Frisch M. J., Head-Gordon M., Pople J. A. (1990). Semi-direct algorithms
for the MP2
energy and gradient. Chem. Phys. Lett..

[94] Head-Gordon M., Head-Gordon T. (1994). Analytic MP2
frequencies without fifth-order storage.
Theory and application to bifurcated hydrogen bonds in the water hexamer. Chem. Phys. Lett..

[95] Cucinotta C. S., Ruini A., Catellani A., Stirling A. (2006). Ab Initio Molecular
Dynamics Study of the Keto-Enol Tautomerism of Acetone in Solution. ChemPhysChem.

[96] González-Rivas N., Cedillo A. (2012). Solvent effects on the energetic parameters and chemical
reactivity in the keto-enol tautomeric equilibrium of substituted
carbonyl compounds. Comput. Theor. Chem..

[97] D’Cunha C., Morozov A. N., Chatfield D. C. (2013). Theoretical
Study of HOCl-Catalyzed
Keto-Enol Tautomerization of *β*-Cyclopentanedione
in an Explicit Water Environment. J. Phys. Chem.
A.

[98] Oziminski W. P. (2016). The kinetics
of water-assisted tautomeric 1,2-proton transfer in azoles: a computational
approach. Struct. Chem..

[99] Romero-Montalvo E., Guevara-Vela J. M., Narváez W. E.
V., Costales A., Pendás A. ´ M., Hernández-Rodríguez M., Rocha-Rinza T. (2017). The bifunctional catalytic role of water clusters in
the formation of acid rain. Chem. Commun..

[100] Sauza-de la Vega A., Salazar-Lozas H., Narváez W. E. V., Hernández-Rodríguez M., Rocha-Rinza T. (2021). Water clusters
as bifunctional catalysts in organic chemistry: the hydrolysis of
oxirane and its methyl derivatives. Org. Biomol.
Chem..

[101] Würmel J., Simmie J. M. (2024). Kinetics of direct
and water-mediated
tautomerization reactions of five-membered cyclic amides or lactams. Int. J. Chem. Kinet..

[102] Bryenton K. R., Adeleke A. A., Dale S. G., Johnson E. R. (2023). Delocalization
error: The greatest outstanding challenge in density-functional theory. WIREs Comput. Mol. Sci..

