# Supplementary material for: Benchmarking DFT for Chain-Length and Water Effects in Thermal Conia–Ene Cyclization
Source: ACS Omega. 2026 Aug 18;11(34):51623–34. doi: 10.1021/acsomega.6c05834 (PMC13625091; doi:10.1021/acsomega.6c05834)
Supplement: Supplementary file 1 [file ao6c05834_si_001.pdf]

Benchmarking DFT for Chain-Length and Water Effects in Thermal Conia–Ene Cyclization

Supplementary Information

Jorge Gutiérrez-Flores[1], Eduardo H. Huerta[2,*], Javier Serrano Medina[1], Jorge Garza[1], Marcos Hernández-Rodríguez[2,*], Rubicelia Vargas[1,*]

[1]Departamento de Química, División de Ciencias Básicas e Ingeniería, Universidad Autónoma Metropolitana Iztapalapa, San Rafael Atlixco 186, Col. Vicentina, C.P. 09340 Iztapalapa, CDMX, México; rvargas@izt.uam.mx

[2]Universidad Nacional Autónoma de México, Instituto de Química, Circuito Exterior, Ciudad Universitaria, Alc. Coyoacán, Ciudad de México, 04510, México; e.hh@quimica.unam.mx, marcoshr@unam.mx

Contents

1 Reference energies at CCSD(T) level **3**

2 Statistical Analysis of Benchmark Performance **4**

2.1 Density functional approximations (DFAs) 4

2.1.1 Gas phase 4

2.1.2 SMD model 5

2.2 Basis sets 6

3 Gas phase energies **22**

4 SMD energies **27**

5 Basis set effect **32**

5.1 6-31G 32

5.2 6-31G(d,p) 37

5.3 6-31+G(d,p) 41

5.4 6-311G(d,p) 46

5.5 6-311++G(2d,2p) 50

5.6 cc-pVDZ 55

5.7 aug-cc-pVDZ 60

5.8 cc-pVTZ 64
5.9 def2-SVP 69
5.10 def2-TZVP 74
5.11 def2-TZVPP 78
5.12 pc-2 83
5.13 pc-3 88

6 XYZ coordinates **93**

6.1 Hex-5-ynal gas phase 93
6.2 Hex-5-ynal implicit solvent 96
6.3 Hept-6-ynal gas phase 98
6.4 Hept-6-ynal implicit solvent 102
6.5 Hex-5-ynal + H_2O gas phase 105
6.6 Hex-5-ynal + H_2O implicit solvent 108
6.7 Hept-6-ynal + H_2O gas phase 112
6.8 Hept-6-ynal + H_2O implicit solvent 115

1 Reference energies at CCSD(T) level

Absolute electronic energies at the CCSD(T)/aug-cc-pVTZ level on CCSD/6-31G(d,p) optimized geometries, in both gas-phase and implicit solvent conditions, are compiled in Table S1. The corresponding optimized XYZ coordinates for all intermediates are given in Section **??**.

Table S1: CCSD(T)/aug-cc-pVTZ single-point energies on CCSD/6-31G(d,p) optimized geometries.

Entry	Model	1	TS_{enol}	2	TS_{conia}	3
**Hex-5-ynal (1b)**						
1	gas	-308.0874483	-307.9784291	-308.0747491	-308.0112212	-308.1013997
2	SMD(H2O)	-308.0957246	-307.9829230	-308.0834588	-308.0177484	-308.1058897
**Hept-6-ynal (2b)**						
3	gas	-347.3289427	-347.2172255	-347.3157755	-347.2769468	-347.3788838
4	SMD(H2O)	-347.3358445	-347.2221241	-347.3234855	-347.2826867	-347.3836673
**Hex-5-ynal (1b) + H_2O**						
5	gas	-384.4419462	-384.3711400	-384.4296563	-384.3639606	-384.4542281
6	SMD(H2O)	-384.4567366	-384.3900176	-384.4426641	-384.3783734	-384.4646662
**Hept-6-ynal (1b) + H_2O**						
7	gas	-423.6824188	-423.6129791	-423.6720732	-423.6289914	-423.7321977
8	SMD(H2O)	-423.6954887	-423.6296614	-423.6857359	-423.6432820	-423.7437923

2 Statistical Analysis of Benchmark Performance

2.1 Density functional approximations (DFAs)

2.1.1 Gas phase

Table S2: Mean error (ME), mean absolute error (MAE), maximum unsigned error (MUE), and standard deviation (SD) of reaction barriers and energies (kcal mol^{-1}) for the thermal Conia–Ene reaction, computed using various density functional approximations at the XC/aug-cc-pVTZ//CCSD/6-31G(d,p) level, relative to CCSD(T)/aug-cc-pVTZ//CCSD/6-31G(d,p) reference values.

	$\Delta E^\dagger$				ΔE				Total			
XC	ME	MAE	MUE	SD	ME	MAE	MUE	SD	ME	MAE	MUE	SD
CCSD(T)	0.0	0.0	0.0	0.0	0.0	0.0	0.0	0.0	0.0	0.0	0.0	0.0
PBE	-10.7	10.7	13.8	1.5	-1.6	1.7	3.0	1.0	-6.1	6.2	13.8	4.7
PBE-D3BJ	-11.3	11.3	13.1	1.3	-1.9	1.9	3.3	0.9	-6.6	6.6	13.1	4.8
PW91	-10.5	10.5	13.8	1.6	-1.4	1.7	2.4	0.7	-6.0	6.1	13.8	4.6
M06L	-1.8	1.8	3.3	0.9	0.3	2.1	3.7	1.2	-0.8	2.0	3.7	1.1
M06L-D3	-1.9	1.9	3.2	0.8	0.3	2.1	3.7	1.2	-0.8	2.0	3.7	1.0
MN12-L	-1.3	1.5	3.8	1.1	-0.4	2.9	4.3	0.8	-0.9	2.2	4.3	1.2
MN15-L	-0.8	1.7	3.3	0.9	-1.6	1.9	4.2	1.5	-1.2	1.8	4.2	1.2
TPSS	-7.4	7.4	9.5	1.3	-1.0	1.1	3.7	1.4	-4.2	4.3	9.5	3.4
TPSS-D3BJ	-8.2	8.2	9.9	1.7	-1.3	1.5	4.2	1.6	-4.7	4.8	9.9	3.7
B3LYP	-0.9	2.1	5.4	1.8	0.6	1.8	4.0	1.1	-0.1	1.9	5.4	1.5
B3LYP-D3BJ	-2.6	2.6	4.4	1.1	0.1	0.9	1.7	0.5	-1.2	1.7	4.4	1.2
PBE0	-4.9	4.9	8.5	2.1	-2.4	2.4	3.9	0.9	-3.6	3.6	8.5	2.0
PBE0-D3BJ	-5.9	5.9	7.8	1.4	-2.6	2.6	4.3	1.2	-4.2	4.2	7.8	2.1
B3PW91	-4.6	4.6	8.8	2.2	-1.6	1.6	2.7	0.8	-3.1	3.1	8.8	2.2
B3PW91-D3	-5.1	5.1	6.7	1.1	-1.4	1.4	3.2	1.1	-3.3	3.3	6.7	2.1
X3LYP	-0.8	2.1	5.2	1.8	0.5	1.7	3.6	1.0	-0.2	1.9	5.2	1.5
B1LYP	0.9	2.2	5.9	1.7	0.8	1.7	4.1	1.2	0.8	1.9	5.9	1.5
mPW1PW91	-4.1	4.1	8.0	2.1	-2.0	2.0	3.4	0.8	-3.1	3.1	8.0	1.9
B972	-2.9	2.9	6.1	1.4	-1.3	1.3	2.9	0.9	-2.1	2.1	6.1	1.4
MN15	-1.9	2.0	4.2	1.7	-0.6	2.2	3.3	0.8	-1.3	2.1	4.2	1.3
M08HX	0.8	1.0	2.1	0.5	0.2	1.7	2.4	0.5	0.5	1.4	2.4	0.6
M06	-1.6	1.7	3.1	1.0	0.8	0.8	2.1	0.7	-0.4	1.2	3.1	1.0
M06-D3	-1.6	1.7	3.2	1.0	0.9	0.9	2.2	0.7	-0.4	1.3	3.2	0.9
M06HF	-2.0	2.5	7.2	2.7	0.1	5.2	6.7	0.7	-1.0	3.8	7.2	2.4
M06HF-D3	-2.0	2.5	7.2	2.6	0.1	5.1	6.4	0.6	-0.9	3.8	7.2	2.3

Continued on next page

Table S2 (continued)

	$\Delta E^{\dagger}$				ΔE				Total			
XC	ME	MAE	MUE	SD	ME	MAE	MUE	SD	ME	MAE	MUE	SD
M062X	-0.7	1.8	4.2	1.2	0.7	2.7	4.1	0.8	0.0	2.2	4.2	1.1
M062X-D3	-0.8	1.8	4.2	1.2	0.7	2.7	4.2	0.9	0.0	2.2	4.2	1.1
M05	-0.7	1.7	2.8	0.9	-0.1	2.1	4.8	1.5	-0.4	1.9	4.8	1.3
M05-D3	-0.7	1.6	2.7	0.7	0.1	2.3	4.7	1.6	-0.3	2.0	4.7	1.3
M052X	-2.1	2.1	5.3	1.8	-0.4	2.1	2.6	0.6	-1.2	2.1	5.3	1.4
M052X-D3	-2.1	2.1	5.3	1.8	-0.3	2.0	2.6	0.6	-1.2	2.1	5.3	1.4
TPSSH	-5.5	5.5	7.6	1.3	-1.3	1.3	4.0	1.5	-3.4	3.4	7.6	2.5
CAM-B3LYP	-0.1	2.0	4.4	1.1	-0.1	0.9	1.6	0.5	-0.1	1.5	4.4	1.0
CAM-B3LYP-D3BJ	-0.5	1.6	3.7	1.2	-0.3	0.6	1.0	0.2	-0.4	1.1	3.7	1.0
wB97XD	-0.3	0.8	1.7	0.5	-1.0	1.2	3.2	1.2	-0.7	1.0	3.2	0.9
B2PLYP	-0.7	1.9	4.8	1.4	0.8	1.1	2.7	0.9	0.1	1.5	4.8	1.3
mPW2PLYP	0.1	1.4	5.2	1.7	0.6	1.0	2.1	0.7	0.3	1.2	5.2	1.3
DSDPBEP86	-1.1	2.1	3.7	0.8	-0.3	0.4	0.8	0.3	-0.7	1.3	3.7	1.0

2.1.2 SMD model

Table S3: Mean error (ME), mean absolute error (MAE), maximum unsigned error (MUE), and standard deviation (SD) of reaction barriers and energies (kcal mol^{-1}) for the thermal Conia–Ene reaction, computed using various density functional approximations at the XC/aug-cc-pVTZ//CCSD/6-31G(d,p) level, relative to CCSD(T)/aug-cc-pVTZ//CCSD/6-31G(d,p) reference values.

	$\Delta E^{\dagger}$				ΔE				Total			
XC	ME	MAE	MUE	SD	ME	MAE	MUE	SD	ME	MAE	MUE	SD
CCSD(T)	0.0	0.0	0.0	0.0	0.0	0.0	0.0	0.0	0.0	0.0	0.0	0.0
PBE	-9.7	9.7	10.5	0.4	-1.5	1.5	3.0	1.1	-5.6	5.6	10.5	4.2
PBE-D3BJ	-10.5	10.5	11.7	0.9	-1.8	1.8	3.7	1.1	-6.1	6.1	11.7	4.5
PW91	-9.6	9.6	10.4	0.4	-1.4	1.5	2.5	0.9	-5.5	5.5	10.4	4.1
M06L	-2.1	2.1	3.2	0.9	0.3	2.6	4.2	1.4	-0.9	2.3	4.2	1.2
M06L-D3	-2.1	2.1	3.1	0.9	0.3	2.5	4.2	1.4	-0.9	2.3	4.2	1.2
MN12-L	-1.9	1.9	5.6	1.8	-0.3	2.2	3.2	0.6	-1.1	2.0	5.6	1.4
MN15-L	-1.3	1.6	2.8	0.8	-1.4	2.5	4.9	1.7	-1.4	2.1	4.9	1.4
TPSS	-6.8	6.8	8.5	1.2	-0.9	1.2	3.8	1.5	-3.8	4.0	8.5	3.1
TPSS-D3BJ	-7.7	7.7	10.0	2.1	-1.2	1.9	4.5	1.6	-4.4	4.8	10.0	3.4
B3LYP	-1.1	1.1	3.0	1.0	0.6	1.2	3.2	1.1	-0.2	1.1	3.2	1.1
B3LYP-D3BJ	-2.4	2.4	3.1	0.7	0.2	0.4	1.0	0.3	-1.1	1.4	3.1	1.1
PBE0	-4.7	4.7	5.9	0.6	-2.3	2.3	4.2	1.4	-3.5	3.5	5.9	1.6

Continued on next page

Table S3 (continued)

	$\Delta E^{\dagger}$				ΔE				Total			
XC	ME	MAE	MUE	SD	ME	MAE	MUE	SD	ME	MAE	MUE	SD
PBE0-D3BJ	-5.3	5.3	6.1	0.9	-2.5	2.5	4.7	1.8	-3.9	3.9	6.1	2.0
B3PW91	-4.3	4.3	5.8	0.6	-1.5	1.5	2.8	0.9	-2.9	2.9	5.8	1.6
B3PW91-D3	-4.6	4.6	5.3	0.8	-1.4	1.6	3.8	1.5	-3.0	3.1	5.3	2.0
X3LYP	-1.0	1.1	3.0	1.0	0.5	1.1	2.8	1.0	-0.2	1.1	3.0	1.0
B1LYP	0.6	1.1	1.9	0.7	0.9	1.2	3.2	1.1	0.7	1.2	3.2	0.9
mPW1PW91	-3.9	3.9	5.3	0.6	-1.9	1.9	3.7	1.2	-2.9	2.9	5.3	1.4
B972	-2.3	2.3	3.6	0.7	-1.1	1.1	3.0	1.1	-1.7	1.7	3.6	1.1
MN15	-1.4	1.5	3.7	1.3	-0.8	1.4	3.5	1.1	-1.1	1.5	3.7	1.2
M08HX	0.8	1.1	1.9	0.4	0.0	1.1	2.6	0.7	0.4	1.1	2.6	0.6
M06	-0.9	1.2	2.4	0.7	0.6	0.8	1.7	0.5	-0.2	1.0	2.4	0.7
M06-D3	-1.4	1.4	2.8	0.9	0.8	1.1	1.8	0.4	-0.3	1.2	2.8	0.7
M06HF	-1.2	1.7	5.8	1.7	0.0	4.7	6.1	0.9	-0.6	3.2	6.1	2.0
M06HF-D3	-1.7	2.3	6.4	2.3	0.4	4.8	6.4	0.8	-0.6	3.5	6.4	2.1
M062X	0.0	1.3	2.5	0.5	0.5	2.1	3.3	0.8	0.2	1.7	3.3	0.8
M062X-D3	-0.5	1.7	3.3	0.8	0.8	2.1	3.5	0.9	0.2	1.9	3.5	0.9
M05	0.4	1.7	3.7	1.3	-0.3	2.8	5.4	1.6	0.1	2.2	5.4	1.6
M05-D3	-0.2	1.2	2.5	1.0	0.0	3.2	5.6	1.6	-0.1	2.2	5.6	1.7
M052X	-1.1	1.2	2.9	0.8	-0.5	1.4	3.0	0.7	-0.8	1.3	3.0	0.8
M052X-D3	-1.5	1.5	3.4	1.1	-0.2	1.2	1.6	0.5	-0.8	1.4	3.4	0.9
TPSSH	-4.5	4.5	6.4	1.9	-1.5	1.7	4.2	1.5	-3.0	3.1	6.4	2.2
CAM-B3LYP	0.3	1.5	2.3	0.7	0.0	0.5	0.9	0.3	0.1	1.0	2.3	0.7
CAM-B3LYP-D3BJ	-0.2	1.0	2.1	0.7	-0.2	0.3	1.0	0.4	-0.2	0.6	2.1	0.6
wB97XD	-0.2	0.6	1.0	0.2	-0.9	1.8	3.7	1.1	-0.5	1.2	3.7	1.0
B2PLYP	-0.1	2.0	7.3	2.1	0.8	0.9	2.1	0.7	0.3	1.4	7.3	1.6
mPW2PLYP	0.5	1.4	7.5	2.4	0.6	0.7	1.5	0.5	0.6	1.0	7.5	1.7
DSDPBEP86	-0.8	2.2	5.6	1.4	-0.3	0.7	1.1	0.3	-0.5	1.4	5.6	1.2

2.2 Basis sets

Table S4: Mean error (ME), mean absolute error (MAE), maximum unsigned error (MUE), and standard deviation (SD) (kcal mol^{-1}) for the thermal Conia–Ene reaction, computed using various density functional approximations at the XC/**6-31G**//CCSD/6-31G(d,p) level, relative to CCSD(T)/aug-cc-pVTZ//CCSD/6-31G(d,p) reference values.

XC	ME	MAE	MUE	SD
CCSD(T)	0.0	0.0	0.0	0.0

Continued on next page

Table S4 (continued)

XC	ME	MAE	MUE	SD
PBE	-6.7	7.3	16.2	5.3
PBE-D3BJ	-7.1	7.8	17.0	5.5
PW91	-6.6	7.2	16.4	5.3
M06L	-1.5	3.7	5.7	1.3
M06L-D3	-1.5	3.6	5.7	1.3
MN12-L	-0.3	1.9	6.2	1.9
MN15-L	-0.7	4.0	7.4	2.0
TPSS	-4.7	5.6	11.3	4.2
TPSS-D3BJ	-5.3	6.3	13.0	4.5
B3LYP	-1.3	2.7	6.9	2.0
B3LYP-D3BJ	-2.1	3.8	8.3	2.0
PBE0	-4.1	5.1	9.9	3.0
PBE0-D3BJ	-4.4	5.6	10.6	3.1
B3PW91	-3.6	4.5	9.9	2.9
B3PW91-D3	-3.6	5.0	9.7	2.6
X3LYP	-1.3	2.8	7.1	2.0
B1LYP	-0.3	2.2	5.7	1.9
mPW1PW91	-3.5	4.6	9.0	2.7
B972	-2.3	3.6	7.0	1.9
MN15	-1.8	2.6	7.5	1.8
M08HX	0.1	2.4	8.6	2.5
M06	-1.8	3.2	6.9	1.6
M06-D3	-1.7	3.2	7.0	1.6
M06HF	-0.6	4.1	9.6	2.5
M06HF-D3	-0.6	4.1	9.7	2.5
M062X	-0.5	2.3	7.4	2.3
M062X-D3	-0.5	2.3	7.4	2.3
M05	-1.4	3.3	6.7	1.7
M05-D3	-1.4	3.4	7.0	1.7
M052X	-1.7	3.0	8.9	2.4
M052X-D3	-1.6	3.0	9.0	2.4
TPSSH	-3.8	5.0	9.1	3.0
CAM-B3LYP	-1.0	2.9	6.7	2.0
CAM-B3LYP-D3BJ	-1.3	3.3	7.3	1.9
wB97XD	-1.1	3.4	6.2	2.0
B2PLYP	1.2	1.9	6.8	1.9
mPW2PLYP	1.3	1.9	7.6	2.2
DSDPBEP86	1.5	2.2	8.2	2.3

Table S5: Mean error (ME), mean absolute error (MAE), maximum unsigned error (MUE), and standard deviation (SD) (kcal mol^{-1}) for the thermal Conia–Ene reaction, computed using various density functional approximations at the XC/**6-31G(d,p)**//CCSD/6-31G(d,p) level, relative to CCSD(T)/aug-cc-pVTZ//CCSD/6-31G(d,p) reference values.

XC	ME	MAE	MUE	SD
CCSD(T)	0.0	0.0	0.0	0.0
PBE	-7.9	8.1	15.6	5.1
PBE-D3BJ	-8.3	8.6	16.4	5.4
PW91	-7.8	8.1	15.9	5.1
M06L	-2.4	3.8	7.9	2.0
M06L-D3	-2.4	3.8	7.8	2.0
MN12-L	-1.7	1.8	5.0	1.3
MN15-L	-1.7	3.3	8.1	2.3
TPSS	-5.9	6.6	12.3	3.8
TPSS-D3BJ	-6.4	7.3	13.7	4.2
B3LYP	-2.4	2.8	6.3	1.8
B3LYP-D3BJ	-3.2	3.8	7.6	2.4
PBE0	-5.3	5.5	9.4	3.2
PBE0-D3BJ	-5.6	6.0	10.1	3.5
B3PW91	-4.8	5.1	9.1	2.9
B3PW91-D3	-4.8	5.3	9.2	3.1
X3LYP	-2.5	2.8	6.4	1.8
B1LYP	-1.5	2.0	4.7	1.4
mPW1PW91	-4.8	5.0	8.7	2.9
B972	-3.5	3.8	7.6	2.3
MN15	-2.8	2.8	7.2	1.7
M08HX	-1.0	1.8	4.1	1.2
M06	-2.5	3.3	5.9	1.5
M06-D3	-2.4	3.3	6.0	1.5
M06HF	-2.2	3.0	9.5	2.5
M06HF-D3	-2.2	3.0	9.6	2.5
M062X	-1.6	1.8	7.0	1.8
M062X-D3	-1.6	1.8	7.0	1.7
M05	-2.3	4.0	9.8	2.4
M05-D3	-2.2	4.1	10.0	2.5
M052X	-2.7	2.7	7.6	2.0
M052X-D3	-2.7	2.7	7.7	2.0
TPSSH	-5.0	5.6	9.9	3.1

Continued on next page

Table S5 (continued)

XC	ME	MAE	MUE	SD
CAM-B3LYP	-2.2	2.6	6.3	2.1
CAM-B3LYP-D3BJ	-2.4	3.0	6.7	2.2
wB97XD	-2.4	3.1	8.6	2.5
B2PLYP	-1.3	2.4	4.4	1.1
mPW2PLYP	-1.1	2.3	4.7	1.1
DSDPBEP86	-1.5	3.0	6.4	1.8

Table S6: Mean error (ME), mean absolute error (MAE), maximum unsigned error (MUE), and standard deviation (SD) (kcal mol^{-1}) for the thermal Conia–Ene reaction, computed using various density functional approximations at the XC/**6-31+G(d,p)**//CCSD/6-31G(d,p) level, relative to CCSD(T)/aug-cc-pVTZ//CCSD/6-31G(d,p) reference values.

XC	ME	MAE	MUE	SD
CCSD(T)	0.0	0.0	0.0	0.0
PBE	-7.0	7.0	13.4	4.7
PBE-D3BJ	-7.4	7.6	13.8	4.9
PW91	-6.9	7.0	13.6	4.8
M06L	-1.9	3.2	6.6	1.6
M06L-D3	-1.9	3.1	6.6	1.6
MN12-L	-1.2	1.5	3.8	1.0
MN15-L	-1.1	2.6	6.3	1.9
TPSS	-5.1	5.7	10.4	3.4
TPSS-D3BJ	-5.7	6.3	11.8	3.7
B3LYP	-1.6	1.7	5.4	1.4
B3LYP-D3BJ	-2.4	2.7	5.1	1.6
PBE0	-4.6	4.6	7.9	2.7
PBE0-D3BJ	-5.0	5.1	8.1	3.0
B3PW91	-4.2	4.2	8.5	2.5
B3PW91-D3	-4.1	4.5	7.2	2.5
X3LYP	-1.6	1.7	5.3	1.4
B1LYP	-0.6	0.9	3.6	1.1
mPW1PW91	-4.1	4.2	7.6	2.5
B972	-2.8	3.0	6.0	1.8
MN15	-2.0	2.0	4.2	1.1
M08HX	-0.4	1.0	2.3	0.6
M06	-1.8	2.5	3.9	0.9
M06-D3	-1.8	2.5	4.0	0.9

Continued on next page

Table S6 (continued)

XC	ME	MAE	MUE	SD
M06HF	-1.6	3.0	7.5	2.2
M06HF-D3	-1.6	3.1	7.6	2.2
M062X	-1.0	1.1	4.8	1.2
M062X-D3	-1.0	1.1	4.8	1.2
M05	-1.6	3.1	8.9	2.4
M05-D3	-1.5	3.2	8.8	2.5
M052X	-2.1	2.1	5.1	1.2
M052X-D3	-2.1	2.1	5.2	1.2
TPSSH	-4.3	4.7	8.1	2.7
CAM-B3LYP	-1.3	1.5	4.4	1.6
CAM-B3LYP-D3BJ	-1.6	1.9	4.6	1.6
wB97XD	-1.7	2.2	6.8	2.1
B2PLYP	-0.8	1.4	2.6	0.7
mPW2PLYP	-0.5	1.3	2.8	0.7
DSDPBEP86	-1.1	2.1	4.6	1.4

Table S7: Mean error (ME), mean absolute error (MAE), maximum unsigned error (MUE), and standard deviation (SD) (kcal mol^{-1}) for the thermal Conia–Ene reaction, computed using various density functional approximations at the XC/**6-311G(d,p)**//CCSD/6-31G(d,p) level, relative to CCSD(T)/aug-cc-pVTZ//CCSD/6-31G(d,p) reference values.

XC	ME	MAE	MUE	SD
CCSD(T)	0.0	0.0	0.0	0.0
PBE	-6.5	6.5	14.2	5.1
PBE-D3BJ	-7.0	7.1	15.0	5.2
PW91	-6.4	6.4	14.4	5.1
M06L	-1.1	2.4	4.3	1.2
M06L-D3	-1.1	2.3	4.3	1.2
MN12-L	-1.0	1.5	4.3	1.1
MN15-L	-1.3	2.5	5.9	1.8
TPSS	-4.5	5.0	9.4	3.5
TPSS-D3BJ	-5.1	5.7	10.7	3.7
B3LYP	-0.8	1.4	5.3	1.5
B3LYP-D3BJ	-1.6	2.0	6.1	1.7
PBE0	-4.1	4.1	8.3	2.5
PBE0-D3BJ	-4.5	4.5	9.0	2.7
B3PW91	-3.6	3.6	8.5	2.4

Continued on next page

Table S7 (continued)

XC	ME	MAE	MUE	SD
B3PW91-D3	-3.5	3.8	8.0	2.2
X3LYP	-0.9	1.4	5.1	1.5
B1LYP	0.1	0.9	3.4	1.0
mPW1PW91	-3.6	3.6	7.5	2.2
B972	-2.4	2.4	5.6	1.6
MN15	-1.3	1.7	5.4	1.4
M08HX	0.1	0.8	2.3	0.7
M06	-0.8	1.5	4.7	1.1
M06-D3	-0.8	1.5	4.8	1.1
M06HF	-1.2	3.7	8.3	2.2
M06HF-D3	-1.2	3.7	8.4	2.2
M062X	-0.4	1.3	5.3	1.3
M062X-D3	-0.3	1.4	5.3	1.3
M05	-0.8	2.4	5.8	1.5
M05-D3	-0.8	2.4	5.9	1.6
M052X	-1.5	1.7	6.2	1.6
M052X-D3	-1.5	1.7	6.3	1.6
TPSSH	-3.7	4.1	7.3	2.5
CAM-B3LYP	-0.5	1.1	4.6	1.4
CAM-B3LYP-D3BJ	-0.8	1.1	5.2	1.4
wB97XD	-1.0	1.5	4.4	1.4
B2PLYP	-0.3	1.1	2.9	0.8
mPW2PLYP	-0.1	0.8	2.4	0.7
DSDPBEP86	-0.9	1.8	2.9	0.8

Table S8: Mean error (ME), mean absolute error (MAE), maximum unsigned error (MUE), and standard deviation (SD) (kcal mol^{-1}) for the thermal Conia–Ene reaction, computed using various density functional approximations at the XC/**6-311++G(2d,2p)**//CCSD/6-31G(d,p) level, relative to CCSD(T)/aug-cc-pVTZ//CCSD/6-31G(d,p) reference values.

XC	ME	MAE	MUE	SD
CCSD(T)	0.0	0.0	0.0	0.0
PBE	-6.1	6.1	13.1	4.5
PBE-D3BJ	-6.5	6.5	12.8	4.7
PW91	-5.9	5.9	13.2	4.6
M06L	-0.9	1.9	4.0	1.1
M06L-D3	-0.9	1.9	3.9	1.1

Continued on next page

Table S8 (continued)

XC	ME	MAE	MUE	SD
MN12-L	-0.8	1.5	3.4	0.9
MN15-L	-1.0	2.1	4.9	1.5
TPSS	-4.2	4.4	8.8	3.3
TPSS-D3BJ	-4.7	5.0	10.1	3.5
B3LYP	-0.4	1.3	4.8	1.3
B3LYP-D3BJ	-1.2	1.5	4.1	1.3
PBE0	-3.8	3.8	7.8	1.9
PBE0-D3BJ	-4.2	4.2	7.4	2.2
B3PW91	-3.2	3.2	8.2	2.0
B3PW91-D3	-3.2	3.2	6.4	2.1
X3LYP	-0.4	1.2	4.7	1.3
B1LYP	0.6	1.3	3.3	1.0
mPW1PW91	-3.2	3.2	7.3	1.7
B972	-2.0	2.0	5.5	1.3
MN15	-1.2	1.7	3.9	1.2
M08HX	0.3	1.0	2.0	0.5
M06	-0.7	1.2	3.0	0.8
M06-D3	-0.6	1.2	3.1	0.8
M06HF	-1.0	3.4	7.1	2.2
M06HF-D3	-1.0	3.4	7.2	2.2
M062X	0.0	1.7	4.0	1.0
M062X-D3	0.0	1.7	4.0	1.0
M05	-0.5	2.0	5.7	1.4
M05-D3	-0.5	2.0	5.6	1.4
M052X	-1.2	1.7	4.9	1.3
M052X-D3	-1.1	1.7	4.9	1.3
TPSSH	-3.4	3.5	7.0	2.4
CAM-B3LYP	-0.1	1.1	3.9	1.0
CAM-B3LYP-D3BJ	-0.4	0.8	3.1	0.9
wB97XD	-0.7	1.1	3.8	1.2
B2PLYP	0.1	0.8	2.4	0.7
mPW2PLYP	0.4	0.7	1.6	0.5
DSDPBEP86	-0.5	0.9	1.7	0.4

Table S9: Mean error (ME), mean absolute error (MAE), maximum unsigned error (MUE), and standard deviation (SD) (kcal mol^{-1}) for the thermal Conia–Ene reaction, computed using various density functional approximations at the XC/**cc-pVDZ**//CCSD/6-31G(d,p) level, relative to CCSD(T)/aug-cc-pVTZ//CCSD/6-31G(d,p) reference values.

XC	ME	MAE	MUE	SD
CCSD(T)	0.0	0.0	0.0	0.0
PBE	-8.6	8.6	17.9	6.0
PBE-D3BJ	-9.0	9.2	18.7	6.1
PW91	-8.5	8.6	18.2	6.1
M06L	-2.8	4.0	6.7	1.6
M06L-D3	-2.7	3.9	6.6	1.6
MN12-L	-2.0	2.2	6.5	1.5
MN15-L	-2.0	3.0	6.8	1.9
TPSS	-6.4	7.1	12.3	4.2
TPSS-D3BJ	-7.0	7.8	13.5	4.5
B3LYP	-3.0	3.2	8.4	2.4
B3LYP-D3BJ	-3.8	4.2	9.7	2.6
PBE0	-5.9	6.0	11.4	3.5
PBE0-D3BJ	-6.2	6.5	12.1	3.7
B3PW91	-5.4	5.5	11.4	3.5
B3PW91-D3	-5.4	5.9	11.2	3.2
X3LYP	-3.1	3.2	8.6	2.4
B1LYP	-2.0	2.3	6.4	1.8
mPW1PW91	-5.4	5.5	10.5	3.3
B972	-4.0	4.3	8.2	2.5
MN15	-3.5	3.5	9.7	2.4
M08HX	-1.7	1.8	5.7	1.4
M06	-3.2	3.8	8.1	1.9
M06-D3	-3.2	3.8	8.2	1.9
M06HF	-3.0	3.7	11.8	3.0
M06HF-D3	-3.0	3.7	11.9	3.0
M062X	-2.3	2.3	8.9	2.2
M062X-D3	-2.2	2.3	8.9	2.3
M05	-3.0	4.8	10.0	2.2
M05-D3	-3.0	4.9	10.0	2.2
M052X	-3.4	3.4	9.7	2.3
M052X-D3	-3.4	3.4	9.8	2.3
TPSSH	-5.5	6.1	10.2	3.4

Continued on next page

Table S9 (continued)

XC	ME	MAE	MUE	SD
CAM-B3LYP	-2.8	3.0	8.3	2.3
CAM-B3LYP-D3BJ	-3.0	3.4	9.0	2.2
wB97XD	-2.9	3.5	7.4	2.1
B2PLYP	-2.1	2.9	5.9	1.4
mPW2PLYP	-1.8	2.5	5.3	1.3
DSDPBEP86	-2.4	3.4	5.5	1.6

Table S10: Mean error (ME), mean absolute error (MAE), maximum unsigned error (MUE), and standard deviation (SD) (kcal mol^{-1}) for the thermal Conia–Ene reaction, computed using various density functional approximations at the XC/**aug-cc-pVDZ**//CCSD/6-31G(d,p) level, relative to CCSD(T)/aug-cc-pVTZ//CCSD/6-31G(d,p) reference values.

XC	ME	MAE	MUE	SD
CCSD(T)	0.0	0.0	0.0	0.0
PBE	-7.5	7.5	13.9	4.8
PBE-D3BJ	-7.9	7.9	14.2	5.1
PW91	-7.4	7.4	14.0	4.8
M06L	-2.1	2.9	5.5	1.4
M06L-D3	-2.1	2.9	5.5	1.4
MN12-L	-1.5	1.9	4.1	1.1
MN15-L	-1.6	2.5	5.7	1.8
TPSS	-5.5	5.7	10.8	3.7
TPSS-D3BJ	-6.0	6.4	12.2	4.0
B3LYP	-2.0	2.0	5.8	1.4
B3LYP-D3BJ	-2.8	2.8	5.2	1.8
PBE0	-5.1	5.1	8.4	2.6
PBE0-D3BJ	-5.4	5.4	8.3	2.9
B3PW91	-4.6	4.6	8.9	2.4
B3PW91-D3	-4.6	4.6	7.6	2.8
X3LYP	-2.0	2.0	5.7	1.4
B1LYP	-1.0	1.1	4.0	1.0
mPW1PW91	-4.6	4.6	8.0	2.4
B972	-3.3	3.3	6.0	1.9
MN15	-2.6	2.6	5.2	1.5
M08HX	-0.9	1.0	2.1	0.7
M06	-2.4	2.5	4.4	1.5
M06-D3	-2.3	2.5	4.4	1.4

Continued on next page

Table S10 (continued)

XC	ME	MAE	MUE	SD
M06HF	-2.4	3.4	8.1	2.3
M06HF-D3	-2.4	3.4	8.2	2.3
M062X	-1.5	1.6	5.0	1.3
M062X-D3	-1.4	1.6	5.0	1.3
M05	-2.2	3.6	9.5	2.4
M05-D3	-2.2	3.7	9.5	2.5
M052X	-2.6	2.6	5.5	1.2
M052X-D3	-2.5	2.5	5.6	1.2
TPSSH	-4.7	4.9	8.6	2.9
CAM-B3LYP	-1.7	1.7	4.9	1.4
CAM-B3LYP-D3BJ	-2.0	2.0	4.2	1.4
wB97XD	-2.1	2.3	6.2	1.9
B2PLYP	-1.5	1.7	3.2	1.0
mPW2PLYP	-1.2	1.3	2.4	0.8
DSDPBEP86	-2.0	2.5	4.3	1.4

Table S11: Mean error (ME), mean absolute error (MAE), maximum unsigned error (MUE), and standard deviation (SD) (kcal mol^{-1}) for the thermal Conia–Ene reaction, computed using various density functional approximations at the XC/**cc-pVTZ**//CCSD/6-31G(d,p) level, relative to CCSD(T)/aug-cc-pVTZ//CCSD/6-31G(d,p) reference values.

XC	ME	MAE	MUE	SD
CCSD(T)	0.0	0.0	0.0	0.0
PBE	-1.0	5.7	13.9	4.6
PBE-D3BJ	-1.3	6.3	14.3	4.7
PW91	-0.8	5.8	13.9	4.5
M06L	0.8	2.1	7.0	1.6
M06L-D3	0.9	2.2	7.0	1.7
MN12-L	0.6	2.7	7.0	1.8
MN15-L	0.6	2.3	7.0	2.0
TPSS	0.2	4.4	9.5	3.2
TPSS-D3BJ	-0.2	5.2	10.2	3.5
B3LYP	1.6	2.6	9.0	2.6
B3LYP-D3BJ	0.9	2.9	10.0	2.6
PBE0	0.2	4.4	11.0	2.8
PBE0-D3BJ	-0.1	4.9	12.0	3.0
B3PW91	0.7	4.1	13.0	3.4

Continued on next page

Table S11 (continued)

XC	ME	MAE	MUE	SD
B3PW91-D3	0.5	4.3	14.0	3.4
X3LYP	2.0	2.9	15.0	3.7
B1LYP	2.5	2.8	16.0	3.8
mPW1PW91	0.8	4.3	17.0	3.9
B972	1.4	3.3	18.0	4.3
MN15	1.7	3.6	19.0	4.4
M08HX	2.2	2.7	20.0	4.7
M06	2.1	3.0	21.0	5.0
M06-D3	2.2	3.1	22.0	5.2
M06HF	2.4	4.8	23.0	5.3
M06HF-D3	2.4	4.8	24.0	5.5
M062X	2.6	3.8	25.0	5.8
M062X-D3	2.7	3.9	26.0	6.0
M05	3.1	3.5	27.0	6.3
M05-D3	3.1	3.6	28.0	6.5
M052X	2.1	4.1	29.0	6.7
M052X-D3	2.2	4.2	30.0	6.9
TPSSH	1.9	5.2	31.0	7.1
CAM-B3LYP	3.1	3.7	32.0	7.5
CAM-B3LYP-D3BJ	2.8	3.5	33.0	7.8
wB97XD	2.7	3.5	34.0	8.0
B2PLYP	-0.1	1.5	4.3	1.2
mPW2PLYP	0.2	1.1	4.7	1.3
DSDPBEP86	-0.9	1.5	3.3	1.0

Table S12: Mean error (ME), mean absolute error (MAE), maximum unsigned error (MUE), and standard deviation (SD) (kcal mol^{-1}) for the thermal Conia–Ene reaction, computed using various density functional approximations at the XC/**def2-SVP**//CCSD/6-31G(d,p) level, relative to CCSD(T)/aug-cc-pVTZ//CCSD/6-31G(d,p) reference values.

XC	ME	MAE	MUE	SD
CCSD(T)	0.0	0.0	0.0	0.0
PBE	-1.5	7.5	18.0	5.6
PBE-D3BJ	-1.7	8.1	18.8	5.8
PW91	-1.3	7.6	18.3	5.6
M06L	0.3	3.5	9.0	2.2
M06L-D3	0.4	3.5	9.0	2.2

Continued on next page

Table S12 (continued)

XC	ME	MAE	MUE	SD
MN12-L	0.5	3.3	9.0	2.6
MN15-L	0.6	2.9	9.0	2.4
TPSS	-0.3	6.1	12.3	3.7
TPSS-D3BJ	-0.6	6.9	13.3	4.1
B3LYP	0.9	3.5	9.0	3.2
B3LYP-D3BJ	0.4	4.5	10.0	3.3
PBE0	-0.2	5.9	11.6	3.5
PBE0-D3BJ	-0.4	6.5	12.3	3.8
B3PW91	0.2	5.6	13.0	3.7
B3PW91-D3	0.1	6.0	14.0	3.9
X3LYP	1.2	3.9	15.0	4.0
B1LYP	1.7	3.1	16.0	4.2
mPW1PW91	0.4	5.9	17.0	4.2
B972	1.0	4.8	18.0	4.3
MN15	1.3	4.6	19.0	4.8
M08HX	1.7	3.4	20.0	4.9
M06	1.3	4.8	21.0	4.9
M06-D3	1.4	4.9	22.0	5.1
M06HF	2.1	5.2	23.0	5.8
M06HF-D3	2.2	5.3	24.0	6.0
M062X	2.1	4.3	25.0	6.2
M062X-D3	2.1	4.4	26.0	6.4
M05	2.5	5.6	27.0	6.1
M05-D3	2.5	5.7	28.0	6.3
M052X	1.7	5.3	29.0	6.7
M052X-D3	1.8	5.3	30.0	7.0
TPSSH	1.5	6.8	31.0	6.9
CAM-B3LYP	2.4	4.9	32.0	7.5
CAM-B3LYP-D3BJ	2.3	5.3	33.0	7.7
wB97XD	2.3	5.3	34.0	7.8
B2PLYP	-1.5	2.2	5.7	1.4
mPW2PLYP	-1.2	1.9	5.2	1.4
DSDPBEP86	-1.7	2.7	5.2	1.6

Table S13: Mean error (ME), mean absolute error (MAE), maximum unsigned error (MUE), and standard deviation (SD) (kcal mol^{-1}) for the thermal Conia–Ene reaction, computed using various density functional approximations at the XC/**def2-TZVP**//CCSD/6-31G(d,p) level, relative to CCSD(T)/aug-cc-pVTZ//CCSD/6-31G(d,p) reference values.

XC	ME	MAE	MUE	SD
CCSD(T)	0.0	0.0	0.0	0.0
PBE	-0.8	5.8	13.5	4.6
PBE-D3BJ	-1.1	6.4	13.6	4.7
PW91	-0.6	5.9	13.5	4.5
M06L	1.2	2.4	10.0	2.3
M06L-D3	1.3	2.4	10.0	2.3
MN12-L	1.0	2.7	10.0	2.4
MN15-L	0.9	2.5	10.0	2.5
TPSS	0.4	4.6	10.0	3.3
TPSS-D3BJ	0.1	5.3	10.3	3.6
B3LYP	1.8	2.5	10.0	3.0
B3LYP-D3BJ	1.1	2.8	10.0	3.1
PBE0	0.4	4.5	11.0	3.1
PBE0-D3BJ	0.2	5.0	12.0	3.2
B3PW91	0.9	4.2	13.0	3.6
B3PW91-D3	0.7	4.4	14.0	3.6
X3LYP	2.1	2.8	15.0	4.0
B1LYP	2.7	2.7	16.0	4.1
mPW1PW91	1.1	4.4	17.0	4.1
B972	1.6	3.4	18.0	4.5
MN15	3.4	4.8	20.9	6.2
M08HX	4.7	4.9	25.3	7.1
M06	4.1	4.7	22.3	6.8
M06-D3	2.3	3.1	22.0	5.4
M06HF	4.2	6.3	23.0	6.7
M06HF-D3	3.0	5.2	24.0	5.6
M062X	4.6	5.5	25.0	7.4
M062X-D3	3.0	3.9	26.0	6.2
M05	5.0	5.3	27.0	7.6
M05-D3	3.4	3.9	28.0	6.6
M052X	4.0	5.4	29.0	8.2
M052X-D3	2.4	4.1	30.0	7.1
TPSSH	3.4	6.4	31.0	8.0

Continued on next page

Table S13 (continued)

XC	ME	MAE	MUE	SD
CAM-B3LYP	3.3	3.5	32.0	7.7
CAM-B3LYP-D3BJ	3.0	3.5	33.0	8.0
wB97XD	3.1	3.8	34.0	8.1
B2PLYP	-0.4	1.1	3.3	0.8
mPW2PLYP	-0.1	0.6	2.5	0.7
DSDPBEP86	-1.1	1.6	2.9	0.7

Table S14: Mean error (ME), mean absolute error (MAE), maximum unsigned error (MUE), and standard deviation (SD) (kcal mol^{-1}) for the thermal Conia–Ene reaction, computed using various density functional approximations at the XC/**def2-TZVPP**//CCSD/6-31G(d,p) level, relative to CCSD(T)/aug-cc-pVTZ//CCSD/6-31G(d,p) reference values.

XC	ME	MAE	MUE	SD
CCSD(T)	0.0	0.0	0.0	0.0
PBE	-6.2	6.2	13.6	4.7
PBE-D3BJ	-6.6	6.6	13.5	4.9
PW91	-6.0	6.1	13.6	4.7
M06L	-0.6	1.8	3.4	0.9
M06L-D3	-0.6	1.8	3.4	0.9
MN12-L	-0.8	2.3	4.6	1.3
MN15-L	-1.2	1.8	4.3	1.2
TPSS	-4.2	4.3	9.3	3.5
TPSS-D3BJ	-4.8	4.9	9.9	3.7
B3LYP	-0.5	1.5	5.1	1.4
B3LYP-D3BJ	-1.3	1.6	4.5	1.3
PBE0	-3.9	3.9	8.3	1.9
PBE0-D3BJ	-4.2	4.2	8.0	2.1
B3PW91	-3.3	3.3	8.6	2.1
B3PW91-D3	-3.3	3.3	6.9	2.1
X3LYP	-0.5	1.5	5.0	1.4
B1LYP	0.5	1.5	3.6	1.0
mPW1PW91	-3.3	3.3	7.8	1.8
B972	-2.1	2.1	5.9	1.4
MN15	-1.1	2.1	4.4	1.3
M08HX	0.6	1.6	2.7	0.7
M06	-0.3	1.1	2.9	0.9
M06-D3	-0.3	1.1	3.0	0.9

Continued on next page

Table S14 (continued)

XC	ME	MAE	MUE	SD
M06HF	-0.8	4.2	7.7	2.3
M06HF-D3	-0.8	4.2	7.7	2.3
M062X	0.0	2.2	4.5	1.1
M062X-D3	0.0	2.3	4.5	1.0
M05	-0.3	1.9	4.9	1.3
M05-D3	-0.3	1.9	4.9	1.4
M052X	-1.2	1.9	5.4	1.4
M052X-D3	-1.1	1.9	5.4	1.4
TPSSH	-3.4	3.4	7.5	2.5
CAM-B3LYP	-0.1	1.4	4.2	1.0
CAM-B3LYP-D3BJ	-0.4	1.0	3.6	1.0
wB97XD	-0.7	1.1	3.4	1.0
B2PLYP	-0.3	1.1	3.3	0.9
mPW2PLYP	0.0	0.8	2.6	0.7
DSDPBEP86	-1.0	1.1	2.7	0.7

Table S15: Mean error (ME), mean absolute error (MAE), maximum unsigned error (MUE), and standard deviation (SD) (kcal mol^{-1}) for the thermal Conia–Ene reaction, computed using various density functional approximations at the XC/**pc-2**//CCSD/6-31G(d,p) level, relative to CCSD(T)/aug-cc-pVTZ//CCSD/6-31G(d,p) reference values.

XC	ME	MAE	MUE	SD
CCSD(T)	0.0	0.0	0.0	0.0
PBE	-6.0	6.1	13.6	4.6
PBE-D3BJ	-6.5	6.5	13.1	4.8
PW91	-5.9	6.0	13.7	4.5
M06L	-0.4	1.7	3.0	0.8
M06L-D3	-0.4	1.7	3.0	0.8
MN12-L	-0.7	2.4	4.6	1.3
MN15-L	-1.1	1.7	4.2	1.2
TPSS	-4.0	4.1	9.3	3.3
TPSS-D3BJ	-4.6	4.7	9.6	3.6
B3LYP	-0.3	1.7	5.1	1.4
B3LYP-D3BJ	-1.1	1.7	4.2	1.1
PBE0	-3.7	3.7	8.3	1.9
PBE0-D3BJ	-4.1	4.1	7.7	2.0
B3PW91	-3.2	3.2	8.6	2.1

Continued on next page

Table S15 (continued)

XC	ME	MAE	MUE	SD
B3PW91-D3	-3.1	3.1	6.6	2.0
X3LYP	-0.4	1.7	5.0	1.3
B1LYP	0.7	1.7	4.1	1.1
mPW1PW91	-3.2	3.2	7.8	1.7
B972	-1.9	1.9	5.9	1.4
MN15	-1.1	2.1	4.1	1.3
M08HX	0.7	1.8	2.9	0.8
M06	-0.3	1.2	2.8	1.0
M06-D3	-0.3	1.1	2.9	0.9
M06HF	-0.4	4.2	7.0	2.1
M06HF-D3	-0.4	4.2	7.1	2.1
M062X	0.2	2.4	4.3	1.0
M062X-D3	0.2	2.4	4.3	1.1
M05	-0.1	1.7	4.6	1.3
M05-D3	-0.1	1.7	4.6	1.4
M052X	-1.0	2.0	5.1	1.4
M052X-D3	-1.0	2.0	5.2	1.3
TPSSH	-3.2	3.2	7.4	2.4
CAM-B3LYP	0.0	1.5	4.3	1.1
CAM-B3LYP-D3BJ	-0.3	1.1	3.5	0.9
wB97XD	-0.6	1.0	3.1	0.9
B2PLYP	-0.1	1.1	3.1	1.0
mPW2PLYP	0.2	0.9	2.4	0.7
DSDPBEP86	-0.7	0.8	2.0	0.6

Table S16: Mean error (ME), mean absolute error (MAE), maximum unsigned error (MUE), and standard deviation (SD) (kcal mol^{-1}) for the thermal Conia–Ene reaction, computed using various density functional approximations at the XC/**pc-3**//CCSD/6-31G(d,p) level, relative to CCSD(T)/aug-cc-pVTZ//CCSD/6-31G(d,p) reference values.

XC	ME	MAE	MUE	SD
CCSD(T)	0.0	0.0	0.0	0.0
PBE	-6.0	6.0	13.5	4.5
PBE-D3BJ	-6.5	6.5	12.9	4.7
PW91	-5.8	6.0	13.6	4.5
M06L	-0.3	1.5	3.1	0.9
M06L-D3	-0.3	1.5	3.1	0.9

Continued on next page

Table S16 (continued)

XC	ME	MAE	MUE	SD
MN12-L	-0.5	2.2	4.5	1.3
MN15-L	-0.7	1.5	3.9	1.3
TPSS	-4.0	4.1	9.2	3.3
TPSS-D3BJ	-4.6	4.7	9.5	3.6
B3LYP	-0.3	1.7	5.1	1.3
B3LYP-D3BJ	-1.1	1.6	4.1	1.1
PBE0	-3.7	3.7	8.2	1.8
PBE0-D3BJ	-4.1	4.1	7.5	2.0
B3PW91	-3.2	3.2	8.6	2.1
B3PW91-D3	-3.2	3.2	6.4	1.9
X3LYP	-0.3	1.6	5.0	1.3
B1LYP	0.6	1.7	4.1	1.1
mPW1PW91	-3.2	3.2	7.8	1.7
B972	-1.9	2.0	5.9	1.4
MN15	-1.0	2.1	3.8	1.1
M08HX	0.8	1.7	2.9	0.7
M06	-0.1	1.1	2.5	0.9
M06-D3	0.0	1.1	2.6	0.9
M06HF	-0.3	3.9	6.9	2.0
M06HF-D3	-0.3	3.9	7.0	2.0
M062X	0.2	2.3	4.1	0.9
M062X-D3	0.3	2.1	3.6	0.9
M05	-0.1	1.8	4.6	1.3
M05-D3	0.0	1.7	4.5	1.3
M052X	-1.0	1.9	4.8	1.3
M052X-D3	-1.0	2.0	4.9	1.3
TPSSH	-3.2	3.3	7.3	2.4
CAM-B3LYP	0.0	1.5	4.2	1.0
CAM-B3LYP-D3BJ	-0.3	1.1	3.5	0.9
wB97XD	-0.6	1.0	3.1	0.9
B2PLYP	-0.1	1.1	3.2	1.0
mPW2PLYP	0.2	0.9	2.4	0.7
DSDPBEP86	-0.9	0.9	2.1	0.6

3 Gas phase energies

Table S17: XC/aug-cc-pVTZ single-point energies on CCSD/6-31G(d,p) optimized geometries for the reaction profile of **hex-5-ynal** (1a).

XC	1	TS_{enol}	2	TS_{conia}	3
CCSD(T)	-308.0874483	-307.9784291	-308.0747491	-308.0112212	-308.1013997
PBE	-308.3124468	-308.2183619	-308.3023772	-308.2560664	-308.3333578
PBE-D3BJ	-308.3253355	-308.2309350	-308.3144611	-308.2699442	-308.3463976
PW91	-308.5825938	-308.4879131	-308.5725976	-308.5259271	-308.6027360
M06L	-308.6599949	-308.5520152	-308.6432001	-308.5849378	-308.6757782
M06L-D3	-308.6603658	-308.5524050	-308.6436088	-308.5852478	-308.6761252
MN12-L	-308.3778945	-308.2717599	-308.3696456	-308.3076554	-308.3939951
MN15-L	-308.4193417	-308.3114247	-308.4061426	-308.3473979	-308.4395324
TPSS	-308.7573011	-308.6570381	-308.7448634	-308.6948795	-308.7769252
TPSS-D3BJ	-308.7734285	-308.6727495	-308.7599475	-308.7121922	-308.7932119
B3LYP	-308.7166599	-308.6105934	-308.7061980	-308.6423191	-308.7305104
B3LYP-D3BJ	-308.7381953	-308.6316168	-308.7264157	-308.6657840	-308.7524484
PBE0	-308.3356170	-308.2330557	-308.3253702	-308.2703283	-308.3581242
PBE0-D3BJ	-308.3467483	-308.2438967	-308.3357844	-308.2821865	-308.3693248
B3PW91	-308.5871474	-308.4846912	-308.5770506	-308.5206429	-308.6075175
B3PW91-D3	-308.5989284	-308.4954013	-308.5869387	-308.5319794	-308.6187328
X3LYP	-308.5699567	-308.4636428	-308.5595296	-308.4956611	-308.5840187
B1LYP	-308.5494799	-308.4411526	-308.5387220	-308.4719026	-308.5626914
mPW1PW91	-308.6264870	-308.5228205	-308.6161948	-308.5595347	-308.6479801
B972	-308.5944196	-308.4897675	-308.5837126	-308.5236686	-308.6141811
MN15	-308.3333546	-308.2299433	-308.3247664	-308.2626786	-308.3502839
M08HX	-308.6303589	-308.5198178	-308.6194522	-308.5544758	-308.6441135
M06	-308.4867003	-308.3811395	-308.4729494	-308.4116701	-308.4991423
M06-D3	-308.4880583	-308.3823954	-308.4741665	-308.4128490	-308.5004322
M06HF	-308.6519561	-308.5460176	-308.6472636	-308.5828428	-308.6650999
M06HF-D3	-308.6526762	-308.5467236	-308.6479693	-308.5834618	-308.6657936
M062X	-308.5769962	-308.4699351	-308.5672261	-308.5025313	-308.5896733
M062X-D3	-308.5773044	-308.4702633	-308.5675769	-308.5027869	-308.5899581
M05	-308.4770105	-308.3722333	-308.4614061	-308.3994250	-308.4945818
M05-D3	-308.4809959	-308.3758826	-308.4648340	-308.4032398	-308.4983971
M052X	-308.6739056	-308.5671297	-308.6650453	-308.6023466	-308.6883795
M052X-D3	-308.6747404	-308.5679349	-308.6658445	-308.6030671	-308.6891836
TPSSH	-308.7230036	-308.6198487	-308.7107086	-308.6573751	-308.7433201
CAM-B3LYP	-308.5484524	-308.4405240	-308.5374827	-308.4708873	-308.5638470
CAM-B3LYP-D3BJ	-308.5583571	-308.4501611	-308.5467534	-308.4813131	-308.5737485
wB97XD	-308.6053760	-308.4973620	-308.5923210	-308.5287827	-308.6241110

Continued on next page

Table S17 (continued)

XC	1	TS_{enol}	2	TS_{conia}	3
B2PLYP	-308.4547969	-308.3479191	-308.4428529	-308.3800764	-308.4669608
mPW2PLYP	-308.4521734	-308.3439077	-308.4402765	-308.3761703	-308.4648373
DSDPBEP86	-308.1472887	-308.039754	-308.1342165	-308.073539	-308.1621251

Table S18: XC/aug-cc-pVTZ single-point energies on CCSD/6-31G(d,p) optimized geometries for the reaction profile of **hept-6-ynal** (1b).

XC	1	TS_{enol}	2	TS_{conia}	3
CCSD(T)	-347.3289427	-347.2172255	-347.3157755	-347.2769468	-347.3788838
PBE	-347.5810085	-347.4847456	-347.5702498	-347.5470389	-347.6331876
PBE-D3BJ	-347.5967061	-347.5004246	-347.5857985	-347.5652737	-347.6508163
PW91	-347.8886201	-347.7917744	-347.8779797	-347.8547402	-347.9403519
M06L	-347.9785632	-347.8690702	-347.9620699	-347.9271844	-348.0254014
M06L-D3	-347.9791263	-347.8696655	-347.9626724	-347.9277059	-348.0259300
MN12-L	-347.6511575	-347.5430367	-347.6434288	-347.6058735	-347.7028148
MN15-L	-347.7027744	-347.5929421	-347.6901144	-347.6545105	-347.7579633
TPSS	-348.0892933	-347.9864700	-348.0758291	-348.0486375	-348.1385567
TPSS-D3BJ	-348.1088527	-348.0060227	-348.0952313	-348.0714295	-348.1605877
B3LYP	-348.0435834	-347.9346771	-348.0318986	-347.9925165	-348.0886959
B3LYP-D3BJ	-348.0696175	-347.9606875	-348.0577410	-348.0231338	-348.1182347
PBE0	-347.6122505	-347.5070390	-347.6012099	-347.5700315	-347.6680516
PBE0-D3BJ	-347.6257754	-347.5205838	-347.6146456	-347.5857274	-347.6832343
B3PW91	-347.8992102	-347.7940459	-347.8879692	-347.8554550	-347.9515622
B3PW91-D3	-347.9141045	-347.8080622	-347.9018499	-347.8713589	-347.9678211
X3LYP	-347.8750370	-347.7658929	-347.8634985	-347.8242465	-347.9208537
B1LYP	-347.8486706	-347.7373538	-347.8366152	-347.7944167	-347.8932403
mPW1PW91	-347.9435099	-347.8371090	-347.9322334	-347.8995869	-347.9978716
B972	-347.9058613	-347.7984251	-347.8939579	-347.8575669	-347.9568845
MN15	-347.6055707	-347.4998749	-347.5970529	-347.5585384	-347.6569291
M08HX	-347.9489655	-347.8363286	-347.9381799	-347.8981795	-347.9987639
M06	-347.7791339	-347.6714987	-347.7652810	-347.7273314	-347.8257273
M06-D3	-347.7809290	-347.6733510	-347.7670357	-347.7291563	-347.8275220
M06HF	-347.9701048	-347.8609248	-347.9646112	-347.9265474	-348.0212747
M06HF-D3	-347.9711160	-347.8619914	-347.9656411	-347.9275394	-348.0222608
M062X	-347.8827011	-347.7733139	-347.8728635	-347.8328328	-347.9303845
M062X-D3	-347.8831805	-347.7738203	-347.8733831	-347.8332683	-347.9308263
M05	-347.7667510	-347.6594831	-347.7503989	-347.7089504	-347.8128404
M05-D3	-347.7718799	-347.6643945	-347.7552551	-347.7143591	-347.8183007

Continued on next page

Table S18 (continued)

XC	1	TS_{enol}	2	TS_{conia}	3
M052X	-347.9945897	-347.8852179	-347.9854193	-347.9492143	-348.0469932
M052X-D3	-347.9957439	-347.8864317	-347.9865830	-347.9503578	-348.0481236
TPSSH	-348.0519608	-347.9460932	-348.0386270	-348.0083922	-348.1028332
CAM-B3LYP	-347.8502629	-347.7393680	-347.8382887	-347.7963542	-347.8988621
CAM-B3LYP-D3BJ	-347.8622543	-347.7514293	-347.8502650	-347.8102621	-347.9123113
wB97XD	-347.9202339	-347.8096078	-347.9068088	-347.8670435	-347.9721162
B2PLYP	-347.7424955	-347.6329464	-347.7296411	-347.6919690	-347.7885023
mPW2PLYP	-347.7401726	-347.6291875	-347.7274655	-347.6886153	-347.7871930
DSDPBEP86	-347.3961340	-347.2861921	-347.3827914	-347.3475088	-347.4472432

Table S19: XC/aug-cc-pVTZ single-point energies on CCSD/6-31G(d,p) optimized geometries for the reaction profile of **hex-5-ynal** (1a) + $\mathbf{H_2O}$.

XC	1	TS_{enol}	2	TS_{conia}	3
CCSD(T)	-384.4419462	-384.3711400	-384.4296563	-384.3639606	-384.4542281
PBE	-384.7012270	-384.6523531	-384.6927145	-384.6435847	-384.7220590
PBE-D3BJ	-384.7175195	-384.6675978	-384.7079696	-384.6601607	-384.7376648
PW91	-385.0302524	-384.9814513	-385.0218489	-384.9723193	-385.0502843
M06L	-385.1168425	-385.0471191	-385.1003225	-385.0387338	-385.1303111
M06L-D3	-385.1174653	-385.0477306	-385.1010106	-385.0393023	-385.1308987
MN12-L	-384.7699834	-384.7016187	-384.7619167	-384.6954470	-384.7820819
MN15-L	-384.8108381	-384.7348296	-384.7967370	-384.7339076	-384.8272782
TPSS	-385.2308012	-385.1750599	-385.2198788	-385.1677366	-385.2503513
TPSS-D3BJ	-385.2512163	-385.1939947	-385.2389327	-385.1884186	-385.2698453
B3LYP	-385.1896748	-385.1274056	-385.1801085	-385.1078277	-385.2035588
B3LYP-D3BJ	-385.2165034	-385.1526611	-385.2052548	-385.1423197	-385.2294860
PBE0	-384.7244106	-384.6671510	-384.7154273	-384.6513128	-384.7462220
PBE0-D3BJ	-384.7386082	-384.6802200	-384.7286812	-384.6718955	-384.7597154
B3PW91	-385.0288223	-384.9721038	-385.0200909	-384.9555429	-385.0489713
B3PW91-D3	-385.0470529	-384.9863086	-385.0356821	-384.9776988	-385.0647529
X3LYP	-385.0144800	-384.9520238	-385.0049138	-384.9322904	-385.0284240
B1LYP	-384.9907673	-384.9255946	-384.9807752	-384.9056874	-385.0039637
mPW1PW91	-385.0764350	-385.0184248	-385.0674554	-385.0020337	-385.0974174
B972	-385.0374347	-384.9763807	-385.0278586	-384.9659508	-385.0570273
MN15	-384.7306682	-384.6646320	-384.7221498	-384.6565725	-384.7446690
M08HX	-385.0788100	-385.0072938	-385.0685847	-384.9995722	-385.0894228
M06	-384.9212929	-384.8545934	-384.9075289	-384.8432347	-384.9317767
M06-D3	-384.9235543	-384.8563060	-384.9096305	-384.8452144	-384.9337263

Continued on next page

Table S19 (continued)

XC	1	TS_{enol}	2	TS_{conia}	3
M06HF	-385.1051539	-385.0455545	-385.1013632	-385.0336856	-385.1152522
M06HF-D3	-385.1063425	-385.0465674	-385.1025969	-385.0347605	-385.1163424
M062X	-385.0203705	-384.9546102	-385.0107835	-384.9426680	-385.0299976
M062X-D3	-385.0208937	-384.9551421	-385.0113656	-384.9431464	-385.0304927
M05	-384.9102421	-384.8425601	-384.8950573	-384.8302075	-384.9273155
M05-D3	-384.9164052	-384.8474742	-384.9005570	-384.8357155	-384.9327292
M052X	-385.1378121	-385.0750450	-385.1293824	-385.0635992	-385.1501258
M052X-D3	-385.1391911	-385.0761835	-385.1307812	-385.0648400	-385.1513744
TPSSH	-385.1882363	-385.1295858	-385.1773402	-385.1218124	-385.2082410
CAM-B3LYP	-384.9957432	-384.9319888	-384.9855173	-384.9169388	-385.0105896
CAM-B3LYP-D3BJ	-385.0084562	-384.9435091	-384.9973833	-384.9295902	-385.0225834
wB97XD	-385.0570809	-384.9886292	-385.0443861	-384.9781738	-385.0736522
B2PLYP	-384.8784923	-384.8131687	-384.8672345	-384.7939110	-384.8900094
mPW2PLYP	-384.8752162	-384.8086654	-384.8639332	-384.7900206	-384.8870680
DSDPBEP86	-384.5075241	-384.4404717	-384.4950550	-384.4234686	-384.5207216

Table S20: XC/aug-cc-pVTZ single-point energies on CCSD/6-31G(d,p) optimized geometries for the reaction profile of **hept-6-ynal** (1b) + $\mathbf{H_2O}$.

XC	1	TS_{enol}	2	TS_{conia}	3
CCSD(T)	-423.6824188	-423.6129791	-423.6720732	-423.6289914	-423.7321977
PBE	-423.9696016	-423.9197455	-423.9624843	-423.9337872	-424.0223605
PBE-D3BJ	-423.9878924	-423.9393116	-423.9812454	-423.9548573	-424.0424454
PW91	-424.3360970	-424.2864331	-424.3291714	-424.3003459	-424.3883778
M06L	-424.4337123	-424.3672453	-424.4210041	-424.3807150	-424.4803204
M06L-D3	-424.4345704	-424.3681458	-424.4219284	-424.3815246	-424.4811177
MN12-L	-424.0401874	-423.9768431	-424.0367311	-423.9935085	-424.0916293
MN15-L	-424.0913310	-424.0210400	-424.0819088	-424.0411111	-424.1456987
TPSS	-424.5629997	-424.5052875	-424.5525428	-424.5206797	-424.6127273
TPSS-D3BJ	-424.5857887	-424.5296668	-424.5759653	-424.5470155	-424.6378182
B3LYP	-424.5168635	-424.4521822	-424.5075845	-424.4640421	-424.5624748
B3LYP-D3BJ	-424.5469049	-424.4843378	-424.5383792	-424.4990023	-424.5957847
PBE0	-424.0005663	-423.9424720	-423.9928481	-423.9565077	-424.0568115
PBE0-D3BJ	-424.0164086	-423.9594209	-424.0091784	-423.9747575	-424.0741977
B3PW91	-424.3409017	-424.2819279	-424.3325242	-424.2956221	-424.3938513
B3PW91-D3	-424.3604446	-424.3016960	-424.3518569	-424.3166730	-424.4144082
X3LYP	-424.3196788	-424.2552655	-424.3107005	-424.2670275	-424.3659387
B1LYP	-424.2902443	-424.2225276	-424.2803877	-424.2342365	-424.3352726

Continued on next page

Table S20 (continued)

XC	1	TS_{enol}	2	TS_{conia}	3
mPW1PW91	-424.3932295	-424.3336487	-424.3849994	-424.3476358	-424.4480882
B972	-424.3489572	-424.2855831	-424.3395776	-424.2990551	-424.4005128
MN15	-424.0008057	-423.9381245	-423.9956941	-423.9520714	-424.0517861
M08HX	-424.3951947	-424.3271744	-424.3883792	-424.3428095	-424.4447392
M06	-424.2121316	-424.1475698	-424.2016993	-424.1583071	-424.2584681
M06-D3	-424.2146839	-424.1502824	-424.2043881	-424.1609632	-424.2609871
M06HF	-424.4217827	-424.3637567	-424.4195012	-424.3765026	-424.4718087
M06HF-D3	-424.4232775	-424.3653474	-424.4210987	-424.3779738	-424.4732412
M062X	-424.3240948	-424.2613686	-424.3176083	-424.2724909	-424.3711750
M062X-D3	-424.3248319	-424.2621364	-424.3184014	-424.2731797	-424.3718519
M05	-424.1997019	-424.1311947	-424.1859267	-424.1391588	-424.2460397
M05-D3	-424.2065446	-424.1381815	-424.1927722	-424.1463911	-424.2530839
M052X	-424.4571087	-424.3960517	-424.4508555	-424.4097863	-424.5090548
M052X-D3	-424.4588009	-424.3978543	-424.4526584	-424.4114727	-424.5106854
TPSSH	-424.5172496	-424.4567735	-424.5068409	-424.4720052	-424.5685472
CAM-B3LYP	-424.2973994	-424.2322887	-424.2880351	-424.2415143	-424.3462709
CAM-B3LYP-D3BJ	-424.3115023	-424.2473943	-424.3026814	-424.2577688	-424.3617427
wB97XD	-424.3703047	-424.3035853	-424.3603853	-424.3159938	-424.4223975
B2PLYP	-424.1659850	-424.0998706	-424.1556812	-424.1136843	-424.2121225
mPW2PLYP	-424.1628593	-424.0959309	-424.1528190	-424.1093941	-424.2099618
DSDPBEP86	-423.7552488	-423.6898077	-423.7451448	-423.7050664	-423.8062732

4 SMD energies

Table S21: XC-SMD(H_2O)/aug-cc-pVTZ single-point energies on CCSD-SMD(H_2O)/6-31G(d,p) optimized geometries for the reaction profile of **hex-5-ynal** (1a).

XC	1	TS_{enol}	2	TS_{conia}	3
CCSD(T)	-308.0957246	-307.9829230	-308.0834588	-308.0177484	-308.1058897
PBE	-308.3211172	-308.2235742	-308.3108353	-308.2619232	-308.3380478
PBE-D3BJ	-308.3340844	-308.2360813	-308.3229469	-308.2759264	-308.3511187
PW91	-308.5913060	-308.4931000	-308.5810087	-308.5318725	-308.6074910
M06L	-308.6682061	-308.5566522	-308.6509359	-308.5902988	-308.6800549
M06L-D3	-308.6685738	-308.5570458	-308.6513451	-308.5906089	-308.6804000
MN12-L	-308.3874606	-308.2769396	-308.3788161	-308.3138474	-308.3989898
MN15-L	-308.4286321	-308.3166977	-308.4148399	-308.3534446	-308.4443643
TPSS	-308.7660735	-308.6621456	-308.7533027	-308.7012167	-308.7817452

Continued on next page

Table S21 (continued)

XC	1	TS_{enol}	2	TS_{conia}	3
TPSS-D3BJ	-308.7823025	-308.6777715	-308.7684236	-308.7186864	-308.7980698
B3LYP	-308.7261547	-308.6162173	-308.7148392	-308.6492105	-308.7360968
B3LYP-D3BJ	-308.7478317	-308.6371199	-308.7351061	-308.6728933	-308.7580907
PBE0	-308.3454825	-308.2390444	-308.3345980	-308.2767968	-308.3637645
PBE0-D3BJ	-308.3566813	-308.2498299	-308.3450376	-308.2887604	-308.3749892
B3PW91	-308.5967470	-308.4905409	-308.5860687	-308.5271312	-308.6130187
B3PW91-D3	-308.6086129	-308.5011350	-308.5960030	-308.5385693	-308.6242593
X3LYP	-308.5795667	-308.4693177	-308.5682334	-308.5026017	-308.5896855
B1LYP	-308.5591588	-308.4468828	-308.5474485	-308.4789639	-308.5684407
mPW1PW91	-308.6363389	-308.5288045	-308.6253694	-308.5660944	-308.6536543
B972	-308.6039733	-308.4955265	-308.5925647	-308.5300661	-308.6196703
MN15	-308.3430410	-308.2355474	-308.3336659	-308.2694668	-308.3559124
M08HX	-308.6406490	-308.5262627	-308.6293278	-308.5616276	-308.6499474
M06	-308.4955816	-308.3861094	-308.4810298	-308.4169549	-308.5041758
M06-D3	-308.4969378	-308.3873705	-308.4822479	-308.4181345	-308.5054642
M06HF	-308.6651659	-308.5549412	-308.6597553	-308.5927378	-308.6732003
M06HF-D3	-308.6658815	-308.5556524	-308.6604624	-308.5933567	-308.6738916
M062X	-308.5874937	-308.4764908	-308.5771184	-308.5096521	-308.5957617
M062X-D3	-308.5877988	-308.4768223	-308.5774693	-308.5099076	-308.5960448
M05	-308.4865615	-308.3778967	-308.4697836	-308.4043115	-308.5001726
M05-D3	-308.4905635	-308.3815100	-308.4732272	-308.4081561	-308.5039942
M052X	-308.6857851	-308.5746413	-308.6757187	-308.6105537	-308.6956325
M052X-D3	-308.6866152	-308.5754517	-308.6765193	-308.6112742	-308.6964343
TPSSH	-308.7322259	-308.6252684	-308.7194445	-308.6638978	-308.7484932
CAM-B3LYP	-308.5586249	-308.4466118	-308.5466866	-308.4782131	-308.5699400
CAM-B3LYP-D3BJ	-308.5685880	-308.4562024	-308.5559812	-308.4887299	-308.5798598
wB97XD	-308.6154366	-308.5033587	-308.6016987	-308.5355849	-308.6298947
B2PLYP	-308.4639474	-308.3532129	-308.4515890	-308.3868555	-308.4723092
mPW2PLYP	-308.4616314	-308.3493783	-308.4491384	-308.3830949	-308.4704202
DSDPBEP86	-308.1563475	-308.0448945	-308.1432304	-308.0803089	-308.1672882

Table S22: XC-SMD(H_2O)/aug-cc-pVTZ single-point energies on CCSD-SMD(H_2O)/6-31G(d,p) optimized geometries for the reaction profile of **hept-6-ynal** (1b).

XC	1	TS_{enol}	2	TS_{conia}	3
CCSD(T)	-347.3358445	-347.2221241	-347.3234855	-347.2826867	-347.3836673
PBE	-347.5881583	-347.4899444	-347.5776779	-347.5526794	-347.6382061
PBE-D3BJ	-347.6039110	-347.5056373	-347.5932986	-347.5709453	-347.6558740

Continued on next page

Table S22 (continued)

XC	1	TS_{enol}	2	TS_{conia}	3
PW91	-347.8958053	-347.7969604	-347.8853714	-347.8604431	-347.9454397
M06L	-347.9854058	-347.8738502	-347.9689516	-347.9327252	-348.0298920
M06L-D3	-347.9859673	-347.8744507	-347.9695643	-347.9332463	-348.0304189
MN12-L	-347.6594017	-347.5488488	-347.6516202	-347.6117811	-347.7080308
MN15-L	-347.7108755	-347.5987497	-347.6979686	-347.6604542	-347.7630025
TPSS	-348.0965762	-347.9916281	-348.0832374	-348.0546865	-348.1436857
TPSS-D3BJ	-348.1162051	-348.0112058	-348.1027379	-348.0775192	-348.1657646
B3LYP	-348.0516985	-347.9404267	-348.0395282	-347.9989544	-348.0946325
B3LYP-D3BJ	-348.0778282	-347.9664413	-348.0654822	-348.0296220	-348.1242401
PBE0	-347.6206289	-347.5132759	-347.6093617	-347.5760853	-347.6740353
PBE0-D3BJ	-347.6341993	-347.5268472	-347.6228730	-347.5918084	-347.6892493
B3PW91	-347.9073670	-347.8000637	-347.8959443	-347.8615359	-347.9574065
B3PW91-D3	-347.9223459	-347.8141319	-347.9099296	-347.8775585	-347.9736945
X3LYP	-347.8832529	-347.7717187	-347.8711814	-347.8307210	-347.9268721
B1LYP	-347.8569933	-347.7432525	-347.8443286	-347.8009819	-347.8993472
mPW1PW91	-347.9519013	-347.8433348	-347.9403515	-347.9057082	-348.0038933
B972	-347.9140098	-347.8043948	-347.9017997	-347.8635956	-347.9627202
MN15	-347.6139381	-347.5060787	-347.6049951	-347.5647720	-347.6628041
M08HX	-347.9578186	-347.8433728	-347.9469685	-347.9046684	-348.0048446
M06	-347.7866581	-347.6767738	-347.7724615	-347.7322946	-347.8309772
M06-D3	-347.7884391	-347.6786574	-347.7742435	-347.7341179	-347.8327754
M06HF	-347.9817181	-347.8708299	-347.9760314	-347.9352550	-348.0297158
M06HF-D3	-347.9827227	-347.8719089	-347.9770782	-347.9362472	-348.0307024
M062X	-347.8917743	-347.7805016	-347.8817752	-347.8393742	-347.9367157
M062X-D3	-347.8922523	-347.7810114	-347.8823030	-347.8398092	-347.9371555
M05	-347.7749323	-347.6653361	-347.7578303	-347.7139177	-347.8186918
M05-D3	-347.7800708	-347.6702566	-347.7627210	-347.7193638	-347.8241644
M052X	-348.0049884	-347.8933550	-347.9950187	-347.9565743	-348.0545236
M052X-D3	-348.0061339	-347.8945837	-347.9962009	-347.9577179	-348.0556553
TPSSH	-348.0597076	-347.9516594	-348.0463132	-348.0145532	-348.1083219
CAM-B3LYP	-347.8590574	-347.7457416	-347.8464558	-347.8030856	-347.9052853
CAM-B3LYP-D3BJ	-347.8710853	-347.7578399	-347.8585139	-347.8170167	-347.9187593
wB97XD	-347.9288960	-347.8161485	-347.9152024	-347.8734810	-347.9782108
B2PLYP	-347.7502764	-347.6384376	-347.7373767	-347.6981644	-347.7941663
mPW2PLYP	-347.7482489	-347.6349106	-347.7353164	-347.6949326	-347.7930982
DSDPBEP86	-347.4037895	-347.2916726	-347.3908059	-347.3535334	-347.4527020

Table S23: XC-SMD(H_2O)/aug-cc-pVTZ single-point energies on CCSD-SMD(H_2O)/6-31G(d,p) optimized geometries for the reaction profile of **hex-5-ynal** (1a) + **H_2O**.

XC	1	TS_{enol}	2	TS_{conia}	3
CCSD(T)	-384.4567366	-384.3900176	-384.4426641	-384.3783734	-384.4646662
PBE	-384.7167709	-384.6652838	-384.7059222	-384.6574789	-384.7327731
PBE-D3BJ	-384.7329672	-384.6811908	-384.7205407	-384.6743354	-384.7483841
PW91	-385.0458255	-384.9943740	-385.0350407	-384.9863399	-385.0610595
M06L	-385.1310932	-385.0658036	-385.1118979	-385.0523396	-385.1405752
M06L-D3	-385.1317143	-385.0664678	-385.1126062	-385.0528975	-385.1411459
MN12-L	-384.7854072	-384.7251920	-384.7739264	-384.7102735	-384.7934723
MN15-L	-384.8259570	-384.7573539	-384.8081005	-384.7482830	-384.8379467
TPSS	-385.2465069	-385.1895967	-385.2335513	-385.1821599	-385.2613779
TPSS-D3BJ	-385.2667998	-385.2094112	-385.2517899	-385.2031956	-385.2808787
B3LYP	-385.2063401	-385.1443778	-385.1943055	-385.1298036	-385.2154391
B3LYP-D3BJ	-385.2330016	-385.1706261	-385.2184037	-385.1578820	-385.2413418
PBE0	-384.7410411	-384.6836530	-384.7294264	-384.6721202	-384.7580808
PBE0-D3BJ	-384.7551602	-384.6973904	-384.7421178	-384.6866405	-384.7715881
B3PW91	-385.0454674	-384.9879390	-385.0345223	-384.9762423	-385.0609529
B3PW91-D3	-385.0631230	-385.0037155	-385.0485780	-384.9926306	-385.0765892
X3LYP	-385.0311744	-384.9692504	-385.0189927	-384.9544504	-385.0402992
B1LYP	-385.0076126	-384.9434770	-384.9951084	-384.9278413	-385.0160208
mPW1PW91	-385.0931774	-385.0349514	-385.0817592	-385.0230398	-385.1094687
B972	-385.0541043	-384.9931071	-385.0421761	-384.9803958	-385.0690131
MN15	-384.7464683	-384.6840727	-384.7345333	-384.6716255	-384.7564241
M08HX	-385.0949888	-385.0308613	-385.0816573	-385.0148429	-385.1013960
M06	-384.9365960	-384.8728916	-384.9199408	-384.8566807	-384.9427889
M06-D3	-384.9389033	-384.8749603	-384.9219396	-384.8586562	-384.9448152
M06HF	-385.1244637	-385.0678909	-385.1178865	-385.0515914	-385.1298083
M06HF-D3	-385.1256724	-385.0690815	-385.1190981	-385.0526510	-385.1309002
M062X	-385.0367547	-384.9742244	-385.0242433	-384.9577825	-385.0421770
M062X-D3	-385.0372737	-384.9747874	-385.0248498	-384.9582510	-385.0426571
M05	-384.9266785	-384.8602293	-384.9083735	-384.8432488	-384.9388657
M05-D3	-384.9326835	-384.8657419	-384.9133758	-384.8488207	-384.9442233
M052X	-385.1559125	-385.0942184	-385.1439523	-385.0799316	-385.1635472
M052X-D3	-385.1573183	-385.0955725	-385.1453130	-385.0811567	-385.1648104
TPSSH	-385.2043576	-385.1454623	-385.1912779	-385.1364178	-385.2196869
CAM-B3LYP	-385.0128434	-384.9497806	-384.9999619	-384.9325387	-385.0229042
CAM-B3LYP-D3BJ	-385.0254934	-384.9619900	-385.0113279	-384.9453903	-385.0349169
wB97XD	-385.0735739	-385.0083808	-385.0580698	-384.9932333	-385.0859924

Continued on next page

Table S23 (continued)

XC	1	TS_{enol}	2	TS_{conia}	3
B2PLYP	-384.8945583	-384.8311206	-384.8810069	-384.8050348	-384.9014447
mPW2PLYP	-384.8915004	-384.8272667	-384.8776764	-384.8014068	-384.8986744
DSDPBEP86	-384.5231994	-384.4594361	-384.5082897	-384.4351163	-384.5319147

Table S24: XC-SMD(H_2O)/aug-cc-pVTZ single-point energies on CCSD-SMD(H_2O)/6-31G(d,p) optimized geometries for the reaction profile of **hept-6-ynal** (1b) + $\mathbf{H_2O}$.

XC	1	TS_{enol}	2	TS_{conia}	3
CCSD(T)	-423.6954887	-423.6296614	-423.6857359	-423.6432820	-423.7437923
PBE	-423.9830046	-423.9318333	-423.9759393	-423.9482304	-424.0340192
PBE-D3BJ	-424.0015031	-423.9514099	-423.9944336	-423.9693362	-424.0542037
PW91	-424.3495244	-424.2984351	-424.3425953	-424.3148846	-424.4000996
M06L	-424.4466324	-424.3837148	-424.4330391	-424.3950357	-424.4916893
M06L-D3	-424.4474822	-424.3846308	-424.4339378	-424.3958374	-424.4924736
MN12-L	-424.0546610	-423.9976861	-424.0499282	-424.0084231	-424.1043592
MN15-L	-424.1052302	-424.0406616	-424.0948056	-424.0557073	-424.1579630
TPSS	-424.5766929	-424.5189895	-424.5663001	-424.5356014	-424.6246200
TPSS-D3BJ	-424.5997406	-424.5433951	-424.5893847	-424.5619812	-424.6498330
B3LYP	-424.5312998	-424.4684306	-424.5218057	-424.4795277	-424.5751924
B3LYP-D3BJ	-424.5616409	-424.5005797	-424.5521626	-424.5145416	-424.6086462
PBE0	-424.0152707	-423.9578899	-424.0071260	-423.9713847	-424.0696406
PBE0-D3BJ	-424.0312965	-423.9748729	-424.0232181	-423.9896617	-424.0871146
B3PW91	-424.3556009	-424.2971707	-424.3469350	-424.3106090	-424.4066428
B3PW91-D3	-424.3754219	-424.3172121	-424.3657305	-424.3316894	-424.4272498
X3LYP	-424.3341424	-424.2715972	-424.3248861	-424.2825474	-424.3787069
B1LYP	-424.3048686	-424.2396565	-424.2947505	-424.2498422	-424.3481658
mPW1PW91	-424.4080621	-424.3493043	-424.3994195	-424.3626140	-424.4610209
B972	-424.3636305	-424.3016762	-424.3539738	-424.3139466	-424.4132743
MN15	-423.9970316	-423.9304378	-423.9928994	-423.9509261	-424.0500716
M08HX	-424.3907218	-424.3218852	-424.3850358	-424.3412248	-424.4423952
M06	-424.2084319	-424.1404954	-424.1988676	-424.1568406	-424.2565424
M06-D3	-424.2284746	-424.1670421	-424.2169139	-424.1747844	-424.2732296
M06HF	-424.4173036	-424.3544375	-424.4161423	-424.3742628	-424.4693771
M06HF-D3	-424.4412372	-424.3851038	-424.4381967	-424.3953841	-424.4890294
M062X	-424.3199759	-424.2527683	-424.3146000	-424.2708048	-424.3690778
M062X-D3	-424.3399362	-424.2792825	-424.3326382	-424.2882621	-424.3851985
M05	-424.1955799	-424.1238089	-424.1836153	-424.1374067	-424.2438003
M05-D3	-424.2208746	-424.1549677	-424.2061827	-424.1604179	-424.2656389

Continued on next page

Table S24 (continued)

XC	1	TS_{enol}	2	TS_{conia}	3
M052X	-424.4529171	-424.3864060	-424.4479853	-424.4082646	-424.5069521
M052X-D3	-424.4752920	-424.4148747	-424.4679615	-424.4275708	-424.5252555
TPSSH	-424.5135779	-424.4479494	-424.5051417	-424.4718070	-424.5667331
CAM-B3LYP	-424.3123914	-424.2489671	-424.3026512	-424.2573565	-424.3595643
CAM-B3LYP-D3BJ	-424.3266505	-424.2641267	-424.3170777	-424.2736279	-424.3751120
wB97XD	-424.3857595	-424.3214627	-424.3746942	-424.3312588	-424.4356051
B2PLYP	-424.1799868	-424.1163867	-424.1697364	-424.1287955	-424.2246001
mPW2PLYP	-424.1770812	-424.1129240	-424.1669257	-424.1246366	-424.2226540
DSDPBEP86	-423.7692197	-423.7066026	-423.7590601	-423.7198276	-423.8186425

5 Basis set effect

5.1 6-31G

Table S25: XC/6-31G single-point energies on CCSD/6-31G(d,p) optimized geometries for the reaction profile of **hex-5-ynal** (1a).

XC	1	TS_{enol}	2	TS_{conia}	3
CCSD(T)	-308.087448	-307.978429	-308.074749	-308.011221	-308.101400
PBE	-308.117604	-308.013115	-308.101390	-308.058742	-308.139484
PBE-D3BJ	-308.130493	-308.025688	-308.113474	-308.072620	-308.152524
PW91	-308.384084	-308.279299	-308.367939	-308.325350	-308.405461
M06L	-308.467615	-308.351175	-308.449882	-308.393523	-308.484637
M06L-D3	-308.467985	-308.351564	-308.450291	-308.393833	-308.484984
MN12-L	-308.190768	-308.071928	-308.178063	-308.117456	-308.206566
MN15-L	-308.192317	-308.071547	-308.174739	-308.117290	-308.210994
TPSS	-308.561101	-308.451941	-308.544329	-308.497458	-308.581429
TPSS-D3BJ	-308.577228	-308.467653	-308.559413	-308.514771	-308.597716
B3LYP	-308.508092	-308.392390	-308.492874	-308.433523	-308.523536
B3LYP-D3BJ	-308.529627	-308.413413	-308.513092	-308.456988	-308.545474
PBE0	-308.137326	-308.023433	-308.122626	-308.069962	-308.160448
PBE0-D3BJ	-308.148457	-308.034274	-308.133041	-308.081820	-308.171649
B3PW91	-308.388677	-308.275571	-308.374119	-308.320317	-308.409869
B3PW91-D3	-308.400459	-308.286281	-308.384008	-308.331654	-308.421084
X3LYP	-308.360116	-308.244055	-308.344924	-308.285702	-308.375814
B1LYP	-308.339370	-308.221336	-308.324164	-308.261835	-308.354128
mPW1PW91	-308.426721	-308.312070	-308.412196	-308.357971	-308.448939

Continued on next page

Table S25 (continued)

XC	1	TS_{enol}	2	TS_{conia}	3
B972	-308.396368	-308.280959	-308.381662	-308.323928	-308.416632
MN15	-308.119705	-308.005629	-308.106057	-308.048020	-308.138493
M08HX	-308.411297	-308.288630	-308.396575	-308.334076	-308.426874
M06	-308.292064	-308.177382	-308.276113	-308.219211	-308.308386
M06-D3	-308.293422	-308.178638	-308.277330	-308.220390	-308.309676
M06HF	-308.393418	-308.274372	-308.386264	-308.321123	-308.407325
M06HF-D3	-308.394138	-308.275078	-308.386970	-308.321742	-308.408019
M062X	-308.367803	-308.248813	-308.354651	-308.292151	-308.382412
M062X-D3	-308.368111	-308.249141	-308.355001	-308.292406	-308.382697
M05	-308.295851	-308.180979	-308.278999	-308.219710	-308.316385
M05-D3	-308.299836	-308.184628	-308.282427	-308.223525	-308.320201
M052X	-308.455128	-308.337699	-308.443719	-308.382808	-308.471326
M052X-D3	-308.455962	-308.338504	-308.444518	-308.383529	-308.472130
TPSSH	-308.525107	-308.412546	-308.508974	-308.458338	-308.546004
CAM-B3LYP	-308.335078	-308.217178	-308.319782	-308.257770	-308.352558
CAM-B3LYP-D3BJ	-308.344983	-308.226815	-308.329053	-308.268196	-308.362459
wB97XD	-308.405719	-308.286888	-308.388912	-308.327866	-308.425405
B2PLYP	-308.061105	-307.941190	-308.044253	-307.978850	-308.070984
mPW2PLYP	-308.070758	-307.949558	-308.054207	-307.988044	-308.081406
DSDPBEP86	-307.649283	-307.527139	-307.631966	-307.565088	-307.660045

Table S26: XC/6-31G single-point energies on CCSD/6-31G(d,p) optimized geometries for the reaction profile of **hept-6-ynal** (1b).

XC	1	TS_{enol}	2	TS_{conia}	3
CCSD(T)	-347.328943	-347.217226	-347.315776	-347.276947	-347.378884
PBE	-347.363722	-347.257653	-347.347773	-347.330931	-347.419766
PBE-D3BJ	-347.379420	-347.273332	-347.363322	-347.349166	-347.437395
PW91	-347.667167	-347.560826	-347.651344	-347.634804	-347.722996
M06L	-347.763232	-347.645650	-347.746405	-347.716706	-347.815299
M06L-D3	-347.763795	-347.646245	-347.747008	-347.717228	-347.815828
MN12-L	-347.442077	-347.321495	-347.430542	-347.397203	-347.496365
MN15-L	-347.448344	-347.326031	-347.432126	-347.401285	-347.505912
TPSS	-347.870257	-347.759148	-347.853338	-347.831883	-347.923052
TPSS-D3BJ	-347.889817	-347.778701	-347.872740	-347.854675	-347.945083
B3LYP	-347.810532	-347.692758	-347.795091	-347.762865	-347.860166
B3LYP-D3BJ	-347.836566	-347.718768	-347.820933	-347.793483	-347.889705
PBE0	-347.391083	-347.275146	-347.376452	-347.350727	-347.450634

Continued on next page

Table S26 (continued)

XC	1	TS_{enol}	2	TS_{conia}	3
PBE0-D3BJ	-347.404608	-347.288691	-347.389888	-347.366423	-347.465817
B3PW91	-347.677743	-347.562525	-347.662887	-347.635848	-347.733847
B3PW91-D3	-347.692637	-347.576542	-347.676768	-347.651752	-347.750106
X3LYP	-347.640568	-347.522458	-347.625281	-347.593334	-347.691001
B1LYP	-347.613843	-347.493628	-347.598352	-347.563335	-347.662966
mPW1PW91	-347.720617	-347.603847	-347.705966	-347.678759	-347.778794
B972	-347.684905	-347.567329	-347.669849	-347.638854	-347.739728
MN15	-347.366990	-347.251067	-347.354235	-347.321996	-347.421918
M08HX	-347.705248	-347.580902	-347.691389	-347.656645	-347.760156
M06	-347.562237	-347.445956	-347.546989	-347.516498	-347.615806
M06-D3	-347.564033	-347.447808	-347.548744	-347.518322	-347.617601
M06HF	-347.679904	-347.558572	-347.672992	-347.637517	-347.734958
M06HF-D3	-347.680915	-347.559639	-347.674022	-347.638509	-347.735944
M062X	-347.649700	-347.528938	-347.637256	-347.602910	-347.703149
M062X-D3	-347.650180	-347.529445	-347.637776	-347.603346	-347.703591
M05	-347.566178	-347.449437	-347.549449	-347.514843	-347.619692
M05-D3	-347.571307	-347.454348	-347.554306	-347.520252	-347.625153
M052X	-347.749685	-347.630265	-347.738789	-347.707144	-347.806704
M052X-D3	-347.750839	-347.631479	-347.739953	-347.708288	-347.807834
TPSSH	-347.831014	-347.716355	-347.814697	-347.789950	-347.885416
CAM-B3LYP	-347.611962	-347.491885	-347.596631	-347.562043	-347.665621
CAM-B3LYP-D3BJ	-347.623953	-347.503947	-347.608607	-347.575950	-347.679070
wB97XD	-347.697524	-347.576679	-347.681198	-347.647174	-347.753697
B2PLYP	-347.295999	-347.174020	-347.278909	-347.242047	-347.342351
mPW2PLYP	-347.307882	-347.184602	-347.291229	-347.253773	-347.355633
DSDPBEP86	-346.829203	-346.705179	-346.812132	-346.774498	-346.878828

Table S27: XC/6-31G single-point energies on CCSD/6-31G(d,p) optimized geometries for the reaction profile of **hex-5-ynal** (1a) + H_2O.

XC	1	TS_{enol}	2	TS_{conia}	3
CCSD(T)	-384.4419462	-384.3711400	-384.4296563	-384.3639606	-384.4542281
PBE	-384.434949	-384.388347	-384.421247	-384.373993	-384.455190
PBE-D3BJ	-384.451241	-384.403592	-384.436502	-384.390569	-384.470796
PW91	-384.759670	-384.713159	-384.746009	-384.698751	-384.779322
M06L	-384.863077	-384.797544	-384.846570	-384.786157	-384.877850
M06L-D3	-384.863700	-384.798156	-384.847258	-384.786725	-384.878438
MN12-L	-384.521150	-384.450449	-384.509849	-384.443961	-384.533311

Continued on next page

Table S27 (continued)

XC	1	TS_{enol}	2	TS_{conia}	3
MN15-L	-384.513610	-384.437273	-384.496171	-384.433469	-384.528113
TPSS	-384.965769	-384.912557	-384.951324	-384.900922	-384.984788
TPSS-D3BJ	-384.986184	-384.931492	-384.970378	-384.921604	-385.004282
B3LYP	-384.909954	-384.850104	-384.896559	-384.834115	-384.924087
B3LYP-D3BJ	-384.936782	-384.875359	-384.921705	-384.861726	-384.950014
PBE0	-384.456117	-384.400044	-384.443675	-384.386931	-384.477696
PBE0-D3BJ	-384.470315	-384.413113	-384.456929	-384.401214	-384.491189
B3PW91	-384.760443	-384.705353	-384.748321	-384.691152	-384.780607
B3PW91-D3	-384.778674	-384.719557	-384.763912	-384.707186	-384.796388
X3LYP	-384.733270	-384.673152	-384.719837	-384.657289	-384.747467
B1LYP	-384.709774	-384.646862	-384.696228	-384.631022	-384.723215
mPW1PW91	-384.806751	-384.749976	-384.794523	-384.736649	-384.827692
B972	-384.769977	-384.710282	-384.757289	-384.696454	-384.789205
MN15	-384.442598	-384.379568	-384.430777	-384.367462	-384.458288
M08HX	-384.781938	-384.712300	-384.769218	-384.701402	-384.794280
M06	-384.663187	-384.600101	-384.648387	-384.587062	-384.677233
M06-D3	-384.665448	-384.601813	-384.650489	-384.589042	-384.679183
M06HF	-384.757801	-384.698688	-384.752764	-384.683553	-384.768700
M06HF-D3	-384.758990	-384.699701	-384.753997	-384.684628	-384.769790
M062X	-384.737261	-384.674174	-384.725820	-384.658661	-384.748795
M062X-D3	-384.737784	-384.674706	-384.726402	-384.659139	-384.749290
M05	-384.665574	-384.601293	-384.649586	-384.586013	-384.684436
M05-D3	-384.671737	-384.606208	-384.655086	-384.591521	-384.689850
M052X	-384.843822	-384.784148	-384.834194	-384.768842	-384.857604
M052X-D3	-384.845201	-384.785286	-384.835593	-384.770083	-384.858852
TPSSH	-384.921791	-384.865231	-384.907894	-384.853831	-384.941380
CAM-B3LYP	-384.709873	-384.648795	-384.696338	-384.630798	-384.725748
CAM-B3LYP-D3BJ	-384.722586	-384.660315	-384.708204	-384.643450	-384.737742
wB97XD	-384.787762	-384.721710	-384.772520	-384.707600	-384.804899
B2PLYP	-384.370277	-384.302173	-384.354897	-384.287108	-384.378944
mPW2PLYP	-384.382580	-384.313445	-384.367383	-384.298582	-384.391807
DSDPBEP86	-383.870522	-383.798608	-383.854648	-383.785163	-383.879627

Table S28: XC/6-31G single-point energies on CCSD/6-31G(d,p) optimized geometries for the reaction profile of **hept-6-ynal** (1b) + $\mathbf{H_2O}$.

XC	1	TS_{enol}	2	TS_{conia}	3
CCSD(T)	-423.682419	-423.612979	-423.672073	-423.628991	-423.732198

Continued on next page

Table S28 (continued)

XC	1	TS_{enol}	2	TS_{conia}	3
PBE	-423.678591	-423.634954	-423.670070	-423.645433	-423.736062
PBE-D3BJ	-423.696882	-423.654520	-423.688832	-423.666503	-423.756147
PW91	-424.040238	-423.996977	-424.031898	-424.007443	-424.097422
M06L	-424.156537	-424.095923	-424.145306	-424.108850	-424.208715
M06L-D3	-424.157395	-424.096823	-424.146230	-424.109660	-424.209512
MN12-L	-423.769238	-423.705224	-423.763504	-423.723401	-423.823450
MN15-L	-423.765509	-423.697328	-423.754760	-423.717538	-423.822880
TPSS	-424.273220	-424.221711	-424.262632	-424.234495	-424.327197
TPSS-D3BJ	-424.296009	-424.246090	-424.286055	-424.260831	-424.352287
B3LYP	-424.210672	-424.152274	-424.201119	-424.162581	-424.261503
B3LYP-D3BJ	-424.240713	-424.184430	-424.231914	-424.197541	-424.294813
PBE0	-423.707854	-423.654261	-423.699679	-423.666831	-423.768513
PBE0-D3BJ	-423.723696	-423.671210	-423.716010	-423.685081	-423.785899
B3PW91	-424.048080	-423.993887	-424.039178	-424.005759	-424.105403
B3PW91-D3	-424.067623	-424.013655	-424.058511	-424.026810	-424.125960
X3LYP	-424.011800	-423.953692	-424.002587	-423.964057	-424.063383
B1LYP	-423.982588	-423.921029	-423.972711	-423.931638	-424.032842
mPW1PW91	-424.098994	-424.043923	-424.090389	-424.056524	-424.158257
B972	-424.057065	-423.998362	-424.047502	-424.010534	-424.113020
MN15	-423.686875	-423.629351	-423.680696	-423.640966	-423.742195
M08HX	-424.072849	-424.009434	-424.065507	-424.023386	-424.127941
M06	-423.931032	-423.872570	-423.921370	-423.883745	-423.984723
M06-D3	-423.933584	-423.875282	-423.924059	-423.886401	-423.987242
M06HF	-424.041828	-423.987744	-424.040995	-423.998885	-424.096542
M06HF-D3	-424.043323	-423.989335	-424.042593	-424.000356	-424.097975
M062X	-424.016458	-423.958858	-424.010111	-423.968747	-424.069831
M062X-D3	-424.017195	-423.959626	-424.010904	-423.969436	-424.070508
M05	-423.933757	-423.872445	-423.922411	-423.880588	-423.988117
M05-D3	-423.940599	-423.879431	-423.929256	-423.887821	-423.995161
M052X	-424.135939	-424.080755	-424.131021	-424.092347	-424.193187
M052X-D3	-424.137632	-424.082557	-424.132824	-424.094033	-424.194818
TPSSH	-424.226090	-424.171163	-424.215815	-424.184558	-424.281574
CAM-B3LYP	-423.984877	-423.926126	-423.975582	-423.934123	-424.039497
CAM-B3LYP-D3BJ	-423.998980	-423.941232	-423.990228	-423.950378	-424.054969
wB97XD	-424.076930	-424.015318	-424.066793	-424.026353	-424.133848
B2PLYP	-423.603649	-423.537390	-423.591725	-423.549396	-423.650900
mPW2PLYP	-423.617985	-423.551291	-423.606630	-423.563413	-423.666563
DSDPBEP86	-423.048493	-422.980086	-423.036804	-422.993713	-423.098808

5.2 6-31G(d,p)

Table S29: XC/6-31G(d,p) single-point energies on CCSD/6-31G(d,p) optimized geometries for the reaction profile of **hex-5-ynal** (1a).

XC	1	TS_{enol}	2	TS_{conia}	3
CCSD(T)	-308.0874483	-307.9784291	-308.0747491	-308.0112212	-308.1013997
PBE	-308.2034016	-308.1080974	-308.1887017	-308.1487089	-308.2292438
PBE-D3BJ	-308.2162903	-308.1206705	-308.2007856	-308.1625868	-308.2422836
PW91	-308.4696077	-308.3738700	-308.4549773	-308.4150188	-308.4949044
M06L	-308.5604858	-308.4520187	-308.5415426	-308.4878304	-308.5807429
M06L-D3	-308.5608566	-308.4524086	-308.5419513	-308.4881404	-308.5810899
MN12-L	-308.2884768	-308.1802270	-308.2772995	-308.2184696	-308.3077147
MN15-L	-308.3048768	-308.1939560	-308.2877041	-308.2317511	-308.3273064
TPSS	-308.6480655	-308.5467677	-308.6314423	-308.5874565	-308.6725200
TPSS-D3BJ	-308.6641929	-308.5624791	-308.6465265	-308.6047693	-308.6888068
B3LYP	-308.6037021	-308.4965118	-308.5886256	-308.5320994	-308.6230739
B3LYP-D3BJ	-308.6252374	-308.5175352	-308.6088433	-308.5555643	-308.6450119
PBE0	-308.2333467	-308.1289477	-308.2188245	-308.1689648	-308.2602986
PBE0-D3BJ	-308.2444780	-308.1397887	-308.2292387	-308.1808230	-308.2714992
B3PW91	-308.4823276	-308.3784181	-308.4680906	-308.4171691	-308.5073686
B3PW91-D3	-308.4941086	-308.3891283	-308.4779787	-308.4285056	-308.5185838
X3LYP	-308.4566528	-308.3491422	-308.4415374	-308.3851192	-308.4762753
B1LYP	-308.4371028	-308.3275207	-308.4217175	-308.3622318	-308.4558044
mPW1PW91	-308.5222524	-308.4169273	-308.5078280	-308.4564557	-308.5482898
B972	-308.4917433	-308.3854446	-308.4770363	-308.4221521	-308.5158123
MN15	-308.2245513	-308.1202460	-308.2116324	-308.1554767	-308.2464006
M08HX	-308.5039492	-308.3918275	-308.4892210	-308.4292005	-308.5223446
M06	-308.3838865	-308.2778664	-308.3667443	-308.3121894	-308.4022354
M06-D3	-308.3852444	-308.2791223	-308.3679614	-308.3133683	-308.4035253
M06HF	-308.4958490	-308.3885069	-308.4878981	-308.4272610	-308.5131319
M06HF-D3	-308.4965690	-308.3892130	-308.4886038	-308.4278800	-308.5138256
M062X	-308.4590766	-308.3503212	-308.4451593	-308.3857240	-308.4763593
M062X-D3	-308.4593848	-308.3506495	-308.4455101	-308.3859796	-308.4766441
M05	-308.3858222	-308.2794675	-308.3665517	-308.3107562	-308.4088231
M05-D3	-308.3898075	-308.2831168	-308.3699797	-308.3145711	-308.4126384
M052X	-308.5547428	-308.4466510	-308.5420658	-308.4844601	-308.5742926
M052X-D3	-308.5555775	-308.4474562	-308.5428649	-308.4851806	-308.5750967
TPSSH	-308.6163819	-308.5119332	-308.6000027	-308.5522630	-308.6413351
CAM-B3LYP	-308.4341362	-308.3250909	-308.4185882	-308.3593895	-308.4552790
CAM-B3LYP-D3BJ	-308.4440410	-308.3347279	-308.4278589	-308.3698152	-308.4651805

Continued on next page

Table S29 (continued)

XC	1	TS_{enol}	2	TS_{conia}	3
wB97XD	-308.5007632	-308.3915202	-308.4839884	-308.4261042	-308.5243040
B2PLYP	-308.2750645	-308.1649870	-308.2580972	-308.1991329	-308.2917511
mPW2PLYP	-308.2778015	-308.1663714	-308.2609079	-308.2008694	-308.2950380
DSDPBEP86	-307.9284419	-307.8165570	-307.9104385	-307.8513116	-307.9469591

Table S30: XC/6-31G(d,p) single-point energies on CCSD/6-31G(d,p) optimized geometries for the reaction profile of **hept-6-ynal** (1b).

XC	1	TS_{enol}	2	TS_{conia}	3
CCSD(T)	-347.328943	-347.217226	-347.315776	-347.276947	-347.378884
PBE	-347.460536	-347.363411	-347.446012	-347.429301	-347.518377
PBE-D3BJ	-347.476234	-347.379090	-347.461560	-347.447536	-347.536006
PW91	-347.763717	-347.666187	-347.749321	-347.732917	-347.821381
M06L	-347.867879	-347.758168	-347.849857	-347.820052	-347.920676
M06L-D3	-347.868442	-347.758764	-347.850460	-347.820574	-347.921205
MN12-L	-347.552182	-347.442004	-347.542197	-347.508338	-347.607794
MN15-L	-347.575265	-347.462579	-347.559440	-347.527377	-347.633662
TPSS	-347.968636	-347.865130	-347.951784	-347.930687	-348.023486
TPSS-D3BJ	-347.988196	-347.884683	-347.971186	-347.953479	-348.045517
B3LYP	-347.918718	-347.809215	-347.903347	-347.871332	-347.970274
B3LYP-D3BJ	-347.944752	-347.835225	-347.929189	-347.901950	-347.999813
PBE0	-347.499384	-347.392700	-347.484858	-347.459225	-347.560383
PBE0-D3BJ	-347.512909	-347.406245	-347.498294	-347.474920	-347.575566
B3PW91	-347.783431	-347.677176	-347.768819	-347.742024	-347.841146
B3PW91-D3	-347.798325	-347.691192	-347.782700	-347.757928	-347.857405
X3LYP	-347.749810	-347.640008	-347.734530	-347.702753	-347.802146
B1LYP	-347.724460	-347.612456	-347.708725	-347.673901	-347.775505
mPW1PW91	-347.828403	-347.720723	-347.813781	-347.786745	-347.888092
B972	-347.792505	-347.683796	-347.777379	-347.746556	-347.848789
MN15	-347.485045	-347.378700	-347.472944	-347.439903	-347.540804
M08HX	-347.809073	-347.695051	-347.795175	-347.760249	-347.864420
M06	-347.664991	-347.557247	-347.648534	-347.617509	-347.718017
M06-D3	-347.666786	-347.559099	-347.650288	-347.619334	-347.719811
M06HF	-347.795498	-347.685225	-347.787517	-347.753233	-347.851180
M06HF-D3	-347.796509	-347.686292	-347.788547	-347.754225	-347.852166
M062X	-347.751988	-347.641179	-347.738675	-347.704398	-347.805310
M062X-D3	-347.752467	-347.641685	-347.739194	-347.704833	-347.805751
M05	-347.666823	-347.558423	-347.647619	-347.613437	-347.719991

Continued on next page

Table S30 (continued)

XC	1	TS_{enol}	2	TS_{conia}	3
M05-D3	-347.671952	-347.563334	-347.652475	-347.618845	-347.725452
M052X	-347.862222	-347.751825	-347.849920	-347.818687	-347.920135
M052X-D3	-347.863377	-347.753039	-347.851084	-347.819831	-347.921265
TPSSH	-347.934228	-347.827426	-347.917591	-347.893133	-347.990518
CAM-B3LYP	-347.723949	-347.612508	-347.708305	-347.673778	-347.779153
CAM-B3LYP-D3BJ	-347.735940	-347.624570	-347.720282	-347.687686	-347.792602
wB97XD	-347.804788	-347.693288	-347.788406	-347.754777	-347.862425
B2PLYP	-347.541479	-347.429113	-347.524283	-347.490878	-347.592845
mPW2PLYP	-347.545292	-347.431555	-347.528304	-347.493975	-347.597784
DSDPBEP86	-347.150375	-347.036363	-347.132634	-347.099605	-347.205898

Table S31: XC/6-31G(d,p) single-point energies on CCSD/6-31G(d,p) optimized geometries for the reaction profile of **hex-5-ynal** (1a) + $\mathbf{H_2O}$.

XC	1	TS_{enol}	2	TS_{conia}	3
CCSD(T)	-384.4419462	-384.3711400	-384.4296563	-384.3639606	-384.4542281
PBE	-384.5538113	-384.5059244	-384.5404984	-384.4963429	-384.5769895
PBE-D3BJ	-384.5701038	-384.5211691	-384.5557535	-384.5129189	-384.5925953
PW91	-384.8779964	-384.8302443	-384.8647345	-384.8205567	-384.9005861
M06L	-384.9880970	-384.9205339	-384.9698137	-384.9118255	-385.0050230
M06L-D3	-384.9887198	-384.9211454	-384.9705018	-384.9123941	-385.0056106
MN12-L	-384.6515040	-384.5831187	-384.6412940	-384.5767951	-384.6662502
MN15-L	-384.6614153	-384.5843111	-384.6438028	-384.5823171	-384.6785561
TPSS	-385.0836743	-385.0287853	-385.0684285	-385.0211168	-385.1058097
TPSS-D3BJ	-385.1040893	-385.0477201	-385.0874824	-385.0417988	-385.1253037
B3LYP	-385.0386952	-384.9773829	-385.0244064	-384.9650427	-385.0556376
B3LYP-D3BJ	-385.0655239	-385.0026384	-385.0495527	-384.9926538	-385.0815648
PBE0	-384.5864564	-384.5292461	-384.5732349	-384.5194499	-384.6107407
PBE0-D3BJ	-384.6006540	-384.5423151	-384.5864888	-384.5337325	-384.6242342
B3PW91	-384.8877025	-384.8315393	-384.8749459	-384.8208447	-384.9106267
B3PW91-D3	-384.9059331	-384.8457441	-384.8905370	-384.8368781	-384.9264083
X3LYP	-384.8630929	-384.8014823	-384.8486967	-384.7892044	-384.8800920
B1LYP	-384.8407664	-384.7763338	-384.8260355	-384.7638746	-384.8570156
mPW1PW91	-384.9362257	-384.8783874	-384.9231757	-384.8682884	-384.9598955
B972	-384.8992270	-384.8382957	-384.8856417	-384.8277649	-384.9211543
MN15	-384.5822609	-384.5184858	-384.5701180	-384.5089890	-384.5998364
M08HX	-384.9091112	-384.8390233	-384.8955656	-384.8302150	-384.9232606
M06	-384.7879095	-384.7229656	-384.7711809	-384.7122221	-384.8029602

Continued on next page

Table S31 (continued)

XC	1	TS_{enol}	2	TS_{conia}	3
M06-D3	-384.7901708	-384.7246782	-384.7732825	-384.7142018	-384.8049098
M06HF	-384.8951394	-384.8361938	-384.8881111	-384.8236109	-384.9083023
M06HF-D3	-384.8963279	-384.8372067	-384.8893448	-384.8246858	-384.9093925
M062X	-384.8618544	-384.7978792	-384.8487359	-384.7846689	-384.8749054
M062X-D3	-384.8623775	-384.7984111	-384.8493180	-384.7851473	-384.8754005
M05	-384.7894982	-384.7217416	-384.7701181	-384.7103438	-384.8096859
M05-D3	-384.7956613	-384.7266557	-384.7756177	-384.7158518	-384.8150996
M052X	-384.9780130	-384.9160291	-384.9659635	-384.9042537	-384.9939498
M052X-D3	-384.9793920	-384.9171676	-384.9673623	-384.9054945	-384.9951984
TPSSH	-385.0447385	-384.9865931	-385.0296924	-384.9786472	-385.0673334
CAM-B3LYP	-384.8427634	-384.7802032	-384.8279277	-384.7654417	-384.8611304
CAM-B3LYP-D3BJ	-384.8554764	-384.7917236	-384.8397937	-384.7780931	-384.8731242
wB97XD	-384.9168996	-384.8499095	-384.9008273	-384.8390873	-384.9367810
B2PLYP	-384.6407600	-384.5739508	-384.6243744	-384.5628135	-384.6548628
mPW2PLYP	-384.6446018	-384.5765115	-384.6281774	-384.5652921	-384.6590558
DSDPBEP86	-384.2191307	-384.1492916	-384.2017341	-384.1395782	-384.2345185

Table S32: XC/6-31G(d,p) single-point energies on CCSD/6-31G(d,p) optimized geometries for the reaction profile of **hept-6-ynal** (1b) + $\mathbf{H_2O}$.

XC	1	TS_{enol}	2	TS_{conia}	3
CCSD(T)	-423.682419	-423.612979	-423.672073	-423.628991	-423.732198
PBE	-423.807634	-423.763065	-423.799409	-423.776371	-423.866883
PBE-D3BJ	-423.825925	-423.782631	-423.818170	-423.797441	-423.886968
PW91	-424.168784	-424.124617	-424.160733	-424.137870	-424.227787
M06L	-424.292300	-424.230144	-424.279608	-424.243784	-424.345343
M06L-D3	-424.293158	-424.231045	-424.280532	-424.244594	-424.346140
MN12-L	-423.911198	-423.849665	-423.906814	-423.866527	-423.966775
MN15-L	-423.926810	-423.858021	-423.916013	-423.878245	-423.984961
TPSS	-424.401695	-424.348838	-424.390288	-424.363673	-424.457706
TPSS-D3BJ	-424.424484	-424.373217	-424.413710	-424.390008	-424.482797
B3LYP	-424.351025	-424.291578	-424.340580	-424.303599	-424.403745
B3LYP-D3BJ	-424.381066	-424.323734	-424.371375	-424.338559	-424.437055
PBE0	-423.849527	-423.795107	-423.840606	-423.809050	-423.911629
PBE0-D3BJ	-423.865370	-423.812056	-423.856936	-423.827300	-423.929015
B3PW91	-424.186449	-424.131508	-424.176951	-424.144975	-424.245388
B3PW91-D3	-424.205991	-424.151276	-424.196283	-424.166026	-424.265945
X3LYP	-424.153361	-424.094171	-424.143180	-424.106174	-424.206808

Continued on next page

Table S32 (continued)

XC	1	TS_{enol}	2	TS_{conia}	3
B1LYP	-424.125480	-424.062820	-424.114441	-424.074861	-424.177619
mPW1PW91	-424.239794	-424.183958	-424.230410	-424.197865	-424.300574
B972	-424.197594	-424.137979	-424.187211	-424.151519	-424.255019
MN15	-423.838762	-423.780847	-423.832272	-423.793175	-423.894917
M08HX	-424.210340	-424.146678	-424.202246	-424.160852	-424.265865
M06	-424.065727	-424.005732	-424.054303	-424.017162	-424.118971
M06-D3	-424.068279	-424.008445	-424.056992	-424.019818	-424.121490
M06HF	-424.191578	-424.137296	-424.188230	-424.148694	-424.246641
M06HF-D3	-424.193072	-424.138887	-424.189828	-424.150165	-424.248074
M062X	-424.151105	-424.092827	-424.143024	-424.102899	-424.204320
M062X-D3	-424.151842	-424.093595	-424.143817	-424.103588	-424.204997
M05	-424.067414	-424.002945	-424.052665	-424.012706	-424.121434
M05-D3	-424.074257	-424.009931	-424.059510	-424.019938	-424.128478
M052X	-424.282089	-424.224763	-424.274625	-424.237885	-424.340165
M052X-D3	-424.283781	-424.226566	-424.276428	-424.239571	-424.341795
TPSSH	-424.360091	-424.303896	-424.348691	-424.318819	-424.417447
CAM-B3LYP	-424.129672	-424.069884	-424.119098	-424.079100	-424.185805
CAM-B3LYP-D3BJ	-424.143775	-424.084990	-424.133744	-424.095354	-424.201277
wB97XD	-424.217309	-424.155075	-424.206435	-424.167412	-424.275729
B2PLYP	-423.904523	-423.840218	-423.891906	-423.853892	-423.956707
mPW2PLYP	-423.909247	-423.844226	-423.896957	-423.857740	-423.962486
DSDPBEP86	-423.437937	-423.372262	-423.425143	-423.387274	-423.494058

5.3 6-31+G(d,p)

Table S33: XC/6-31+G(d,p) single-point energies on CCSD/6-31G(d,p) optimized geometries for the reaction profile of **hex-5-ynal** (1a).

XC	1	TS_{enol}	2	TS_{conia}	3
CCSD(T)	-308.0874483	-307.9784291	-308.0747491	-308.0112212	-308.1013997
PBE	-308.2197527	-308.1246053	-308.2068044	-308.1634492	-308.2443595
PBE-D3BJ	-308.2326414	-308.1371784	-308.2188883	-308.1773271	-308.2573993
PW91	-308.4867565	-308.3912032	-308.4739332	-308.4304877	-308.5107612
M06L	-308.5692240	-308.4611475	-308.5515664	-308.4958037	-308.5887252
M06L-D3	-308.5695949	-308.4615373	-308.5519751	-308.4961137	-308.5890723
MN12-L	-308.2992520	-308.1919477	-308.2898672	-308.2285802	-308.3175781
MN15-L	-308.3165752	-308.2064208	-308.3012903	-308.2423171	-308.3379942
TPSS	-308.6620502	-308.5610378	-308.6471302	-308.6001679	-308.6853996

Continued on next page

Table S33 (continued)

XC	1	TS_{enol}	2	TS_{conia}	3
TPSS-D3BJ	-308.6781776	-308.5767492	-308.6622143	-308.6174806	-308.7016863
B3LYP	-308.6195180	-308.5128673	-308.6064497	-308.5464591	-308.6375741
B3LYP-D3BJ	-308.6410533	-308.5338907	-308.6266674	-308.5699240	-308.6595121
PBE0	-308.2461040	-308.1421493	-308.2332169	-308.1805318	-308.2720643
PBE0-D3BJ	-308.2572353	-308.1529903	-308.2436310	-308.1923900	-308.2832649
B3PW91	-308.4945636	-308.3910610	-308.4819168	-308.4282920	-308.5186361
B3PW91-D3	-308.5063446	-308.4017711	-308.4918049	-308.4396285	-308.5298513
X3LYP	-308.4729815	-308.3660472	-308.4599288	-308.3999360	-308.4912462
B1LYP	-308.4529233	-308.3439682	-308.4395904	-308.3766042	-308.4702992
mPW1PW91	-308.5348381	-308.4299938	-308.5220297	-308.4678980	-308.5599073
B972	-308.5038932	-308.3980649	-308.4907949	-308.4332073	-308.5270326
MN15	-308.2390394	-308.1351102	-308.2279499	-308.1682974	-308.2594519
M08HX	-308.5173299	-308.4061850	-308.5044647	-308.4415826	-308.5347292
M06	-308.3967971	-308.2912738	-308.3813048	-308.3238717	-308.4142310
M06-D3	-308.3981550	-308.2925297	-308.3825219	-308.3250506	-308.4155209
M06HF	-308.5102227	-308.4042063	-308.5041125	-308.4406331	-308.5265713
M06HF-D3	-308.5109427	-308.4049123	-308.5048182	-308.4412520	-308.5272650
M062X	-308.4714075	-308.3634387	-308.4592268	-308.3969657	-308.4877235
M062X-D3	-308.4717157	-308.3637670	-308.4595776	-308.3972213	-308.4880084
M05	-308.3999023	-308.2937276	-308.3819899	-308.3234483	-308.4221635
M05-D3	-308.4038876	-308.2973769	-308.3854179	-308.3272632	-308.4259788
M052X	-308.5671428	-308.4596736	-308.5560241	-308.4955767	-308.5855944
M052X-D3	-308.5679775	-308.4604788	-308.5568232	-308.4962972	-308.5863985
TPSSH	-308.6292566	-308.5251948	-308.6145398	-308.5639888	-308.6531886
CAM-B3LYP	-308.4503520	-308.3416882	-308.4367120	-308.3739418	-308.4700850
CAM-B3LYP-D3BJ	-308.4602567	-308.3513252	-308.4459828	-308.3843676	-308.4799865
wB97XD	-308.5130168	-308.4041715	-308.4979225	-308.4371083	-308.5354683
B2PLYP	-308.2916679	-308.1825094	-308.2768257	-308.2155138	-308.3075225
mPW2PLYP	-308.2946720	-308.1842146	-308.2799378	-308.2173882	-308.3110187
DSDPBEP86	-307.9440576	-307.8332320	-307.9281068	-307.8672211	-307.9619697

Table S34: XC/6-31+G(d,p) single-point energies on CCSD/6-31G(d,p) optimized geometries for the reaction profile of **hept-6-ynal** (1b).

XC	1	TS_{enol}	2	TS_{conia}	3
CCSD(T)	-347.328943	-347.217226	-347.315776	-347.276947	-347.378884
PBE	-347.478106	-347.380804	-347.464584	-347.444444	-347.533668
PBE-D3BJ	-347.493804	-347.396483	-347.480133	-347.462679	-347.551297

Continued on next page

Table S34 (continued)

XC	1	TS_{enol}	2	TS_{conia}	3
PW91	-347.782153	-347.684473	-347.768806	-347.748841	-347.837432
M06L	-347.877031	-347.767489	-347.859895	-347.828030	-347.928478
M06L-D3	-347.877594	-347.768085	-347.860498	-347.828551	-347.929006
MN12-L	-347.563575	-347.454010	-347.554796	-347.518527	-347.617571
MN15-L	-347.587812	-347.475560	-347.573104	-347.538201	-347.644456
TPSS	-347.983568	-347.879997	-347.967750	-347.943614	-348.036354
TPSS-D3BJ	-348.003127	-347.899550	-347.987152	-347.966406	-348.058385
B3LYP	-347.935651	-347.826252	-347.921457	-347.885914	-347.984767
B3LYP-D3BJ	-347.961685	-347.852263	-347.947300	-347.916531	-348.014306
PBE0	-347.512994	-347.406430	-347.499445	-347.470958	-347.572142
PBE0-D3BJ	-347.526519	-347.419975	-347.512881	-347.486654	-347.587325
B3PW91	-347.796472	-347.690281	-347.782814	-347.753269	-347.852377
B3PW91-D3	-347.811366	-347.704297	-347.796695	-347.769173	-347.868635
X3LYP	-347.767298	-347.657637	-347.753229	-347.717817	-347.817120
B1LYP	-347.741393	-347.629553	-347.726861	-347.688473	-347.789963
mPW1PW91	-347.841826	-347.734275	-347.828175	-347.798335	-347.899673
B972	-347.805483	-347.696889	-347.791326	-347.757750	-347.859969
MN15	-347.500496	-347.394367	-347.489419	-347.453126	-347.554146
M08HX	-347.823276	-347.709978	-347.810616	-347.772878	-347.876806
M06	-347.678661	-347.571173	-347.663223	-347.629377	-347.729990
M06-D3	-347.680456	-347.573025	-347.664978	-347.631202	-347.731785
M06HF	-347.810875	-347.701684	-347.804080	-347.767019	-347.864751
M06HF-D3	-347.811887	-347.702751	-347.805110	-347.768011	-347.865737
M062X	-347.765109	-347.654839	-347.752889	-347.715864	-347.816677
M062X-D3	-347.765589	-347.655346	-347.753408	-347.716299	-347.817119
M05	-347.681898	-347.573323	-347.663406	-347.626380	-347.733343
M05-D3	-347.687027	-347.578235	-347.668262	-347.631789	-347.738803
M052X	-347.875347	-347.765330	-347.863968	-347.829928	-347.931411
M052X-D3	-347.876501	-347.766543	-347.865132	-347.831071	-347.932542
TPSSH	-347.947946	-347.841186	-347.932327	-347.905009	-348.002318
CAM-B3LYP	-347.741241	-347.629761	-347.726676	-347.688544	-347.793938
CAM-B3LYP-D3BJ	-347.753233	-347.641822	-347.738652	-347.702452	-347.807387
wB97XD	-347.817797	-347.706383	-347.802424	-347.765915	-347.873541
B2PLYP	-347.559328	-347.447637	-347.543533	-347.507699	-347.608917
mPW2PLYP	-347.563425	-347.450391	-347.547846	-347.510930	-347.614031
DSDPBEP86	-347.167169	-347.054102	-347.150861	-347.116040	-347.221349

Table S35: XC/6-31+G(d,p) single-point energies on CCSD/6-31G(d,p) optimized geometries for the reaction profile of **hex-5-ynal** (1a) + $\mathbf{H_2O}$.

XC	1	TS_{enol}	2	TS_{conia}	3
CCSD(T)	-384.4419462	-384.3711400	-384.4296563	-384.3639606	-384.4542281
PBE	-384.5783794	-384.5289465	-384.5679443	-384.5210036	-384.6029108
PBE-D3BJ	-384.5946719	-384.5441912	-384.5831994	-384.5375796	-384.6185166
PW91	-384.9035072	-384.8543356	-384.8932277	-384.8461575	-384.9274709
M06L	-385.0021017	-384.9333662	-384.9854822	-384.9258396	-385.0195974
M06L-D3	-385.0027245	-384.9339777	-384.9861703	-384.9264081	-385.0201850
MN12-L	-384.6684511	-384.5989992	-384.6599616	-384.5937797	-384.6833199
MN15-L	-384.6792061	-384.6010124	-384.6638468	-384.6003052	-384.6970114
TPSS	-385.1048307	-385.0488305	-385.0922191	-385.0424524	-385.1281004
TPSS-D3BJ	-385.1252457	-385.0677652	-385.1112731	-385.0631345	-385.1475944
B3LYP	-385.0619131	-384.9997529	-385.0506134	-384.9883882	-385.0800583
B3LYP-D3BJ	-385.0887417	-385.0250084	-385.0757597	-385.0159993	-385.1059854
PBE0	-384.6056873	-384.5474639	-384.5949690	-384.5388473	-384.6310584
PBE0-D3BJ	-384.6198849	-384.5605329	-384.6082228	-384.5531299	-384.6445518
B3PW91	-384.9063686	-384.8491309	-384.8959605	-384.8396704	-384.9302970
B3PW91-D3	-384.9245992	-384.8633357	-384.9115517	-384.8557038	-384.9460786
X3LYP	-384.8869170	-384.8245126	-384.8756071	-384.8131499	-384.9051467
B1LYP	-384.8638298	-384.7987003	-384.8521219	-384.7870699	-384.8812618
mPW1PW91	-384.9551520	-384.8964191	-384.9445367	-384.8873916	-384.9798450
B972	-384.9174726	-384.8557303	-384.9063558	-384.8462585	-384.9404983
MN15	-384.6047639	-384.5388213	-384.5946795	-384.5310011	-384.6226724
M08HX	-384.9296535	-384.8585913	-384.9185468	-384.8508809	-384.9445293
M06	-384.8075623	-384.7413466	-384.7930429	-384.7318532	-384.8235206
M06-D3	-384.8098237	-384.7430592	-384.7951445	-384.7338329	-384.8254703
M06HF	-384.9164484	-384.8571516	-384.9122300	-384.8450843	-384.9307066
M06HF-D3	-384.9176369	-384.8581644	-384.9134636	-384.8461593	-384.9317968
M062X	-384.8805544	-384.8155924	-384.8697525	-384.8033913	-384.8943371
M062X-D3	-384.8810776	-384.8161243	-384.8703346	-384.8038697	-384.8948322
M05	-384.8105487	-384.7416321	-384.7937350	-384.7315398	-384.8324145
M05-D3	-384.8167118	-384.7465462	-384.7992346	-384.7370477	-384.8378282
M052X	-384.9969945	-384.9337452	-384.9872462	-384.9231761	-385.0137116
M052X-D3	-384.9983736	-384.9348837	-384.9886451	-384.9244169	-385.0149602
TPSSH	-385.0642217	-385.0051255	-385.0516955	-384.9983289	-385.0878804
CAM-B3LYP	-384.8667475	-384.8029748	-384.8547521	-384.7893207	-384.8861741
CAM-B3LYP-D3BJ	-384.8794605	-384.8144952	-384.8666181	-384.8019721	-384.8981679
wB97XD	-384.9356733	-384.8674207	-384.9218508	-384.8577479	-384.9563150

Continued on next page

Table S35 (continued)

XC	1	TS_{enol}	2	TS_{conia}	3
B2PLYP	-384.6653154	-384.5980550	-384.6521067	-384.5884531	-384.6807872
mPW2PLYP	-384.6693004	-384.6008928	-384.6561218	-384.5910067	-384.6851257
DSDPBEP86	-384.2425016	-384.1722138	-384.2281009	-384.1643162	-384.2591609

Table S36: XC/6-31+G(d,p) single-point energies on CCSD/6-31G(d,p) optimized geometries for the reaction profile of **hept-6-ynal** (1b) + $\mathbf{H_2O}$.

XC	1	TS_{enol}	2	TS_{conia}	3
CCSD(T)	-423.682419	-423.612979	-423.672073	-423.628991	-423.732198
PBE	-423.836435	-423.786429	-423.827522	-423.801278	-423.892562
PBE-D3BJ	-423.854725	-423.805995	-423.846283	-423.822348	-423.912647
PW91	-424.198682	-424.149062	-424.189948	-424.163764	-424.254474
M06L	-424.308365	-424.242860	-424.295482	-424.257764	-424.359459
M06L-D3	-424.309223	-424.243761	-424.296406	-424.258574	-424.360256
MN12-L	-423.930124	-423.865435	-423.925768	-423.883502	-423.983590
MN15-L	-423.947414	-423.874841	-423.936528	-423.896263	-424.003148
TPSS	-424.426435	-424.368958	-424.414497	-424.385118	-424.479661
TPSS-D3BJ	-424.449224	-424.393338	-424.437919	-424.411454	-424.504752
B3LYP	-424.378240	-424.314052	-424.367353	-424.327007	-424.427829
B3LYP-D3BJ	-424.408281	-424.346207	-424.398148	-424.361967	-424.461139
PBE0	-423.872032	-423.813432	-423.862712	-423.828485	-423.931625
PBE0-D3BJ	-423.887875	-423.830381	-423.879042	-423.846735	-423.949011
B3PW91	-424.208226	-424.149142	-424.198296	-424.163813	-424.264711
B3PW91-D3	-424.227769	-424.168910	-424.217629	-424.184864	-424.285268
X3LYP	-424.181301	-424.117329	-424.170689	-424.130198	-424.231538
B1LYP	-424.152517	-424.085241	-424.141078	-424.098093	-424.201524
mPW1PW91	-424.261882	-424.202016	-424.252116	-424.216999	-424.320211
B972	-424.219017	-424.155432	-424.208272	-424.170032	-424.274049
MN15	-423.864403	-423.801710	-423.857385	-423.815405	-423.917548
M08HX	-424.233680	-424.166434	-424.225684	-424.181566	-424.286886
M06	-424.088292	-424.024199	-424.076605	-424.036786	-424.139137
M06-D3	-424.090844	-424.026912	-424.079293	-424.039442	-424.141656
M06HF	-424.216177	-424.158729	-424.212946	-424.170369	-424.268880
M06HF-D3	-424.217671	-424.160319	-424.214543	-424.171841	-424.270313
M062X	-424.172545	-424.110750	-424.164505	-424.121691	-424.223455
M062X-D3	-424.173282	-424.111518	-424.165298	-424.122380	-424.224132
M05	-424.092193	-424.023054	-424.076849	-424.033960	-424.143809
M05-D3	-424.099036	-424.030040	-424.083695	-424.041193	-424.150853

Continued on next page

Table S36 (continued)

XC	1	TS_{enol}	2	TS_{conia}	3
M052X	-424.303981	-424.242676	-424.296282	-424.256769	-424.359548
M052X-D3	-424.305673	-424.244479	-424.298085	-424.258455	-424.361179
TPSSH	-424.382865	-424.322452	-424.371033	-424.338549	-424.437645
CAM-B3LYP	-424.157566	-424.092756	-424.146455	-424.103026	-424.210488
CAM-B3LYP-D3BJ	-424.171669	-424.107861	-424.161102	-424.119280	-424.225959
wB97XD	-424.239023	-424.172669	-424.227798	-424.186081	-424.294883
B2PLYP	-423.932882	-423.864871	-423.920490	-423.879857	-423.982571
mPW2PLYP	-423.937827	-423.869107	-423.925746	-423.883763	-423.988487
DSDPBEP86	-423.464699	-423.395947	-423.452415	-423.412449	-423.518761

5.4 6-311G(d,p)

Table S37: XC/6-311G(d,p) single-point energies on CCSD/6-31G(d,p) optimized geometries for the reaction profile of **hex-5-ynal** (1a).

XC	1	TS_{enol}	2	TS_{conia}	3
CCSD(T)	-308.0874483	-307.9784291	-308.0747491	-308.0112212	-308.1013997
PBE	-308.2782457	-308.1832446	-308.2657438	-308.2208781	-308.2995418
PBE-D3BJ	-308.2911344	-308.1958177	-308.2778277	-308.2347560	-308.3125816
PW91	-308.5471176	-308.4516044	-308.5347145	-308.4896355	-308.5676690
M06L	-308.6325110	-308.5244486	-308.6150169	-308.5569330	-308.6485227
M06L-D3	-308.6328819	-308.5248385	-308.6154256	-308.5572430	-308.6488697
MN12-L	-308.3477574	-308.2403086	-308.3375030	-308.2760523	-308.3640449
MN15-L	-308.3854044	-308.2756923	-308.3698222	-308.3116791	-308.4059137
TPSS	-308.7234071	-308.6225043	-308.7089666	-308.6602834	-308.7435141
TPSS-D3BJ	-308.7395345	-308.6382157	-308.7240507	-308.6775961	-308.7598008
B3LYP	-308.6809358	-308.5740689	-308.6683912	-308.6061208	-308.6951519
B3LYP-D3BJ	-308.7024712	-308.5950923	-308.6886089	-308.6295856	-308.7170899
PBE0	-308.3021050	-308.1983538	-308.2896584	-308.2355918	-308.3249719
PBE0-D3BJ	-308.3132363	-308.2091949	-308.3000725	-308.2474500	-308.3361725
B3PW91	-308.5533293	-308.4499218	-308.5412029	-308.4857814	-308.5740297
B3PW91-D3	-308.5651103	-308.4606319	-308.5510910	-308.4971178	-308.5852449
X3LYP	-308.5338977	-308.4267385	-308.5213376	-308.4591409	-308.5483438
B1LYP	-308.5138037	-308.4046234	-308.5009854	-308.4357861	-308.5273954
mPW1PW91	-308.5923391	-308.4876029	-308.5799604	-308.5242951	-308.6141987
B972	-308.5616236	-308.4559062	-308.5488681	-308.4899951	-308.5819348
MN15	-308.2947875	-308.1902750	-308.2841460	-308.2225135	-308.3113395
M08HX	-308.5800999	-308.4687572	-308.5673343	-308.5035898	-308.5948301

Continued on next page

Table S37 (continued)

XC	1	TS_{enol}	2	TS_{conia}	3
M06	-308.4542550	-308.3481397	-308.4391003	-308.3786784	-308.4673396
M06-D3	-308.4556129	-308.3493956	-308.4403173	-308.3798574	-308.4686295
M06HF	-308.5767390	-308.4711189	-308.5712328	-308.5070110	-308.5904424
M06HF-D3	-308.5774590	-308.4718249	-308.5719385	-308.5076300	-308.5911361
M062X	-308.5390071	-308.4307382	-308.5271252	-308.4634216	-308.5524295
M062X-D3	-308.5393153	-308.4310665	-308.5274760	-308.4636772	-308.5527143
M05	-308.4408910	-308.3351574	-308.4235579	-308.3627767	-308.4591748
M05-D3	-308.4448764	-308.3388068	-308.4269858	-308.3665916	-308.4629900
M052X	-308.6290362	-308.5213637	-308.6183495	-308.5564502	-308.6442557
M052X-D3	-308.6298710	-308.5221689	-308.6191486	-308.5571708	-308.6450598
TPSSH	-308.6891911	-308.5852567	-308.6749321	-308.6227444	-308.7099692
CAM-B3LYP	-308.5123455	-308.4036832	-308.4994172	-308.4342287	-308.5280195
CAM-B3LYP-D3BJ	-308.5222502	-308.4133203	-308.5086880	-308.4446545	-308.5379210
wB97XD	-308.5716938	-308.4629378	-308.5569762	-308.4941080	-308.5905751
B2PLYP	-308.3635984	-308.2550419	-308.3489981	-308.2866299	-308.3759630
mPW2PLYP	-308.3648478	-308.2548964	-308.3503258	-308.2867422	-308.3777391
DSDPBEP86	-308.0208392	-307.9111115	-308.0049607	-307.9438645	-308.0357478

Table S38: XC/6-311G(d,p) single-point energies on CCSD/6-31G(d,p) optimized geometries for the reaction profile of **hept-6-ynal** (1b).

XC	1	TS_{enol}	2	TS_{conia}	3
CCSD(T)	-347.328943	-347.217226	-347.315776	-347.276947	-347.378884
PBE	-347.543168	-347.445973	-347.530353	-347.509049	-347.596341
PBE-D3BJ	-347.558866	-347.461652	-347.545902	-347.527284	-347.613970
PW91	-347.849330	-347.751637	-347.836666	-347.815462	-347.902125
M06L	-347.947816	-347.838191	-347.930803	-347.896869	-347.996077
M06L-D3	-347.948379	-347.838786	-347.931406	-347.897390	-347.996605
MN12-L	-347.617640	-347.508116	-347.608177	-347.571657	-347.670337
MN15-L	-347.664855	-347.553086	-347.650124	-347.615541	-347.720959
TPSS	-348.051740	-347.948280	-348.036618	-348.011108	-348.102119
TPSS-D3BJ	-348.071300	-347.967833	-348.056020	-348.033900	-348.124150
B3LYP	-348.003900	-347.894234	-347.990502	-347.953106	-348.050088
B3LYP-D3BJ	-348.029934	-347.920244	-348.016344	-347.983723	-348.079626
PBE0	-347.575150	-347.468779	-347.562245	-347.532576	-347.631941
PBE0-D3BJ	-347.588675	-347.482323	-347.575681	-347.548271	-347.647123
B3PW91	-347.861668	-347.755578	-347.848724	-347.817704	-347.915005
B3PW91-D3	-347.876563	-347.769594	-347.862605	-347.833608	-347.931264

Continued on next page

Table S38 (continued)

XC	1	TS_{enol}	2	TS_{conia}	3
X3LYP	-347.835006	-347.725062	-347.821712	-347.784510	-347.881926
B1LYP	-347.809045	-347.696935	-347.795305	-347.755103	-347.854728
mPW1PW91	-347.905648	-347.798216	-347.892620	-347.861498	-347.961055
B972	-347.869607	-347.761121	-347.855976	-347.821241	-347.921900
MN15	-347.562455	-347.455580	-347.552150	-347.514420	-347.613634
M08HX	-347.892921	-347.779446	-347.880629	-347.842312	-347.944522
M06	-347.743031	-347.634840	-347.728040	-347.691593	-347.790968
M06-D3	-347.744826	-347.636692	-347.729795	-347.693418	-347.792762
M06HF	-347.884228	-347.775425	-347.878399	-347.841176	-347.936554
M06HF-D3	-347.885239	-347.776492	-347.879429	-347.842168	-347.937540
M062X	-347.840568	-347.729941	-347.828889	-347.790595	-347.889872
M062X-D3	-347.841047	-347.730447	-347.829408	-347.791031	-347.890313
M05	-347.726682	-347.618493	-347.708959	-347.669490	-347.774481
M05-D3	-347.731811	-347.623405	-347.713815	-347.674899	-347.779941
M052X	-347.944290	-347.834008	-347.933531	-347.898608	-347.998045
M052X-D3	-347.945444	-347.835222	-347.934694	-347.899752	-347.999175
TPSSH	-348.014493	-347.907866	-347.999529	-347.970860	-348.066474
CAM-B3LYP	-347.810157	-347.698605	-347.796562	-347.756474	-347.859725
CAM-B3LYP-D3BJ	-347.822149	-347.710666	-347.808539	-347.770382	-347.873174
wB97XD	-347.882879	-347.771528	-347.868078	-347.829651	-347.935668
B2PLYP	-347.639512	-347.528265	-347.624271	-347.587497	-347.686443
mPW2PLYP	-347.641650	-347.528972	-347.626598	-347.588757	-347.689636
DSDPBEP86	-347.252809	-347.140634	-347.236872	-347.201703	-347.304669

Table S39: XC/6-311G(d,p) single-point energies on CCSD/6-31G(d,p) optimized geometries for the reaction profile of **hex-5-ynal** (1a) + $\mathbf{H_2O}$.

XC	1	TS_{enol}	2	TS_{conia}	3
CCSD(T)	-384.4419462	-384.3711400	-384.4296563	-384.3639606	-384.4542281
PBE	-384.6558693	-384.6068476	-384.6440060	-384.5963710	-384.6748641
PBE-D3BJ	-384.6721618	-384.6220922	-384.6592611	-384.6129471	-384.6904699
PW91	-384.9835031	-384.9345423	-384.9717242	-384.9238237	-385.0016864
M06L	-385.0829936	-385.0142768	-385.0654162	-385.0041537	-385.0957685
M06L-D3	-385.0836165	-385.0148882	-385.0661043	-385.0047222	-385.0963561
MN12-L	-384.7324927	-384.6631668	-384.7222444	-384.6562504	-384.7440002
MN15-L	-384.7686894	-384.6921961	-384.7518711	-384.6896463	-384.7840310
TPSS	-385.1867392	-385.1306275	-385.1729281	-385.1221363	-385.2047130
TPSS-D3BJ	-385.2071543	-385.1495623	-385.1919821	-385.1428183	-385.2242070

Continued on next page

Table S39 (continued)

XC	1	TS_{enol}	2	TS_{conia}	3
B3LYP	-385.1433574	-385.0809613	-385.1308501	-385.0671576	-385.1556091
B3LYP-D3BJ	-385.1701860	-385.1062168	-385.1559964	-385.0947687	-385.1815362
PBE0	-384.6806408	-384.6225983	-384.6687209	-384.6119043	-384.7010477
PBE0-D3BJ	-384.6948384	-384.6356672	-384.6819748	-384.6261869	-384.7145412
B3PW91	-384.9849457	-384.9277002	-384.9735157	-384.9160369	-385.0037428
B3PW91-D3	-385.0031762	-384.9419050	-384.9891068	-384.9320702	-385.0195244
X3LYP	-384.9677088	-384.9050761	-384.9551192	-384.8912934	-384.9800002
B1LYP	-384.9446405	-384.8792433	-384.9317268	-384.8652741	-384.9562384
mPW1PW91	-385.0321998	-384.9734219	-385.0204187	-384.9623562	-385.0518480
B972	-384.9946630	-384.9328604	-384.9822892	-384.9215764	-385.0130230
MN15	-384.6808382	-384.6150380	-384.6699453	-384.6046719	-384.6933760
M08HX	-385.0147209	-384.9429497	-385.0021338	-384.9340505	-385.0250534
M06	-384.8814249	-384.8153296	-384.8658318	-384.8023306	-384.8914985
M06-D3	-384.8836863	-384.8170422	-384.8679334	-384.8043103	-384.8934482
M06HF	-385.0121213	-384.9517116	-385.0066016	-384.9388984	-385.0216312
M06HF-D3	-385.0133098	-384.9527244	-385.0078352	-384.9399734	-385.0227214
M062X	-384.9706364	-384.9046137	-384.9585988	-384.8912960	-384.9798905
M062X-D3	-384.9711596	-384.9051456	-384.9591809	-384.8917744	-384.9803856
M05	-384.8654865	-384.7973064	-384.8475086	-384.7841461	-384.8813767
M05-D3	-384.8716496	-384.8022205	-384.8530083	-384.7896541	-384.8867903
M052X	-385.0807974	-385.0173787	-385.0699469	-385.0048847	-385.0925654
M052X-D3	-385.0821765	-385.0185172	-385.0713458	-385.0061255	-385.0938140
TPSSH	-385.1444819	-385.0852392	-385.1308036	-385.0764617	-385.1630521
CAM-B3LYP	-384.9487749	-384.8851079	-384.9357316	-384.8686693	-384.9620668
CAM-B3LYP-D3BJ	-384.9614880	-384.8966283	-384.9475977	-384.8813206	-384.9740605
wB97XD	-385.0134106	-384.9450878	-384.9984619	-384.9329294	-385.0287701
B2PLYP	-384.7613938	-384.6949890	-384.7467597	-384.6828339	-384.7713546
mPW2PLYP	-384.7631524	-384.6954482	-384.7484792	-384.6831273	-384.7734450
DSDPBEP86	-384.3458284	-384.2770950	-384.3299733	-384.2666527	-384.3576141

Table S40: XC/6-311G(d,p) single-point energies on CCSD/6-31G(d,p) optimized geometries for the reaction profile of **hept-6-ynal** (1b) + $\mathbf{H_2O}$.

XC	1	TS_{enol}	2	TS_{conia}	3
CCSD(T)	-423.682419	-423.612979	-423.672073	-423.628991	-423.732198
PBE	-423.917849	-423.871025	-423.910597	-423.884162	-423.972512
PBE-D3BJ	-423.936140	-423.890591	-423.929358	-423.905232	-423.992597
PW91	-424.282733	-424.236232	-424.275709	-424.249250	-424.336965

Continued on next page

Table S40 (continued)

XC	1	TS_{enol}	2	TS_{conia}	3
M06L	-424.395215	-424.331321	-424.383232	-424.344017	-424.443830
M06L-D3	-424.396073	-424.332222	-424.384157	-424.344827	-424.444628
MN12-L	-423.998173	-423.935546	-423.994105	-423.951948	-424.051048
MN15-L	-424.043431	-423.974350	-424.033324	-423.993982	-424.099454
TPSS	-424.512768	-424.457728	-424.502323	-424.472476	-424.564369
TPSS-D3BJ	-424.535557	-424.482107	-424.525746	-424.498811	-424.589460
B3LYP	-424.464109	-424.402245	-424.454817	-424.413612	-424.511559
B3LYP-D3BJ	-424.494151	-424.434400	-424.485612	-424.448572	-424.544869
PBE0	-423.950961	-423.894817	-423.942928	-423.908403	-424.008948
PBE0-D3BJ	-423.966803	-423.911766	-423.959258	-423.926653	-424.026334
B3PW91	-424.291155	-424.234269	-424.282588	-424.247406	-424.345795
B3PW91-D3	-424.310698	-424.254037	-424.301921	-424.268457	-424.366352
X3LYP	-424.266411	-424.204838	-424.257396	-424.216144	-424.314548
B1LYP	-424.237714	-424.172727	-424.227845	-424.184049	-424.284601
mPW1PW91	-424.343104	-424.285492	-424.334623	-424.299021	-424.399709
B972	-424.300489	-424.239079	-424.290891	-424.252331	-424.354026
MN15	-423.944812	-423.884008	-423.939262	-423.896513	-423.996329
M08HX	-424.323529	-424.257719	-424.316508	-424.272610	-424.375478
M06	-424.167037	-424.105084	-424.156738	-424.115004	-424.215416
M06-D3	-424.169589	-424.107797	-424.159427	-424.117660	-424.217935
M06HF	-424.316366	-424.260211	-424.314257	-424.272392	-424.368106
M06HF-D3	-424.317860	-424.261802	-424.315855	-424.273864	-424.369538
M062X	-424.268637	-424.207637	-424.261528	-424.218232	-424.317901
M062X-D3	-424.269374	-424.208405	-424.262321	-424.218920	-424.318578
M05	-424.148526	-424.082546	-424.134784	-424.090678	-424.197423
M05-D3	-424.155369	-424.089533	-424.141630	-424.097911	-424.204467
M052X	-424.392892	-424.333309	-424.386303	-424.346613	-424.446832
M052X-D3	-424.394585	-424.335112	-424.388106	-424.348300	-424.448462
TPSSH	-424.467501	-424.409315	-424.457015	-424.424063	-424.520620
CAM-B3LYP	-424.244086	-424.181925	-424.234715	-424.190357	-424.294698
CAM-B3LYP-D3BJ	-424.258189	-424.197031	-424.249361	-424.206611	-424.310170
wB97XD	-424.321101	-424.256753	-424.311098	-424.268320	-424.374806
B2PLYP	-424.034879	-423.970105	-424.023719	-423.983223	-424.082708
mPW2PLYP	-424.037339	-423.971784	-424.026480	-423.984668	-424.086169
DSDPBEP86	-423.574737	-423.509619	-423.563371	-423.524103	-423.627265

5.5 6-311++G(2d,2p)

Table S41: XC/6-311++G(2d,2p) single-point energies on CCSD/6-31G(d,p) optimized geometries for the reaction profile of **hex-5-ynal** (1a).

XC	1	TS_{enol}	2	TS_{conia}	3
CCSD(T)	-308.0874483	-307.9784291	-308.0747491	-308.0112212	-308.1013997
PBE	-308.2948353	-308.1998385	-308.2839064	-308.2377060	-308.3159851
PBE-D3BJ	-308.3077240	-308.2124117	-308.2959903	-308.2515838	-308.3290250
PW91	-308.5644993	-308.4689393	-308.5536371	-308.5071799	-308.5849158
M06L	-308.6445498	-308.5363856	-308.6280526	-308.5697188	-308.6610559
M06L-D3	-308.6449207	-308.5367755	-308.6284614	-308.5700288	-308.6614030
MN12-L	-308.3643739	-308.2571041	-308.3549130	-308.2934833	-308.3810464
MN15-L	-308.4007179	-308.2914906	-308.3863916	-308.3275542	-308.4208385
TPSS	-308.7403426	-308.6393092	-308.7270309	-308.6773368	-308.7601782
TPSS-D3BJ	-308.7564699	-308.6550206	-308.7421150	-308.6946495	-308.7764650
B3LYP	-308.6983886	-308.5915439	-308.6872127	-308.6235508	-308.7125068
B3LYP-D3BJ	-308.7199239	-308.6125673	-308.7074304	-308.6470157	-308.7344448
PBE0	-308.3183580	-308.2147762	-308.3072127	-308.2522516	-308.3410754
PBE0-D3BJ	-308.3294893	-308.2256172	-308.3176269	-308.2641098	-308.3522759
B3PW91	-308.5694651	-308.4661148	-308.5585757	-308.5022749	-308.5900764
B3PW91-D3	-308.5812461	-308.4768250	-308.5684638	-308.5136114	-308.6012916
X3LYP	-308.5516248	-308.4445125	-308.5404582	-308.4768312	-308.5659584
B1LYP	-308.5314646	-308.4223458	-308.5199875	-308.4533888	-308.5449353
mPW1PW91	-308.6089820	-308.5043572	-308.5978322	-308.5412999	-308.6307108
B972	-308.5772434	-308.4716494	-308.5656895	-308.5057777	-308.5972452
MN15	-308.3098354	-308.2055246	-308.3001468	-308.2383176	-308.3268868
M08HX	-308.6011652	-308.4897288	-308.5896222	-308.5249344	-308.6159393
M06	-308.4688841	-308.3630225	-308.4551227	-308.3943132	-308.4823526
M06-D3	-308.4702421	-308.3642784	-308.4563398	-308.3954921	-308.4836426
M06HF	-308.6066274	-308.4998629	-308.6013507	-308.5366093	-308.6205722
M06HF-D3	-308.6073474	-308.5005689	-308.6020565	-308.5372282	-308.6212659
M062X	-308.5568065	-308.4485326	-308.5460600	-308.4813754	-308.5699983
M062X-D3	-308.5571146	-308.4488609	-308.5464108	-308.4816310	-308.5702831
M05	-308.4570165	-308.3513918	-308.4410487	-308.3794697	-308.4755715
M05-D3	-308.4610018	-308.3550411	-308.4444766	-308.3832846	-308.4793868
M052X	-308.6489781	-308.5411661	-308.6394875	-308.5766243	-308.6639686
M052X-D3	-308.6498128	-308.5419713	-308.6402867	-308.5773448	-308.6647727
TPSSH	-308.7059825	-308.6019985	-308.6928028	-308.6397256	-308.7265014
CAM-B3LYP	-308.5302083	-308.4214597	-308.5184891	-308.4521395	-308.5458812
CAM-B3LYP-D3BJ	-308.5401130	-308.4310968	-308.5277599	-308.4625653	-308.5557827
wB97XD	-308.5878144	-308.4789926	-308.5741560	-308.5106736	-308.6069132

Continued on next page

Table S41 (continued)

XC	1	TS_{enol}	2	TS_{conia}	3
B2PLYP	-308.4017209	-308.2933018	-308.3889704	-308.3254342	-308.4135372
mPW2PLYP	-308.4017275	-308.2919468	-308.3890001	-308.3242335	-308.4140895
DSDPBEP86	-308.0697314	-307.9602057	-308.0558405	-307.9938214	-308.0838893

Table S42: XC/6-311++G(2d,2p) single-point energies on CCSD/6-31G(d,p) optimized geometries for the reaction profile of **hept-6-ynal** (1b).

XC	1	TS_{enol}	2	TS_{conia}	3
CCSD(T)	-347.328943	-347.217226	-347.315776	-347.276947	-347.378884
PBE	-347.561481	-347.464285	-347.549946	-347.526935	-347.614104
PBE-D3BJ	-347.577178	-347.479964	-347.565495	-347.545170	-347.631733
PW91	-347.868566	-347.770827	-347.857147	-347.834215	-347.920799
M06L	-347.961289	-347.851560	-347.945154	-347.910539	-348.009391
M06L-D3	-347.961852	-347.852155	-347.945757	-347.911061	-348.009919
MN12-L	-347.636143	-347.526764	-347.627182	-347.590331	-347.688702
MN15-L	-347.681544	-347.570290	-347.667737	-347.632059	-347.736821
TPSS	-348.070576	-347.966949	-348.056303	-348.029412	-348.120239
TPSS-D3BJ	-348.090136	-347.986502	-348.075706	-348.052204	-348.142270
B3LYP	-348.023329	-347.913618	-348.011008	-347.971952	-348.068972
B3LYP-D3BJ	-348.049364	-347.939628	-348.036851	-348.002569	-348.098511
PBE0	-347.593203	-347.486952	-347.581336	-347.550367	-347.649492
PBE0-D3BJ	-347.606728	-347.500497	-347.594772	-347.566063	-347.664675
B3PW91	-347.879679	-347.773596	-347.867718	-347.835444	-347.932559
B3PW91-D3	-347.894574	-347.787613	-347.881599	-347.851348	-347.948818
X3LYP	-347.854720	-347.744753	-347.842520	-347.803618	-347.901073
B1LYP	-347.828708	-347.716572	-347.816009	-347.774143	-347.873807
mPW1PW91	-347.924201	-347.816822	-347.912137	-347.879762	-347.979101
B972	-347.886921	-347.778515	-347.874240	-347.838117	-347.938487
MN15	-347.579152	-347.472477	-347.569536	-347.531223	-347.630581
M08HX	-347.916769	-347.803181	-347.905370	-347.865829	-347.967976
M06	-347.759336	-347.651363	-347.745526	-347.708362	-347.807406
M06-D3	-347.761131	-347.653215	-347.747281	-347.710187	-347.809201
M06HF	-347.918857	-347.808880	-347.912910	-347.874676	-347.971133
M06HF-D3	-347.919868	-347.809947	-347.913940	-347.875668	-347.972119
M062X	-347.860537	-347.749862	-347.849731	-347.809997	-347.909161
M062X-D3	-347.861017	-347.750369	-347.850250	-347.810433	-347.909603
M05	-347.744604	-347.636513	-347.727993	-347.687164	-347.792133
M05-D3	-347.749733	-347.641425	-347.732849	-347.692572	-347.797594

Continued on next page

Table S42 (continued)

XC	1	TS_{enol}	2	TS_{conia}	3
M052X	-347.966798	-347.856319	-347.956995	-347.920796	-348.019972
M052X-D3	-347.967953	-347.857533	-347.958159	-347.921940	-348.021103
TPSSH	-348.033197	-347.926469	-348.019041	-347.989099	-348.084493
CAM-B3LYP	-347.830043	-347.718306	-347.817385	-347.775806	-347.879185
CAM-B3LYP-D3BJ	-347.842034	-347.730367	-347.829362	-347.789714	-347.892634
wB97XD	-347.900949	-347.789493	-347.886983	-347.847484	-347.953565
B2PLYP	-347.682406	-347.571299	-347.668794	-347.630699	-347.728588
mPW2PLYP	-347.683110	-347.570593	-347.669621	-347.630436	-347.730340
DSDPBEP86	-347.307987	-347.196060	-347.293868	-347.257710	-347.359118

Table S43: XC/6-311++G(2d,2p) single-point energies on CCSD/6-31G(d,p) optimized geometries for the reaction profile of **hex-5-ynal** (1a) + $\mathbf{H_2O}$.

XC	1	TS_{enol}	2	TS_{conia}	3
CCSD(T)	-384.4419462	-384.3711400	-384.4296563	-384.3639606	-384.4542281
PBE	-384.6802404	-384.6303868	-384.6708769	-384.6217410	-384.7009830
PBE-D3BJ	-384.6965329	-384.6456314	-384.6861320	-384.6383170	-384.7165888
PW91	-385.0086607	-384.9588949	-384.9993912	-384.9499515	-385.0286463
M06L	-385.1015920	-385.0319294	-385.0854776	-385.0237225	-385.1159001
M06L-D3	-385.1022148	-385.0325408	-385.0861657	-385.0242910	-385.1164877
MN12-L	-384.7562827	-384.6861109	-384.7471081	-384.6811638	-384.7691511
MN15-L	-384.7918066	-384.7145252	-384.7768390	-384.7137768	-384.8081944
TPSS	-385.2102391	-385.1535358	-385.1983907	-385.1464862	-385.2296453
TPSS-D3BJ	-385.2306541	-385.1724706	-385.2174446	-385.1671683	-385.2491393
B3LYP	-385.1680850	-385.1049828	-385.1577947	-385.0925317	-385.1819510
B3LYP-D3BJ	-385.1949136	-385.1302382	-385.1829410	-385.1201428	-385.2078782
PBE0	-384.7037098	-384.6453103	-384.6938110	-384.6359998	-384.7254342
PBE0-D3BJ	-384.7179074	-384.6583793	-384.7070649	-384.6502824	-384.7389277
B3PW91	-385.0077175	-384.9499989	-384.9981678	-384.9397722	-385.0278196
B3PW91-D3	-385.0259480	-384.9642037	-385.0137590	-384.9558056	-385.0436012
X3LYP	-384.9928007	-384.9294853	-384.9824864	-384.9170312	-385.0067318
B1LYP	-384.9695215	-384.9034896	-384.9587989	-384.8907604	-384.9826957
mPW1PW91	-385.0554856	-384.9963845	-385.0456183	-384.9866403	-385.0764179
B972	-385.0167832	-384.9546648	-385.0063346	-384.9444988	-385.0363217
MN15	-384.7032285	-384.6365453	-384.6937893	-384.6282416	-384.7172896
M08HX	-385.0426182	-384.9704715	-385.0317893	-384.9629093	-385.0541572
M06	-384.9035511	-384.8365759	-384.8898942	-384.8258597	-384.9152160
M06-D3	-384.9058125	-384.8382885	-384.8919957	-384.8278394	-384.9171657

Continued on next page

Table S43 (continued)

XC	1	TS_{enol}	2	TS_{conia}	3
M06HF	-385.0475036	-384.9870195	-385.0428240	-384.9749393	-385.0582255
M06HF-D3	-385.0486921	-384.9880324	-385.0440577	-384.9760143	-385.0593157
M062X	-384.9947387	-384.9283777	-384.9843223	-384.9160725	-385.0048228
M062X-D3	-384.9952618	-384.9289095	-384.9849044	-384.9165508	-385.0053179
M05	-384.8886717	-384.8199771	-384.8729722	-384.8085042	-384.9065523
M05-D3	-384.8948348	-384.8248912	-384.8784719	-384.8140122	-384.9119659
M052X	-385.1073219	-385.0436329	-385.0982895	-385.0322635	-385.1199697
M052X-D3	-385.1087010	-385.0447714	-385.0996883	-385.0335042	-385.1212183
TPSSH	-385.1675998	-385.1079184	-385.1557628	-385.1004480	-385.1874683
CAM-B3LYP	-384.9742463	-384.9096480	-384.9632622	-384.8948445	-384.9891289
CAM-B3LYP-D3BJ	-384.9869593	-384.9211683	-384.9751282	-384.9074959	-385.0011227
wB97XD	-385.0363703	-384.9672067	-385.0230621	-384.9568232	-385.0532009
B2PLYP	-384.8134975	-384.7465276	-384.8014408	-384.7360288	-384.8244989
mPW2PLYP	-384.8134770	-384.7452973	-384.8013667	-384.7345084	-384.8248536
DSDPBEP86	-384.4119870	-384.3427723	-384.3986998	-384.3341350	-384.4244244

Table S44: XC/6-311++G(2d,2p) single-point energies on CCSD/6-31G(d,p) optimized geometries for the reaction profile of **hept-6-ynal** (1b) + $\mathbf{H_2O}$.

XC	1	TS_{enol}	2	TS_{conia}	3
CCSD(T)	-423.682419	-423.612979	-423.672073	-423.628991	-423.732198
PBE	-423.946360	-423.896009	-423.938648	-423.910252	-423.999526
PBE-D3BJ	-423.964651	-423.915575	-423.957409	-423.931322	-424.019611
PW91	-424.312207	-424.262126	-424.304684	-424.276251	-424.364947
M06L	-424.416760	-424.350281	-424.404331	-424.364257	-424.464490
M06L-D3	-424.417618	-424.351182	-424.405256	-424.365066	-424.465287
MN12-L	-424.025216	-423.959981	-424.020356	-423.977854	-424.077347
MN15-L	-424.069799	-423.997923	-424.059128	-424.018400	-424.124094
TPSS	-424.540327	-424.482185	-424.529198	-424.497788	-424.590413
TPSS-D3BJ	-424.563116	-424.506565	-424.552621	-424.524123	-424.615504
B3LYP	-424.492992	-424.427893	-424.483218	-424.440114	-424.539089
B3LYP-D3BJ	-424.523034	-424.460049	-424.514012	-424.475074	-424.572398
PBE0	-423.977772	-423.919009	-423.969336	-423.933353	-424.034460
PBE0-D3BJ	-423.993614	-423.935958	-423.985667	-423.951603	-424.051846
B3PW91	-424.317655	-424.258126	-424.308656	-424.272128	-424.371086
B3PW91-D3	-424.337198	-424.277894	-424.327988	-424.293179	-424.391643
X3LYP	-424.295714	-424.230874	-424.286218	-424.243006	-424.342464
B1LYP	-424.266758	-424.198593	-424.256391	-424.210689	-424.312269

Continued on next page

Table S44 (continued)

XC	1	TS_{enol}	2	TS_{conia}	3
mPW1PW91	-424.370179	-424.309995	-424.361258	-424.324303	-424.425531
B972	-424.326242	-424.262253	-424.316197	-424.276086	-424.378301
MN15	-423.970420	-423.907130	-423.964203	-423.920803	-424.021334
M08HX	-424.355823	-424.287669	-424.348358	-424.303350	-424.406688
M06	-424.192544	-424.127807	-424.181987	-424.139357	-424.240209
M06-D3	-424.195096	-424.130519	-424.184676	-424.142013	-424.242728
M06HF	-424.357842	-424.299746	-424.354754	-424.312118	-424.408993
M06HF-D3	-424.359336	-424.301336	-424.356352	-424.313589	-424.410425
M062X	-424.296392	-424.233270	-424.288990	-424.244217	-424.344329
M062X-D3	-424.297129	-424.234037	-424.289783	-424.244906	-424.345006
M05	-424.175742	-424.106762	-424.161654	-424.115667	-424.223449
M05-D3	-424.182585	-424.113748	-424.168499	-424.122899	-424.230494
M052X	-424.423555	-424.361872	-424.416688	-424.375776	-424.476191
M052X-D3	-424.425247	-424.363674	-424.418491	-424.377462	-424.477822
TPSSH	-424.494533	-424.433548	-424.483416	-424.449036	-424.546211
CAM-B3LYP	-424.273663	-424.208115	-424.263706	-424.217662	-424.323022
CAM-B3LYP-D3BJ	-424.287766	-424.223220	-424.278352	-424.233916	-424.338494
wB97XD	-424.347742	-424.280595	-424.337241	-424.293216	-424.400493
B2PLYP	-424.093788	-424.026278	-424.082795	-424.040525	-424.140052
mPW2PLYP	-424.094305	-424.026029	-424.083554	-424.039946	-424.141571
DSDPBEP86	-423.649013	-423.581573	-423.638115	-423.597351	-423.700004

5.6 cc-pVDZ

Table S45: XC/cc-pVDZ single-point energies on CCSD/6-31G(d,p) optimized geometries for the reaction profile of **hex-5-ynal** (1a).

XC	1	TS_{enol}	2	TS_{conia}	3
CCSD(T)	-308.0874483	-307.9784291	-308.0747491	-308.0112212	-308.1013997
PBE	-308.2110748	-308.1188711	-308.1980088	-308.1580032	-308.2366933
PBE-D3BJ	-308.2239635	-308.1314442	-308.2100927	-308.1718811	-308.2497332
PW91	-308.4768431	-308.3842063	-308.4638472	-308.4238757	-308.5019101
M06L	-308.5740713	-308.4683669	-308.5564133	-308.5020628	-308.5937154
M06L-D3	-308.5744422	-308.4687568	-308.5568220	-308.5023728	-308.5940624
MN12-L	-308.3079091	-308.2022964	-308.2978560	-308.2383514	-308.3262361
MN15-L	-308.3321880	-308.2239559	-308.3164250	-308.2596059	-308.3538982
TPSS	-308.6619145	-308.5635412	-308.6467186	-308.6024905	-308.6861124
TPSS-D3BJ	-308.6780419	-308.5792526	-308.6618027	-308.6198033	-308.7023991

Continued on next page

Table S45 (continued)

XC	1	TS_{enol}	2	TS_{conia}	3
B3LYP	-308.6084347	-308.5043892	-308.5952882	-308.5383334	-308.6272045
B3LYP-D3BJ	-308.6299700	-308.5254126	-308.6155060	-308.5617983	-308.6491425
PBE0	-308.2435385	-308.1421793	-308.2304528	-308.1805473	-308.2701891
PBE0-D3BJ	-308.2546698	-308.1530203	-308.2408669	-308.1924055	-308.2813897
B3PW91	-308.4911492	-308.3902077	-308.4783533	-308.4273892	-308.5158797
B3PW91-D3	-308.5029302	-308.4009178	-308.4882414	-308.4387256	-308.5270949
X3LYP	-308.4614805	-308.3571567	-308.4483293	-308.3914627	-308.4804942
B1LYP	-308.4424155	-308.3360020	-308.4289878	-308.3690107	-308.4604601
mPW1PW91	-308.5323705	-308.4300292	-308.5193202	-308.4679217	-308.5581061
B972	-308.5046605	-308.4011822	-308.4911838	-308.4362423	-308.5284218
MN15	-308.2263846	-308.1257134	-308.2158971	-308.1591573	-308.2475407
M08HX	-308.5281830	-308.4195406	-308.5152601	-308.4550024	-308.5470598
M06	-308.3801213	-308.2773608	-308.3647565	-308.3104216	-308.3984785
M06-D3	-308.3814793	-308.2786167	-308.3659735	-308.3116005	-308.3997684
M06HF	-308.5188706	-308.4153994	-308.5130676	-308.4519672	-308.5373502
M06HF-D3	-308.5195906	-308.4161055	-308.5137733	-308.4525861	-308.5380439
M062X	-308.4798066	-308.3747563	-308.4680979	-308.4082792	-308.4973211
M062X-D3	-308.4801148	-308.3750846	-308.4684486	-308.4085348	-308.4976059
M05	-308.3833767	-308.2799723	-308.3648330	-308.3096616	-308.4070671
M05-D3	-308.3873620	-308.2836216	-308.3682609	-308.3134765	-308.4108824
M052X	-308.5683496	-308.4636779	-308.5576874	-308.4998069	-308.5879770
M052X-D3	-308.5691844	-308.4644831	-308.5584865	-308.5005275	-308.5887811
TPSSH	-308.6303879	-308.5288496	-308.6153715	-308.5674053	-308.6550635
CAM-B3LYP	-308.4386796	-308.3329938	-308.4252368	-308.3655331	-308.4591601
CAM-B3LYP-D3BJ	-308.4485843	-308.3426309	-308.4345075	-308.3759588	-308.4690616
wB97XD	-308.5090820	-308.4029224	-308.4938169	-308.4355699	-308.5322595
B2PLYP	-308.2825199	-308.1761816	-308.2672317	-308.2089883	-308.2988375
mPW2PLYP	-308.2854079	-308.1776889	-308.2702096	-308.2107714	-308.3022422
DSDPBEP86	-307.9372589	-307.8293668	-307.9206582	-307.8628740	-307.9556151

Table S46: XC/cc-pVDZ single-point energies on CCSD/6-31G(d,p) optimized geometries for the reaction profile of **hept-6-ynal** (1b).

XC	1	TS_{enol}	2	TS_{conia}	3
CCSD(T)	-347.328943	-347.217226	-347.315776	-347.276947	-347.378884
PBE	-347.466924	-347.372733	-347.453617	-347.436725	-347.524469
PBE-D3BJ	-347.482622	-347.388412	-347.469166	-347.454960	-347.542097
PW91	-347.769553	-347.674946	-347.756355	-347.739795	-347.826914

Continued on next page

Table S46 (continued)

XC	1	TS_{enol}	2	TS_{conia}	3
M06L	-347.881176	-347.774156	-347.864130	-347.833647	-347.933075
M06L-D3	-347.881739	-347.774751	-347.864733	-347.834168	-347.933603
MN12-L	-347.572191	-347.464642	-347.562918	-347.528205	-347.626685
MN15-L	-347.604397	-347.494305	-347.589577	-347.556176	-347.661583
TPSS	-347.982111	-347.881337	-347.966228	-347.944716	-348.036512
TPSS-D3BJ	-348.001671	-347.900890	-347.985631	-347.967508	-348.058543
B3LYP	-347.921510	-347.814873	-347.907627	-347.875143	-347.972485
B3LYP-D3BJ	-347.947545	-347.840884	-347.933470	-347.905760	-348.002024
PBE0	-347.508895	-347.405041	-347.495360	-347.469453	-347.569400
PBE0-D3BJ	-347.522420	-347.418586	-347.508796	-347.485149	-347.584583
B3PW91	-347.791269	-347.687778	-347.777665	-347.750694	-347.848601
B3PW91-D3	-347.806163	-347.701794	-347.791546	-347.766598	-347.864860
X3LYP	-347.752716	-347.645814	-347.738950	-347.706665	-347.804430
B1LYP	-347.727921	-347.618782	-347.713687	-347.678320	-347.778297
mPW1PW91	-347.837800	-347.732891	-347.824097	-347.796855	-347.897030
B972	-347.805153	-347.699051	-347.790806	-347.759769	-347.860974
MN15	-347.483710	-347.380824	-347.473644	-347.439812	-347.538578
M08HX	-347.834231	-347.723548	-347.821651	-347.786311	-347.889396
M06	-347.658211	-347.553652	-347.643176	-347.612244	-347.711114
M06-D3	-347.660006	-347.555505	-347.644931	-347.614069	-347.712909
M06HF	-347.818472	-347.711742	-347.812078	-347.777008	-347.874573
M06HF-D3	-347.819483	-347.712809	-347.813107	-347.778000	-347.875559
M062X	-347.772837	-347.665565	-347.761314	-347.726377	-347.825880
M062X-D3	-347.773316	-347.666071	-347.761834	-347.726813	-347.826322
M05	-347.662222	-347.556540	-347.643322	-347.609372	-347.715516
M05-D3	-347.667350	-347.561451	-347.648178	-347.614780	-347.720977
M052X	-347.875067	-347.767858	-347.864318	-347.832682	-347.932741
M052X-D3	-347.876221	-347.769072	-347.865482	-347.833825	-347.933872
TPSSH	-347.947975	-347.843877	-347.932236	-347.907354	-348.003760
CAM-B3LYP	-347.726536	-347.618125	-347.712524	-347.677393	-347.780967
CAM-B3LYP-D3BJ	-347.738527	-347.630186	-347.724500	-347.691301	-347.794416
wB97XD	-347.812040	-347.703408	-347.796706	-347.762558	-347.869081
B2PLYP	-347.547608	-347.438669	-347.531682	-347.498747	-347.598455
mPW2PLYP	-347.551611	-347.441260	-347.535890	-347.501904	-347.603513
DSDPBEP86	-347.158222	-347.047903	-347.141488	-347.109446	-347.213319

Table S47: XC/cc-pVDZ single-point energies on CCSD/6-31G(d,p) optimized geometries for the reaction profile of **hex-5-ynal** (1a) + $\mathbf{H_2O}$.

XC	1	TS_{enol}	2	TS_{conia}	3
CCSD(T)	-384.4419462	-384.3711400	-384.4296563	-384.3639606	-384.4542281
PBE	-384.5635657	-384.5196780	-384.5510268	-384.5082348	-384.5869103
PBE-D3BJ	-384.5798582	-384.5349227	-384.5662819	-384.5248108	-384.6025161
PW91	-384.8873228	-384.8436333	-384.8748483	-384.8320093	-384.9100161
M06L	-385.0050758	-384.9396545	-384.9873042	-384.9298267	-385.0215506
M06L-D3	-385.0056986	-384.9402660	-384.9879923	-384.9303953	-385.0221382
MN12-L	-384.6774522	-384.6116462	-384.6675224	-384.6035895	-384.6912773
MN15-L	-384.6951326	-384.6205058	-384.6780039	-384.6171222	-384.7118788
TPSS	-385.1014214	-385.0499589	-385.0867096	-385.0404707	-385.1235872
TPSS-D3BJ	-385.1218365	-385.0688937	-385.1057635	-385.0611528	-385.1430812
B3LYP	-385.0454880	-384.9880776	-385.0322389	-384.9738115	-385.0621696
B3LYP-D3BJ	-385.0723166	-385.0133331	-385.0573852	-385.0014226	-385.0880968
PBE0	-384.5995851	-384.5458453	-384.5868632	-384.5343234	-384.6238282
PBE0-D3BJ	-384.6137827	-384.5589143	-384.6001170	-384.5486060	-384.6373217
B3PW91	-384.8994075	-384.8466927	-384.8871968	-384.8342838	-384.9223013
B3PW91-D3	-384.9176380	-384.8608975	-384.9027880	-384.8503171	-384.9380829
X3LYP	-384.8699651	-384.8123091	-384.8566324	-384.7980793	-384.8866840
B1LYP	-384.8482372	-384.7876868	-384.8345576	-384.7732764	-384.8641504
mPW1PW91	-384.9494060	-384.8949738	-384.9368140	-384.8831323	-384.9729931
B972	-384.9154550	-384.8576669	-384.9021842	-384.8454871	-384.9373103
MN15	-384.5872152	-384.5279254	-384.5765512	-384.5163347	-384.6045253
M08HX	-384.9390228	-384.8724914	-384.9263282	-384.8618749	-384.9536112
M06	-384.7853506	-384.7241008	-384.7696820	-384.7119898	-384.8004734
M06-D3	-384.7876120	-384.7258134	-384.7717836	-384.7139695	-384.8024230
M06HF	-384.9256877	-384.8707581	-384.9196500	-384.8558376	-384.9400147
M06HF-D3	-384.9268763	-384.8717710	-384.9208837	-384.8569125	-384.9411049
M062X	-384.8882868	-384.8276709	-384.8763309	-384.8132264	-384.9016940
M062X-D3	-384.8888099	-384.8282027	-384.8769129	-384.8137048	-384.9021891
M05	-384.7866613	-384.7222161	-384.7672928	-384.7092937	-384.8077944
M05-D3	-384.7928244	-384.7271302	-384.7727924	-384.7148017	-384.8132080
M052X	-384.9958907	-384.9378046	-384.9848100	-384.9241756	-385.0121086
M052X-D3	-384.9972698	-384.9389431	-384.9862088	-384.9254164	-385.0133572
TPSSH	-385.0627823	-385.0079056	-385.0481813	-384.9981958	-385.0853479
CAM-B3LYP	-384.8494422	-384.7910252	-384.8357867	-384.7742051	-384.8674659
CAM-B3LYP-D3BJ	-384.8621553	-384.8025455	-384.8476527	-384.7868565	-384.8794597
wB97XD	-384.9276637	-384.8639327	-384.9121973	-384.8513872	-384.9474402

Continued on next page

Table S47 (continued)

XC	1	TS_{enol}	2	TS_{conia}	3
B2PLYP	-384.652873	-384.590541	-384.637335	-384.577656	-384.666741
mPW2PLYP	-384.656730	-384.593052	-384.641148	-384.580055	-384.670899
DSDPBEP86	-384.234262	-384.169089	-384.217454	-384.157692	-384.249542

Table S48: XC/cc-pVDZ single-point energies on CCSD/6-31G(d,p) optimized geometries for the reaction profile of **hept-6-ynal** (1b) + $\mathbf{H_2O}$.

XC	1	TS_{enol}	2	TS_{conia}	3
CCSD(T)	-423.682419	-423.612979	-423.672073	-423.628991	-423.732198
PBE	-423.816082	-423.775134	-423.809140	-423.786574	-423.875614
PBE-D3BJ	-423.834373	-423.794700	-423.827901	-423.807644	-423.895699
PW91	-424.176620	-424.136160	-424.169930	-424.147528	-424.235923
M06L	-424.308946	-424.248702	-424.297213	-424.261354	-424.361481
M06L-D3	-424.309804	-424.249603	-424.298137	-424.262164	-424.362278
MN12-L	-423.937508	-423.878477	-423.933898	-423.893443	-423.992464
MN15-L	-423.962456	-423.895649	-423.952387	-423.914110	-424.019849
TPSS	-424.418979	-424.369211	-424.408494	-424.382194	-424.475087
TPSS-D3BJ	-424.441768	-424.393590	-424.431916	-424.408530	-424.500178
B3LYP	-424.355871	-424.299842	-424.346903	-424.310113	-424.408555
B3LYP-D3BJ	-424.385912	-424.331998	-424.377697	-424.345073	-424.441865
PBE0	-423.861910	-423.810617	-423.853836	-423.822736	-423.924040
PBE0-D3BJ	-423.877752	-423.827565	-423.870166	-423.840986	-423.941426
B3PW91	-424.197113	-424.145306	-424.188512	-424.157026	-424.256182
B3PW91-D3	-424.216656	-424.165074	-424.207844	-424.178077	-424.276739
X3LYP	-424.158293	-424.102558	-424.149627	-424.112788	-424.211675
B1LYP	-424.131088	-424.071806	-424.121515	-424.082068	-424.183099
mPW1PW91	-424.252145	-424.199414	-424.243583	-424.211516	-424.312987
B972	-424.213487	-424.156700	-424.203731	-424.168526	-424.270939
MN15	-423.840581	-423.786662	-423.836005	-423.796921	-423.896458
M08HX	-424.240780	-424.180460	-424.234212	-424.192968	-424.296798
M06	-424.059928	-424.003422	-424.050310	-424.013638	-424.113644
M06-D3	-424.062480	-424.006135	-424.052999	-424.016294	-424.116163
M06HF	-424.221607	-424.170991	-424.219818	-424.180225	-424.277922
M06HF-D3	-424.223102	-424.172581	-424.221416	-424.181697	-424.279354
M062X	-424.177453	-424.122186	-424.171057	-424.131068	-424.231000
M062X-D3	-424.178190	-424.122954	-424.171850	-424.131757	-424.231677
M05	-424.062294	-424.000848	-424.048125	-424.008905	-424.117037
M05-D3	-424.069137	-424.007834	-424.054971	-424.016137	-424.124081

Continued on next page

Table S48 (continued)

XC	1	TS_{enol}	2	TS_{conia}	3
M052X	-424.299135	-424.245205	-424.293032	-424.256643	-424.357537
M052X-D3	-424.300827	-424.247007	-424.294835	-424.258329	-424.359168
TPSSH	-424.377778	-424.324522	-424.367158	-424.337615	-424.435157
CAM-B3LYP	-424.134350	-424.078173	-424.125397	-424.085510	-424.190319
CAM-B3LYP-D3BJ	-424.148453	-424.093278	-424.140044	-424.101765	-424.205790
wB97XD	-424.226912	-424.167563	-424.217029	-424.178189	-424.285328
B2PLYP	-423.915121	-423.855006	-423.903894	-423.866939	-423.967385
mPW2PLYP	-423.919890	-423.858967	-423.908977	-423.870719	-423.973137
DSDPBEP86	-423.451848	-423.390651	-423.440159	-423.403861	-423.508155

5.7 aug-cc-pVDZ

Table S49: XC/aug-cc-pVDZ single-point energies on CCSD/6-31G(d,p) optimized geometries for the reaction profile of **hex-5-ynal** (1a).

XC	1	TS_{enol}	2	TS_{conia}	3
CCSD(T)	-308.0874483	-307.9784291	-308.0747491	-308.0112212	-308.1013997
PBE	-308.2354808	-308.1418961	-308.2243394	-308.1817124	-308.2606117
PBE-D3BJ	-308.2483695	-308.1544692	-308.2364233	-308.1955902	-308.2736515
PW91	-308.5022471	-308.4081936	-308.4911711	-308.4485099	-308.5268311
M06L	-308.5886718	-308.4823116	-308.5726754	-308.5169687	-308.6081083
M06L-D3	-308.5890427	-308.4827014	-308.5730842	-308.5172787	-308.6084553
MN12-L	-308.3230209	-308.2171804	-308.3144719	-308.2540735	-308.3410254
MN15-L	-308.3513637	-308.2431309	-308.3373784	-308.2797682	-308.3731778
TPSS	-308.6828398	-308.5832167	-308.6693441	-308.6230334	-308.7065267
TPSS-D3BJ	-308.6989672	-308.5989281	-308.6844282	-308.6403461	-308.7228134
B3LYP	-308.6324929	-308.5273567	-308.6212950	-308.5616612	-308.6507458
B3LYP-D3BJ	-308.6540282	-308.5483802	-308.6415127	-308.5851261	-308.6726838
PBE0	-308.2632356	-308.1609759	-308.2518339	-308.2001068	-308.2895264
PBE0-D3BJ	-308.2743669	-308.1718169	-308.2622481	-308.2119650	-308.3007270
B3PW91	-308.5107791	-308.4088575	-308.4996601	-308.4468306	-308.5351478
B3PW91-D3	-308.5225601	-308.4195676	-308.5095482	-308.4581670	-308.5463630
X3LYP	-308.4858937	-308.3805005	-308.4747161	-308.4151034	-308.5043655
B1LYP	-308.4662430	-308.3588295	-308.4547686	-308.3921298	-308.4837589
mPW1PW91	-308.5519856	-308.4487489	-308.5405858	-308.4874119	-308.5773742
B972	-308.5236792	-308.4193130	-308.5118542	-308.4551553	-308.5471151
MN15	-308.2534869	-308.1518251	-308.2448775	-308.1859647	-308.2742214
M08HX	-308.5487964	-308.4396727	-308.5374367	-308.4754729	-308.5672419

Continued on next page

Table S49 (continued)

XC	1	TS_{enol}	2	TS_{conia}	3
M06	-308.4016111	-308.2980459	-308.3881986	-308.3317125	-308.4197226
M06-D3	-308.4029691	-308.2993018	-308.3894157	-308.3328914	-308.4210125
M06HF	-308.5439953	-308.4401775	-308.5392629	-308.4771953	-308.5624584
M06HF-D3	-308.5447153	-308.4408836	-308.5399686	-308.4778143	-308.5631521
M062X	-308.5006888	-308.3949634	-308.4906304	-308.4288868	-308.5178619
M062X-D3	-308.5009970	-308.3952917	-308.4909812	-308.4291424	-308.5181468
M05	-308.4042915	-308.2998229	-308.3872833	-308.3304441	-308.4278456
M05-D3	-308.4082769	-308.3034723	-308.3907112	-308.3342590	-308.4316609
M052X	-308.5900628	-308.4844416	-308.5807712	-308.5209612	-308.6091972
M052X-D3	-308.5908975	-308.4852468	-308.5815703	-308.5216818	-308.6100013
TPSSH	-308.6498877	-308.5472765	-308.6365065	-308.5867062	-308.6741131
CAM-B3LYP	-308.4630973	-308.3562283	-308.4514304	-308.3891160	-308.4830857
CAM-B3LYP-D3BJ	-308.4730020	-308.3658653	-308.4607012	-308.3995418	-308.4929872
wB97XD	-308.5285429	-308.4214286	-308.5149180	-308.4548300	-308.5513711
B2PLYP	-308.3158974	-308.2091507	-308.3027598	-308.2434109	-308.3318971
mPW2PLYP	-308.3180462	-308.2099385	-308.3049690	-308.2443167	-308.3345356
DSDPBEP86	-307.9746687	-307.8667552	-307.9600751	-307.9022545	-307.9928726

Table S50: XC/aug-cc-pVDZ single-point energies on CCSD/6-31G(d,p) optimized geometries for the reaction profile of **hept-6-ynal** (1b).

XC	1	TS_{enol}	2	TS_{conia}	3
CCSD(T)	-347.328943	-347.217226	-347.315776	-347.276947	-347.378884
PBE	-347.494425	-347.398738	-347.482756	-347.463084	-347.551436
PBE-D3BJ	-347.510123	-347.414417	-347.498305	-347.481319	-347.569064
PW91	-347.798196	-347.702061	-347.786638	-347.767248	-347.855019
M06L	-347.897576	-347.789840	-347.882156	-347.849937	-347.949077
M06L-D3	-347.898139	-347.790435	-347.882758	-347.850459	-347.949606
MN12-L	-347.588818	-347.480946	-347.580926	-347.544991	-347.642993
MN15-L	-347.625667	-347.515481	-347.612352	-347.577828	-347.682974
TPSS	-348.005556	-347.903422	-347.991173	-347.967445	-348.059434
TPSS-D3BJ	-348.025116	-347.922975	-348.010575	-347.990237	-348.081465
B3LYP	-347.948674	-347.840770	-347.936433	-347.901140	-347.999035
B3LYP-D3BJ	-347.974708	-347.866781	-347.962276	-347.931757	-348.028574
PBE0	-347.530902	-347.426052	-347.518863	-347.490904	-347.591121
PBE0-D3BJ	-347.544427	-347.439597	-347.532299	-347.506600	-347.606304
B3PW91	-347.813312	-347.708732	-347.801202	-347.772168	-347.870365
B3PW91-D3	-347.828206	-347.722749	-347.815083	-347.788072	-347.886623

Continued on next page

Table S50 (continued)

XC	1	TS_{enol}	2	TS_{conia}	3
X3LYP	-347.780245	-347.672098	-347.768136	-347.732976	-347.831306
B1LYP	-347.754783	-347.644456	-347.742194	-347.704021	-347.804515
mPW1PW91	-347.859744	-347.753828	-347.847515	-347.818264	-347.918685
B972	-347.826415	-347.719318	-347.813546	-347.780478	-347.881898
MN15	-347.514823	-347.410941	-347.506407	-347.470635	-347.570012
M08HX	-347.857187	-347.745919	-347.845984	-347.808839	-347.912038
M06	-347.682780	-347.577305	-347.669494	-347.636331	-347.735718
M06-D3	-347.684575	-347.579157	-347.671248	-347.638156	-347.737513
M06HF	-347.846792	-347.739746	-347.841425	-347.805446	-347.903507
M06HF-D3	-347.847804	-347.740813	-347.842454	-347.806438	-347.904493
M062X	-347.796527	-347.688511	-347.786508	-347.749499	-347.849492
M062X-D3	-347.797007	-347.689018	-347.787028	-347.749934	-347.849934
M05	-347.685567	-347.578668	-347.667995	-347.631849	-347.738439
M05-D3	-347.690696	-347.583579	-347.672851	-347.637258	-347.743900
M052X	-347.899536	-347.791337	-347.890057	-347.856471	-347.957125
M052X-D3	-347.900690	-347.792550	-347.891221	-347.857614	-347.958256
TPSSH	-347.969758	-347.864482	-347.955480	-347.928597	-348.025116
CAM-B3LYP	-347.753999	-347.644230	-347.741491	-347.703610	-347.807900
CAM-B3LYP-D3BJ	-347.765990	-347.656291	-347.753467	-347.717518	-347.821349
wB97XD	-347.833924	-347.724213	-347.820042	-347.783810	-347.890676
B2PLYP	-347.585208	-347.475870	-347.571356	-347.537048	-347.635896
mPW2PLYP	-347.588323	-347.477577	-347.574630	-347.539164	-347.640006
DSDPBEP86	-347.200348	-347.090123	-347.185696	-347.153271	-347.255646

Table S51: XC/aug-cc-pVDZ single-point energies on CCSD/6-31G(d,p) optimized geometries for the reaction profile of **hex-5-ynal** (1a) + $\mathbf{H_2O}$.

XC	1	TS_{enol}	2	TS_{conia}	3
CCSD(T)	-384.4419462	-384.3711400	-384.4296563	-384.3639606	-384.4542281
PBE	-384.6039779	-384.5553334	-384.5943326	-384.5488995	-384.6291331
PBE-D3BJ	-384.6202704	-384.5705781	-384.6095877	-384.5654755	-384.6447389
PW91	-384.9290321	-384.8805880	-384.9194885	-384.8739451	-384.9536266
M06L	-385.0309675	-384.9623163	-385.0152538	-384.9562598	-385.0483625
M06L-D3	-385.0315903	-384.9629277	-385.0159419	-384.9568283	-385.0489501
MN12-L	-384.7037777	-384.6354019	-384.6955258	-384.6308106	-384.7182012
MN15-L	-384.7264054	-384.6496783	-384.7115255	-384.6500709	-384.7445871
TPSS	-385.1366746	-385.0811412	-385.1246155	-385.0761639	-385.1603694
TPSS-D3BJ	-385.1570897	-385.1000760	-385.1436694	-385.0968460	-385.1798634

Continued on next page

Table S51 (continued)

XC	1	TS_{enol}	2	TS_{conia}	3
B3LYP	-385.0847314	-385.0231957	-385.0743276	-385.0131921	-385.1031278
B3LYP-D3BJ	-385.1115600	-385.0484511	-385.0994739	-385.0408032	-385.1290550
PBE0	-384.6327824	-384.5753563	-384.6226109	-384.5681462	-384.6584369
PBE0-D3BJ	-384.6469800	-384.5884253	-384.6358648	-384.5824289	-384.6719304
B3PW91	-384.9325471	-384.8759939	-384.9227373	-384.8679317	-384.9567828
B3PW91-D3	-384.9507776	-384.8901988	-384.9383284	-384.8839650	-384.9725643
X3LYP	-384.9096946	-384.8479342	-384.8992805	-384.8379336	-384.9281578
B1LYP	-384.8870447	-384.8225483	-384.8762315	-384.8122530	-384.9046517
mPW1PW91	-384.9823839	-384.9243530	-384.9722609	-384.9167391	-385.0073390
B972	-384.9474997	-384.8862744	-384.9367602	-384.8782395	-384.9708276
MN15	-384.6310373	-384.5664527	-384.6223672	-384.5600102	-384.6490668
M08HX	-384.9741449	-384.9037898	-384.9636461	-384.8973541	-384.9896632
M06	-384.8208723	-384.7556695	-384.8074720	-384.7477930	-384.8373243
M06-D3	-384.8231336	-384.7573821	-384.8095736	-384.7497727	-384.8392739
M06HF	-384.9663677	-384.9076870	-384.9624936	-384.8970744	-384.9819321
M06HF-D3	-384.9675562	-384.9086998	-384.9637272	-384.8981493	-384.9830223
M062X	-384.9231016	-384.8584958	-384.9131789	-384.8480451	-384.9374128
M062X-D3	-384.9236248	-384.8590277	-384.9137610	-384.8485235	-384.9379079
M05	-384.8218038	-384.7534670	-384.8051879	-384.7452431	-384.8449269
M05-D3	-384.8279669	-384.7583811	-384.8106876	-384.7507511	-384.8503406
M052X	-385.0321686	-384.9697232	-385.0231802	-384.9604092	-385.0493131
M052X-D3	-385.0335477	-384.9708617	-385.0245790	-384.9616500	-385.0505617
TPSSH	-385.0958148	-385.0372072	-385.0837726	-385.0317955	-385.1197975
CAM-B3LYP	-384.8895239	-384.8265694	-384.8785064	-384.8142198	-384.9090869
CAM-B3LYP-D3BJ	-384.9022370	-384.8380898	-384.8903724	-384.8268712	-384.9210807
wB97XD	-384.9607703	-384.8928690	-384.9474684	-384.8846937	-384.9816008
B2PLYP	-384.7054916	-384.6397072	-384.6930100	-384.6318445	-384.7209664
mPW2PLYP	-384.7081661	-384.6411627	-384.6956712	-384.6329829	-384.7239588
DSDPBEP86	-384.2922560	-384.2240165	-384.2783070	-384.2179376	-384.3089235

Table S52: XC/aug-cc-pVDZ single-point energies on CCSD/6-31G(d,p) optimized geometries for the reaction profile of **hept-6-ynal** (1b) + $\mathbf{H_2O}$.

XC	1	TS_{enol}	2	TS_{conia}	3
CCSD(T)	-423.682419	-423.612979	-423.672073	-423.628991	-423.732198
PBE	-423.862867	-423.813565	-423.854839	-423.829554	-423.920385
PBE-D3BJ	-423.881157	-423.833131	-423.873600	-423.850624	-423.940470
PW91	-424.224941	-424.176005	-424.217111	-424.191949	-424.282233

Continued on next page

Table S52 (continued)

XC	1	TS_{enol}	2	TS_{conia}	3
M06L	-424.338397	-424.273007	-424.326605	-424.288984	-424.389637
M06L-D3	-424.339255	-424.273908	-424.327529	-424.289794	-424.390434
MN12-L	-423.966905	-423.903468	-423.963021	-423.921573	-424.020767
MN15-L	-423.997852	-423.926686	-423.987448	-423.948238	-424.054292
TPSS	-424.459683	-424.402601	-424.448294	-424.419810	-424.513983
TPSS-D3BJ	-424.482472	-424.426981	-424.471717	-424.446146	-424.539074
B3LYP	-424.401338	-424.337626	-424.391441	-424.351840	-424.452087
B3LYP-D3BJ	-424.431379	-424.369782	-424.422236	-424.386801	-424.485397
PBE0	-423.900036	-423.842159	-423.891314	-423.858181	-423.960669
PBE0-D3BJ	-423.915878	-423.859108	-423.907644	-423.876431	-423.978055
B3PW91	-424.235200	-424.176725	-424.225924	-424.192453	-424.292797
B3PW91-D3	-424.254743	-424.196493	-424.245256	-424.213504	-424.313354
X3LYP	-424.204321	-424.140851	-424.194716	-424.154984	-424.255717
B1LYP	-424.176024	-424.109225	-424.165561	-424.123309	-424.226107
mPW1PW91	-424.289990	-424.230781	-424.280798	-424.246794	-424.349394
B972	-424.250364	-424.187211	-424.240000	-424.202804	-424.306356
MN15	-423.890551	-423.829405	-423.885324	-423.844366	-423.945155
M08HX	-424.280424	-424.213940	-424.273361	-424.230243	-424.334968
M06	-424.100822	-424.037871	-424.090805	-424.051906	-424.153288
M06-D3	-424.103375	-424.040584	-424.093494	-424.054562	-424.155807
M06HF	-424.267761	-424.211303	-424.265409	-424.224436	-424.323266
M06HF-D3	-424.269256	-424.212894	-424.267006	-424.225907	-424.324699
M062X	-424.217138	-424.155693	-424.210308	-424.168166	-424.269367
M062X-D3	-424.217875	-424.156461	-424.211101	-424.168855	-424.270044
M05	-424.102825	-424.034198	-424.087892	-424.046154	-424.155884
M05-D3	-424.109668	-424.041184	-424.094738	-424.053386	-424.162928
M052X	-424.340462	-424.279843	-424.333702	-424.295253	-424.397465
M052X-D3	-424.342154	-424.281646	-424.335505	-424.296939	-424.399095
TPSSH	-424.415806	-424.355811	-424.404445	-424.372908	-424.471560
CAM-B3LYP	-424.180440	-424.116344	-424.170476	-424.127860	-424.234506
CAM-B3LYP-D3BJ	-424.194543	-424.131449	-424.185123	-424.144114	-424.249978
wB97XD	-424.264692	-424.198714	-424.254238	-424.213250	-424.321567
B2PLYP	-423.974735	-423.908418	-423.963351	-423.924722	-424.025472
mPW2PLYP	-423.978216	-423.911111	-423.967110	-423.927074	-424.029905
DSDPBEP86	-423.516904	-423.450611	-423.505438	-423.468310	-423.572062

5.8 cc-pVTZ

Table S53: XC/cc-pVTZ single-point energies on CCSD/6-31G(d,p) optimized geometries for the reaction profile of **hex-5-ynal** (1a).

XC	1	TS_{enol}	2	TS_{conia}	3
CCSD(T)	-308.0874483	-307.9784291	-308.0747491	-308.0112212	-308.1013997
PBE	-308.3076590	-308.2136499	-308.2970532	-308.2512966	-308.3286795
PBE-D3BJ	-308.3205477	-308.2262231	-308.3091371	-308.2651745	-308.3417193
PW91	-308.5775667	-308.4829400	-308.5670224	-308.5209192	-308.5978320
M06L	-308.6541162	-308.5464827	-308.6372954	-308.5793463	-308.6700791
M06L-D3	-308.6544871	-308.5468726	-308.6377042	-308.5796563	-308.6704262
MN12-L	-308.3722449	-308.2660819	-308.3636424	-308.3021525	-308.3887125
MN15-L	-308.4103747	-308.3023555	-308.3966232	-308.3385545	-308.4306147
TPSS	-308.7531348	-308.6529670	-308.7402998	-308.6908082	-308.7729704
TPSS-D3BJ	-308.7692622	-308.6686783	-308.7553839	-308.7081210	-308.7892571
B3LYP	-308.7123794	-308.6063583	-308.7014144	-308.6380645	-308.7263789
B3LYP-D3BJ	-308.7339148	-308.6273817	-308.7216321	-308.6615294	-308.7483169
PBE0	-308.3317151	-308.2291984	-308.3210761	-308.2664222	-308.3543682
PBE0-D3BJ	-308.3428464	-308.2400394	-308.3314902	-308.2782804	-308.3655688
B3PW91	-308.5833392	-308.4809630	-308.5729115	-308.5168197	-308.6038345
B3PW91-D3	-308.5951203	-308.4916732	-308.5827996	-308.5281562	-308.6150497
X3LYP	-308.5656030	-308.4593149	-308.5546396	-308.4913404	-308.5798286
B1LYP	-308.5451404	-308.4368356	-308.5338712	-308.4675902	-308.5585078
mPW1PW91	-308.6224937	-308.5188646	-308.6118437	-308.5555272	-308.6441279
B972	-308.5905133	-308.4859203	-308.5794491	-308.5198234	-308.6104422
MN15	-308.3265901	-308.2231557	-308.3175338	-308.2554718	-308.3431375
M08HX	-308.6243425	-308.5137386	-308.6131792	-308.5485047	-308.6382887
M06	-308.4807960	-308.3753115	-308.4667806	-308.4056784	-308.4931570
M06-D3	-308.4821540	-308.3765674	-308.4679977	-308.4068573	-308.4944469
M06HF	-308.6420456	-308.5358967	-308.6373702	-308.5728294	-308.6554788
M06HF-D3	-308.6427657	-308.5366028	-308.6380760	-308.5734484	-308.6561725
M062X	-308.5727428	-308.4656883	-308.5626380	-308.4983103	-308.5856321
M062X-D3	-308.5730510	-308.4660165	-308.5629888	-308.4985659	-308.5859170
M05	-308.4718764	-308.3671020	-308.4557777	-308.3942946	-308.4893513
M05-D3	-308.4758617	-308.3707513	-308.4592056	-308.3981095	-308.4931666
M052X	-308.6695238	-308.5628453	-308.6605177	-308.5979840	-308.6842156
M052X-D3	-308.6703585	-308.5636505	-308.6613168	-308.5987046	-308.6850197
TPSSH	-308.7191019	-308.6160202	-308.7064419	-308.6535430	-308.7396303
CAM-B3LYP	-308.5439947	-308.4361697	-308.5325629	-308.4664561	-308.5595473
CAM-B3LYP-D3BJ	-308.5538994	-308.4458068	-308.5418337	-308.4768819	-308.5694488
wB97XD	-308.6013393	-308.4934500	-308.5880027	-308.5247642	-308.6201852

Continued on next page

Table S53 (continued)

XC	1	TS_{enol}	2	TS_{conia}	3
B2PLYP	-308.4448086	-308.3379128	-308.4324218	-308.3698786	-308.4572183
mPW2PLYP	-308.4425533	-308.3342425	-308.4301857	-308.3663618	-308.4554706
DSDPBEP86	-308.1336305	-308.0260381	-308.1202483	-308.0595371	-308.1487379

Table S54: XC/cc-pVTZ single-point energies on CCSD/6-31G(d,p) optimized geometries for the reaction profile of **hept-6-ynal** (1b).

XC	1	TS_{enol}	2	TS_{conia}	3
CCSD(T)	-347.3289427	-347.2172255	-347.3157755	-347.2769468	-347.3788838
PBE	-347.5758960	-347.4796929	-347.5646385	-347.5420904	-347.6283059
PBE-D3BJ	-347.5915936	-347.4953719	-347.5801872	-347.5603252	-347.6459346
PW91	-347.8832435	-347.7864400	-347.8720907	-347.8495349	-347.9352248
M06L	-347.9718064	-347.8627649	-347.9552937	-347.9208192	-348.0188377
M06L-D3	-347.9723694	-347.8633602	-347.9558961	-347.9213407	-348.0193664
MN12-L	-347.6447379	-347.5367134	-347.6367139	-347.5997473	-347.6968180
MN15-L	-347.6926667	-347.5827037	-347.6795081	-347.6445252	-347.7478620
TPSS	-348.0848593	-347.9821280	-348.0710098	-348.0444147	-348.1344482
TPSS-D3BJ	-348.1044186	-348.0016808	-348.0904119	-348.0672067	-348.1564791
B3LYP	-348.0390117	-347.9301625	-348.0268693	-347.9881292	-348.0843992
B3LYP-D3BJ	-348.0650459	-347.9561729	-348.0527117	-348.0187465	-348.1139379
PBE0	-347.6081100	-347.5029351	-347.5966813	-347.5660114	-347.6641678
PBE0-D3BJ	-347.6216349	-347.5164799	-347.6101170	-347.5817072	-347.6793504
B3PW91	-347.8951488	-347.7900591	-347.8835718	-347.8515041	-347.9477279
B3PW91-D3	-347.9100431	-347.8040754	-347.8974525	-347.8674080	-347.9639868
X3LYP	-347.8703948	-347.7612905	-347.8583710	-347.8198009	-347.9165091
B1LYP	-347.8440310	-347.7327577	-347.8315157	-347.7899706	-347.8888932
mPW1PW91	-347.9392531	-347.8328940	-347.9276192	-347.8954477	-347.9938719
B972	-347.9016972	-347.7943204	-347.8894470	-347.8535645	-347.9529765
MN15	-347.5980252	-347.4922276	-347.5889776	-347.5505891	-347.6489859
M08HX	-347.9422515	-347.8296417	-347.9312611	-347.8917121	-347.9923796
M06	-347.7724145	-347.6649723	-347.7583595	-347.7207532	-347.8190733
M06-D3	-347.7742095	-347.6668246	-347.7601141	-347.7225781	-347.8208680
M06HF	-347.9587003	-347.8494198	-347.9532866	-347.9151683	-348.0100892
M06HF-D3	-347.9597115	-347.8504864	-347.9543165	-347.9161602	-348.0110753
M062X	-347.8780747	-347.7687132	-347.8678861	-347.8283941	-347.9260909
M062X-D3	-347.8785541	-347.7692196	-347.8684058	-347.8288296	-347.9265327
M05	-347.7611096	-347.6538677	-347.7443155	-347.7034675	-347.8072476
M05-D3	-347.7662385	-347.6587791	-347.7491717	-347.7088762	-347.8127080

Continued on next page

Table S54 (continued)

XC	1	TS_{enol}	2	TS_{conia}	3
M052X	-347.9898520	-347.8805552	-347.9804785	-347.9446076	-348.0425386
M052X-D3	-347.9910062	-347.8817690	-347.9816423	-347.9457511	-348.0436690
TPSSH	-348.0478176	-347.9420225	-348.0341210	-348.0044290	-348.0990111
CAM-B3LYP	-347.8455036	-347.7347274	-347.8330892	-347.7917946	-347.8944129
CAM-B3LYP-D3BJ	-347.8574949	-347.7467887	-347.8450655	-347.8057025	-347.9078620
wB97XD	-347.9158890	-347.8054020	-347.9021657	-347.8628372	-347.9679729
B2PLYP	-347.7314113	-347.6218302	-347.7181095	-347.6807696	-347.7777183
mPW2PLYP	-347.7295218	-347.6184840	-347.7163465	-347.6778794	-347.7768650
DSDPBEP86	-347.3808202	-347.2707889	-347.3671187	-347.3318718	-347.4321795

Table S55: XC/cc-pVTZ single-point energies on CCSD/6-31G(d,p) optimized geometries for the reaction profile of **hex-5-ynal** (1a) + $\mathbf{H_2O}$.

XC	1	TS_{enol}	2	TS_{conia}	3
CCSD(T)	-384.4419462	-384.3711400	-384.4296563	-384.3639606	-384.4542281
PBE	-384.6938891	-384.6451976	-384.6840877	-384.6357614	-384.7133652
PBE-D3BJ	-384.7101817	-384.6604423	-384.6993428	-384.6523375	-384.7289710
PW91	-385.0225659	-384.9738812	-385.0128394	-384.9641243	-385.0412330
M06L	-385.1091214	-385.0398160	-385.0921405	-385.0311540	-385.1220869
M06L-D3	-385.1097442	-385.0404274	-385.0928286	-385.0317226	-385.1226745
MN12-L	-384.7619100	-384.6935784	-384.7532284	-384.6875204	-384.7739444
MN15-L	-384.7985929	-384.7232694	-384.7835746	-384.7217015	-384.8141580
TPSS	-385.2243243	-385.1686524	-385.2123314	-385.1609042	-385.2428414
TPSS-D3BJ	-385.2447394	-385.1875872	-385.2313853	-385.1815863	-385.2623354
B3LYP	-385.1831878	-385.1210067	-385.1724753	-385.1078277	-385.1959621
B3LYP-D3BJ	-385.2100165	-385.1462621	-385.1976216	-385.1354388	-385.2218893
PBE0	-384.7184753	-384.6612701	-384.7084860	-384.6513128	-384.7393423
PBE0-D3BJ	-384.7326729	-384.6743391	-384.7217399	-384.6655954	-384.7528358
B3PW91	-385.0230782	-384.9664042	-385.0134551	-384.9555429	-385.0423360
B3PW91-D3	-385.0413088	-384.9806090	-385.0290463	-384.9715763	-385.0581176
X3LYP	-385.0078633	-384.9454858	-384.9970959	-384.9322904	-385.0206781
B1LYP	-384.9842460	-384.9191311	-384.9731064	-384.9056874	-384.9963555
mPW1PW91	-385.0704250	-385.0123988	-385.0604999	-385.0020337	-385.0905183
B972	-385.0315525	-384.9705390	-385.0210327	-384.9597695	-385.0502418
MN15	-384.7216134	-384.6558357	-384.7122161	-384.6468396	-384.7345028
M08HX	-385.0703449	-384.9987689	-385.0592821	-384.9908460	-385.0804521
M06	-384.9133115	-384.8467627	-384.8988400	-384.8349257	-384.9229459
M06-D3	-384.9155729	-384.8484753	-384.9009416	-384.8369054	-384.9248956

Continued on next page

Table S55 (continued)

XC	1	TS_{enol}	2	TS_{conia}	3
M06HF	-385.0922144	-385.0320123	-385.0876863	-385.0201288	-385.1021269
M06HF-D3	-385.0934030	-385.0330251	-385.0889199	-385.0212037	-385.1032171
M062X	-385.0140297	-384.9484801	-385.0036698	-384.9361178	-385.0231949
M062X-D3	-385.0145529	-384.9490120	-385.0042519	-384.9365961	-385.0236900
M05	-384.9029376	-384.8352572	-384.8865142	-384.8225063	-384.9187271
M05-D3	-384.9091007	-384.8401713	-384.8920139	-384.8280143	-384.9241407
M052X	-385.1315686	-385.0688365	-385.1224290	-385.0570556	-385.1433764
M052X-D3	-385.1329477	-385.0699750	-385.1238279	-385.0582964	-385.1446250
TPSSH	-385.1821988	-385.1235738	-385.1703072	-385.1154451	-385.2012919
CAM-B3LYP	-384.9889763	-384.9254373	-384.9776280	-384.9097994	-385.0027929
CAM-B3LYP-D3BJ	-385.0016893	-384.9369576	-384.9894940	-384.9224508	-385.0147867
wB97XD	-385.0510526	-384.9828532	-385.0375375	-384.9718849	-385.0668904
B2PLYP	-384.8643706	-384.7993071	-384.8520276	-384.7794807	-384.8750111
mPW2PLYP	-384.8615813	-384.7952340	-384.8491684	-384.7760570	-384.8725459
DSDPBEP86	-384.4886943	-384.4219814	-384.4753059	-384.4043059	-384.5012547

Table S56: XC/cc-pVTZ single-point energies on CCSD/6-31G(d,p) optimized geometries for the reaction profile of **hept-6-ynal** (1b) + $\mathbf{H_2O}$.

XC	1	TS_{enol}	2	TS_{conia}	3
CCSD(T)	-423.6824188	-423.6129791	-423.6720732	-423.6289914	-423.7321977
PBE	-423.9603206	-423.9124355	-423.9536525	-423.9259488	-424.0136789
PBE-D3BJ	-423.9786113	-423.9320015	-423.9724136	-423.9470188	-424.0337637
PW91	-424.3264137	-424.2787239	-424.3199384	-424.2921198	-424.3793052
M06L	-424.4243751	-424.3593063	-424.4119981	-424.3724750	-424.4713641
M06L-D3	-424.4252331	-424.3602068	-424.4129224	-424.3732847	-424.4721615
MN12-L	-424.0308822	-423.9683820	-424.0273831	-423.9850807	-424.0828750
MN15-L	-424.0769624	-424.0082380	-424.0675514	-424.0279271	-424.1316963
TPSS	-424.5549035	-424.4988106	-424.5448276	-424.5138253	-424.6052276
TPSS-D3BJ	-424.5776924	-424.5231899	-424.5682502	-424.5401611	-424.6303185
B3LYP	-424.5087081	-424.4456825	-424.4997738	-424.4571634	-424.5548714
B3LYP-D3BJ	-424.5387495	-424.4778381	-424.5305684	-424.4921235	-424.5881812
PBE0	-423.9932015	-423.9364983	-423.9857442	-423.9502149	-424.0499538
PBE0-D3BJ	-424.0090438	-423.9534471	-424.0020746	-423.9684646	-424.0673400
B3PW91	-424.3338055	-424.2761438	-424.3257003	-424.2894820	-424.3871961
B3PW91-D3	-424.3533484	-424.2959118	-424.3450329	-424.3105329	-424.4077530
X3LYP	-424.3113538	-424.2486334	-424.3027155	-424.2600330	-424.3581999
B1LYP	-424.2820617	-424.2159648	-424.2725357	-424.2273308	-424.3276510

Continued on next page

Table S56 (continued)

XC	1	TS_{enol}	2	TS_{conia}	3
mPW1PW91	-424.3858339	-424.3275555	-424.3778559	-424.3412258	-424.4411660
B972	-424.3416530	-424.2796421	-424.3325700	-424.2928297	-424.3936971
MN15	-423.9901406	-423.9284707	-423.9849380	-423.9417086	-424.0409418
M08HX	-424.3852490	-424.3182538	-424.3784781	-424.3337309	-424.4352557
M06	-424.2025057	-424.1392500	-424.1922879	-424.1495652	-424.2491249
M06-D3	-424.2050579	-424.1419626	-424.1949767	-424.1522213	-424.2516439
M06HF	-424.4067361	-424.3489532	-424.4044057	-424.3616691	-424.4570211
M06HF-D3	-424.4082308	-424.3505438	-424.4060032	-424.3631404	-424.4584536
M062X	-424.3166058	-424.2550154	-424.3101570	-424.2658316	-424.3641783
M062X-D3	-424.3173428	-424.2557832	-424.3109501	-424.2665203	-424.3648553
M05	-424.1905834	-424.1235077	-424.1769952	-424.1312320	-424.2372595
M05-D3	-424.1974261	-424.1304944	-424.1838407	-424.1384644	-424.2443036
M052X	-424.4497100	-424.3895659	-424.4435524	-424.4031008	-424.5020732
M052X-D3	-424.4514022	-424.3913685	-424.4453552	-424.4047871	-424.5037039
TPSSH	-424.5097536	-424.4507036	-424.4996516	-424.4656238	-424.5616099
CAM-B3LYP	-424.2890116	-424.2256316	-424.2799607	-424.2343836	-424.3384712
CAM-B3LYP-D3BJ	-424.3031146	-424.2407372	-424.2946069	-424.2506381	-424.3539430
wB97XD	-424.3630205	-424.2976527	-424.3532987	-424.3096233	-424.4155391
B2PLYP	-424.1495189	-424.0849271	-424.1394609	-424.0982170	-424.1962633
mPW2PLYP	-424.1469218	-424.0815086	-424.1371130	-424.0945002	-424.1946521
DSDPBEP86	-423.7337382	-423.6695558	-423.7237785	-423.6842153	-423.7853044

5.9 def2-SVP

Table S57: XC/def2-SVP single-point energies on CCSD/6-31G(d,p) optimized geometries for the reaction profile of **hex-5-ynal** (1a).

XC	1	TS_{enol}	2	TS_{conia}	3
CCSD(T)	-308.0874483	-307.9784291	-308.0747491	-308.0112212	-308.1013997
PBE	-307.9719038	-307.8784796	-307.9588138	-307.9164901	-307.9970219
PBE-D3BJ	-307.9847924	-307.8910527	-307.9708977	-307.9303680	-308.0100618
PW91	-308.2382336	-308.1443669	-308.2251472	-308.1828851	-308.2627972
M06L	-308.3266837	-308.2196560	-308.3091835	-308.2529573	-308.3462603
M06L-D3	-308.3270546	-308.2200459	-308.3095922	-308.2532673	-308.3466073
MN12-L	-308.0393191	-307.9331013	-308.0306327	-307.9686330	-308.0574505
MN15-L	-308.0162757	-307.9070966	-308.0013900	-307.9418188	-308.0371687
TPSS	-308.4206647	-308.3208134	-308.4054182	-308.3587066	-308.4444246
TPSS-D3BJ	-308.4367921	-308.3365248	-308.4205023	-308.3760194	-308.4607113

Continued on next page

Table S57 (continued)

XC	1	TS_{enol}	2	TS_{conia}	3
B3LYP	-308.3686121	-308.2633100	-308.3554154	-308.2960948	-308.3869541
B3LYP-D3BJ	-308.3901475	-308.2843334	-308.3756331	-308.3195597	-308.4088921
PBE0	-308.0008969	-307.8982633	-307.9880376	-307.9357411	-308.0272817
PBE0-D3BJ	-308.0120282	-307.9091043	-307.9984517	-307.9475993	-308.0384823
B3PW91	-308.2503727	-308.1481554	-308.2376932	-308.1844244	-308.2748720
B3PW91-D3	-308.2621538	-308.1588656	-308.2475813	-308.1957608	-308.2860872
X3LYP	-308.2214730	-308.1158867	-308.2082813	-308.1490188	-308.2400425
B1LYP	-308.2010344	-308.0933651	-308.1875880	-308.1252083	-308.2186576
mPW1PW91	-308.2899190	-308.1862965	-308.2770433	-308.2233042	-308.3154327
B972	-308.2603221	-308.1555831	-308.2471007	-308.1897278	-308.2837053
MN15	-307.9502414	-307.8481973	-307.9397385	-307.8803912	-307.9709193
M08HX	-308.2667342	-308.1565636	-308.2540457	-308.1909618	-308.2847765
M06	-308.1444688	-308.0402788	-308.1288564	-308.0728301	-308.1629380
M06-D3	-308.1458268	-308.0415347	-308.1300734	-308.0740090	-308.1642279
M06HF	-308.2604215	-308.1547111	-308.2546216	-308.1895477	-308.2774716
M06HF-D3	-308.2611415	-308.1554172	-308.2553274	-308.1901667	-308.2781653
M062X	-308.2210710	-308.1145126	-308.2094380	-308.1468486	-308.2376763
M062X-D3	-308.2213792	-308.1148409	-308.2097888	-308.1471042	-308.2379611
M05	-308.1354268	-308.0303187	-308.1165681	-308.0594498	-308.1587276
M05-D3	-308.1394122	-308.0339680	-308.1199960	-308.0632647	-308.1625428
M052X	-308.3049938	-308.1984736	-308.2940900	-308.2333222	-308.3239679
M052X-D3	-308.3058285	-308.1992788	-308.2948891	-308.2340427	-308.3247720
TPSSH	-308.3879215	-308.2849114	-308.3729553	-308.3224900	-308.4122423
CAM-B3LYP	-308.1999002	-308.0929370	-308.1863626	-308.1243848	-308.2201013
CAM-B3LYP-D3BJ	-308.2098050	-308.1025741	-308.1956334	-308.1348106	-308.2300028
wB97XD	-308.2676250	-308.1603048	-308.2525541	-308.1922805	-308.2908597
B2PLYP	-308.0363333	-307.9283773	-308.0210822	-307.9597901	-308.0521788
mPW2PLYP	-308.0391247	-307.9298074	-308.0239938	-307.9615239	-308.0554962
DSDPBEP86	-307.6885046	-307.5787848	-307.6720646	-307.6109787	-307.7064244

Table S58: XC/def2-SVP single-point energies on CCSD/6-31G(d,p) optimized geometries for the reaction profile of **hept-6-ynal** (1b).

XC	1	TS_{enol}	2	TS_{conia}	3
CCSD(T)	-347.328943	-347.217226	-347.315776	-347.276947	-347.378884
PBE	-347.198464	-347.103373	-347.185361	-347.167076	-347.255321
PBE-D3BJ	-347.214161	-347.119052	-347.200910	-347.185311	-347.272950
PW91	-347.501727	-347.406203	-347.488664	-347.470682	-347.558339

Continued on next page

Table S58 (continued)

XC	1	TS_{enol}	2	TS_{conia}	3
M06L	-347.603690	-347.495712	-347.586921	-347.555528	-347.655748
M06L-D3	-347.604253	-347.496307	-347.587523	-347.556050	-347.656277
MN12-L	-347.270191	-347.162507	-347.262359	-347.226593	-347.324842
MN15-L	-347.248575	-347.137937	-347.234724	-347.200160	-347.305426
TPSS	-347.711528	-347.609605	-347.695777	-347.672648	-347.765271
TPSS-D3BJ	-347.731087	-347.629158	-347.715179	-347.695440	-347.787302
B3LYP	-347.652450	-347.544886	-347.638713	-347.604683	-347.702775
B3LYP-D3BJ	-347.678484	-347.570896	-347.664556	-347.635300	-347.732314
PBE0	-347.236639	-347.131867	-347.223506	-347.196237	-347.296822
PBE0-D3BJ	-347.250164	-347.145412	-347.236942	-347.211933	-347.312004
B3PW91	-347.521126	-347.416704	-347.507824	-347.479480	-347.578084
B3PW91-D3	-347.536020	-347.430721	-347.521705	-347.495384	-347.594343
X3LYP	-347.483450	-347.375619	-347.469838	-347.435992	-347.534511
B1LYP	-347.457107	-347.347044	-347.443037	-347.406097	-347.506852
mPW1PW91	-347.565766	-347.459925	-347.552408	-347.523795	-347.624660
B972	-347.530963	-347.423939	-347.517031	-347.484515	-347.586304
MN15	-347.173163	-347.069303	-347.163296	-347.127925	-347.227543
M08HX	-347.540417	-347.428575	-347.528230	-347.491149	-347.594926
M06	-347.394001	-347.288421	-347.378891	-347.347239	-347.446976
M06-D3	-347.395796	-347.290274	-347.380645	-347.349063	-347.448770
M06HF	-347.528066	-347.419513	-347.521898	-347.483855	-347.582689
M06HF-D3	-347.529078	-347.420580	-347.522928	-347.484846	-347.583675
M062X	-347.482120	-347.373718	-347.470867	-347.434318	-347.534468
M062X-D3	-347.482599	-347.374224	-347.471386	-347.434754	-347.534910
M05	-347.384214	-347.277200	-347.365147	-347.330379	-347.437345
M05-D3	-347.389343	-347.282112	-347.370003	-347.335788	-347.442806
M052X	-347.579267	-347.470593	-347.568451	-347.534878	-347.636234
M052X-D3	-347.580421	-347.471807	-347.569614	-347.536021	-347.637364
TPSSH	-347.676044	-347.570817	-347.660521	-347.634055	-347.731312
CAM-B3LYP	-347.458689	-347.349357	-347.444768	-347.408228	-347.512680
CAM-B3LYP-D3BJ	-347.470680	-347.361418	-347.456744	-347.422136	-347.526129
wB97XD	-347.541264	-347.431846	-347.526293	-347.491144	-347.598391
B2PLYP	-347.271461	-347.161303	-347.255727	-347.220772	-347.321914
mPW2PLYP	-347.275365	-347.163816	-347.259862	-347.223881	-347.326883
DSDPBEP86	-346.879265	-346.767570	-346.862835	-346.828657	-346.934216

Table S59: XC/def2-SVP single-point energies on CCSD/6-31G(d,p) optimized geometries for the reaction profile of **hex-5-ynal** (1a) + $\mathbf{H_2O}$.

XC	1	TS_{enol}	2	TS_{conia}	3
CCSD(T)	-384.4419462	-384.3711400	-384.4296563	-384.3639606	-384.4542281
PBE	-384.2628858	-384.2188510	-384.2504926	-384.2048226	-384.2856847
PBE-D3BJ	-384.2791783	-384.2340957	-384.2657477	-384.2213986	-384.3012905
PW91	-384.5872362	-384.5433591	-384.5748430	-384.5291547	-384.6093886
M06L	-384.6936244	-384.6284259	-384.6762616	-384.6162868	-384.7100229
M06L-D3	-384.6942472	-384.6290374	-384.6769497	-384.6168554	-384.7106105
MN12-L	-384.3432564	-384.2788009	-384.3349414	-384.2679002	-384.3569836
MN15-L	-384.3040147	-384.2299133	-384.2879330	-384.2238319	-384.3199147
TPSS	-384.7969563	-384.7451580	-384.7824088	-384.7331593	-384.8187058
TPSS-D3BJ	-384.8173714	-384.7640928	-384.8014627	-384.7538413	-384.8381998
B3LYP	-384.7433476	-384.6858791	-384.7302211	-384.6689160	-384.7596053
B3LYP-D3BJ	-384.7701762	-384.7111346	-384.7553674	-384.6965271	-384.7855325
PBE0	-384.2943108	-384.2406032	-384.2820252	-384.2266009	-384.3183160
PBE0-D3BJ	-384.3085084	-384.2536722	-384.2952791	-384.2408835	-384.3318094
B3PW91	-384.5962596	-384.5435325	-384.5843724	-384.5286509	-384.6189301
B3PW91-D3	-384.6144902	-384.5577374	-384.5999636	-384.5446843	-384.6347116
X3LYP	-384.5675863	-384.5098700	-384.5543862	-384.4929265	-384.5838725
B1LYP	-384.5441820	-384.4836036	-384.5306587	-384.4664794	-384.5596912
mPW1PW91	-384.6442614	-384.5898441	-384.6320603	-384.5755477	-384.6676575
B972	-384.6080895	-384.5503910	-384.5952879	-384.5356792	-384.6296072
MN15	-384.2429996	-384.1833122	-384.2324643	-384.1690904	-384.2597336
M08HX	-384.6118491	-384.5452627	-384.5996447	-384.5318520	-384.6255963
M06	-384.4879492	-384.4265886	-384.4722343	-384.4122777	-384.5031323
M06-D3	-384.4902105	-384.4283012	-384.4743359	-384.4142573	-384.5050820
M06HF	-384.6017960	-384.5459851	-384.5959719	-384.5278270	-384.6147487
M06HF-D3	-384.6029845	-384.5469979	-384.5972055	-384.5289020	-384.6158389
M062X	-384.5640728	-384.5033018	-384.5524052	-384.4860116	-384.5765526
M062X-D3	-384.5645959	-384.5038337	-384.5529872	-384.4864900	-384.5770477
M05	-384.4747962	-384.4100249	-384.4552994	-384.3948960	-384.4955375
M05-D3	-384.4809594	-384.4149390	-384.4607990	-384.4004040	-384.5009512
M052X	-384.6657101	-384.6065862	-384.6545513	-384.5905740	-384.6812471
M052X-D3	-384.6670892	-384.6077247	-384.6559501	-384.5918148	-384.6824957
TPSSH	-384.7568150	-384.7016991	-384.7424909	-384.6895011	-384.7790689
CAM-B3LYP	-384.5485725	-384.4900914	-384.5350030	-384.4706189	-384.5663316
CAM-B3LYP-D3BJ	-384.5612856	-384.5016118	-384.5468690	-384.4832703	-384.5783254
wB97XD	-384.6236601	-384.5601994	-384.6086137	-384.5452106	-384.6434988

Continued on next page

Table S59 (continued)

XC	1	TS_{enol}	2	TS_{conia}	3
B2PLYP	-384.3426843	-384.2797837	-384.3273509	-384.2642007	-384.3561020
mPW2PLYP	-384.3463914	-384.2822046	-384.3310486	-384.2665024	-384.3601356
DSDPBEP86	-383.9206936	-383.8547287	-383.9042260	-383.8407717	-383.9355702

Table S60: XC/def2-SVP single-point energies on CCSD/6-31G(d,p) optimized geometries for the reaction profile of **hept-6-ynal** (1b) + $\mathbf{H_2O}$.

XC	1	TS_{enol}	2	TS_{conia}	3
CCSD(T)	-423.682419	-423.612979	-423.672073	-423.628991	-423.732198
PBE	-423.486208	-423.445427	-423.479543	-423.455019	-423.545020
PBE-D3BJ	-423.504499	-423.464993	-423.498304	-423.476089	-423.565105
PW91	-423.847424	-423.807068	-423.840930	-423.816541	-423.905941
M06L	-423.967502	-423.907757	-423.956200	-423.918777	-424.020165
M06L-D3	-423.968360	-423.908657	-423.957124	-423.919587	-424.020962
MN12-L	-423.570091	-423.512675	-423.568051	-423.525820	-423.625177
MN15-L	-423.531557	-423.465570	-423.522528	-423.482560	-423.588592
TPSS	-424.085318	-424.035456	-424.075048	-424.046587	-424.140756
TPSS-D3BJ	-424.108107	-424.059836	-424.098471	-424.072922	-424.165847
B3LYP	-424.024629	-423.968814	-424.015868	-423.976991	-424.076632
B3LYP-D3BJ	-424.054671	-424.000970	-424.046663	-424.011951	-424.109942
PBE0	-423.527159	-423.476172	-423.519596	-423.486590	-423.588975
PBE0-D3BJ	-423.543001	-423.493121	-423.535927	-423.504840	-423.606361
B3PW91	-423.864722	-423.813187	-423.856536	-423.823137	-423.923413
B3PW91-D3	-423.884265	-423.832955	-423.875868	-423.844187	-423.943970
X3LYP	-423.826799	-423.771272	-423.818345	-423.779398	-423.879498
B1LYP	-423.797750	-423.738692	-423.788395	-423.746844	-423.849101
mPW1PW91	-423.917562	-423.865103	-423.909452	-423.875476	-423.978073
B972	-423.876420	-423.819945	-423.867181	-423.829967	-423.933401
MN15	-423.462001	-423.408196	-423.457750	-423.416558	-423.517326
M08HX	-423.881348	-423.821370	-423.875363	-423.831761	-423.936729
M06	-423.734075	-423.677859	-423.724516	-423.686494	-423.787814
M06-D3	-423.736627	-423.680571	-423.727204	-423.689150	-423.790333
M06HF	-423.865872	-423.814796	-423.864432	-423.821412	-423.920742
M06HF-D3	-423.867367	-423.816387	-423.866030	-423.822883	-423.922175
M062X	-423.821345	-423.766362	-423.815340	-423.773197	-423.874171
M062X-D3	-423.822082	-423.767130	-423.816133	-423.773886	-423.874848
M05	-423.720513	-423.658985	-423.706241	-423.665672	-423.775123
M05-D3	-423.727355	-423.665971	-423.713087	-423.672904	-423.782167

Continued on next page

Table S60 (continued)

XC	1	TS_{enol}	2	TS_{conia}	3
M052X	-423.936579	-423.882050	-423.930500	-423.891709	-423.994270
M052X-D3	-423.938271	-423.883852	-423.932303	-423.893396	-423.995900
TPSSH	-424.042495	-423.989235	-424.032191	-424.000522	-424.099359
CAM-B3LYP	-423.804557	-423.748609	-423.795774	-423.753899	-423.860053
CAM-B3LYP-D3BJ	-423.818660	-423.763714	-423.810420	-423.770154	-423.875525
wB97XD	-423.893716	-423.834978	-423.884342	-423.843868	-423.952187
B2PLYP	-423.575142	-423.514747	-423.564095	-423.524677	-423.626962
mPW2PLYP	-423.579771	-423.518613	-423.569066	-423.528359	-423.632591
DSDPBEP86	-423.108250	-423.046595	-423.096868	-423.058006	-423.164364

5.10 def2-TZVP

Table S61: XC/def2-TZVP single-point energies on CCSD/6-31G(d,p) optimized geometries for the reaction profile of **hex-5-ynal** (1a).

XC	1	TS_{enol}	2	TS_{conia}	3
CCSD(T)	-308.0874483	-307.9784291	-308.0747491	-308.0112212	-308.1013997
PBE	-308.3160396	-308.2213677	-308.3043759	-308.2589450	-308.3372144
PBE-D3BJ	-308.3289283	-308.2339408	-308.3164598	-308.2728228	-308.3502542
PW91	-308.5857236	-308.4904778	-308.5741867	-308.5283892	-308.6061917
M06L	-308.6679577	-308.5593579	-308.6496234	-308.5917826	-308.6833503
M06L-D3	-308.6683286	-308.5597477	-308.6500321	-308.5920926	-308.6836974
MN12-L	-308.3895310	-308.2826462	-308.3800781	-308.3182144	-308.4054029
MN15-L	-308.4190395	-308.3107354	-308.4041211	-308.3464479	-308.4388037
TPSS	-308.7609602	-308.6602846	-308.7473768	-308.6979787	-308.7807627
TPSS-D3BJ	-308.7770876	-308.6759960	-308.7624610	-308.7152915	-308.7970494
B3LYP	-308.7199259	-308.6133309	-308.7079604	-308.6450169	-308.7341539
B3LYP-D3BJ	-308.7414613	-308.6343543	-308.7281781	-308.6684817	-308.7560919
PBE0	-308.3400228	-308.2368424	-308.3283426	-308.2739508	-308.3627030
PBE0-D3BJ	-308.3511541	-308.2476834	-308.3387568	-308.2858090	-308.3739036
B3PW91	-308.5911505	-308.4881080	-308.5796827	-308.5239253	-308.6117553
B3PW91-D3	-308.6029315	-308.4988182	-308.5895708	-308.5352618	-308.6229705
X3LYP	-308.5732049	-308.4663559	-308.5612590	-308.4983323	-308.5876464
B1LYP	-308.5530318	-308.4441735	-308.5407744	-308.4748771	-308.5666000
mPW1PW91	-308.6307498	-308.5264732	-308.6190959	-308.5630418	-308.6524451
B972	-308.5989131	-308.4937293	-308.5869257	-308.5274684	-308.6187598
MN15	-308.3341481	-308.2300437	-308.3240210	-308.2622658	-308.3507812
M08HX	-308.6250596	-308.5139943	-308.6133711	-308.5482500	-308.6389096

Continued on next page

Table S61 (continued)

XC	1	TS_{enol}	2	TS_{conia}	3
M06	-308.4935821	-308.3871979	-308.4781806	-308.4176000	-308.5062530
M06-D3	-308.4949400	-308.3884538	-308.4793976	-308.4187790	-308.5075429
M06HF	-308.6291608	-308.5230510	-308.6247911	-308.5588495	-308.6418071
M06HF-D3	-308.6298808	-308.5237570	-308.6254969	-308.5594684	-308.6425008
M062X	-308.5765826	-308.4688692	-308.5656742	-308.5011627	-308.5895315
M062X-D3	-308.5768907	-308.4691975	-308.5660250	-308.5014183	-308.5898163
M05	-308.4798818	-308.3745326	-308.4629942	-308.4016938	-308.4976825
M05-D3	-308.4838672	-308.3781820	-308.4664221	-308.4055087	-308.5014978
M052X	-308.6702379	-308.5628542	-308.6601661	-308.5976191	-308.6850401
M052X-D3	-308.6710727	-308.5636593	-308.6609653	-308.5983397	-308.6858442
TPSSH	-308.7269373	-308.6233320	-308.7135113	-308.6606948	-308.7473992
CAM-B3LYP	-308.5517659	-308.4432959	-308.5393044	-308.4735285	-308.5675422
CAM-B3LYP-D3BJ	-308.5616707	-308.4529329	-308.5485751	-308.4839543	-308.5774437
wB97XD	-308.6097425	-308.5011917	-308.5954426	-308.5323714	-308.6287220
B2PLYP	-308.4455728	-308.3380453	-308.4320400	-308.3698703	-308.4580703
mPW2PLYP	-308.4439615	-308.3350389	-308.4304823	-308.3669919	-308.4569520
DSDPBEP86	-308.1290699	-308.0208665	-308.1144879	-308.0541157	-308.1441250

Table S62: XC/def2-TZVP single-point energies on CCSD/6-31G(d,p) optimized geometries for the reaction profile of **hept-6-ynal** (1b).

XC	1	TS_{enol}	2	TS_{conia}	3
CCSD(T)	-347.328943	-347.217226	-347.315776	-347.276947	-347.378884
PBE	-347.584711	-347.487871	-347.572364	-347.550158	-347.637358
PBE-D3BJ	-347.600409	-347.503550	-347.587913	-347.568393	-347.654987
PW91	-347.891805	-347.794394	-347.879619	-347.857383	-347.944068
M06L	-347.986763	-347.876881	-347.968822	-347.934548	-348.033335
M06L-D3	-347.987326	-347.877476	-347.969424	-347.935070	-348.033864
MN12-L	-347.663393	-347.554727	-347.654575	-347.617168	-347.714702
MN15-L	-347.701010	-347.590842	-347.686782	-347.651980	-347.755642
TPSS	-348.093057	-347.989829	-348.078441	-348.051985	-348.142714
TPSS-D3BJ	-348.112616	-348.009381	-348.097844	-348.074777	-348.164745
B3LYP	-348.046895	-347.937473	-348.033683	-347.995336	-348.092551
B3LYP-D3BJ	-348.072929	-347.963483	-348.059525	-348.025953	-348.122090
PBE0	-347.616860	-347.511062	-347.604383	-347.573971	-347.673015
PBE0-D3BJ	-347.630385	-347.524607	-347.617819	-347.589666	-347.688198
B3PW91	-347.903354	-347.797637	-347.890725	-347.858986	-347.956123
B3PW91-D3	-347.918248	-347.811653	-347.904605	-347.874890	-347.972382

Continued on next page

Table S62 (continued)

XC	1	TS_{enol}	2	TS_{conia}	3
X3LYP	-347.878333	-347.768665	-347.865253	-347.827041	-347.924692
B1LYP	-347.852296	-347.740464	-347.838718	-347.797537	-347.897384
mPW1PW91	-347.947947	-347.840974	-347.935298	-347.903379	-348.002692
B972	-347.910540	-347.802600	-347.897352	-347.861660	-347.961852
MN15	-347.605748	-347.499382	-347.595700	-347.557595	-347.656813
M08HX	-347.942483	-347.829469	-347.931003	-347.890824	-347.992487
M06	-347.786244	-347.677966	-347.770836	-347.733812	-347.833194
M06-D3	-347.788039	-347.679818	-347.772590	-347.735637	-347.834989
M06HF	-347.942727	-347.833426	-347.937584	-347.897768	-347.993139
M06HF-D3	-347.943738	-347.834492	-347.938614	-347.898760	-347.994125
M062X	-347.881900	-347.771953	-347.870940	-347.831151	-347.929941
M062X-D3	-347.882380	-347.772459	-347.871460	-347.831586	-347.930383
M05	-347.769238	-347.661402	-347.751621	-347.711066	-347.815802
M05-D3	-347.774367	-347.666313	-347.756477	-347.716475	-347.821262
M052X	-347.989911	-347.879977	-347.979499	-347.943381	-348.042693
M052X-D3	-347.991066	-347.881191	-347.980663	-347.944524	-348.043824
TPSSH	-348.056024	-347.949724	-348.041551	-348.011975	-348.107244
CAM-B3LYP	-347.853620	-347.742214	-347.840125	-347.799120	-347.902758
CAM-B3LYP-D3BJ	-347.865611	-347.754276	-347.852101	-347.813028	-347.916207
wB97XD	-347.924802	-347.813710	-347.910120	-347.870994	-347.977095
B2PLYP	-347.730807	-347.620635	-347.716313	-347.679332	-347.777237
mPW2PLYP	-347.729699	-347.618085	-347.715362	-347.677210	-347.777136
DSDPBEP86	-347.373574	-347.263014	-347.358667	-347.323738	-347.424931

Table S63: XC/def2-TZVP single-point energies on CCSD/6-31G(d,p) optimized geometries for the reaction profile of **hex-5-ynal** (1a) + $\mathbf{H_2O}$.

XC	1	TS_{enol}	2	TS_{conia}	3
CCSD(T)	-384.4419462	-384.3711400	-384.4296563	-384.3639606	-384.4542281
PBE	-384.7040353	-384.6546716	-384.6936405	-384.6450879	-384.7239211
PBE-D3BJ	-384.7203278	-384.6699163	-384.7088956	-384.6616639	-384.7395269
PW91	-385.0324991	-384.9832428	-385.0222503	-384.9732950	-385.0516451
M06L	-385.1245510	-385.0547817	-385.1066358	-385.0451298	-385.1372372
M06L-D3	-385.1251738	-385.0553932	-385.1073239	-385.0456984	-385.1378249
MN12-L	-384.7824191	-384.7139822	-384.7734861	-384.7068691	-384.7940494
MN15-L	-384.8104271	-384.7351020	-384.7948383	-384.7327319	-384.8256506
TPSS	-385.2340705	-385.1778941	-385.2217612	-385.1699175	-385.2527893
TPSS-D3BJ	-385.2544856	-385.1968289	-385.2408152	-385.1905996	-385.2722832

Continued on next page

Table S63 (continued)

XC	1	TS_{enol}	2	TS_{conia}	3
B3LYP	-385.1921535	-385.1294569	-385.1808622	-385.1161388	-385.2054363
B3LYP-D3BJ	-385.2189822	-385.1547124	-385.2060085	-385.1437499	-385.2313635
PBE0	-384.7282814	-384.6704888	-384.7177145	-384.6602781	-384.7493896
PBE0-D3BJ	-384.7424789	-384.6835578	-384.7309684	-384.6745607	-384.7628830
B3PW91	-385.0322382	-384.9749896	-385.0220086	-384.9639464	-385.0517952
B3PW91-D3	-385.0504688	-384.9891944	-385.0375998	-384.9799798	-385.0675768
X3LYP	-385.0169156	-384.9540360	-385.0055976	-384.9406728	-385.0302441
B1LYP	-384.9936126	-384.9280149	-384.9819037	-384.9143920	-385.0062089
mPW1PW91	-385.0801499	-385.0216009	-385.0696703	-385.0109721	-385.1005082
B972	-385.0414938	-384.9800021	-385.0304611	-384.9688931	-385.0602895
MN15	-308.3341481	-308.2300437	-308.3240210	-308.2622658	-308.3507812
M08HX	-308.6250596	-308.5139943	-308.6133711	-308.5482500	-308.6389096
M06	-308.4935821	-308.3871979	-308.4781806	-308.4176000	-308.5062530
M06-D3	-384.9312552	-384.8636708	-384.9158317	-384.8516747	-384.9411772
M06HF	-308.6291608	-308.5230510	-308.6247911	-308.5588495	-308.6418071
M06HF-D3	-385.0759516	-385.0156930	-385.0724166	-385.0029551	-385.0852678
M062X	-308.5765826	-308.4688692	-308.5656742	-308.5011627	-308.5895315
M062X-D3	-385.0177826	-384.9517048	-385.0071915	-384.9388576	-385.0271474
M05	-308.4798818	-308.3745326	-308.4629942	-308.4016938	-308.4976825
M05-D3	-384.9208210	-384.8515751	-384.9034725	-384.8390438	-384.9364676
M052X	-308.6702379	-308.5628542	-308.6601661	-308.5976191	-308.6850401
M052X-D3	-385.1319528	-385.0684147	-385.1223198	-385.0562592	-385.1439401
TPSSH	-308.7269373	-308.6233320	-308.7135113	-308.6606948	-308.7473992
CAM-B3LYP	-384.9981935	-384.9340335	-384.9862864	-384.9182704	-385.0125361
CAM-B3LYP-D3BJ	-385.0109066	-384.9455539	-384.9981524	-384.9309218	-385.0245299
wB97XD	-385.0608831	-384.9921690	-385.0468844	-384.9808879	-385.0770732
B2PLYP	-384.8640049	-384.7984130	-384.8510221	-384.7867174	-384.8749413
mPW2PLYP	-384.8620804	-384.7952387	-384.8490639	-384.7832395	-384.8733465
DSDPBEP86	-384.4813065	-384.4140941	-384.4672713	-384.4040417	-384.4939531

Table S64: XC/def2-TZVP single-point energies on CCSD/6-31G(d,p) optimized geometries for the reaction profile of **hept-6-ynal** (1b) + $\mathbf{H_2O}$.

XC	1	TS_{enol}	2	TS_{conia}	3
CCSD(T)	-423.682419	-423.612979	-423.672073	-423.628991	-423.732198
PBE	-423.971307	-423.922273	-423.963390	-423.935688	-424.024574
PBE-D3BJ	-423.989597	-423.941839	-423.982151	-423.956758	-424.044659
PW91	-424.337180	-424.288376	-424.329485	-424.301655	-424.390028

Continued on next page

Table S64 (continued)

XC	1	TS_{enol}	2	TS_{conia}	3
M06L	-424.441210	-424.375361	-424.427621	-424.387733	-424.487607
M06L-D3	-424.442068	-424.376261	-424.428545	-424.388542	-424.488405
MN12-L	-424.052927	-423.989962	-424.048922	-424.005717	-424.104109
MN15-L	-424.088703	-424.019718	-424.078420	-424.038471	-424.142688
TPSS	-424.565360	-424.508343	-424.554402	-424.523229	-424.615482
TPSS-D3BJ	-424.588149	-424.532722	-424.577824	-424.549565	-424.640573
B3LYP	-424.518409	-424.454387	-424.508230	-424.465725	-424.564564
B3LYP-D3BJ	-424.548451	-424.486543	-424.539025	-424.500685	-424.597874
PBE0	-424.003748	-423.946137	-423.995195	-423.959611	-424.060356
PBE0-D3BJ	-424.019590	-423.963086	-424.011525	-423.977861	-424.077742
B3PW91	-424.343616	-424.285087	-424.334430	-424.298269	-424.396977
B3PW91-D3	-424.363159	-424.304855	-424.353762	-424.319320	-424.417534
X3LYP	-424.321166	-424.257433	-424.311277	-424.268655	-424.367972
B1LYP	-424.292206	-424.225131	-424.281437	-424.236310	-424.337751
mPW1PW91	-424.396273	-424.337136	-424.387239	-424.350582	-424.451513
B972	-424.352304	-424.289469	-424.342235	-424.302408	-424.404158
MN15	-423.999839	-423.937220	-423.993641	-423.950399	-424.050490
M08HX	-424.383840	-424.316849	-424.376731	-424.330922	-424.433601
M06	-424.219586	-424.155469	-424.208079	-424.165456	-424.266281
M06-D3	-424.222138	-424.158182	-424.210768	-424.168112	-424.268800
M06HF	-424.386533	-424.328793	-424.384253	-424.339941	-424.435660
M06HF-D3	-424.388028	-424.330384	-424.385851	-424.341412	-424.437093
M062X	-424.320057	-424.257797	-424.312778	-424.267964	-424.367472
M062X-D3	-424.320794	-424.258564	-424.313571	-424.268653	-424.368149
M05	-424.202817	-424.134954	-424.188381	-424.142458	-424.249662
M05-D3	-424.209660	-424.141941	-424.195226	-424.149690	-424.256706
M052X	-424.448312	-424.387528	-424.441183	-424.400170	-424.500577
M052X-D3	-424.450004	-424.389331	-424.442986	-424.401856	-424.502208
TPSSH	-424.520079	-424.460166	-424.509122	-424.474907	-424.571718
CAM-B3LYP	-424.299000	-424.234542	-424.288674	-424.243095	-424.348402
CAM-B3LYP-D3BJ	-424.313103	-424.249648	-424.303321	-424.259349	-424.363874
wB97XD	-424.373645	-424.307480	-424.362899	-424.319172	-424.426157
B2PLYP	-424.148135	-424.082660	-424.136736	-424.095499	-424.194670
mPW2PLYP	-424.146562	-424.080263	-424.135427	-424.092778	-424.194059
DSDPBEP86	-423.723938	-423.659069	-423.712704	-423.673076	-423.775167

5.11 def2-TZVPP

Table S65: XC/def2-TZVPP single-point energies on CCSD/6-31G(d,p) optimized geometries for the reaction profile of **hex-5-ynal** (1a).

XC	1	TS_{enol}	2	TS_{conia}	3
CCSD(T)	-308.0874483	-307.9784291	-308.0747491	-308.0112212	-308.1013997
PBE	-308.3189129	-308.2246057	-308.3085003	-308.2622260	-308.3400772
PBE-D3BJ	-308.3318015	-308.2371789	-308.3205842	-308.2761039	-308.3531170
PW91	-308.5887464	-308.4938592	-308.5784221	-308.5318061	-308.6091963
M06L	-308.6716061	-308.5634450	-308.6551195	-308.5961048	-308.6872013
M06L-D3	-308.6719770	-308.5638348	-308.6555283	-308.5964148	-308.6875483
MN12-L	-308.3924527	-308.2861401	-308.3844573	-308.3218717	-308.4083484
MN15-L	-308.4224538	-308.3143733	-308.4088919	-308.3503228	-308.4423699
TPSS	-308.7635000	-308.6631423	-308.7509256	-308.7008732	-308.7833136
TPSS-D3BJ	-308.7796274	-308.6788536	-308.7660097	-308.7181859	-308.7996003
B3LYP	-308.7230947	-308.6168453	-308.7124009	-308.6485458	-308.7372675
B3LYP-D3BJ	-308.7446300	-308.6378687	-308.7326187	-308.6720107	-308.7592055
PBE0	-308.3426681	-308.2398923	-308.3322208	-308.2770548	-308.3653544
PBE0-D3BJ	-308.3537994	-308.2507333	-308.3426350	-308.2889130	-308.3765550
B3PW91	-308.5940036	-308.4913608	-308.5837512	-308.5272202	-308.6145963
B3PW91-D3	-308.6057847	-308.5020709	-308.5936393	-308.5385567	-308.6258115
X3LYP	-308.5763677	-308.4698627	-308.5656952	-308.5018560	-308.5907552
B1LYP	-308.5561827	-308.4476657	-308.5452003	-308.4783866	-308.5696990
mPW1PW91	-308.6335388	-308.5296664	-308.6230893	-308.5662818	-308.6552339
B972	-308.6016592	-308.4968329	-308.5908377	-308.5306404	-308.6215522
MN15	-308.3370472	-308.2334638	-308.3280590	-308.2657120	-308.3535815
M08HX	-308.6299545	-308.5191584	-308.6192746	-308.5533756	-308.6437873
M06	-308.4974265	-308.3915218	-308.4836825	-308.4219363	-308.5100172
M06-D3	-308.4987844	-308.3927777	-308.4848995	-308.4231152	-308.5113072
M06HF	-308.6380296	-308.5320485	-308.6338584	-308.5680987	-308.6511810
M06HF-D3	-308.6387496	-308.5327546	-308.6345642	-308.5687176	-308.6518747
M062X	-308.5796878	-308.4723450	-308.5697350	-308.5045914	-308.5925801
M062X-D3	-308.5799960	-308.4726733	-308.5700858	-308.5048470	-308.5928650
M05	-308.4849660	-308.3797620	-308.4691628	-308.4069215	-308.5028863
M05-D3	-308.4889514	-308.3834114	-308.4725908	-308.4107364	-308.5067016
M052X	-308.6746671	-308.5676161	-308.6656296	-308.6023449	-308.6894015
M052X-D3	-308.6755018	-308.5684212	-308.6664287	-308.6030655	-308.6902056
TPSSH	-308.7294681	-308.6262009	-308.7170689	-308.6636019	-308.7499463
CAM-B3LYP	-308.5548839	-308.4467989	-308.5436924	-308.4770497	-308.5705883
CAM-B3LYP-D3BJ	-308.5647886	-308.4564360	-308.5529632	-308.4874755	-308.5804897
wB97XD	-308.6129969	-308.5048180	-308.5999519	-308.5360869	-308.6319705

Continued on next page

Table S65 (continued)

XC	1	TS_{enol}	2	TS_{conia}	3
B2PLYP	-308.4547486	-308.3476436	-308.4426693	-308.3797271	-308.4673146
mPW2PLYP	-308.4526595	-308.3441528	-308.4406149	-308.3763463	-308.4657117
DSDPBEP86	-308.1428609	-308.0350435	-308.1297527	-308.0687185	-308.1580944

Table S66: XC/def2-TZVPP single-point energies on CCSD/6-31G(d,p) optimized geometries for the reaction profile of **hept-6-ynal** (1b).

XC	1	TS_{enol}	2	TS_{conia}	3
CCSD(T)	-347.328943	-347.217226	-347.315776	-347.276947	-347.378884
PBE	-347.588251	-347.491748	-347.577132	-347.554125	-347.640845
PBE-D3BJ	-347.603949	-347.507427	-347.592680	-347.572360	-347.658474
PW91	-347.895530	-347.798453	-347.884535	-347.861530	-347.947743
M06L	-347.991147	-347.881607	-347.974989	-347.939542	-348.037742
M06L-D3	-347.991710	-347.882203	-347.975592	-347.940064	-348.038271
MN12-L	-347.666936	-347.558742	-347.659501	-347.621475	-347.718199
MN15-L	-347.705276	-347.595251	-347.692290	-347.656686	-347.759910
TPSS	-348.096206	-347.993280	-348.082590	-348.055498	-348.145832
TPSS-D3BJ	-348.115765	-348.012833	-348.101992	-348.078290	-348.167863
B3LYP	-348.050801	-347.941692	-348.038841	-347.999639	-348.096376
B3LYP-D3BJ	-348.076835	-347.967702	-348.064683	-348.030256	-348.125915
PBE0	-347.620127	-347.514697	-347.608863	-347.577714	-347.676245
PBE0-D3BJ	-347.633652	-347.528242	-347.622299	-347.593410	-347.691428
B3PW91	-347.906871	-347.801522	-347.895443	-347.862967	-347.959592
B3PW91-D3	-347.921765	-347.815538	-347.909324	-347.878871	-347.975851
X3LYP	-347.882233	-347.772876	-347.870406	-347.831340	-347.928514
B1LYP	-347.856184	-347.744659	-347.843860	-347.801821	-347.901194
mPW1PW91	-347.951388	-347.844787	-347.939928	-347.907294	-348.006097
B972	-347.913937	-347.806322	-347.901895	-347.865500	-347.965245
MN15	-347.609296	-347.503404	-347.600353	-347.561730	-347.660236
M08HX	-347.948532	-347.835752	-347.938039	-347.897179	-347.998549
M06	-347.790826	-347.682955	-347.777013	-347.738926	-347.837687
M06-D3	-347.792621	-347.684807	-347.778768	-347.740751	-347.839481
M06HF	-347.953891	-347.844769	-347.948954	-347.909276	-348.004878
M06HF-D3	-347.954903	-347.845835	-347.949984	-347.910268	-348.005864
M062X	-347.885758	-347.776147	-347.875739	-347.835379	-347.933764
M062X-D3	-347.886238	-347.776653	-347.876259	-347.835814	-347.934206
M05	-347.775464	-347.667736	-347.758884	-347.717445	-347.822081
M05-D3	-347.780593	-347.672647	-347.763740	-347.722853	-347.827542

Continued on next page

Table S66 (continued)

XC	1	TS_{enol}	2	TS_{conia}	3
M052X	-347.995383	-347.885759	-347.986000	-347.949205	-348.048110
M052X-D3	-347.996538	-347.886973	-347.987164	-347.950348	-348.049241
TPSSH	-348.059163	-347.953181	-348.045708	-348.015501	-348.110356
CAM-B3LYP	-347.857466	-347.746408	-347.845224	-347.803413	-347.906514
CAM-B3LYP-D3BJ	-347.869457	-347.758469	-347.857200	-347.817321	-347.919963
wB97XD	-347.928794	-347.818032	-347.915351	-347.875491	-347.981070
B2PLYP	-347.742298	-347.632515	-347.729245	-347.691508	-347.788754
mPW2PLYP	-347.740589	-347.629355	-347.727673	-347.688765	-347.788048
DSDPBEP86	-347.390884	-347.280673	-347.377439	-347.341849	-347.442368

Table S67: XC/def2-TZVPP single-point energies on CCSD/6-31G(d,p) optimized geometries for the reaction profile of **hex-5-ynal** (1a) + $\mathbf{H_2O}$.

XC	1	TS_{enol}	2	TS_{conia}	3
CCSD(T)	-384.4419462	-384.3711400	-384.4296563	-384.3639606	-384.4542281
PBE	-384.7098328	-384.6606420	-384.7006172	-384.6514900	-384.7300147
PBE-D3BJ	-384.7261254	-384.6758867	-384.7158723	-384.6680660	-384.7456205
PW91	-385.0384348	-384.9893565	-385.0293248	-384.9798156	-385.0578536
M06L	-385.1323823	-385.0623001	-385.1159979	-385.0537669	-385.1453743
M06L-D3	-385.1330051	-385.0629116	-385.1166860	-385.0543354	-385.1459619
MN12-L	-384.7887858	-384.7207044	-384.7810798	-384.7140313	-384.8005079
MN15-L	-384.8170627	-384.7416371	-384.8026876	-384.7399746	-384.8326327
TPSS	-385.2390720	-385.1831605	-385.2277019	-385.1754678	-385.2580925
TPSS-D3BJ	-385.2594871	-385.2020953	-385.2467558	-385.1961498	-385.2775865
B3LYP	-385.1984057	-385.1357898	-385.1882891	-385.1229175	-385.2119065
B3LYP-D3BJ	-385.2252344	-385.1610453	-385.2134354	-385.1505286	-385.2378337
PBE0	-384.7338440	-384.6762641	-384.7244122	-384.6664663	-384.7552234
PBE0-D3BJ	-384.7480416	-384.6893330	-384.7376661	-384.6807489	-384.7687169
B3PW91	-385.0380350	-384.9810134	-385.0289188	-384.9703502	-385.0578586
B3PW91-D3	-385.0562655	-384.9952182	-385.0445100	-384.9863836	-385.0736402
X3LYP	-385.0231680	-384.9603599	-385.0130253	-384.9474505	-385.0367069
B1LYP	-384.9998581	-384.9343214	-384.9893284	-384.9211578	-385.0126675
mPW1PW91	-385.0858567	-385.0275390	-385.0764839	-385.0172930	-385.1064791
B972	-385.0470610	-384.9857301	-385.0371200	-384.9750585	-385.0661783
MN15	-384.7366290	-384.6704777	-384.7276338	-384.6616861	-384.7498415
M08HX	-385.0772036	-385.0060446	-385.0671014	-384.9971428	-385.0876214
M06	-384.9367491	-384.8694965	-384.9228648	-384.8580285	-384.9469876
M06-D3	-384.9390104	-384.8712091	-384.9249664	-384.8600081	-384.9489373

Continued on next page

Table S67 (continued)

XC	1	TS_{enol}	2	TS_{conia}	3
M06HF	-385.0862116	-385.0272311	-385.0826528	-385.0137761	-385.0963253
M06HF-D3	-385.0874002	-385.0282440	-385.0838864	-385.0148511	-385.0974156
M062X	-385.0229609	-384.9572218	-385.0131128	-384.9445345	-385.0324982
M062X-D3	-385.0234841	-384.9577536	-385.0136949	-384.9450128	-385.0329933
M05	-384.9230054	-384.8544033	-384.9072665	-384.8421340	-384.9396921
M05-D3	-384.9291686	-384.8593175	-384.9127661	-384.8476420	-384.9451057
M052X	-385.1380184	-385.0750745	-385.1292424	-385.0628893	-385.1502911
M052X-D3	-385.1393975	-385.0762130	-385.1306412	-385.0641300	-385.1515397
TPSSH	-385.1968882	-385.1380388	-385.1856130	-385.1299414	-385.2164314
CAM-B3LYP	-385.0044651	-384.9404237	-384.9937158	-384.9250939	-385.0189810
CAM-B3LYP-D3BJ	-385.0171782	-384.9519441	-385.0055818	-384.9377453	-385.0309748
wB97XD	-385.0673719	-384.9987224	-385.0544926	-384.9879640	-385.0837748
B2PLYP	-384.8785121	-384.8130401	-384.8668372	-384.8020953	-384.8897828
mPW2PLYP	-384.8759315	-384.8092003	-384.8642079	-384.7979309	-384.8875116
DSDPBEP86	-384.5018032	-384.4346947	-384.4890659	-384.4255456	-384.5148853

Table S68: XC/def2-TZVPP single-point energies on CCSD/6-31G(d,p) optimized geometries for the reaction profile of **hept-6-ynal** (1b) + $\mathbf{H_2O}$.

XC	1	TS_{enol}	2	TS_{conia}	3
CCSD(T)	-423.682419	-423.612979	-423.672073	-423.628991	-423.732198
PBE	-423.978128	-423.928849	-423.971116	-423.942677	-424.031351
PBE-D3BJ	-423.996419	-423.948415	-423.989877	-423.963747	-424.051436
PW91	-424.344161	-424.295131	-424.337354	-424.308812	-424.396968
M06L	-424.449968	-424.383367	-424.437607	-424.397002	-424.496384
M06L-D3	-424.450826	-424.384268	-424.438531	-424.397812	-424.497181
MN12-L	-424.060102	-423.997048	-424.057050	-424.013511	-424.111195
MN15-L	-424.096424	-424.026935	-424.086998	-424.046496	-424.150428
TPSS	-424.571303	-424.514166	-424.561050	-424.529306	-424.621416
TPSS-D3BJ	-424.594092	-424.538545	-424.584473	-424.555642	-424.646507
B3LYP	-424.525714	-424.461383	-424.516468	-424.473192	-424.571800
B3LYP-D3BJ	-424.555755	-424.493538	-424.547263	-424.508153	-424.605110
PBE0	-424.010247	-423.952443	-424.002605	-423.966353	-424.066829
PBE0-D3BJ	-424.026089	-423.969392	-424.018935	-423.984603	-424.084215
B3PW91	-424.350409	-424.291688	-424.342102	-424.305273	-424.403731
B3PW91-D3	-424.369951	-424.311457	-424.361434	-424.326324	-424.424288
X3LYP	-424.328461	-424.264418	-424.319515	-424.276125	-424.375201
B1LYP	-424.299492	-424.232099	-424.289668	-424.243767	-424.344972

Continued on next page

Table S68 (continued)

XC	1	TS_{enol}	2	TS_{conia}	3
mPW1PW91	-424.402947	-424.343636	-424.394804	-424.357496	-424.458163
B972	-424.358848	-424.295781	-424.349629	-424.309156	-424.410707
MN15	-424.006481	-423.944003	-424.001081	-423.957380	-424.057002
M08HX	-424.393336	-424.326060	-424.386847	-424.340491	-424.443057
M06	-424.228256	-424.163502	-424.217948	-424.174545	-424.274833
M06-D3	-424.230808	-424.166214	-424.220637	-424.177201	-424.277352
M06HF	-424.400533	-424.343346	-424.398408	-424.354040	-424.450252
M06HF-D3	-424.402027	-424.344937	-424.400006	-424.355511	-424.451685
M062X	-424.326767	-424.264420	-424.320196	-424.274855	-424.374167
M062X-D3	-424.327504	-424.265188	-424.320989	-424.275544	-424.374844
M05	-424.212552	-424.143742	-424.198835	-424.152151	-424.259405
M05-D3	-424.219395	-424.150728	-424.205681	-424.159384	-424.266449
M052X	-424.457073	-424.396194	-424.450659	-424.409077	-424.509292
M052X-D3	-424.458765	-424.397997	-424.452462	-424.410764	-424.510923
TPSSH	-424.526048	-424.466023	-424.515820	-424.481039	-424.577682
CAM-B3LYP	-424.306285	-424.241557	-424.296923	-424.250611	-424.355611
CAM-B3LYP-D3BJ	-424.320388	-424.256663	-424.311569	-424.266866	-424.371083
wB97XD	-424.381159	-424.314648	-424.371377	-424.326959	-424.433647
B2PLYP	-424.165265	-424.099522	-424.155029	-424.113112	-424.211860
mPW2PLYP	-424.162897	-424.096332	-424.152918	-424.109588	-424.210450
DSDPBEP86	-423.748260	-423.683086	-423.738225	-423.698007	-423.799656

5.12 pc-2

Table S69: XC/pc-2 single-point energies on CCSD/6-31G(d,p) optimized geometries for the reaction profile of **hex-5-ynal** (1a).

XC	1	TS_{enol}	2	TS_{conia}	3
CCSD(T)	-308.0874483	-307.9784291	-308.0747491	-308.0112212	-308.1013997
PBE	-308.3279491	-308.2336017	-308.3177625	-308.2711714	-308.3487525
PBE-D3BJ	-308.3408378	-308.2461748	-308.3298464	-308.2850493	-308.3617923
PW91	-308.5985940	-308.5036956	-308.5885458	-308.5415457	-308.6186599
M06L	-308.6684744	-308.5600387	-308.6520953	-308.5926835	-308.6835651
M06L-D3	-308.6688453	-308.5604286	-308.6525041	-308.5929935	-308.6839121
MN12-L	-308.3723098	-308.2657758	-308.3645500	-308.3015735	-308.3880937
MN15-L	-308.4433064	-308.3348539	-308.4299328	-308.3710100	-308.4633027
TPSS	-308.7665866	-308.6661019	-308.7541705	-308.7036539	-308.7859023
TPSS-D3BJ	-308.7827140	-308.6818132	-308.7692546	-308.7209666	-308.8021890

Continued on next page

Table S69 (continued)

XC	1	TS_{enol}	2	TS_{conia}	3
B3LYP	-308.7301390	-308.6238440	-308.7196759	-308.6554722	-308.7438803
B3LYP-D3BJ	-308.7516743	-308.6448674	-308.7398937	-308.6789371	-308.7658183
PBE0	-308.3466862	-308.2437900	-308.3363911	-308.2809191	-308.3689596
PBE0-D3BJ	-308.3578175	-308.2546310	-308.3468053	-308.2927773	-308.3801602
B3PW91	-308.5993990	-308.4966603	-308.5893228	-308.5324564	-308.6195710
B3PW91-D3	-308.6111801	-308.5073705	-308.5992109	-308.5437929	-308.6307862
X3LYP	-308.5832507	-308.4767022	-308.5728174	-308.5086195	-308.5972031
B1LYP	-308.5624514	-308.4538820	-308.5516931	-308.4845279	-308.5755234
mPW1PW91	-308.6380258	-308.5340552	-308.6277586	-308.5705968	-308.6592850
B972	-308.6021539	-308.4971861	-308.5914730	-308.5309316	-308.6215806
MN15	-308.3781351	-308.2744358	-308.3693887	-308.3068843	-308.3947609
M08HX	-308.6456690	-308.5350103	-308.6352621	-308.5688421	-308.6588711
M06	-308.5065151	-308.4006315	-308.4931272	-308.4312679	-308.5190062
M06-D3	-308.5078731	-308.4018874	-308.4943443	-308.4324468	-308.5202961
M06HF	-308.6608103	-308.5544454	-308.6562168	-308.5894600	-308.6729160
M06HF-D3	-308.6615304	-308.5551515	-308.6569225	-308.5900789	-308.6736097
M062X	-308.5890031	-308.4816319	-308.5791614	-308.5137112	-308.6013870
M062X-D3	-308.5893113	-308.4819602	-308.5795122	-308.5139668	-308.6016719
M05	-308.4959021	-308.3904511	-308.4803009	-308.4175667	-308.5132701
M05-D3	-308.4998874	-308.3941004	-308.4837288	-308.4213815	-308.5170854
M052X	-308.6850402	-308.5780393	-308.6761179	-308.6124932	-308.6990464
M052X-D3	-308.6858750	-308.5788445	-308.6769170	-308.6132138	-308.6998505
TPSSH	-308.7311508	-308.6277333	-308.7188887	-308.6649840	-308.7511271
CAM-B3LYP	-308.5622210	-308.4540656	-308.5512424	-308.4843084	-308.5775281
CAM-B3LYP-D3BJ	-308.5721258	-308.4637027	-308.5605131	-308.4947342	-308.5874296
wB97XD	-308.6180604	-308.5097033	-308.6051294	-308.5409802	-308.6366939
B2PLYP	-308.4506955	-308.3432363	-308.4387299	-308.3752026	-308.4627355
mPW2PLYP	-308.4488278	-308.3399890	-308.4369120	-308.3720612	-308.4613487
DSDPBEP86	-308.1316207	-308.0232184	-308.1185076	-308.0567752	-308.1462796

Table S70: XC/pc-2 single-point energies on CCSD/6-31G(d,p) optimized geometries for the reaction profile of **hept-6-ynal** (1b).

XC	1	TS_{enol}	2	TS_{conia}	3
CCSD(T)	-347.328943	-347.217226	-347.315776	-347.276947	-347.378884
PBE	-347.598546	-347.501999	-347.587635	-347.564257	-347.650659
PBE-D3BJ	-347.614244	-347.517678	-347.603183	-347.582491	-347.668288
PW91	-347.906725	-347.809632	-347.895982	-347.872540	-347.958406

Continued on next page

Table S70 (continued)

XC	1	TS_{enol}	2	TS_{conia}	3
M06L	-347.987695	-347.877854	-347.971611	-347.935505	-348.033433
M06L-D3	-347.988259	-347.878450	-347.972213	-347.936027	-348.033961
MN12-L	-347.643931	-347.535427	-347.636682	-347.598180	-347.694901
MN15-L	-347.729466	-347.619018	-347.716670	-347.680808	-347.784370
TPSS	-348.099694	-347.996654	-348.086224	-348.058609	-348.148695
TPSS-D3BJ	-348.119253	-348.016206	-348.105626	-348.081401	-348.170726
B3LYP	-348.058819	-347.949667	-348.047076	-348.007465	-348.103827
B3LYP-D3BJ	-348.084853	-347.975677	-348.072918	-348.038082	-348.133366
PBE0	-347.624738	-347.519191	-347.613614	-347.582140	-347.680379
PBE0-D3BJ	-347.638263	-347.532736	-347.627050	-347.597836	-347.695562
B3PW91	-347.913026	-347.807587	-347.901765	-347.868917	-347.965222
B3PW91-D3	-347.927920	-347.821604	-347.915646	-347.884821	-347.981481
X3LYP	-347.890067	-347.780668	-347.878462	-347.838980	-347.935777
B1LYP	-347.863324	-347.751747	-347.851210	-347.808760	-347.907758
mPW1PW91	-347.956516	-347.849814	-347.945221	-347.912209	-348.010694
B972	-347.914514	-347.806749	-347.902603	-347.865848	-347.965305
MN15	-347.656084	-347.550097	-347.647350	-347.608474	-347.706972
M08HX	-347.966396	-347.853661	-347.956071	-347.914761	-348.015645
M06	-347.801216	-347.693329	-347.787701	-347.749353	-347.847668
M06-D3	-347.803011	-347.695182	-347.789456	-347.751178	-347.849463
M06HF	-347.980237	-347.870674	-347.974747	-347.934519	-348.030202
M06HF-D3	-347.981248	-347.871741	-347.975777	-347.935511	-348.031188
M062X	-347.896506	-347.786830	-347.886527	-347.845832	-347.943853
M062X-D3	-347.896985	-347.787337	-347.887047	-347.846267	-347.944295
M05	-347.788055	-347.679991	-347.771617	-347.729684	-347.834143
M05-D3	-347.793183	-347.684902	-347.776474	-347.735093	-347.839603
M052X	-348.007098	-347.897535	-347.997806	-347.960726	-348.059006
M052X-D3	-348.008252	-347.898749	-347.998970	-347.961869	-348.060136
TPSSH	-348.061069	-347.954949	-348.047740	-348.017053	-348.111663
CAM-B3LYP	-347.865815	-347.754693	-347.853767	-347.811607	-347.914324
CAM-B3LYP-D3BJ	-347.877807	-347.766754	-347.865743	-347.825515	-347.927773
wB97XD	-347.934614	-347.823658	-347.921260	-347.881052	-347.986441
B2PLYP	-347.737899	-347.627765	-347.724962	-347.686614	-347.783797
mPW2PLYP	-347.736430	-347.624862	-347.723639	-347.684124	-347.783319
DSDPBEP86	-347.378448	-347.267666	-347.365026	-347.328741	-347.429435

Table S71: XC/pc-2 single-point energies on CCSD/6-31G(d,p) optimized geometries for the reaction profile of **hex-5-ynal** (1a) + $\mathbf{H_2O}$.

XC	1	TS_{enol}	2	TS_{conia}	3
CCSD(T)	-384.4419462	-384.3711400	-384.4296563	-384.3639606	-384.4542281
PBE	-384.7207215	-384.6715288	-384.7119694	-384.6624949	-384.7410793
PBE-D3BJ	-384.7370140	-384.6867735	-384.7272245	-384.6790709	-384.7566851
PW91	-385.0503203	-385.0013191	-385.0417408	-384.9918358	-385.0699214
M06L	-385.1273572	-385.0572753	-385.1111967	-385.0486787	-385.1402229
M06L-D3	-385.1279800	-385.0578868	-385.1118848	-385.0492473	-385.1408105
MN12-L	-384.7661285	-384.6985455	-384.7588554	-384.6915942	-384.7782858
MN15-L	-384.8407313	-384.7652990	-384.8267517	-384.7637896	-384.8570213
TPSS	-385.2431734	-385.1871928	-385.2321559	-385.1794621	-385.2621926
TPSS-D3BJ	-385.2635884	-385.2061276	-385.2512098	-385.2001442	-385.2816866
B3LYP	-385.2068765	-385.1442301	-385.1971994	-385.1314238	-385.2203940
B3LYP-D3BJ	-385.2337051	-385.1694855	-385.2223457	-385.1590349	-385.2463212
PBE0	-384.7386917	-384.6810511	-384.7296127	-384.6713194	-384.7601227
PBE0-D3BJ	-384.7528892	-384.6941200	-384.7428665	-384.6856021	-384.7736162
B3PW91	-385.0445371	-384.9874819	-385.0357705	-384.9768427	-385.0643581
B3PW91-D3	-385.0627677	-385.0016867	-385.0513617	-384.9928761	-385.0801397
X3LYP	-385.0314471	-384.9686178	-385.0217657	-384.9557743	-385.0450210
B1LYP	-385.0073859	-384.9418145	-384.9972882	-384.9287092	-385.0201965
mPW1PW91	-385.0912840	-385.0329626	-385.0822826	-385.0227185	-385.1119167
B972	-385.0479878	-384.9865458	-385.0383672	-384.9759285	-385.0670634
MN15	-384.7853019	-384.7192566	-384.7767314	-384.7106531	-384.7992198
M08HX	-385.0956920	-385.0248389	-385.0860917	-385.0157143	-385.1061075
M06	-384.9464839	-384.8796435	-384.9331970	-384.8682857	-384.9570614
M06-D3	-384.9487452	-384.8813560	-384.9352986	-384.8702654	-384.9590111
M06HF	-385.1128057	-385.0532229	-385.1090812	-385.0392905	-385.1225287
M06HF-D3	-385.1139943	-385.0542357	-385.1103148	-385.0403654	-385.1236189
M062X	-385.0332422	-384.9676285	-385.0237002	-384.9548731	-385.0427866
M062X-D3	-385.0337654	-384.9681604	-385.0242823	-384.9553515	-385.0432817
M05	-384.9359933	-384.8670325	-384.9207026	-384.8550384	-384.9526592
M05-D3	-384.9421564	-384.8719467	-384.9262023	-384.8605464	-384.9580728
M052X	-385.1505505	-385.0876361	-385.1420536	-385.0753532	-385.1625871
M052X-D3	-385.1519296	-385.0887746	-385.1434524	-385.0765939	-385.1638357
TPSSH	-385.1992779	-385.1403378	-385.1883241	-385.1322113	-385.2187934
CAM-B3LYP	-385.0131980	-384.9491743	-385.0028907	-384.9339251	-385.0278054
CAM-B3LYP-D3BJ	-385.0259110	-384.9606946	-385.0147567	-384.9465765	-385.0397992
wB97XD	-385.0733106	-385.0046177	-385.0607491	-384.9939160	-385.0898098

Continued on next page

Table S71 (continued)

XC	1	TS_{enol}	2	TS_{conia}	3
B2PLYP	-384.8730233	-384.8072206	-384.8616797	-384.7963161	-384.8842963
mPW2PLYP	-384.8707580	-384.8037293	-384.8593908	-384.7924935	-384.8823453
DSDPBEP86	-384.4872737	-384.4195924	-384.4747305	-384.4104987	-384.5003304
B2PLYP	-384.9322884	-384.8666088	-384.9212959	-384.8559849	-384.9439917
mPW2PLYP	-384.9271121	-384.8602060	-384.9160844	-384.8492409	-384.9391455
DSDPBEP86	-384.5751710	-384.5077069	-384.5630857	-384.4988968	-384.5885996

Table S72: XC/pc-2 single-point energies on CCSD/6-31G(d,p) optimized geometries for the reaction profile of **hept-6-ynal** (1b) + $\mathbf{H_2O}$.

XC	1	TS_{enol}	2	TS_{conia}	3
CCSD(T)	-423.682419	-423.612979	-423.672073	-423.628991	-423.732198
PBE	-423.990675	-423.940867	-423.983752	-423.954874	-424.043432
PBE-D3BJ	-424.008966	-423.960433	-424.002513	-423.975945	-424.063517
PW91	-424.357823	-424.308285	-424.351139	-424.322110	-424.410128
M06L	-424.444866	-424.377733	-424.432566	-424.391264	-424.490483
M06L-D3	-424.445724	-424.378633	-424.433490	-424.392074	-424.491281
MN12-L	-424.035016	-423.971638	-424.032049	-423.988045	-424.085902
MN15-L	-424.123936	-424.053788	-424.114534	-424.073678	-424.178207
TPSS	-424.576178	-424.518480	-424.565990	-424.533641	-424.625706
TPSS-D3BJ	-424.598967	-424.542860	-424.589413	-424.559977	-424.650797
B3LYP	-424.535483	-424.470696	-424.526387	-424.482606	-424.581019
B3LYP-D3BJ	-424.565525	-424.502852	-424.557181	-424.517566	-424.614329
PBE0	-424.016023	-423.957725	-424.008460	-423.971770	-424.072147
PBE0-D3BJ	-424.031865	-423.974674	-424.024790	-423.990020	-424.089533
B3PW91	-424.357961	-424.298802	-424.349776	-424.312482	-424.410785
B3PW91-D3	-424.377504	-424.318570	-424.369108	-424.333533	-424.431342
X3LYP	-424.338039	-424.273523	-424.329240	-424.285333	-424.384224
B1LYP	-424.308216	-424.240364	-424.298535	-424.252125	-424.353137
mPW1PW91	-424.409334	-424.349569	-424.401303	-424.363525	-424.464051
B972	-424.360161	-424.296578	-424.351003	-424.310089	-424.411525
MN15	-424.061257	-423.998299	-424.055993	-424.011874	-424.111748
M08HX	-424.414419	-424.346633	-424.408013	-424.361165	-424.463459
M06	-424.239652	-424.174637	-424.229556	-424.185863	-424.285809
M06-D3	-424.242204	-424.177350	-424.232245	-424.188519	-424.288328
M06HF	-424.431229	-424.372554	-424.428418	-424.383428	-424.479995
M06HF-D3	-424.432724	-424.374144	-424.430015	-424.384900	-424.481427
M062X	-424.338849	-424.275997	-424.332302	-424.286512	-424.385638

Continued on next page

Table S72 (continued)

XC	1	TS_{enol}	2	TS_{conia}	3
M062X-D3	-424.339586	-424.276765	-424.333095	-424.287201	-424.386315
M05	-424.227634	-424.157883	-424.213867	-424.166645	-424.273894
M05-D3	-424.234477	-424.164870	-424.220712	-424.173877	-424.280938
M052X	-424.471271	-424.409955	-424.464923	-424.422908	-424.522712
M052X-D3	-424.472963	-424.411757	-424.466726	-424.424595	-424.524342
TPSSH	-424.529012	-424.468435	-424.518845	-424.483487	-424.580085
CAM-B3LYP	-424.316389	-424.251204	-424.307164	-424.260380	-424.365190
CAM-B3LYP-D3BJ	-424.330492	-424.266310	-424.321810	-424.276634	-424.380662
wB97XD	-424.388176	-424.321136	-424.378437	-424.333574	-424.440211
B2PLYP	-424.159843	-424.093284	-424.149662	-424.106972	-424.205876
mPW2PLYP	-424.157820	-424.090451	-424.147901	-424.103805	-424.204802
DSDPBEP86	-423.732971	-423.666755	-423.722900	-423.681799	-423.783853

5.13 pc-3

Table S73: XC/pc-3 single-point energies on CCSD/6-31G(d,p) optimized geometries for the reaction profile of **hex-5-ynal** (1a).

XC	1	TS_{enol}	2	TS_{conia}	3
CCSD(T)	-308.0874483	-307.9784291	-308.0747491	-308.0112212	-308.1013997
PBE	-308.3426204	-308.2482385	-308.3325311	-308.2858042	-308.3635238
PBE-D3BJ	-308.3555091	-308.2608116	-308.3446150	-308.2996820	-308.3765636
PW91	-308.6132264	-308.5182753	-308.6032670	-308.5561289	-308.6333669
M06L	-308.6973713	-308.5887477	-308.6808627	-308.6209005	-308.7125248
M06L-D3	-308.6977422	-308.5891376	-308.6812715	-308.6212105	-308.7128719
MN12-L	-308.4085450	-308.3016607	-308.4005397	-308.3369600	-308.4241978
MN15-L	-308.4854579	-308.3765925	-308.4720726	-308.4117775	-308.5048863
TPSS	-308.7828757	-308.6822788	-308.7704705	-308.7198946	-308.8023978
TPSS-D3BJ	-308.7990031	-308.6979902	-308.7855546	-308.7372073	-308.8186845
B3LYP	-308.7451864	-308.6388456	-308.7348017	-308.6705028	-308.7590838
B3LYP-D3BJ	-308.7667217	-308.6598690	-308.7550194	-308.6939677	-308.7810218
PBE0	-308.3621331	-308.2592734	-308.3518918	-308.2963736	-308.3846186
PBE0-D3BJ	-308.3732644	-308.2701144	-308.3623059	-308.3082318	-308.3958192
B3PW91	-308.6145244	-308.5117881	-308.6044771	-308.5475802	-308.6348823
B3PW91-D3	-308.6263055	-308.5224983	-308.6143652	-308.5589166	-308.6460975
X3LYP	-308.5982824	-308.4916959	-308.5879347	-308.5236431	-308.6123945
B1LYP	-308.5775826	-308.4689757	-308.5669034	-308.4996562	-308.5908437
mPW1PW91	-308.6533375	-308.5493917	-308.6430999	-308.5859160	-308.6748095

Continued on next page

Table S73 (continued)

XC	1	TS_{enol}	2	TS_{conia}	3
B972	-308.6021539	-308.4971861	-308.5914730	-308.5309316	-308.6215806
MN15	-308.4087677	-308.3047930	-308.4002401	-308.3372077	-308.4253276
M08HX	-308.6613569	-308.5503964	-308.6507077	-308.5842738	-308.6749636
M06	-308.5291093	-308.4230215	-308.5156180	-308.4530956	-308.5410573
M06-D3	-308.5304673	-308.4242774	-308.5168351	-308.4542745	-308.5423472
M06HF	-308.6886020	-308.5816224	-308.6835681	-308.6169285	-308.7008045
M06HF-D3	-308.6893220	-308.5823285	-308.6842738	-308.6175474	-308.7014982
M062X	-308.6040996	-308.4966399	-308.5944273	-308.5288309	-308.6168505
M062X-D3	-308.6044078	-308.4969682	-308.5947781	-308.5290865	-308.6171353
M05	-308.5150655	-308.4097774	-308.4996494	-308.4366959	-308.5323834
M05-D3	-308.5190508	-308.4134268	-308.5030773	-308.4405108	-308.5361987
M052X	-308.6990518	-308.5919178	-308.6901947	-308.6266285	-308.7134538
M052X-D3	-308.6998866	-308.5927229	-308.6909938	-308.6273491	-308.7142579
TPSSH	-308.7477356	-308.6442517	-308.7354831	-308.6815550	-308.7679616
CAM-B3LYP	-308.5622210	-308.4540656	-308.5512424	-308.4843084	-308.5775281
CAM-B3LYP-D3BJ	-308.5871061	-308.4786403	-308.5755705	-308.5096889	-308.6025293
wB97XD	-308.6180604	-308.5097033	-308.6051294	-308.5409802	-308.6366939
B2PLYP	-308.4972248	-308.3901088	-308.4855180	-308.4220460	-308.5094693
mPW2PLYP	-308.4930643	-308.3845596	-308.4814003	-308.4166024	-308.5058067
DSDPBEP86	-308.2007545	-308.0929448	-308.1879991	-308.1263590	-308.2156527

Table S74: XC/pc-3 single-point energies on CCSD/6-31G(d,p) optimized geometries for the reaction profile of **hept-6-ynal** (1b).

XC	1	TS_{enol}	2	TS_{conia}	3
CCSD(T)	-347.328943	-347.217226	-347.315776	-347.276947	-347.378884
PBE	-347.615117	-347.518536	-347.604301	-347.580733	-347.667281
PBE-D3BJ	-347.630814	-347.534215	-347.619850	-347.598968	-347.684910
PW91	-347.923257	-347.826111	-347.912606	-347.888968	-347.974974
M06L	-348.020892	-347.910717	-348.004675	-347.968260	-348.067139
M06L-D3	-348.021455	-347.911312	-348.005277	-347.968782	-348.067668
MN12-L	-347.685854	-347.577009	-347.678378	-347.639373	-347.737021
MN15-L	-347.777478	-347.666708	-347.764621	-347.727522	-347.831751
TPSS	-348.118161	-348.014989	-348.104701	-348.076977	-348.167323
TPSS-D3BJ	-348.137720	-348.034542	-348.124103	-348.099769	-348.189354
B3LYP	-348.075859	-347.966657	-348.064209	-348.024474	-348.121030
B3LYP-D3BJ	-348.101893	-347.992667	-348.090051	-348.055092	-348.150569
PBE0	-347.642177	-347.536654	-347.631113	-347.599515	-347.697948

Continued on next page

Table S74 (continued)

XC	1	TS_{enol}	2	TS_{conia}	3
PBE0-D3BJ	-347.655702	-347.550199	-347.644549	-347.615211	-347.713130
B3PW91	-347.930131	-347.824670	-347.918906	-347.885967	-347.982471
B3PW91-D3	-347.945026	-347.838686	-347.932787	-347.901871	-347.998730
X3LYP	-347.907084	-347.797645	-347.895582	-347.855973	-347.952960
B1LYP	-347.880461	-347.768843	-347.868442	-347.825882	-347.925092
mPW1PW91	-347.973821	-347.867121	-347.962562	-347.929456	-348.028150
B972	-347.933280	-347.825519	-347.921378	-347.884580	-347.984347
MN15	-347.691073	-347.584823	-347.682586	-347.643212	-347.741876
M08HX	-347.984335	-347.871256	-347.973753	-347.932391	-348.033905
M06	-347.827161	-347.718982	-347.813525	-347.774461	-347.873146
M06-D3	-347.828957	-347.720834	-347.815280	-347.776286	-347.874941
M06HF	-348.012855	-347.902690	-348.006945	-347.966917	-348.063204
M06HF-D3	-348.013866	-347.903757	-348.007975	-347.967909	-348.064190
M062X	-347.913632	-347.803834	-347.903869	-347.862980	-347.961340
M062X-D3	-347.914112	-347.804340	-347.904388	-347.863415	-347.961782
M05	-347.809865	-347.702031	-347.793632	-347.751434	-347.855691
M05-D3	-347.814994	-347.706942	-347.798488	-347.756843	-347.861152
M052X	-348.023103	-347.913361	-348.013913	-347.976830	-348.075358
M052X-D3	-348.024257	-347.914575	-348.015077	-347.977973	-348.076488
TPSSH	-348.079864	-347.973655	-348.066547	-348.035778	-348.130648
CAM-B3LYP	-347.882775	-347.771595	-347.870834	-347.828509	-347.931388
CAM-B3LYP-D3BJ	-347.894767	-347.783656	-347.882810	-347.842417	-347.944838
wB97XD	-347.952250	-347.841382	-347.938991	-347.898691	-348.004113
B2PLYP	-347.790452	-347.680657	-347.777793	-347.739426	-347.836511
mPW2PLYP	-347.786388	-347.675153	-347.773870	-347.734332	-347.833455
DSDPBEP86	-347.456549	-347.346336	-347.443485	-347.407196	-347.507658

Table S75: XC/pc-3 single-point energies on CCSD/6-31G(d,p) optimized geometries for the reaction profile of **hex-5-ynal** (1a) + $\mathbf{H_2O}$.

XC	1	TS_{enol}	2	TS_{conia}	3
CCSD(T)	-384.4419462	-384.3711400	-384.4296563	-384.3639606	-384.4542281
PBE	-384.7207215	-384.6715288	-384.7119694	-384.6624949	-384.7410793
PBE-D3BJ	-384.7370140	-384.6867735	-384.7272245	-384.6790709	-384.7566851
PW91	-385.0503203	-385.0013191	-385.0417408	-384.9918358	-385.0699214
M06L	-385.1273572	-385.0572753	-385.1111967	-385.0486787	-385.1402229
M06L-D3	-385.1279800	-385.0578868	-385.1118848	-385.0492473	-385.1408105
MN12-L	-384.7661285	-384.6985455	-384.7588554	-384.6915942	-384.7782858

Continued on next page

Table S75 (continued)

XC	1	TS_{enol}	2	TS_{conia}	3
MN15-L	-384.8407313	-384.7652990	-384.8267517	-384.7637896	-384.8570213
TPSS	-385.2431734	-385.1871928	-385.2321559	-385.1794621	-385.2621926
TPSS-D3BJ	-385.2635884	-385.2061276	-385.2512098	-385.2001442	-385.2816866
B3LYP	-385.2068765	-385.1442301	-385.1971994	-385.1314238	-385.2203940
B3LYP-D3BJ	-385.2337051	-385.1694855	-385.2223457	-385.1590349	-385.2463212
PBE0	-384.7386917	-384.6810511	-384.7296127	-384.6713194	-384.7601227
PBE0-D3BJ	-384.7528892	-384.6941200	-384.7428665	-384.6856021	-384.7736162
B3PW91	-385.0445371	-384.9874819	-385.0357705	-384.9768427	-385.0643581
B3PW91-D3	-385.0627677	-385.0016867	-385.0513617	-384.9928761	-385.0801397
X3LYP	-385.0314471	-384.9686178	-385.0217657	-384.9557743	-385.0450210
B1LYP	-385.0073859	-384.9418145	-384.9972882	-384.9287092	-385.0201965
mPW1PW91	-385.0912840	-385.0329626	-385.0822826	-385.0227185	-385.1119167
B972	-385.0479878	-384.9865458	-385.0383672	-384.9759285	-385.0670634
MN15	-384.7853019	-384.7192566	-384.7767314	-384.7106531	-384.7992198
M08HX	-385.0956920	-385.0248389	-385.0860917	-385.0157143	-385.1061075
M06	-384.9464839	-384.8796435	-384.9331970	-384.8682857	-384.9570614
M06-D3	-384.9487452	-384.8813560	-384.9352986	-384.8702654	-384.9590111
M06HF	-385.1128057	-385.0532229	-385.1090812	-385.0392905	-385.1225287
M06HF-D3	-385.1139943	-385.0542357	-385.1103148	-385.0403654	-385.1236189
M062X	-385.0332422	-384.9676285	-385.0237002	-384.9548731	-385.0427866
M062X-D3	-385.0337654	-384.9681604	-385.0242823	-384.9553515	-385.0432817
M05	-384.9359933	-384.8670325	-384.9207026	-384.8550384	-384.9526592
M05-D3	-384.9421564	-384.8719467	-384.9262023	-384.8605464	-384.9580728
M052X	-385.1505505	-385.0876361	-385.1420536	-385.0753532	-385.1625871
M052X-D3	-385.1519296	-385.0887746	-385.1434524	-385.0765939	-385.1638357
TPSSH	-385.1992779	-385.1403378	-385.1883241	-385.1322113	-385.2187934
CAM-B3LYP	-385.0131980	-384.9491743	-385.0028907	-384.9339251	-385.0278054
CAM-B3LYP-D3BJ	-385.0259110	-384.9606946	-385.0147567	-384.9465765	-385.0397992
wB97XD	-385.0733106	-385.0046177	-385.0607491	-384.9939160	-385.0898098
B2PLYP	-384.8730233	-384.8072206	-384.8616797	-384.7963161	-384.8842963
mPW2PLYP	-384.8707580	-384.8037293	-384.8593908	-384.7924935	-384.8823453
DSDPBEP86	-384.4872737	-384.4195924	-384.4747305	-384.4104987	-384.5003304
B2PLYP	-384.9322884	-384.8666088	-384.9212959	-384.8559849	-384.9439917
mPW2PLYP	-384.9271121	-384.8602060	-384.9160844	-384.8492409	-384.9391455
DSDPBEP86	-384.5751710	-384.5077069	-384.5630857	-384.4988968	-384.5885996

Table S76: XC/pc-3 single-point energies on CCSD/6-31G(d,p) optimized geometries for the reaction profile of **hept-6-ynal** (1b) + $\mathbf{H_2O}$.

XC	1	TS_{enol}	2	TS_{conia}	3
CCSD(T)	-423.682419	-423.612979	-423.672073	-423.628991	-423.732198
PBE	-424.011763	-423.961435	-424.004560	-423.975499	-424.064491
PBE-D3BJ	-424.030053	-423.981001	-424.023321	-423.996569	-424.084576
PW91	-424.378933	-424.328824	-424.371953	-424.342747	-424.431188
M06L	-424.485290	-424.417726	-424.472618	-424.431024	-424.531299
M06L-D3	-424.486148	-424.418626	-424.473542	-424.431834	-424.532097
MN12-L	-424.083994	-424.019976	-424.080761	-424.036250	-424.134999
MN15-L	-424.181836	-424.110370	-424.172139	-424.129962	-424.235332
TPSS	-424.599305	-424.541063	-424.588787	-424.556408	-424.648923
TPSS-D3BJ	-424.622094	-424.565442	-424.612210	-424.582743	-424.674014
B3LYP	-424.556946	-424.491799	-424.547650	-424.503766	-424.602596
B3LYP-D3BJ	-424.586988	-424.523955	-424.578445	-424.538726	-424.635906
PBE0	-424.037860	-423.979274	-424.030086	-423.993321	-424.094061
PBE0-D3BJ	-424.053702	-423.996223	-424.046416	-424.011571	-424.111447
B3PW91	-424.379382	-424.319942	-424.370985	-424.333660	-424.432307
B3PW91-D3	-424.398925	-424.339710	-424.390318	-424.354711	-424.452864
X3LYP	-424.359501	-424.294620	-424.350503	-424.306487	-424.405799
B1LYP	-424.329778	-424.261582	-424.319901	-424.273401	-424.374846
mPW1PW91	-424.431010	-424.370952	-424.422756	-424.384946	-424.485827
B972	-424.383423	-424.319541	-424.373999	-424.333078	-424.435007
MN15	-424.103624	-424.040292	-424.098454	-424.053990	-424.154054
M08HX	-424.437449	-424.368992	-424.430794	-424.383973	-424.486832
M06	-424.270962	-424.205447	-424.260486	-424.216194	-424.316618
M06-D3	-424.273514	-424.208160	-424.263175	-424.218850	-424.319137
M06HF	-424.471301	-424.411629	-424.468249	-424.423589	-424.520519
M06HF-D3	-424.472796	-424.413220	-424.469846	-424.425060	-424.521951
M062X	-424.361113	-424.297739	-424.354595	-424.308774	-424.408244
M062X-D3	-424.361850	-424.298506	-424.355389	-424.309463	-424.408921
M05	-424.253564	-424.183923	-424.239771	-424.192300	-424.299566
M05-D3	-424.260407	-424.190909	-424.246617	-424.199532	-424.306610
M052X	-424.491734	-424.430012	-424.485404	-424.443511	-424.543542
M052X-D3	-424.493426	-424.431815	-424.487207	-424.445197	-424.545172
TPSSH	-424.552412	-424.491402	-424.541955	-424.506599	-424.603611
CAM-B3LYP	-424.337771	-424.272211	-424.328385	-424.281489	-424.386638
CAM-B3LYP-D3BJ	-424.351874	-424.287317	-424.343031	-424.297743	-424.402110
wB97XD	-424.410156	-424.343030	-424.400350	-424.355466	-424.462218

Continued on next page

Table S76 (continued)

XC	1	TS_{enol}	2	TS_{conia}	3
B2PLYP	-424.225420	-424.158739	-424.215183	-424.172531	-424.271581
mPW2PLYP	-424.220188	-424.152693	-424.210208	-424.166142	-424.267309
DSDPBEP86	-423.830024	-423.763874	-423.820013	-423.778993	-423.881015

6 XYZ coordinates

6.1 Hex-5-ynal gas phase

Hex-5-ynal (1a)			
Theory level: CCSD/6-31G(d,p)			
Energy [Hartrees]	-307.706464		
No. Im. Freq.	0		
Atom	x	y	z
C	0.6012	0.9602	-0.9011
H	0.8194	0.1349	-1.6031
O	1.4520	1.7818	-0.6207
C	-0.8254	1.0174	-0.3929
H	-1.4028	1.5250	-1.1740
H	-0.8466	1.6580	0.4902
C	-1.4625	-0.3486	-0.1190
H	-2.5430	-0.2227	-0.0229
H	-1.2987	-1.0101	-0.9746
C	-0.9456	-1.0328	1.1561
H	-1.5122	-1.9507	1.3292
H	-1.1301	-0.3829	2.0158
C	0.4889	-1.3586	1.0991
C	1.6692	-1.6324	1.0388
H	2.7089	-1.8511	0.9866

TS_{enol} of hex-5-ynal			
Theory level: CCSD/6-31G(d,p)			
Energy [Hartrees]	-307.586651		
No. Im. Freq.	1		
Atom	x	y	z
C	-0.9079	0.9465	-0.6949
H	-0.0955	0.2270	-0.7640

Continued on next page

Table (continued)

Atom	x	y	z
O	-0.7990	2.0871	-1.2786
C	-2.1816	0.8429	-0.0749
H	-2.0280	2.0425	-0.9676
H	-2.2457	1.5534	0.7511
C	-2.8893	-0.4715	0.1669
H	-3.9658	-0.2898	0.2194
H	-2.7337	-1.1415	-0.6822
C	-2.4637	-1.1979	1.4588
H	-3.0749	-2.0930	1.6015
H	-2.6497	-0.5476	2.3178
C	-1.0430	-1.5858	1.4501
C	0.1257	-1.9120	1.4211
H	1.1487	-2.2033	1.4162

(*E*)-hex-1-en-5-yn-1-ol (2a)

Theory level: CCSD/6-31G(d,p)

Energy [Hartrees]	-307.688817
No. Im. Freq.	0

Atom	x	y	z
C	0.1718	1.0574	-1.3385
H	0.4726	0.2798	-2.0284
O	0.8245	2.2301	-1.6130
C	-0.7237	0.8511	-0.3675
H	0.5490	2.8906	-0.9673
H	-0.9854	1.6646	0.3048
C	-1.3789	-0.4803	-0.1309
H	-2.4677	-0.3719	-0.1456
H	-1.1179	-1.1688	-0.9380
C	-0.9818	-1.1175	1.2152
H	-1.5628	-2.0297	1.3729
H	-1.2396	-0.4364	2.0308
C	0.4520	-1.4399	1.2886
C	1.6310	-1.7213	1.3345
H	2.6678	-1.9545	1.3755

TS_{conia} of (*E*)-hex-1-en-5-yn-1-ol			
Theory level: CCSD/6-31G(d,p)			
Energy [Hartrees]		-307.615661	
No. Im. Freq.		1	
Atom	x	y	z
C	-0.4201	1.5798	0.1994
H	-0.5755	1.9639	-0.8126
O	0.5779	2.0505	0.8980
C	-1.0336	0.4139	0.5958
H	1.3003	1.1236	0.8749
H	-1.0143	0.1528	1.6469
C	-1.6718	-0.5690	-0.3667
H	-2.6291	-0.9812	-0.0489
H	-1.7971	-0.1127	-1.3501
C	-0.5107	-1.5878	-0.3746
H	-0.2823	-2.0373	-1.3430
H	-0.6831	-2.3961	0.3400
C	0.6045	-0.7126	0.1335
C	1.7005	-0.1912	0.4857
H	2.7454	-0.4403	0.5133

2-Methylenecyclobutane-1-carbaldehyde (3a)			
Theory level: CCSD/6-31G(d,p)			
Energy [Hartrees]		-307.722883	
No. Im. Freq.		0	
Atom	x	y	z
C	-0.4516	1.6011	-0.0100
H	-0.2634	1.7738	-1.0868
O	-0.4721	2.5295	0.7760
C	-0.6376	0.1567	0.3718
H	2.2832	0.3166	0.6313
H	-0.9753	0.0945	1.4078
C	-1.4608	-0.7409	-0.6019
H	-2.4545	-1.0329	-0.2674
H	-1.5398	-0.2865	-1.5913
C	-0.3391	-1.8156	-0.5487
H	-0.0157	-2.2518	-1.4939
H	-0.5722	-2.6227	0.1491
C	0.5548	-0.7562	0.0745
C	1.8708	-0.5817	0.1897

Continued on next page

Table (continued)

Atom	x	y	z
H	2.5711	-1.3339	-0.1499

6.2 Hex-5-ynal implicit solvent

Hex-5-ynal (1a)			
Theory level: CCSD-SMD(H_2O)/6-31G(d,p)			
Energy [Hartrees]	-307.713442		
No. Im. Freq.	0		
Atom	x	y	z
C	0.5928	0.9360	-0.8921
H	0.7945	0.1543	-1.6405
O	1.4801	1.7116	-0.5575
C	-0.8240	1.0134	-0.3925
H	-1.3851	1.5123	-1.1906
H	-0.8528	1.6579	0.4874
C	-1.4671	-0.3492	-0.1182
H	-2.5444	-0.2128	-0.0145
H	-1.3084	-1.0091	-0.9749
C	-0.9496	-1.0299	1.1570
H	-1.5077	-1.9527	1.3274
H	-1.1357	-0.3812	2.0162
C	0.4885	-1.3454	1.0982
C	1.6747	-1.5986	1.0300
H	2.7167	-1.8192	0.9721

TS_{enol} of hex-5-ynal			
Theory level: CCSD-SMD(H_2O)/6-31G(d,p)			
Energy [Hartrees]	-307.590491		
No. Im. Freq.	1		
Atom	x	y	z
C	-0.9287	0.9327	-0.7693
H	-0.1752	0.1822	-0.9953
O	-0.8061	2.1308	-1.2566
C	-2.1395	0.8255	-0.0556
H	-2.0278	2.0708	-0.8472
H	-2.1408	1.5099	0.7948

Continued on next page

Table (continued)

Atom	x	y	z
C	-2.8744	-0.4768	0.1598
H	-3.9494	-0.2849	0.1959
H	-2.7036	-1.1421	-0.6895
C	-2.4827	-1.2062	1.4584
H	-3.0900	-2.1066	1.5762
H	-2.6962	-0.5616	2.3145
C	-1.0580	-1.5797	1.4841
C	0.1184	-1.8839	1.4818
H	1.1504	-2.1528	1.4889

(*E*)-hex-1-en-5-yn-1-ol (2a)			
Theory level: CCSD-SMD(H_2O)/6-31G(d,p)			
Energy [Hartrees]		-307.697276	
No. Im. Freq.		0	
**Atom**	**x**	**y**	**z**
C	0.1755	1.0402	-1.3358
H	0.4691	0.2664	-2.0337
O	0.8409	2.2253	-1.6022
C	-0.7191	0.8442	-0.3626
H	0.5554	2.8871	-0.9577
H	-0.9671	1.6613	0.3093
C	-1.3857	-0.4826	-0.1317
H	-2.4724	-0.3625	-0.1362
H	-1.1335	-1.1710	-0.9413
C	-0.9908	-1.1208	1.2131
H	-1.5633	-2.0381	1.3656
H	-1.2502	-0.4429	2.0298
C	0.4467	-1.4332	1.2849
C	1.6321	-1.6961	1.3258
H	2.6733	-1.9235	1.3659

TS_{conia} of (*E*)-hex-1-en-5-yn-1-ol			
Theory level: CCSD-SMD(H_2O)/6-31G(d,p)			
Energy [Hartrees]		-307.621756	
No. Im. Freq.		1	
**Atom**	**x**	**y**	**z**
C	-0.4245	1.5653	0.1937

Continued on next page

Table (continued)

Atom	x	y	z
H	-0.5436	1.9245	-0.8297
O	0.5725	2.0538	0.9155
C	-1.0610	0.4253	0.6031
H	1.3153	1.0953	0.8603
H	-1.0299	0.1539	1.6520
C	-1.6783	-0.5646	-0.3628
H	-2.6252	-0.9931	-0.0389
H	-1.8093	-0.1078	-1.3443
C	-0.5123	-1.5749	-0.3771
H	-0.2896	-2.0160	-1.3496
H	-0.6749	-2.3829	0.3385
C	0.6188	-0.7174	0.1193
C	1.7026	-0.1807	0.4844
H	2.7503	-0.4244	0.5270

2-Methylenecyclobutane-1-carbaldehyde (3a)			
Theory level: CCSD-SMD(H_2O)/6-31G(d,p)			
Energy [Hartrees]	-307.726563		
No. Im. Freq.	0		
Atom	x	y	z
C	-0.4439	1.5863	-0.0080
H	-0.2399	1.7663	-1.0754
O	-0.4722	2.5215	0.7830
C	-0.6425	0.1530	0.3758
H	2.2741	0.3336	0.6132
H	-0.9860	0.0842	1.4089
C	-1.4590	-0.7397	-0.6071
H	-2.4551	-1.0261	-0.2760
H	-1.5240	-0.2812	-1.5947
C	-0.3434	-1.8183	-0.5427
H	-0.0151	-2.2606	-1.4830
H	-0.5839	-2.6174	0.1612
C	0.5511	-0.7591	0.0773
C	1.8668	-0.5721	0.1806
H	2.5706	-1.3202	-0.1630

6.3 Hept-6-ynal gas phase

Hept-6-ynal (1b)			
Theory level: CCSD/6-31G(d,p)			
Energy [Hartrees]	-346.898783		
No. Im. Freq.	0		
Atom	x	y	z
C	-1.1606	0.2131	-0.6889
H	-1.5917	-0.7201	-1.0675
H	-1.9635	0.9557	-0.7152
C	-0.7231	0.0194	0.7702
H	0.0408	-0.7581	0.8347
C	-0.0538	0.6745	-1.6438
H	-0.5199	0.9209	-2.6015
H	0.4002	1.5952	-1.2692
C	1.0522	-0.3595	-1.9226
H	0.5969	-1.3289	-2.1459
H	1.5945	-0.0604	-2.8229
C	-1.8918	-0.3960	1.6293
H	-2.4016	-1.3250	1.3087
C	2.0347	-0.5217	-0.8366
C	2.8768	-0.6420	0.0285
H	3.5992	-0.7480	0.8020
O	-2.2863	0.2167	2.6039
H	-0.3046	0.9389	1.1814

TS_{enol} of hept-6-ynal			
Theory level: CCSD/6-31G(d,p)			
Energy [Hartrees]	-346.776308		
No. Im. Freq.	1		
Atom	x	y	z
C	-1.1974	-0.0634	-0.8575
H	-1.3876	-1.0466	-1.3069
H	-2.1238	0.5078	-0.9693
C	-0.8715	-0.1767	0.6176
H	-0.0595	-0.8532	0.8765
C	-0.1127	0.6439	-1.6833
H	-0.5376	0.9103	-2.6553
H	0.1720	1.5753	-1.1894
C	1.1458	-0.1959	-1.9620
H	0.8482	-1.1533	-2.4000
H	1.7488	0.3137	-2.7181

Continued on next page

Table (continued)

Atom	x	y	z
C	-1.8499	-0.1640	1.6380
H	-2.9032	-0.4421	1.6105
C	2.0063	-0.4516	-0.7933
C	2.7579	-0.6591	0.1363
H	3.3969	-0.8356	0.9680
O	-1.3555	0.3109	2.7286
H	-0.4604	0.5666	1.8480

(E)-hex-1-en-5-yn-1-ol (2a)			
Theory level: CCSD/6-31G(d,p)			
Energy [Hartrees]		-346.880788	
No. Im. Freq.		0	
Atom	x	y	z
C	-1.2371	-0.2066	-0.9080
H	-1.3877	-1.1933	-1.3610
H	-2.1895	0.3222	-1.0072
C	-0.9203	-0.3556	0.5560
H	-0.0474	-0.9361	0.8322
C	-0.1820	0.5509	-1.7309
H	-0.6097	0.7689	-2.7138
H	0.0336	1.5105	-1.2556
C	1.1338	-0.2097	-1.9703
H	0.9083	-1.2115	-2.3476
H	1.6950	0.2966	-2.7599
C	-1.6650	0.2122	1.5103
H	-2.5446	0.8054	1.2941
C	2.0170	-0.3253	-0.7960
C	2.7882	-0.4090	0.1369
H	3.4501	-0.4794	0.9664
O	-1.4509	0.1394	2.8634
H	-0.6550	-0.3828	3.0143

TS_{conia} of (*E*)-hept-1-en-6-yn-1-ol			
Theory level: CCSD/6-31G(d,p)			
Energy [Hartrees]	-346.833305		
No. Im. Freq.	1		
Atom	x	y	z
C	-1.4555	-0.2541	-1.1161
H	-1.6910	-1.1714	-1.6596
H	-2.3750	0.3335	-1.0591
C	-0.9331	-0.5887	0.2586
H	-0.6330	-1.6059	0.4704
C	-0.3633	0.5359	-1.8648
H	-0.5179	0.5286	-2.9457
H	-0.3838	1.5774	-1.5339
C	0.9952	-0.0651	-1.4977
H	1.0688	-1.0839	-1.8901
H	1.8157	0.5136	-1.9280
C	-1.0466	0.2703	1.3265
H	-1.5264	1.2452	1.1989
C	1.1744	-0.0992	-0.0214
C	1.7524	-0.0376	1.0971
H	2.7402	0.0888	1.4992
O	-0.3888	0.0523	2.4332
H	0.6853	-0.0767	2.0500

2-Methylenecyclopentane-1-carbaldehyde (3b)			
Theory level: CCSD/6-31G(d,p)			
Energy [Hartrees]	-346.951441		
No. Im. Freq.	0		
Atom	x	y	z
C	-1.3402	-0.3271	-1.0611
H	-1.4489	-1.2966	-1.5520
H	-2.3408	0.0996	-0.9642
C	-0.6405	-0.5243	0.3041
H	-0.7933	-1.5226	0.7171
C	-0.3833	0.5616	-1.8721
H	-0.5647	0.5080	-2.9460
H	-0.4946	1.6070	-1.5704
C	1.0046	0.0511	-1.4619
H	1.2350	-0.8687	-2.0097
H	1.8097	0.7609	-1.6544

Continued on next page

Table (continued)

Atom	x	y	z
C	-1.1163	0.4691	1.3410
H	-1.2971	1.4941	0.9641
C	0.8420	-0.2566	0.0135
C	1.7987	-0.2411	0.9470
H	2.8288	-0.0212	0.6978
O	-1.2734	0.2024	2.5174
H	1.5763	-0.4516	1.9853

6.4 Hept-6-ynal implicit solvent

Hept-6-ynal (1b)			
Theory level: CCSD-SMD(H_2O)/6-31G(d,p)			
Energy [Hartrees]		-346.904502	
No. Im. Freq.		0	
Atom	x	y	z
C	-1.1704	0.2233	-0.6964
H	-1.6404	-0.6794	-1.0979
H	-1.9400	1.0003	-0.6846
C	-0.7205	-0.0430	0.7471
H	-0.0359	-0.8940	0.7778
C	-0.0543	0.6721	-1.6432
H	-0.5113	0.9094	-2.6066
H	0.4005	1.5942	-1.2727
C	1.0467	-0.3715	-1.9008
H	0.5910	-1.3507	-2.0703
H	1.5748	-0.1095	-2.8202
C	-1.8971	-0.3900	1.6102
H	-2.5083	-1.2390	1.2656
C	2.0475	-0.4826	-0.8237
C	2.8993	-0.5609	0.0389
H	3.6325	-0.6332	0.8102
O	-2.2018	0.2054	2.6374
H	-0.2139	0.8238	1.1737

TS_{enol} of hept-6-ynal			
Theory level: CCSD-SMD(H_2O)/6-31G(d,p)			
Energy [Hartrees]	-346.780614		
No. Im. Freq.	1		
Atom	x	y	z
C	-1.2219	-0.0899	-0.8805
H	-1.4357	-1.0431	-1.3767
H	-2.1346	0.5087	-0.9491
C	-0.9001	-0.2993	0.5842
H	-0.2370	-1.1275	0.8308
C	-0.1279	0.6330	-1.6775
H	-0.5381	0.8979	-2.6553
H	0.1387	1.5670	-1.1770
C	1.1430	-0.1928	-1.9358
H	0.8627	-1.1721	-2.3328
H	1.7338	0.3004	-2.7112
C	-1.8274	-0.0793	1.6170
H	-2.9151	-0.0673	1.5896
C	2.0130	-0.3831	-0.7605
C	2.7682	-0.5247	0.1805
H	3.4121	-0.6542	1.0209
O	-1.2107	0.2203	2.7240
H	-0.3062	0.2942	1.7976

(*E*)-hex-1-en-5-yn-1-ol (2a)			
Theory level: CCSD-SMD(H_2O)/6-31G(d,p)			
Energy [Hartrees]	-346.888245		
No. Im. Freq.	0		
Atom	x	y	z
C	-1.2751	-0.1760	-0.9397
H	-1.4916	-1.1343	-1.4236
H	-2.1906	0.4187	-1.0010
C	-0.9443	-0.4079	0.5108
H	-0.1615	-1.1168	0.7579
C	-0.1871	0.5405	-1.7533
H	-0.5881	0.7370	-2.7508
H	0.0333	1.5100	-1.2999
C	1.1206	-0.2481	-1.9349
H	0.8894	-1.2754	-2.2290
H	1.6881	0.1916	-2.7582

Continued on next page

Table (continued)

Atom	x	y	z
C	-1.5808	0.2282	1.4989
H	-2.3744	0.9453	1.3304
C	1.9964	-0.2709	-0.7488
C	2.7533	-0.2726	0.2016
H	3.3989	-0.2780	1.0506
O	-1.3336	0.0728	2.8544
H	-0.6165	-0.5672	2.9577

TS_{conia} of (*E*)-hept-1-en-6-yn-1-ol			
Theory level: CCSD-SMD(H_2O)/6-31G(d,p)			
Energy [Hartrees]		-346.838604	
No. Im. Freq.		1	
Atom	x	y	z
C	-1.4660	-0.2647	-1.1225
H	-1.6793	-1.1832	-1.6715
H	-2.3888	0.3175	-1.0772
C	-0.9664	-0.5998	0.2583
H	-0.6514	-1.6145	0.4688
C	-0.3698	0.5387	-1.8476
H	-0.5164	0.5424	-2.9288
H	-0.3960	1.5746	-1.5014
C	0.9905	-0.0634	-1.4915
H	1.0588	-1.0844	-1.8758
H	1.8057	0.5167	-1.9298
C	-1.0433	0.2756	1.3068
H	-1.4777	1.2675	1.1678
C	1.1991	-0.0887	-0.0230
C	1.7514	-0.0326	1.1092
H	2.7408	0.0840	1.5169
O	-0.3932	0.0392	2.4342
H	0.7193	-0.0608	2.0247

2-Methylenecyclopentane-1-carbaldehyde (3b)			
Theory level: CCSD-SMD(H_2O)/6-31G(d,p)			
Energy [Hartrees]	-346.955333		
No. Im. Freq.	0		
Atom	x	y	z
C	-1.3415	-0.3340	-1.0605
H	-1.4377	-1.3051	-1.5497
H	-2.3441	0.0854	-0.9588
C	-0.6410	-0.5312	0.3039
H	-0.7954	-1.5271	0.7211
C	-0.3900	0.5635	-1.8653
H	-0.5709	0.5107	-2.9392
H	-0.5071	1.6055	-1.5557
C	1.0000	0.0573	-1.4602
H	1.2305	-0.8608	-2.0103
H	1.8032	0.7702	-1.6480
C	-1.1049	0.4747	1.3210
H	-1.2327	1.5051	0.9530
C	0.8417	-0.2589	0.0128
C	1.7982	-0.2426	0.9475
H	2.8279	-0.0178	0.6989
O	-1.3131	0.2102	2.4992
H	1.5789	-0.4611	1.9857

6.5 Hex-5-ynal + H_2O gas phase

Hex-5-ynal (1a) + H_2O			
Theory level: CCSD/6-31G(d,p)			
Energy [Hartrees]	-383.949853		
No. Im. Freq.	0		
Atom	x	y	z
C	0.3327	0.7221	-1.4506
H	0.5677	-0.2056	-2.0013
O	1.1759	1.5968	-1.3423
C	-1.0785	0.8235	-0.9235
H	-1.7220	0.9246	-1.8049
H	-1.1652	1.7323	-0.3301
C	-1.5356	-0.4016	-0.1202
H	-2.6194	-0.3495	0.0009

Continued on next page

Table (continued)

Atom	x	y	z
H	-1.3246	-1.3182	-0.6785
C	-0.9032	-0.4925	1.2780
H	-1.3868	-1.2927	1.8426
H	-1.0786	0.4422	1.8132
C	0.5473	-0.7472	1.2426
C	1.7421	-0.9595	1.2105
H	2.7882	-1.1496	1.1705
O	0.6065	2.5316	1.4114
H	0.8775	2.5765	0.4859
H	1.1406	1.8079	1.7537

TS_{enol}(1W) of hex-5-ynal

Theory level: CCSD/6-31G(d,p)

Energy [Hartrees] -383.8681879

No. Im. Freq. 1

Atom	x	y	z
C	-1.0611	1.6994	-0.6496
H	-2.0140	1.3769	-1.0892
O	-0.8194	2.9540	-0.6733
C	-0.1195	0.7264	-0.2565
H	0.4430	0.9806	0.6414
H	0.9241	1.4676	-1.0312
C	-0.4755	-0.7389	-0.3786
H	-1.1623	-0.8795	-1.2188
H	0.4149	-1.3286	-0.6103
C	-1.1312	-1.3274	0.8861
H	-1.4116	-2.3695	0.7108
H	-2.0530	-0.7814	1.1043
C	-0.2454	-1.2615	2.0607
C	0.5013	-1.2060	3.0155
H	1.1453	-1.1630	3.8608
O	1.4344	2.4995	-1.2783
H	0.4735	2.9628	-0.9222
H	2.1209	2.6296	-0.6135

(E)-hex-1-en-5-yn-1-ol (2a) + H_2O			
Theory level: CCSD/6-31G(d,p)			
Energy [Hartrees]	-383.932422		
No. Im. Freq.	0		
Atom	x	y	z
C	-0.2189	0.9464	-1.5829
H	0.0353	0.1693	-2.2953
O	0.3824	2.1340	-1.8835
C	-1.0375	0.7189	-0.5468
H	-1.2973	1.5380	0.1168
H	1.0025	1.7066	0.9846
C	-1.5495	-0.6471	-0.1933
H	-2.6423	-0.6864	-0.2356
H	-1.1733	-1.3824	-0.9086
C	-1.1211	-1.0599	1.2306
H	-1.4624	-2.0749	1.4471
H	-1.6069	-0.4028	1.9570
C	0.3366	-0.9869	1.4330
C	1.5371	-0.9135	1.6018
H	2.5905	-0.9036	1.7511
O	1.0257	2.6620	0.8456
H	0.4449	2.6386	-1.0569
H	1.9577	2.8939	0.8982

TS_{conia}(1W) of (E)-hex-1-en-5-yn-1-ol			
Theory level: CCSD/6-31G(d,p)			
Energy [Hartrees]	-383.857664		
No. Im. Freq.	1		
Atom	x	y	z
C	-0.5800	1.6340	-0.2182
H	-0.4901	1.7999	-1.2938
O	0.2385	2.2935	0.5754
C	-1.2633	0.5491	0.2683
H	0.9880	1.4156	0.8454
H	-1.4627	0.5033	1.3320
C	-1.6587	-0.6331	-0.5932
H	-2.6537	-1.0335	-0.4029
H	-1.5771	-0.3842	-1.6526
C	-0.5091	-1.5672	-0.1555
H	-0.0789	-2.1867	-0.9448

Continued on next page

Table (continued)

Atom	x	y	z
H	-0.8189	-2.2244	0.6601
C	0.4748	-0.5614	0.3793
C	1.4639	0.0794	0.8339
H	2.4833	-0.1040	1.1204
O	-0.8781	1.8577	3.2640
H	-0.5330	2.2572	2.4535
H	-0.1159	1.3990	3.6287

3a + H_2O			
Theory level: CCSD/6-31G(d,p)			
Energy [Hartrees]		-383.963762	
No. Im. Freq.		0	
Atom	x	y	z
C	-0.6635	1.0278	-0.8162
H	-1.2333	0.7652	-1.7259
O	-0.0555	2.0859	-0.7683
C	-0.6682	-0.0032	0.2799
H	1.3006	2.0498	0.6922
H	-0.4278	0.4676	1.2321
C	-1.8860	-0.9738	0.2331
H	-2.2096	-1.2543	1.2339
H	-2.7543	-0.6321	-0.3321
C	-1.0002	-2.0613	-0.4410
H	-1.1855	-2.1556	-1.5138
H	-1.0239	-3.0576	0.0009
C	0.2102	-1.1976	-0.1180
C	1.5307	-1.3690	-0.0805
H	1.9830	-2.3127	-0.3593
O	1.8996	1.7316	1.3806
H	2.6224	2.3644	1.3865
H	2.1752	-0.5679	0.2555

6.6 Hex-5-ynal + H_2O implicit solvent

Hex-5-ynal (1a) + H_2O			
Theory level: CCSD-SMD(H_2O)/6-31G(d,p)			
Energy [Hartrees]	-383.964316		
No. Im. Freq.	0		
Atom	x	y	z
C	0.3103	0.6944	-1.4462
H	0.5179	-0.2294	-2.0063
O	1.1756	1.5606	-1.3609
C	-1.0773	0.8223	-0.8853
H	-1.7233	0.9830	-1.7553
H	-1.1277	1.7105	-0.2557
C	-1.5613	-0.4253	-0.1374
H	-2.6460	-0.3720	-0.0360
H	-1.3371	-1.3189	-0.7252
C	-0.9624	-0.5685	1.2691
H	-1.4086	-1.4312	1.7675
H	-1.2164	0.3130	1.8623
C	0.5028	-0.7240	1.2620
C	1.7130	-0.8325	1.2430
H	2.7741	-0.9410	1.2207
O	0.9125	2.6743	1.3743
H	1.0000	2.5178	0.4238
H	1.1187	1.8079	1.7437

TS_{enol}(1W) of hex-5-ynal			
Theory level: CCSD-SMD(H_2O)/6-31G(d,p)			
Energy [Hartrees]	-383.8865844		
No. Im. Freq.	1		
Atom	x	y	z
C	-0.3092	1.2456	-1.6443
H	-0.0654	0.4437	-2.3593
O	0.0856	2.4353	-1.9294
C	-0.8741	0.8470	-0.4346
H	-1.3843	1.6188	0.1410
H	0.4931	1.3695	0.1968
C	-1.3334	-0.5750	-0.2177
H	-2.4052	-0.6849	-0.4159
H	-0.8146	-1.2368	-0.9169
C	-1.0854	-1.0779	1.2151
H	-1.4654	-2.0962	1.3248

Continued on next page

Table (continued)

Atom	x	y	z
H	-1.6418	-0.4544	1.9196
C	0.3403	-1.0521	1.5854
C	1.5184	-1.0224	1.8819
H	2.5524	-0.9999	2.1426
O	1.2833	2.1426	0.3625
H	1.0357	2.6793	-0.4400
H	1.0348	2.6589	1.1463

(*E*)-hex-1-en-5-yn-1-ol (2a) + H_2O			
Theory level: CCSD-SMD(H_2O)/6-31G(d,p)			
Energy [Hartrees]	-383.946798		
No. Im. Freq.	0		
Atom	x	y	z
C	-0.1495	0.8834	-1.5200
H	0.0791	0.1088	-2.2428
O	0.4952	2.0712	-1.8163
C	-0.9765	0.6814	-0.4877
H	-1.1925	1.4967	0.1957
H	0.9799	2.2248	1.0985
C	-1.5688	-0.6632	-0.1764
H	-2.6613	-0.6244	-0.2095
H	-1.2463	-1.3924	-0.9231
C	-1.1645	-1.1567	1.2261
H	-1.5895	-2.1459	1.4076
H	-1.5840	-0.4856	1.9795
C	0.2968	-1.2112	1.4051
C	1.5038	-1.2321	1.5434
H	2.5627	-1.2655	1.6669
O	0.9707	3.1298	0.7608
H	0.5418	2.5943	-0.9941
H	1.9067	3.3370	0.6492

TS$_{conia}$(1W) of (*E*)-hex-1-en-5-yn-1-ol			
Theory level: CCSD-SMD(H_2O)/6-31G(d,p)			
Energy [Hartrees]	-383.873039		
No. Im. Freq.	1		
Atom	x	y	z
C	-0.6197	1.6344	-0.2504
H	-0.4724	1.7675	-1.3223
O	0.1640	2.3292	0.5696
C	-1.3305	0.5708	0.2268
H	0.9324	1.4205	0.8750
H	-1.5515	0.5206	1.2859
C	-1.6609	-0.6381	-0.6220
H	-2.6514	-1.0578	-0.4558
H	-1.5434	-0.4050	-1.6806
C	-0.5171	-1.5498	-0.1309
H	-0.0596	-2.1745	-0.8993
H	-0.8450	-2.1936	0.6872
C	0.4585	-0.5475	0.4181
C	1.4052	0.1323	0.9031
H	2.4142	-0.0168	1.2476
O	-0.6117	1.9534	3.3421
H	-0.3966	2.1905	2.4264
H	-0.0858	1.1577	3.4796

3a + H_2O			
Theory level: CCSD-SMD(H_2O)/6-31G(d,p)			
Energy [Hartrees]	-383.975815		
No. Im. Freq.	0		
Atom	x	y	z
C	-0.6736	1.0408	-0.7911
H	-1.1970	0.7870	-1.7252
O	-0.0832	2.1130	-0.6968
C	-0.6970	0.0020	0.2893
H	1.2183	2.1456	0.8092
H	-0.4927	0.4671	1.2526
C	-1.8940	-0.9892	0.2052
H	-2.2562	-1.2603	1.1950
H	-2.7350	-0.6615	-0.4055
C	-0.9674	-2.0695	-0.4211
H	-1.1232	-2.1944	-1.4946

Continued on next page

Table (continued)

Atom	x	y	z
H	-0.9734	-3.0525	0.0496
C	0.2158	-1.1714	-0.0971
C	1.5418	-1.3024	-0.0730
H	2.0245	-2.2322	-0.3485
O	1.8769	2.0409	1.5113
H	2.6550	1.7262	1.0383
H	2.1742	-0.4821	0.2420

6.7 Hept-6-ynal + H_2O gas phase

Hept-6-ynal (1b) + H_2O			
Theory level: CCSD/6-31G(d,p)			
Energy [Hartrees]		-423.139921	
No. Im. Freq.		0	
Atom	x	y	z
C	-0.7455	0.2846	-0.2899
H	-1.4881	-0.2262	-0.9131
H	-1.2547	1.0982	0.2281
C	-0.2073	-0.6829	0.7810
H	0.2477	-1.5601	0.3223
C	0.3330	0.9005	-1.1859
H	-0.1445	1.6716	-1.7964
H	1.0743	1.4062	-0.5634
C	1.0384	-0.0739	-2.1461
H	0.2907	-0.6858	-2.6599
H	1.5482	0.5028	-2.9219
C	-1.3359	-1.1174	1.6790
H	-1.8860	-2.0262	1.3797
C	2.0350	-0.9560	-1.5132
C	2.8872	-1.6658	-1.0210
H	3.6236	-2.2883	-0.5723
O	-1.6922	-0.5049	2.6756
H	-0.7476	1.2659	2.7667
O	-0.2419	2.0421	2.4915
H	-0.5974	2.7640	3.0162
H	0.5435	-0.1704	1.3827

TS_{enol}(1W) of hept-6-ynal			
Theory level: CCSD/6-31G(d,p)			
Energy [Hartrees]		-423.060566	
No. Im. Freq.		1	
Atom	x	y	z
C	-1.1083	-0.0725	-0.8097
H	-1.3738	-0.9874	-1.3528
H	-2.0165	0.5404	-0.7935
C	-0.7179	-0.3904	0.6212
H	-0.1341	-1.2974	0.7657
C	-0.0608	0.6874	-1.6355
H	-0.5251	1.0071	-2.5733
H	0.2395	1.5941	-1.1034
C	1.1934	-0.1234	-2.0029
H	0.8893	-1.0717	-2.4559
H	1.7562	0.4175	-2.7681
C	-1.6399	-0.1186	1.6520
H	-2.5982	0.3539	1.3984
C	2.0999	-0.3965	-0.8746
C	2.8805	-0.6021	0.0310
H	3.5229	-0.7956	0.8561
O	-1.3817	-0.2463	2.8970
H	-0.0628	-0.0732	2.9108
O	0.9159	0.2418	2.4791
H	1.0778	1.1563	2.7426
H	0.3736	0.2175	1.4251

(*E*)-hex-1-en-5-yn-1-ol (2a) + H_2O			
Theory level: CCSD/6-31G(d,p)			
Energy [Hartrees]		-423.125295	
No. Im. Freq.		0	
Atom	x	y	z
C	-1.3351	-0.1598	-1.0634
H	-1.5728	-1.1388	-1.4941
H	-2.2279	0.4582	-1.1950
C	-1.0371	-0.3139	0.4020
H	-0.3440	-1.0937	0.6958
C	-0.1910	0.4641	-1.8790
H	-0.4679	0.4476	-2.9366
H	-0.0673	1.5117	-1.5970

Continued on next page

Table (continued)

Atom	x	y	z
C	1.1631	-0.2599	-1.7240
H	1.0035	-1.3372	-1.6252
H	1.7572	-0.1194	-2.6301
C	-1.5873	0.4612	1.3465
H	-2.3187	1.2248	1.1052
C	1.9838	0.2224	-0.5969
C	2.7238	0.6614	0.2609
H	3.3751	1.0810	0.9903
O	-1.3354	0.4162	2.6841
H	-0.5709	-0.1664	2.8289
O	1.1559	-0.9727	2.7354
H	1.7542	-0.7058	3.4394
H	1.5183	-0.5772	1.9324

TS_{conia}(1W) of (*E*)-hept-1-en-6-yn-1-ol			
Theory level: CCSD/6-31G(d,p)			
Energy [Hartrees]	-423.074653		
No. Im. Freq.	1		
Atom	x	y	z
C	-1.4325	-0.2621	-1.1528
H	-1.6293	-1.1843	-1.7027
H	-2.3806	0.2739	-1.0678
C	-0.8616	-0.5861	0.2037
H	-0.4955	-1.5852	0.4015
C	-0.4031	0.5966	-1.9144
H	-0.5819	0.5923	-2.9914
H	-0.4735	1.6318	-1.5711
C	0.9976	0.0705	-1.5912
H	1.1141	-0.9439	-1.9835
H	1.7700	0.6932	-2.0487
C	-0.9804	0.2570	1.2775
H	-1.4958	1.2157	1.1829
C	1.2341	0.0510	-0.1244
C	1.8300	0.1499	0.9816
H	2.8177	0.3445	1.3569
O	-0.2789	0.0501	2.3723
H	0.8011	0.0639	1.9522
O	0.2767	-2.8652	2.3446

Continued on next page

Table (continued)

Atom	x	y	z
H	1.2118	-2.7209	2.1727
H	-0.0357	-1.9891	2.6073

3b + H_2O			
Theory level: CCSD/6-31G(d,p)			
Energy [Hartrees]		-423.193081	
No. Im. Freq.		0	
Atom	x	y	z
C	-1.2561	-0.3811	-1.2361
H	-1.1854	-1.2737	-1.8612
H	-2.3175	-0.1786	-1.0770
C	-0.4855	-0.6292	0.0786
H	-0.5073	-1.6678	0.4092
C	-0.5111	0.7856	-1.9048
H	-0.7270	0.8676	-2.9704
H	-0.8011	1.7325	-1.4406
C	0.9656	0.4874	-1.6068
H	1.3571	-0.2188	-2.3458
H	1.6012	1.3733	-1.6342
C	-1.0204	0.2341	1.1963
H	-1.3333	1.2528	0.9064
C	0.9447	-0.1712	-0.2377
C	1.9787	-0.3448	0.5917
H	2.9711	0.0024	0.3320
O	-1.1079	-0.1104	2.3646
H	1.8580	-0.8702	1.5295
O	0.4103	-2.5571	2.7875
H	0.7311	-2.6227	3.6908
H	-0.1910	-1.8003	2.7980

6.8 Hept-6-ynal + H_2O implicit solvent

Hept-6-ynal (1b) + H_2O			
Theory level: CCSD-SMD(H_2O)/6-31G(d,p)			
Energy [Hartrees]	-423.153983		
No. Im. Freq.	0		
Atom	x	y	z
C	-0.8059	0.3184	-0.2935
H	-1.5577	-0.1823	-0.9107
H	-1.3132	1.1363	0.2252
C	-0.2742	-0.6660	0.7643
H	0.1339	-1.5579	0.2898
C	0.2712	0.9231	-1.1971
H	-0.2134	1.6604	-1.8413
H	1.0024	1.4635	-0.5904
C	1.0017	-0.0770	-2.1099
H	0.2762	-0.7593	-2.5605
H	1.4689	0.4698	-2.9318
C	-1.4015	-1.0752	1.6646
H	-2.0538	-1.8839	1.3055
C	2.0529	-0.8618	-1.4367
C	2.9455	-1.4895	-0.9028
H	3.7178	-2.0456	-0.4213
O	-1.6492	-0.5343	2.7396
H	-0.5836	1.1291	2.9730
O	-0.0476	1.9353	2.9520
H	-0.1914	2.2646	2.0580
H	0.5014	-0.1898	1.3635

TS_{enol}(1W) of hept-6-ynal			
Theory level: CCSD-SMD(H_2O)/6-31G(d,p)			
Energy [Hartrees]	-423.077152		
No. Im. Freq.	1		
Atom	x	y	z
C	-1.1241	-0.0598	-0.8141
H	-1.4148	-0.9324	-1.4122
H	-2.0071	0.5867	-0.7721
C	-0.7716	-0.4717	0.5989
H	-0.1063	-1.3237	0.7225
C	-0.0355	0.6972	-1.5867
H	-0.4728	1.0759	-2.5148
H	0.2917	1.5666	-1.0097

Continued on next page

Table (continued)

Atom	x	y	z
C	1.1894	-0.1437	-1.9830
H	0.8535	-1.0768	-2.4434
H	1.7579	0.3931	-2.7462
C	-1.6645	-0.2337	1.6441
H	-2.5422	0.3894	1.4085
C	2.1118	-0.4587	-0.8768
C	2.9132	-0.7003	0.0038
H	3.5956	-0.9189	0.7937
O	-1.4780	-0.5483	2.8769
H	0.1560	0.0566	3.0006
O	0.8743	0.4450	2.4227
H	0.9175	1.3945	2.6215
H	0.2855	0.3040	1.4753

(*E*)-hex-1-en-5-yn-1-ol (2a) + H_2O			
Theory level: CCSD-SMD(H_2O)/6-31G(d,p)			
Energy [Hartrees]		-423.140455	
No. Im. Freq.		0	
Atom	x	y	z
C	-1.2995	-0.1665	-1.0684
H	-1.4844	-1.1608	-1.4886
H	-2.2292	0.3988	-1.1777
C	-0.9637	-0.2944	0.3924
H	-0.1721	-0.9789	0.6727
C	-0.2162	0.5186	-1.9146
H	-0.5614	0.5469	-2.9510
H	-0.0942	1.5544	-1.5889
C	1.1536	-0.1843	-1.8968
H	1.0140	-1.2666	-1.9639
H	1.7193	0.1120	-2.7829
C	-1.5954	0.4035	1.3433
H	-2.4096	1.0843	1.1237
C	1.9850	0.1264	-0.7186
C	2.7119	0.4107	0.2132
H	3.3436	0.6652	1.0344
O	-1.3228	0.3619	2.6977
H	-0.5041	-0.1528	2.8344
O	1.1793	-0.9028	3.1030

Continued on next page

Table (continued)

Atom	x	y	z
H	1.5789	-0.1779	3.5996
H	1.5463	-0.7936	2.2163

TS_{conia}(1W) of (*E*)-hept-1-en-6-yn-1-ol			
Theory level: CCSD-SMD(H_2O)/6-31G(d,p)			
Energy [Hartrees]		-423.089698	
No. Im. Freq.		1	
Atom	x	y	z
C	-1.4507	-0.2570	-1.1785
H	-1.6318	-1.1708	-1.7463
H	-2.3967	0.2835	-1.1063
C	-0.9161	-0.6021	0.1855
H	-0.5401	-1.6011	0.3652
C	-0.4026	0.6115	-1.9003
H	-0.5673	0.6323	-2.9787
H	-0.4698	1.6371	-1.5301
C	0.9922	0.0667	-1.5848
H	1.0967	-0.9464	-1.9807
H	1.7674	0.6884	-2.0387
C	-1.0057	0.2446	1.2524
H	-1.4837	1.2200	1.1519
C	1.2480	0.0397	-0.1256
C	1.8063	0.1120	1.0018
H	2.7923	0.2733	1.4029
O	-0.3174	0.0142	2.3653
H	0.8038	0.0314	1.9243
O	0.4012	-2.8057	2.5763
H	1.2032	-2.7250	2.0480
H	0.0750	-1.8931	2.6019

3b + H_2O			
Theory level: CCSD-SMD(H_2O)/6-31G(d,p)			
Energy [Hartrees]		-423.205651	
No. Im. Freq.		0	
Atom	x	y	z
C	-1.2595	-0.3600	-1.2251
H	-1.2219	-1.2609	-1.8404

Continued on next page

Table (continued)

Atom	x	y	z
H	-2.3100	-0.1143	-1.0596
C	-0.4987	-0.6272	0.0916
H	-0.5255	-1.6704	0.4049
C	-0.4724	0.7705	-1.9038
H	-0.6820	0.8462	-2.9710
H	-0.7250	1.7296	-1.4438
C	0.9893	0.4181	-1.5996
H	1.3361	-0.3488	-2.2992
H	1.6712	1.2659	-1.6710
C	-1.0309	0.2156	1.2167
H	-1.3299	1.2417	0.9560
C	0.9360	-0.1627	-0.1998
C	1.9388	-0.2408	0.6815
H	2.9328	0.1153	0.4406
O	-1.1243	-0.1646	2.3803
H	1.7949	-0.6784	1.6615
O	0.1808	-2.7287	2.8775
H	1.0681	-2.4430	3.1205
H	-0.2937	-1.8931	2.7523
